# Mechanistically Driven Development of Kumada Catalyst-Transfer Polymerizations: A Rapid Injection NMR Study

**DOI:** 10.1021/acscatal.5c07930

**Published:** 2025-12-02

**Authors:** Seokmin Kang, Wentao Cen, Achyut Ranjan Gogoi, Jeanette Piña, Adhya Suresh, Fernando Ramirez, Osvaldo Gutierrez, Andy A. Thomas

**Affiliations:** † Department of Chemistry, 2655 Texas A&M University, College Station, Texas 77845, United States; ‡ Department of Chemistry and Biochemistry, University of California, Los Angeles, Los Angeles, California 90095, United States

**Keywords:** Kumada−Tamao−Corriu Cross Coupling, Palladium Catalyzed Cross Coupling, Transmetalation, Catalyst Transfer Polymerization, Biarylmonophosphine Ligand, Rapid Injection NMR Spectroscopy

## Abstract

Transmetalation is a pivotal step in the Kumada–Tamao–Corriu cross-coupling reaction, yet its mechanistic details have remained relatively unexplored compared to other palladium-catalyzed processes. Herein, we systematically investigate how diverse phosphine ligands influence the transmetalation rate, using rapid injection NMR (RI-NMR) to directly monitor the formation of the cross-coupling product. The study reveals that both ligand electronic and steric effects significantly affect *k*_obs_, with electron-rich ligands tending to slow down the reaction and heteroatom-substituted ligands (CPhos) dramatically accelerating transmetalation. Computational studies suggest that the relatively lower transmetalation transition state barriers for CPhos over SPhos oxidative addition complexes are due to both increased electrophilicity of the Pd-atom as well as the ability to access a more favorable square-pyramidal transition state geometry. Finally, we leverage these kinetic findings to guide the synthesis of poly­(3-hexylthiophene) (P3HT) by catalyst-transfer polymerizations. Fast transmetalation ligands, such as CPhos, give higher-molar-mass polymers with controlled dispersities, whereas slower ligands afford inferior control. These results collectively emphasize the centrality of transmetalation in dictating overall cross-coupling performance and pave the way for the rational design of palladium catalysts for both small-molecule synthesis and advanced polymer applications.

## Graphical abstract

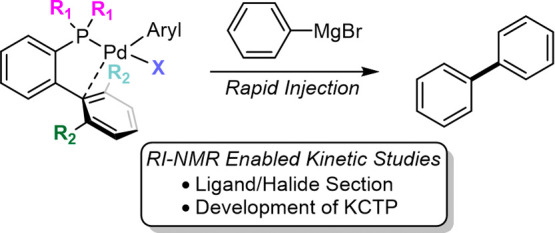

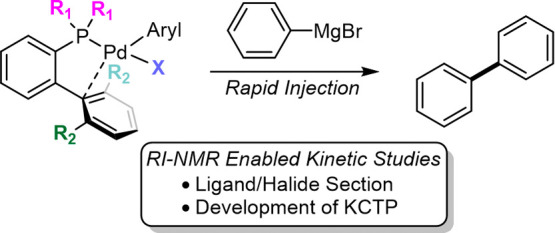

## 1 Introduction

Conjugated polymers are used in several organic electronic devices such as field effect transistors (OFETs),^1^ light-emitting diodes (OLEDs),^2^ and photovoltaic cells (OPVs).^3^ Since device performance is highly dependent on the organic material’s molar mass, dispersity, and end-group composition, controlled polymerization procedures are required. Currently, most commercial syntheses of all-conjugated polymers rely on step-growth methods, which can make it difficult to control these parameters. To circumvent these issues catalyst transfer polymerizations (CTPs), originally developed by McCullough^4^ and Yokozawa,^5^ have continued to gain in popularity due to their ability to control all of these parameters simultaneously. Mechanistically, CTPs follow the same basic catalytic cycle of typical transition metal catalyzed cross-coupling reactions; however, their distinguishing feature is that the oxidative addition occurs through a ring walking mechanism that provides an avenue for living polymerizations to be achieved (Figure 1).^6,7^ For example, nickel catalyzed systems using precatalysts, such as ((Ph_2_PCH_2_)_2_CH_2_)­NiCl_2_ (dpppNiCl_2_), have been shown to be very successful at ring walking and as a result at preparing various materials.^4,5^ Despite these achievements depending on the monomer used, these systems are often unpredictable and facetious in nature, making the preparation of uniform materials routinely on scale challenging. This is the result of many reasons; however, two major contributing factors that influence propagation are (1) the catalyst’s affinity for the π-surface of the growing polymer and (2) the ability for the catalyst to undergo productive turnover^8^ (Figure 1). Even if effective ring walking can be achieved, the dispersity and average molar mass can be greatly impacted if the turnover limiting step (often transmetalation) is slow. To address these problems numerous studies have emerged focused on expanding the polymer scope through modulating the reactivity of the transmetalation species via tuning the organic donor, e.g., Murahashi,^9a^ Kumada,^5^ Negishi,^4^ Suzuki-Miyaura,^20^ Stille,^9b^ etc. Although the ability to swap the identity of the organic donor clearly has its preparative advantages, its variation (Li, Mg, Zn, B, and Sn) naturally leads to diverse mechanistic pathways. As a result, far less is known about the individual transmetalation processes.^10^ Accordingly, the *a priori* prediction of the catalyst efficiency for any specific monomer remains a challenging objective. Considering the inherent attractive features of palladium catalyzed (e.g., higher tolerance to oxygen over Ni, number of commercially available catalysts, etc.),^6c^ Kumada couplings (relatively fast transmetalation) encouraged an in-depth investigation into the transmetalation step for applications in catalyst transfer polymerization reactions. The study described herein focuses on elucidating the structural features that influence the reactivity of phosphine ligated palladium oxidative complexes (OACs) with Grignard donors toward transmetalation by rapid injection nuclear magnetic resonance (RI-NMR) spectroscopy. Detailed rate information is provided with a diverse range of OACs bearing various phosphine ligands, allowing for insights into the molecular principles that lead to favorable transmetalation events to be revealed. Specifically, we demonstrate that the Buchwald ligand CPhos exhibited enhanced transmetalation rates (ca. 3 orders of magnitude over SPhos) and report the successful application of CPhos-derived palladium catalysts to promote Kumada catalyst transfer polymerization reactions for the synthesis of poly­(3-hexylthiophene) (P3HT).

**Figure 1:**
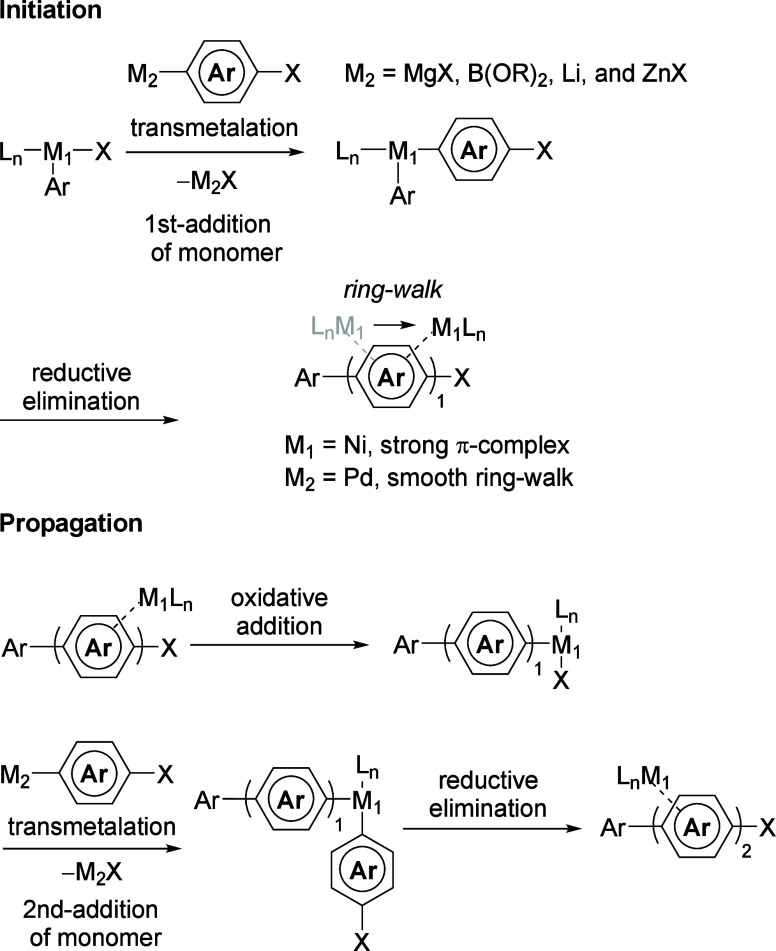
General scheme for catalyst transfer polymerization.

## 2 Background

### 2.1 Rapid Injection NMR

To investigate the transmetalation step, our rapid injection NMR (RI-NMR) apparatus was called into action. Originally developed by McGarrity and co-workers in 1981, RI-NMR enables the observation and characterization of short-lived reaction intermediates as well as detailed kinetic analysis.^11^ In layman’s terms one substrate is placed inside the NMR tube which is then lowered into the probe within the NMR spectrometer and the second reagent is introduced via a calibrated syringe assembly. Data collection starts immediately upon the addition of the reagent, enabling real-time observation and quantification of transient species. This approach provides several advantages: (1) inert atmospheres can be used, which allows for air and moisture sensitive reactions to be investigated, mimicking a Schlenk flask inside the spectrometer; (2) detailed kinetics can be obtained inside the NMR spectrometer, allowing for structural and kinetic data to be linked; and (3) intermediate species can be identified and structurally characterized. Of note, because the rates of transmetalation are unknown and likely to be greatly affected by the identity of the supporting phosphine ligands RI-NMR is particularly well suited to detect and track fast (seconds)^12^ and slower (minutes to hours) kinetic profiles.

### 2.2 Mechanistic Investigations on Kumada–Corriu Cross Coupling in Previous Studies

A detailed understanding of transmetalation in the Kumada–Corriu reaction remains a challenging pursuit.^13b,7b^ This complexity largely stems from the behavior of Grignard reagents, which can participate in Schlenk equilibria and may follow heterobimetallic pathways.^13,14^ Consequently, most investigations rely on overall reaction yields, rather than isolated transmetalation steps, to assess and optimize the complete catalytic cycle.^14^ Because nickel was the first transition metal employed in the Kumada–Corriu reaction, mechanistic and methodological studies have predominantly focused on Ni-based systems.^15^ Notably, Ni’s lower electronegativity compared to Pd facilitates coupling with less reactive electrophiles such as aryl fluorides.^16^ Computational work suggests that Ni/Mg bimetallic cooperation activates the strong C–F bond (∼126 kcal/mol) through Lewis acid activation, wherein Mg binds to fluorine.^13a^ In parallel, the McNeil group described ligand dependence on the rate-limiting steps by characterizing resting state speciation of the catalytic cycle via ^31^P NMR spectroscopy,^24a,24b^ further demonstrating that electronic perturbations of the ligand manifold substantially modulate the kinetics of Ni-catalyzed chain-growth polymerizations.^24c^ In contrast, recent attention has turned to Pd-based Kumada–Corriu reactions, largely due to the vast array of ligands developed for Pd-catalyzed cross-couplings. Interestingly, Tomson and Organ independently demonstrated Pd-catalyzed reactions using DavePhos and PEPPSI ligands, respectively, identifying the turnover-limiting step as oxidative addition for DavePhos and transmetalation for PEPPSI—highlighting the critical role of ligand design.^13b,17^ Both studies also underscored the importance of Grignard reagents at each stage of the catalytic cycle. Motivated by these developments, we focused on elucidating the mechanistic details of Pd-catalyzed Kumada–Corriu reactions, with particular emphasis on the rate of transmetalation, where the unique properties of Grignard reagents are pivotal.

### 2.3 Current Progress on Pd-Based Catalyst Transfer Polymerization

Building on Pd’s extensive use in small-molecule cross-couplings, Pd precatalysts have increasingly been explored in catalyst-transfer polymerization (CTP).^6c^ McNeil’s work using a Pd-PEPPSI system, [1,3-bis­(2,6-diisopropylphenyl)­imidazol-2-ylidene]­(3-chloropyridyl)­palladium­(II) dichloride, demonstrated notable improvements in the molar mass and dispersity of polymers synthesized via KCTP (Figure 2A).^18^ Yokozawa’s group introduced an ArPd­(*t*Bu_3_P)I system designed to reduce undesired catalyst migration from the Pd center on the π-face of one substrate to the C=C bond of another in Suzuki catalyst transfer polymerization (SCTP) (Figure 2B).^19a^ More recently, Choi and co-workers employed Pd-based oxidative addition complexes bearing Buchwald ligands, leveraging well-established ligand libraries and employing *N*-methylimidodiacetic (MIDA) boronate protection to prevent protodeboronation in the monomer (Figure 2C).^20a^ Notably, both the Yokozawa and Choi strategies utilize externally initiated catalysts (i.e., oxidative addition complexes), enabling efficient initiation and preserving end-group integrity, thus offering valuable tools for advanced polymer synthesis.

**Figure 2:**
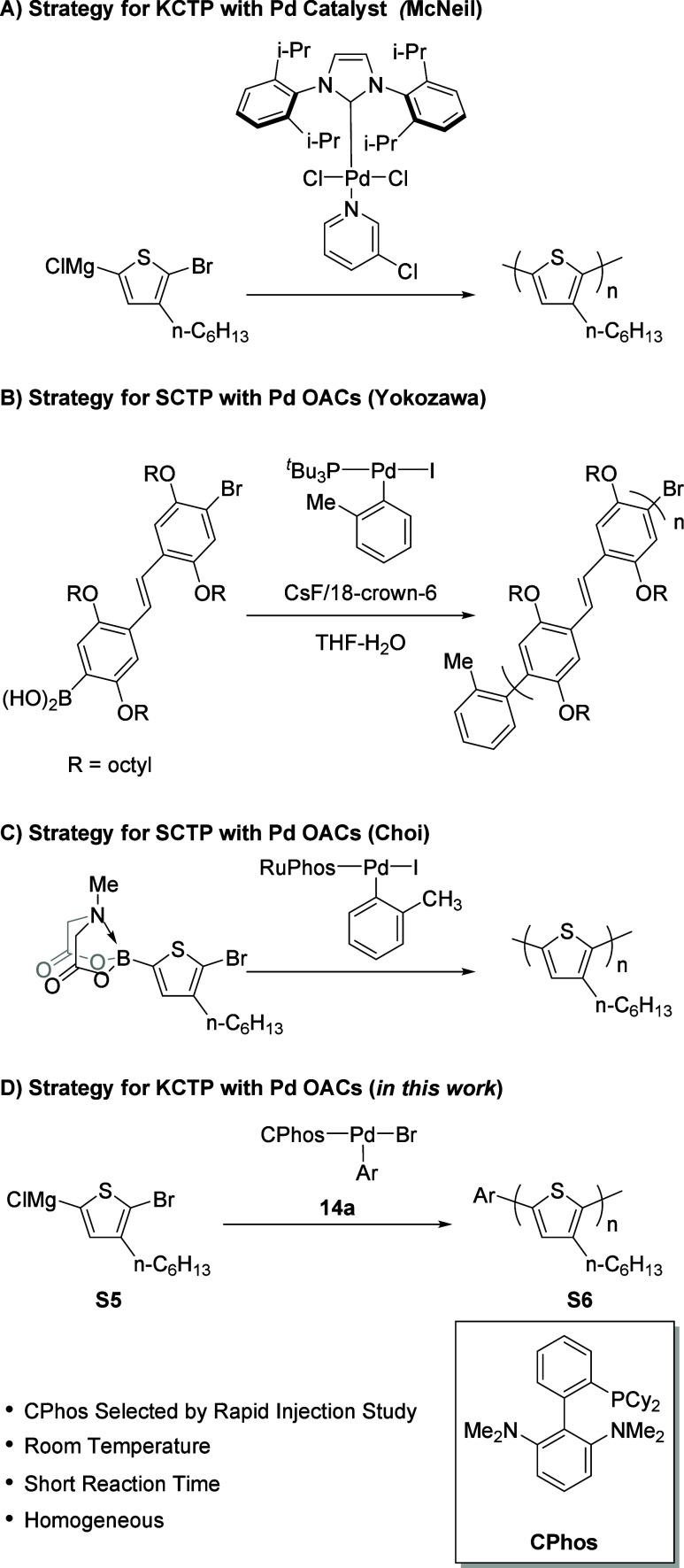
Strategies for the catalyst transfer polymerization with a controlled chain-growth mechanism: (A) KCTP in previous works; (B) and (C) SCTP in previous works; (D) this work of KCTP with Pd-CPhos oxidative addition complex guided by rapid injection experiments.

Alongside the synthetic development of CTP, comprehensive mechanistic investigations have been undertaken. In SCTP, Yokozawa’s work elucidated that CsF promotes *in situ* formation of an Pd–F intermediate that facilitates transmetalation with organoboron compounds.^19b^ Complementarily, Choi’s group executed a systematic parametrization of SCTP components—boron reagents, halide, and ligand classes—to optimize polymerization outcomes.^20b,20c^

## 3 Goals of This Study

The overarching goal of the study is to determine how the structure of oxidative addition complexes influences the transmetalation step in Kumada–Corriu cross-coupling reactions, employing both RI-NMR spectroscopy and DFT computational methods. By clarifying these structure–reactivity relationships and tabulating the corresponding kinetic parameters, we aim to guide future catalyst-transfer polymerization strategies. The specific goals of the study are as follows: (1) investigate stoichiometric reactions of preformed palladium oxidative addition complexes with Grignard reagent and determine how phosphine ligands impact the rate transmetalation, (2) examine the effect of various halides (X = Cl, Br, or I) on oxidative addition complexes in modulating transmetalation kinetics, (3) evaluate the kinetic parameters to guide the development of an improved catalyst transfer polymerization methods, and (4) obtain computational insights into the transmetalation Step. Ultimately, this study will highlight the critical role of transmetalation in determining the overall efficiency of the Kumada–Corriu cross-coupling reactions and pave the way for the rational development of Pd catalysts in KCTP.

## 4 Results and Discussion

### 4.1 Preparation of Oxidative Addition Complexes

In order to obtain detailed kinetic information about the transmetalation step in Kumada cross-coupling reactions, our studies were directed toward the preparation of fluorine labeled aryl palladium halide phosphine complexes (or oxidative addition complexes, OACs) such that the ^19^F and ^31^P NMR signals could be monitored. A series of monodentate and chelating phosphine OACs were synthesized and explored as described below. Following a recently developed procedure by Buchwald,^21^ Campora’s Palladacycle **S2** was treated with the desired phosphine ligand in the presence of 1-bromo or 1-chloro or 1-iodo-4-fluorobenzene (Scheme 1A). This method proved to be well suited to prepare T-shaped aryl palladium halide complexes (**3a**, **4a**, **6a**, **8a**, **8b**, **9a**, **11a**, **11b**, **11c**, **13a**, **14a**, **14b**, and **14c**) ligated by Buchwald ligands as well as simple phosphines such as *i-*Pr_3_P (**15**). However, bis-ligating phosphine ligands were found to not be amenable to this process due to failure in the activation (reductive elimination) step of Campora’s complex. This is presumably owing to the binding of two phosphines to the Pd center, which hampered the reduction to form the active Pd(0) species. As a result, our efforts shifted to a thermodynamic ligand exchange process by mixing *trans*-(Ph_3_P)_2_(4-F-C_6_H_4_)­PdBr and the described bis-phosphine ligand (Scheme 1B). Of note, in all cases upon employing a chelating ligand, the two Ph_3_P ligands were successfully replaced with the desired ligand (**7**, **10**, **12**, **16**). With a range of OACs bearing diverse phosphine ligands in hand, our efforts shifted to investigating how structure affects the transmetalation step.

**Scheme 1:**
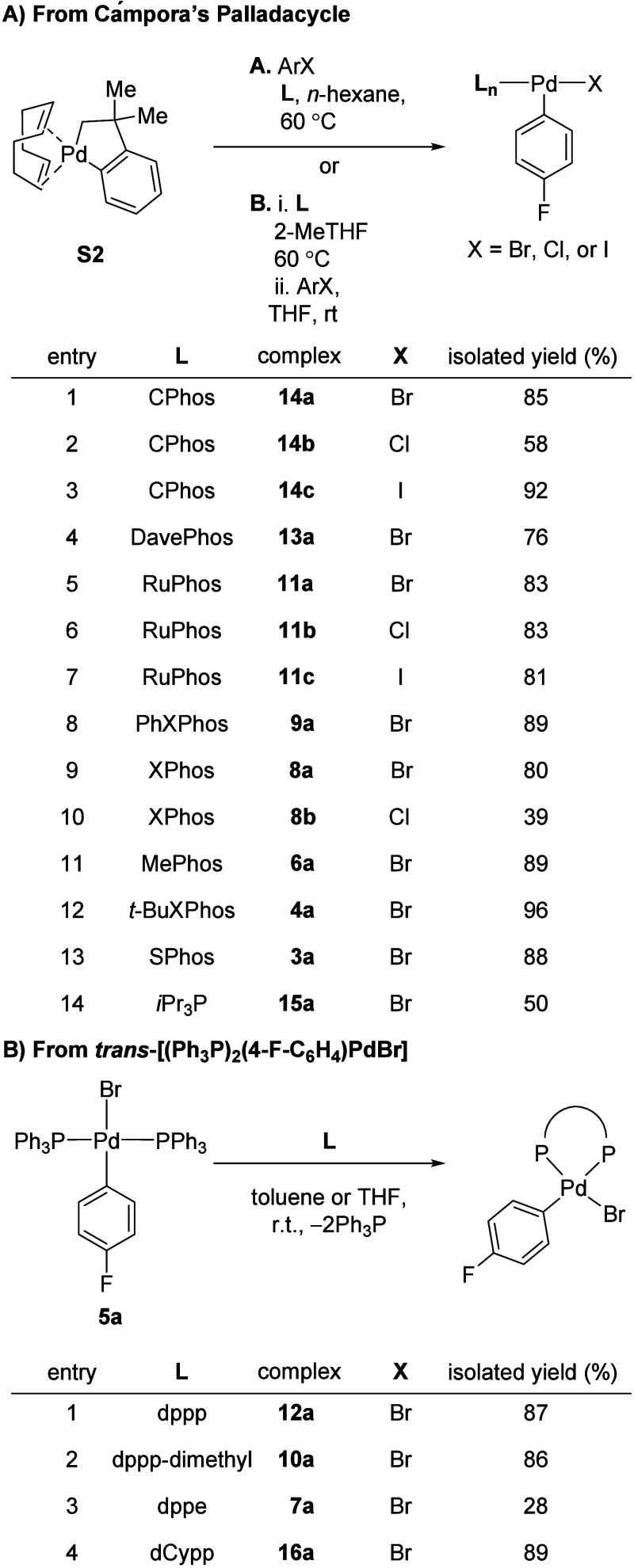
Synthesis of Oxidative Addition Complexes (A) from Palladacycles and (B) from *trans*-[(Ph_3_P)_2_(4-F-C_6_H_4_)­PdBr]

### 4.2 Kinetic Analysis of Transmetalation

To establish the effect of phosphine on the rate of transmetalation, complexes **3a**–**16a** were combined separately with 3-F-C_6_H_4_MgBr (12.0 equiv/Pd) at −10 °C under pseudo-first-order conditions such that their ^19^F NMR signals could be monitored. Of note, each of the OAC described below exhibited first-order kinetics, providing accurate values for *k*_obs_ (Table 1). Specifically, the effects of the ligand (Pd–L) and halide (Pd–X) were probed by monitoring the formation of the cross-coupling product (**2**) by ^19^F NMR spectroscopy. To allow for a straightforward comparison of the phosphine ligands, the rates were normalized to the complex supported by the biaryl monophosphine ligand SPhos (**3**), which showed slowest rate (Table 1, entry 1). We first investigated how Buchwald ligands (biaryl monophosphine ligands) affect the rates of transmetalation.

**Table 1:**
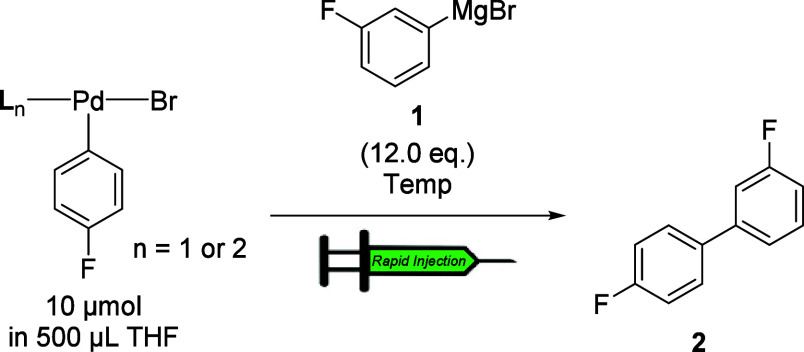

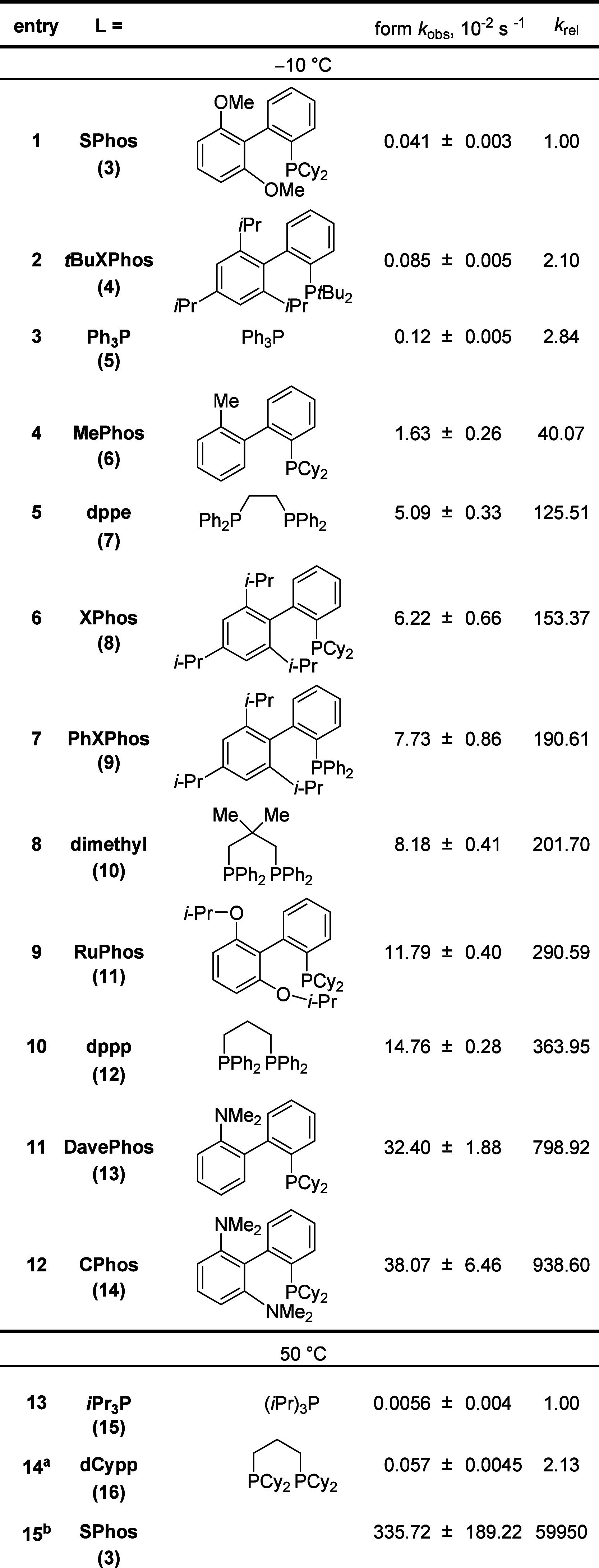
Effect of the Phosphine Ligands on the Rate of Transmetalation

a The *k*_obs_ was obtained by observing consumption of OAC.

b *k*_obs_ at 50 °C was extracted from Eyring analysis by extrapolation.

#### 4.2.1 Effect of Biaryl Monophosphines on the Transmetalation Step

##### 4.2.1.1 Investigations of BiAryl-PCy_2_ Oxidative Addition Complexes

The RuPhos complex (**11a**), bearing isopropoxy groups (−O*i*Pr) instead of methoxys, forged the cross-coupling product (**2**) ca. 290 times faster than the SPhos complex (**3a**) indicating that larger substituents at the southern ring led to faster transmetalation rates. To shed light on this possibility, the XPhos complex (**8a**) bearing isopropyl groups was subjected to the standard reaction conditions where the cross-coupling product (**2**) was observed ∼153 times faster than **3a** (ca. 2 times slower than **11a**). These combined results clearly indicated that the rates of the transmetalation event are influenced not only by the steric structure of the ligand but also by the electronic properties of it as well. Therefore, our efforts became focused on exploring phosphine ligands bearing other heteroatomic subunits. DavePhos complex (**13a**), a well-known ligand for Kumada cross-coupling reactions at low temperatures,^22a^ provided the cross-coupling product (**2**) nearly 800 times faster than (**3a**), clearly suggesting the dimethylamino group was playing a positive role in the transmetalation event. Moreover, the CPhos complex (**14a**), bearing two dimethylamine groups, was nearly 3 orders of magnitude (ca. 938 times) more reactive than the SPhos complex (**3a**).^22b^

##### 4.2.1.2 Investigations of Biaryl-PR_2_ Oxidative Addition Complexes

As described *vide supra*, the XPhos complex (**8a**) was roughly 153 times faster than the SPhos complex (**3a**) indicating that larger steric properties at southern ring can lead to positive influences on the transmetalation step. Therefore, we became interested in exploring the steric and electronic properties of the alkyl substituents bound to phosphorus. The *t-*BuXPhos complex (**4a**) was significantly slower (roughly 75 times slower) than the XPhos complex (**8a**). Of note, the PhXPhos complex (**9a**) provided comparable rates to the XPhos complex (**8a**) for transmetalation.

##### 4.2.1.3 Investigations of L_2_Pd­(4-C_6_H_4_-F)Br Complexes

Next, we turned our attention to bidentate ligands with carbon linkers—1,3-bis­(diphenylphosphino)­propane (dppp, **12**), 1,2-bis­(diphenylphosphino)­ethane (dppe, **7**), and a dppp derivative containing two geminal methyl groups (dppp-dimethyl, **10**). The only structural difference between dppp (**12**) (*k*_obs_ = 14.76 × 10^–2^ s^–1^) and dppe (**7**) (*k*_obs_ = 5.09 × 10^–2^ s^–1^) is the addition of one methylene unit in the former, yet it results in an approximately 3-fold faster rate. The dppp-dimethyl (**10**) ligand with two geminal methyl groups gave an intermediate value of 8.18 × 10^–2^ s^–1^. These differences are likely attributable to variations in bite angle (dppp (**12**), 91° > dppe (**7**), 84.4°) and more rigid binding of dppp-dimethyl (**10**).^23a,23b^ This ligand effect on the Kumada cross-coupling reaction was also pronounced in previously studies by McNeil with the rate-limiting step with Ni­(dppp)­Cl_2_ being transmetalation and that with Ni­(dppe)­Cl_2_ being reductive elimination.^24a,24b^ We also tested trialkylphosphines, including triisopropylphosphine (*i*Pr_3_P, **15**) and 1,3-bis­(dicyclohexylphosphino)­propane (dcpp, **16**), which barely converted to product at −10 °C. Even at 50 °C, *i*Pr_3_P (**15**) remained ∼60000 times slower than SPhos (**3**) at the same temperature. (*k*_obs_ for SPhos at 50 °C extracted from Eyring analysis), confirming that electron-rich phosphines tend to suppress the Kumada cross-coupling. Additional experiments with the CPhos complex (**14a**) (fastest) examined the initial rate dependence on the relative concentration of a single species to determine the reaction order. The results confirmed that both CPhos complex (**14a**) and the Grignard reagent (**1**) appear in the rate law with partial orders near unity, strongly suggesting that transmetalation is the rate-limiting step. (See the Supporting Informaiton for rate determination.) This is because transmetalation involves the simultaneous interaction of both the OAC ligand and the Grignard reagent, making their contributions critical to the overall reaction rate.

#### 4.2.2 Effect of Halides on the Rate of Transmetalation

Next, we examined the effect of halides with complexes supported by CPhos, RuPhos, and XPhos to gain more practical insights into transmetalation behavior (Table 2). The CPhos complex (**14a**) reacted five times faster than the iodide analogous complex (**14c**), while the chloride complex (**14b**) displayed intermediate reactivity. In contrast, chloride Pd-complex with RuPhos (**11b**) or XPhos (**8b**) was the fastest in transmetalation matching previous rationale from the Buchwald group that enhanced polarity of Pd–Cl bonds and the smaller size of Cl atom facilitate transmetalation.^25^

**Table 2:**
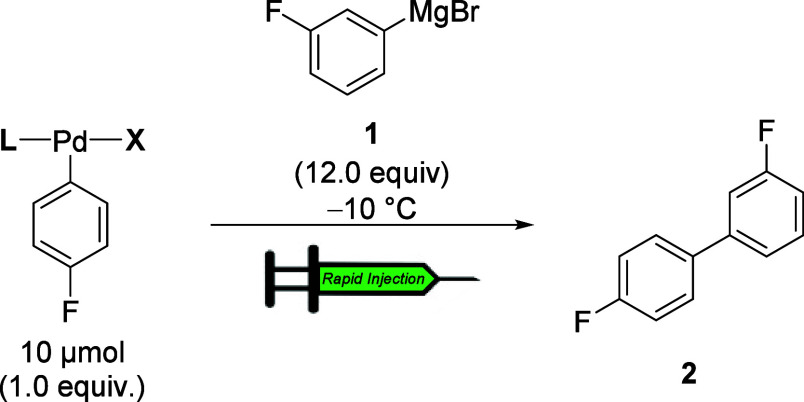
Effect of the Halides on the Rate of Transmetalation

entry	L	X	form *k*_obs_, 10^–2^ s^–1^	*k*_rel_
1	CPhos	I	3.55 ± 0.53	1.00
2	CPhos	Cl	12.69 ± 1.03	3.58
3	CPhos	Br	19.93 ± 1.73	5.62
4	RuPhos	I	4.50 ± 1.13	1.00
5	RuPhos	Br	11.79 ± 0.40	2.62
6	RuPhos	Cl	19.61 ± 1.02	4.36
7	XPhos	Br	6.22 ± 0.66	1.00
8	XPhos	Cl	21.58 ± 0.43	3.47

#### 4.2.3 Eyring Analysis of Transmetalation with Different Ligands

To understand the reaction mechanism further, we conducted Eyring analysis on three ligands: CPhos (**14**) (fastest), Ph_3_P (**5**) (traditional benchmark), and SPhos (**3**) (slowest) (Table 3). CPhos (**14**) showed a negative entropic contribution at its transition state, suggesting strong binding interactions in the pre-transmetalation complex. In the case of SPhos (**5**), a positive entropy value suggests that the transition state of transmetalation requires an increment of disorder in the complex. Presumably, there is a strong binding between −OMe and Pd center.

**Table 3:**
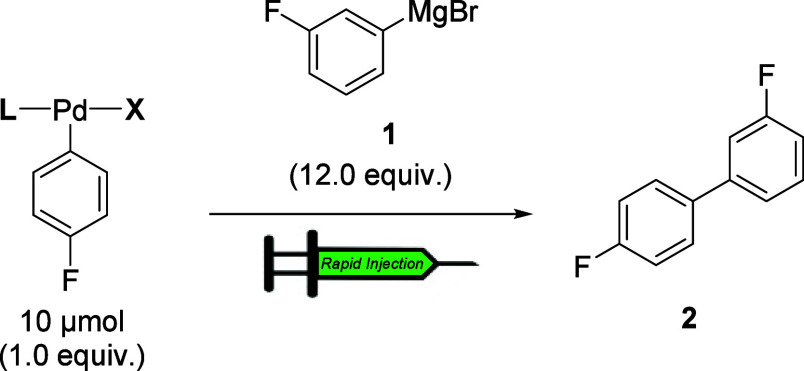
Activation Parameters for the Transmetalation

entry	L	Δ*G* ^⧧^_263.15_, kcal/mol	Δ*H* ^⧧^, kcal/mol	Δ*S* ^⧧^, kcal/mol·K
1	SPhos	19.44 ± 0.05	24.74 ± 1.55	0.020 ± 0.0057
2	PPh_3_	18.91 ± 0.84	14.68 ± 0.43	–0.016 ± 0.0016
3	CPhos	15.88 ± 0.50	3.62 ± 0.25	–0.047 ± 0.00097

### 4.3 Computational Insights into the Transmetalation Step

To gain insights into the origin of the observed transmetalation rate differences, we employed dispersion-corrected density functional theory (DFT-D3) to model the corresponding transmetalation transition states for three representative oxidative addition complexes: SPhos (**3a**), RuPhos (**11a**), and CPhos (**14a**). Given the challenges associated with accurately modeling Grignard reagents,^29,14a,30^ we adopted a simplified model in which the ground state of aryl magnesium bromide is represented as a monomeric species coordinated by two THF ligands around the magnesium center. Overall, the computed relative transition state barriers for transmetalation between oxidative addition complexes and the aryl Grignard reagent were in agreement with the observed experimental trends (Figure 3). A closer inspection of the transition states revealed that notable correlations between relative rates and atomic charge and electrophilicity at the Pd-center were present. Specifically, we observed that the pre-transmetalation CPhos complex (**14a**) possessed the most positive Pd charge (Hirshfeld, CM5, and NBO analyses) and highest electrophilicity followed by RuPhos (**11a**) and SPhos (**3a**). Presumably, this increased positive character enhances the susceptibility of the Pd center toward nucleophilic attack by aryl Grignard reagents, thereby lowering the transmetalation barrier for **14a**.

**Figure 3:**
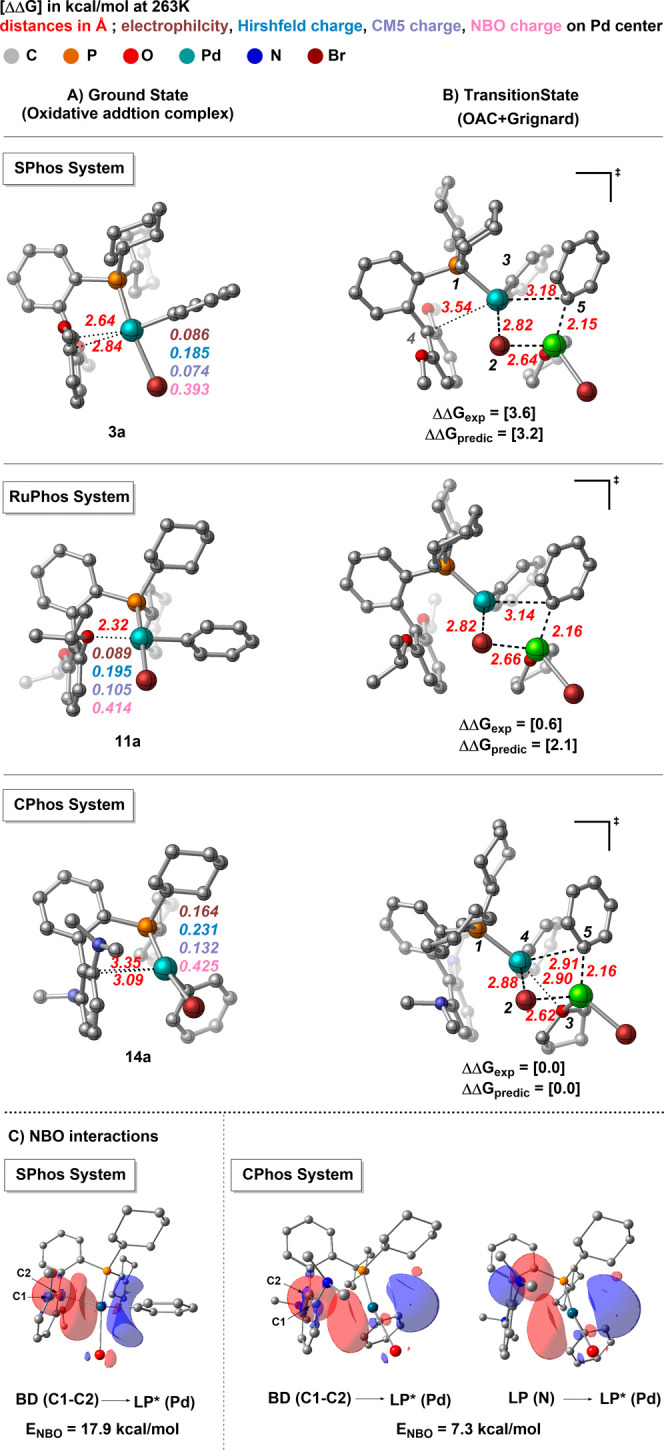
(A) Atomic charge and electrophilicity analysis for the SPhos, RuPhos and CPhos-ligated oxidative addition complexes. (B) Transmetalation transition states for SPhos (atoms 1–5 for trigonal-bipyramidal-like geometry), RuPhos and CPhos (atoms 1–5 for square-pyramidal geometry) ligated system. (C) Key NBO interactions between ligand and Pd center in the SPhos and CPhos system Calculations are done at RM06L-D3/6-311+g­(d,p)-SDD­(Pd,Br)-CPCM­(THF)//RM06L-D3/6-31g­(d,p)-LANL2DZ­(Pd,Br)-CPCM­(THF) level of theory. [ΔΔ*G*] in kcal/mol at 263 K, distances in Å; electrophilicity (brown), Hirshfeld charge (turquoise), CM5 (lavender), and NBO charge (pink) on Pd center.

Further, natural bond orbital (NBO) analysis revealed how ligand-specific interactions could influence the Pd electrophilicity. In the SPhos complex **3a**, the Pd center engages in significant π-donation from the aromatic C–C bonds (total interaction energy, *E*_NBO_ = 17.9 kcal/mol; Figure 3C), which increases electron density at the Pd-atom and, in turn, diminishes its positive charge and reduces electrophilicity, consequently increasing the transmetalation barrier. In contrast, the CPhos complex **14a** exhibits weaker donor interactions, with reduced electron donation from both aromatic π orbitals and the amine lone pairs (*E*_NBO_ = 7.3 kcal/mol, Figure 3C), leaving the Pd center comparatively electron-deficient and more electrophilic. NBO analysis in transition state also follows this trend (*E*_NBO_ = 9.6 kcal/mol for SPhos system, *E*_NBO_ = 6.7 kcal/mol for CPhos system, see Supporting Information, S115)

Moreover, these subtle electronic distinctions are reflected in the geometries of the transmetalation transition states. In SPhos complex **3a**, persistent Pd–aromatic interactions enforce a trigonal-bipyramidal-like geometry, directing the incoming Grignard reagent to approach from the opposite face under electronic and steric constraint. Conversely, in the CPhos complex **14a**, the Pd atom adopts a square-pyramidal geometry, allowing the axial approach of the nucleophile. This arrangement is electronically favorable, thus stabilizing the transition state and accelerating transmetalation.^31^ Collectively, these findings attribute the superior reactivity of **14a** to the higher electrophilicity of its Pd center and its ability to access a more favorable transmetalation transition state geometry.

### 4.4 Catalyst Transfer Polymerization— P3HT Synthesis

#### 4.4.1 Effect of Phosphine Ligands and Halides on Catalyst Transfer Polymerization Reactions

With the kinetic information in hand, we shifted our focus to evaluating catalyst performance in catalyst-transfer polymerization reactions (CTP) specifically for the synthesis of poly­(3-hexylthiophene) (P3HT). We chose CTP as the model reaction because transmetalation occurs in both the initiation and propagation steps, allowing us to assess catalyst behavior through number-averaged molar mass (*M*_n_) and dispersity (*Đ*) values. Using the oxidative addition complexes described above as the catalyst precursor provided several advantages: (1) if the initiator is bound to the surface of the material from which the polymer is growing, it can lead to surface-grafted conjugated polymers;^26^ (2) reactivity of the initiator can be tuned by modifying the structure of the ligand, aryl group, and halide ligands; and (3) the oxidative addition complex, L–Pd–ArX, enables unidirectional polymer growth,^27^ while initiators like Ni­(dppp)­Cl_2_, which require double additions of the monomer for initiation, may still result in bidirectional/random growth, causing tail-to-tail defects at unexpected locations. With this in mind, we begin with the CPhos complex (**14a**) which was found to have the fastest transmetalation rate of the complexes surveyed (Table 4, entry 10). Polymerization using **14a** was successfully performed, yielding polymers with a profile (*M*_n_ = 13.9 k, *Đ* = 1.37) competitive with known methods using PEPPSI ligands (*M*_n_ = 17.0 k, *Đ* = 1.25). By contrast, complexes shown to exhibit slower transmetalation rates such as SPhos (**3a**), Ph_3_P (**5a**), and MePhos (**6a**) either failed to form a polymer or yielded low-molar mass products. Notably, SPhos complex (**3a**), which is known from previous literature for its efficient ring-walking behavior in SCTP (Suzuki catalyst transfer polymerization), produced polymers with low molar masses.^20^ This is likely due to relatively slow transmetalation rates, which can hinder the propagation step of the polymerization process. Although ring-walking and oxidative addition are known to play crucial roles in CTP, our data underscore that transmetalation rates exert a critical influence on achieving a high molar mass and controlled dispersity. We further probed halide effects in XPhos- and RuPhos-based systems (**8a**, **8b**, **11a**, **11b**, and **11c**), finding that the polymer molar mass positively correlates with the transmetalation rate. Increasing the monomer-to-initiator ratio did not alter *M*_n_ (Table 4, entries 11 and 12), indicating a limit in the catalyst’s turnover number, which ultimately restricts polymer chain growth. This highlights the need for further catalyst development to overcome these limitations and achieve more efficient polymerization.

**Table 4:**
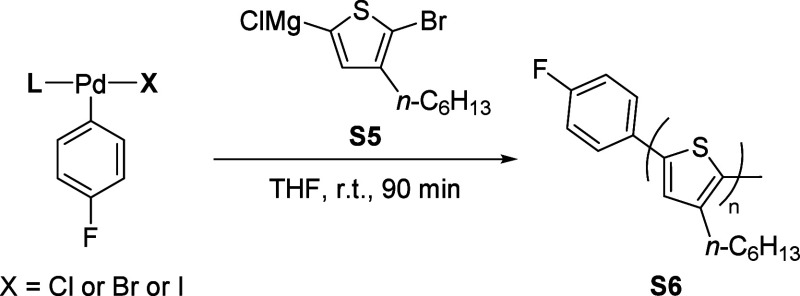
Catalyst Transfer Polymerization with Various L–Pd–X Initiators

entry	L	X	form *k*_obs_,^b^ 10^–2^ s^–1^	*M*/*I*	*M*_n_ (*Đ*)
1	SPhos	Br	0.041 ± 0.003	60	5.9 k (1.57)
2	PPh_3_	Br	0.12 ± 0.005	60	
3	MePhos	Br	1.63 ± 0.26	60	
4	XPhos	Br	6.22 ± 0.66	60	2.9 k (1.61)
5	XPhos	CI	12.69 ± 1.03	60	3.1 k (1.63)
6	RuPhos	I	4.50 ± 1.13	60	8.8 k (1.62)
7	RuPhos	Br	11.79 ± 0.40	60	10.8 k (1.42)
8	RuPhos	CI	19.61 ± 1.02	60	12.9 k (1.47)
9	dppp	Br	14.76 ± 0.28	60	1.5 k (1.12)
10	CPhos	Br	38.07 ± 6.46	60	13.9 k (1.37)
11^a^	CPhos	Br	38.07 ± 6.46	60	14.1 k (1.43)
12^a^	CPhos	Br	38.07 ± 6.46	120	14.0 k (1.58)
13	PEPPSI-Ipr			60	17.0 k (1.25)

a The reaction time was 15 h.

b Kinetic constants (*k*_obs_) measured at −10 °C.

#### 4.4.2 Product Distribution Experiment for Ring-walking Efficiency

The transmetalation rate alone does not fully explain the observed polymerization outcomes given that oxidative addition and ring-walking can also be critical steps. Moreover, prior studies show that the rate-limiting step in CTP can shift depending on the phosphine ligand, rendering transmetalation data alone insufficient for predicting polymerization efficiency.^24^ For example, despite the very slow transmetalation rate (*k*_obs_ = 0.041 × 10^–2^ s^–1^) of the OAC with SPhos (**6**), it still delivered a moderate polymer profile (*M*_n_ = 5.9 k, *Đ* = 1.57). Meanwhile, faster ligands such as Ph_3_P (**5**) and MePhos (**6**) (entry 2 and 3, Table 4) did not successfully form polymer. The observation led us to perform a product distribution experiment representing ring-walking efficiency.^28^ Under a substoichiometric amount of 4-fluorophenylmagnesium bromide, the monosubstituted product (**A1**) would be statistically favored, while significant formation of the disubstituted product (**A2**) indicates efficient intramolecular catalyst transfer (Table 5). Our results showed that most ligands tested (entries 1, 2, 3, and 5, Table 5) predominantly yielded **A2**, whereas dppp produced mainly **A1**. This difference explains why SPhos (**6**), despite its slow transmetalation (*k*_obs_ = 0.041 × 10^–2^ s^–1^), still produced moderate polymer, and why dppp—a faster transmetalating ligand (*k*_obs_ = 14.76 × 10^–2^ s^–1^)—nonetheless gave poor KCTP results. These findings illustrate that ring-walking and oxidative addition can also influence the polymerization performance, complementing the role of transmetalation.

**Table 5:**
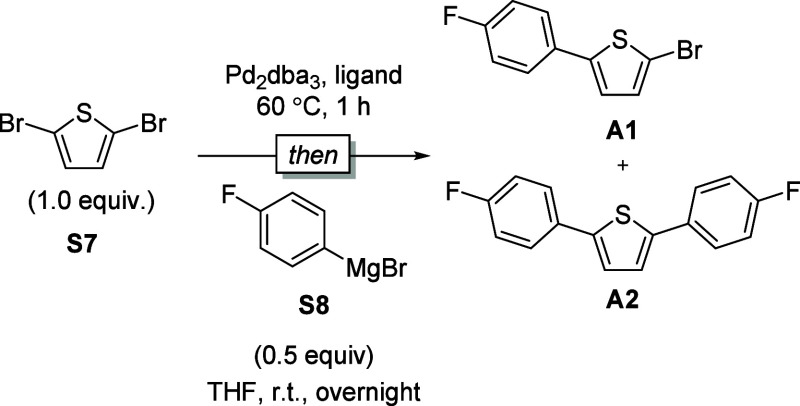
Screening of the Ligands on Ring-Walking Process in Small Molecules

entry	Pd (equiv)	ligand (equiv)	A1:A2^a^
1	Pd_2_dba_3_ (0.02)	SPhos (0.08)	6:94
2	Pd_2_dba_3_ (0.02)	XPhos (0.08)	15:85
3	Pd_2_dba_3_ (0.02)	RuPhos (0.08)	3:97
4	Pd_2_dba_3_ (0.02)	dppp (0.08)	94:4
5	Pd_2_dba_3_ (0.02)	CPhos (0.08)	9:91

a A1:A2 ratio was determined by ^19^F NMR spectroscopy against 1,4-difluorobenzene as an internal standard.

#### 4.4.3 Chain Extension Experiment in Catalyst Transfer Polymerization

Next, we investigated the living nature of polymerization using CPhos complex (**14a**) by adding a second aliquot of monomer after 3 h (Figure 4). The polymer’s *M*_n_ increased and its dispersity broadened slightly, indicating that most polymer chains remained active under the reaction conditions.^18^ These results suggest that this method could be extended to block copolymer synthesis and the incorporation of various functional end groups, thereby broadening the scope of catalyst-transfer polymerization.

**Figure 4:**
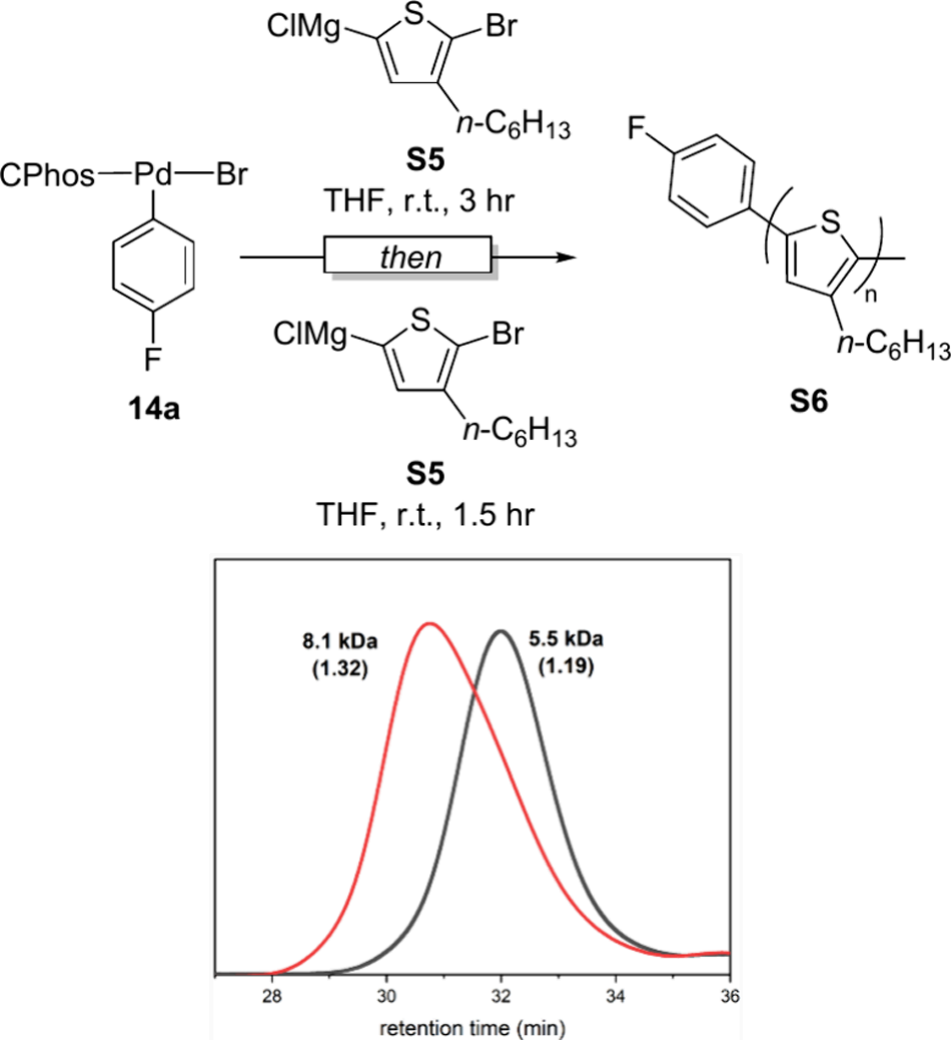
Chain extension experiment of P3HT synthesis and the SEC profile. SEC profile after 3 h from first addition of monomer (black) and after 1.5 h from second addition of monomer (red).

#### 4.4.4 Reaction Monitoring in Catalyst Transfer Polymerization

To elucidate the chain growth mechanism, the polymerization reaction was monitored at 10 min intervals, observing *M*_n_ and dispersity (Figure 5). The number-average molar mass (*M*_n_) increased in proportion to the conversion of the monomer, whereas the dispersity remained narrow throughout the polymerization (Figure 5A). Additionally, semilogarithmic kinetic plot showed linearity, further supporting a chain-growth mechanism (Figure 5B). Of note, the polymerization reached to ∼95% conversion at 40 min (fast), which highlights the advantage of Kumada-type CTP over other types of reactions such as Suzuki–Miyaura and Negishi catalyst-transfer polymerization often requiring elongated reaction time.

**Figure 5:**
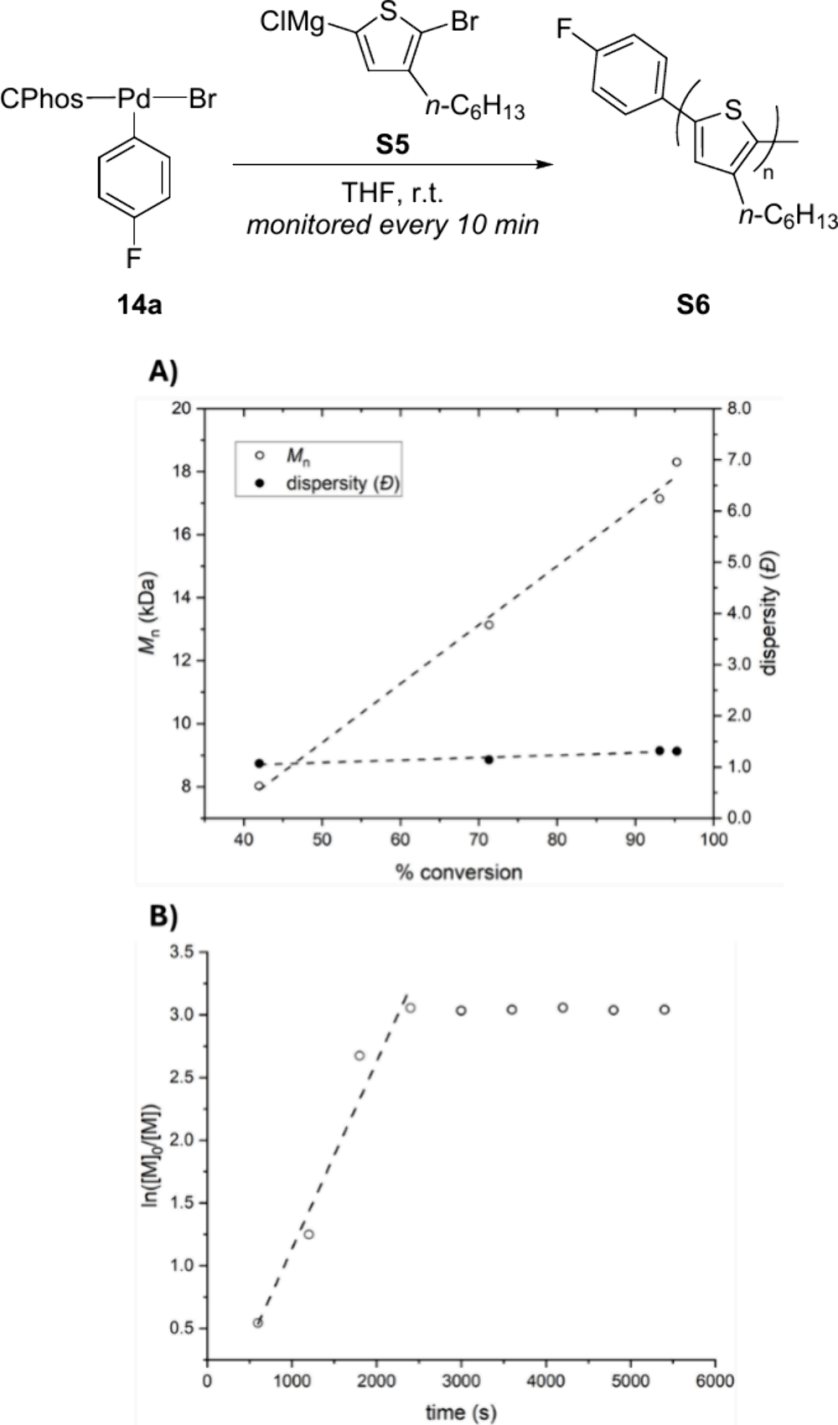
Plot of (A) *M*_n_ and dispersity versus conversion of monomer and (B) logarithm of [M]_0_/[M] versus time.

## 5 Summary

### 5.1 Effect of Phosphine Ligands and Halides (Cl, Br, and I) on the Rate of Transmetalation

Our systematic screening demonstrates that the structural properties of oxidative addition complexes markedly affect transmetalation rates (Figure 6A). Biaryl monophosphines with heteroatom substituents (e.g., CPhos, DavePhos, and RuPhos) promote rapid transmetalation, whereas electron-rich or sterically hindered phosphines (e.g., *t*-BuXPhos, dCypp, and *i*-Pr_3_P) considerably slow the process. Moreover, bisphosphine ligands with different bite angles (e.g., dppp (**12**), dppe (**7**), and dppp-dimethyl (**10**)) showed significantly different reactivities on transmetalation. Additionally, oxidative addition complexes (OACs) derived from chloride and bromide exhibit faster transmetalation than their iodide analogues, likely due to enhanced Pd–X bond polarization and reduced steric hindrance (Figure 6B). Eyring analyses further reveal that the transition state is primarily entropically governed, highlighting the role of ligand organization during transmetalation.

**Figure 6:**
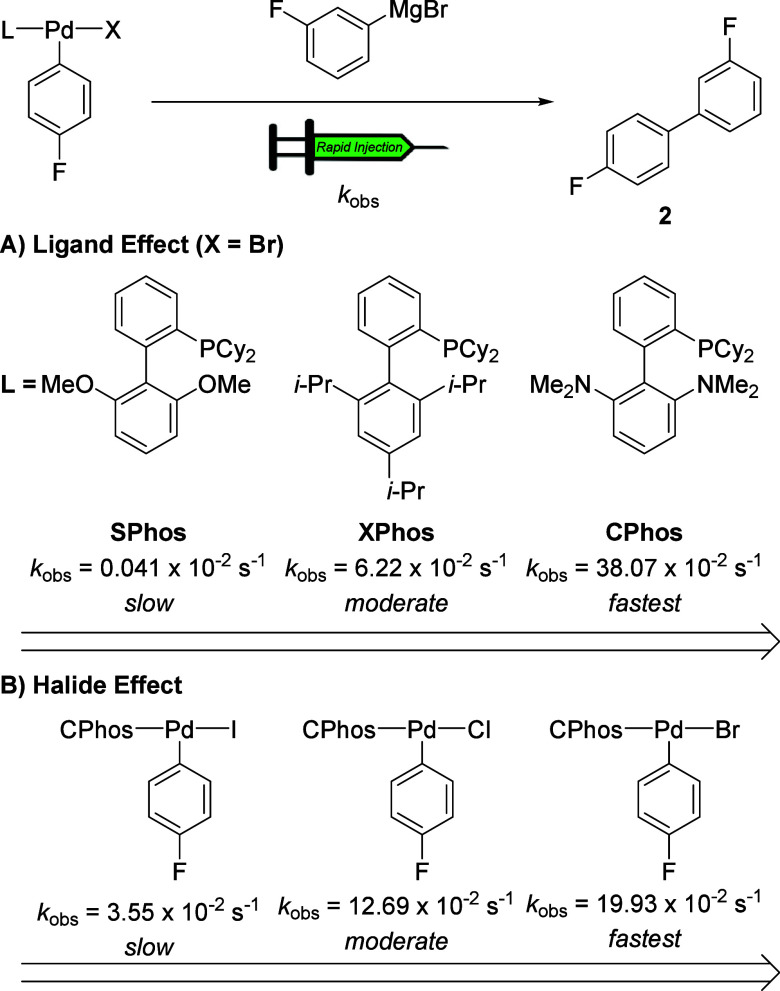
(A) Ligand effect on transmetalation. (B) Halide effect on transmetalation.

### 5.2 Catalyst Transfer Polymerization— P3HT Synthesis

Finally, harnessing these insights, we studied the catalyst-transfer polymerization (CTP) of 3-hexylthiophene. Oxidative addition complexes that demonstrate faster transmetalation rates yield higher molar mass polymers under otherwise identical conditions. (Figure 7). Slower ligands, despite sometimes favorable ring-walking behavior, fail to achieve comparable polymer chain growth. Chain-extension experiment suggested that CTP with CPhos (**14**) has living chain growth character, which is a desirable mechanism to gain controlled polymer profile. Interestingly, dppp that showed fast transmetalation rate provided poor polymer profile which is derived from inefficient of ring-walking or oxidative addition. These findings underscore the necessity of tuning transmetalation kinetics to optimize CTP outcomes and offer a blueprint for developing next-generation Pd catalysts in Kumada–Corriu cross-coupling.

**Figure 7:**
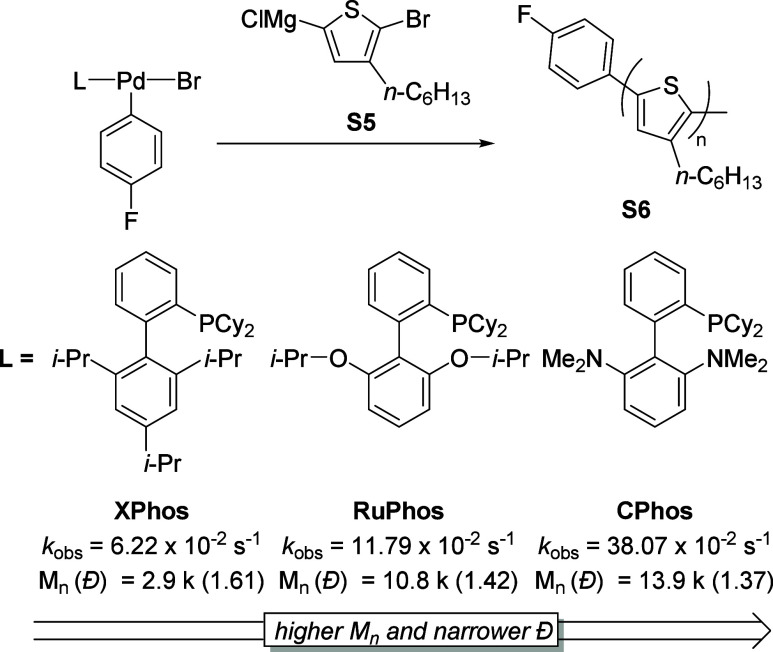
Impact of the transmetalation rate on KCTP profiles.

## 6 Conclusions

Despite its pivotal role in the Kumada–Corriu cross-coupling reaction, the mechanistic details of transmetalation remain insufficiently explored, with prior studies largely neglecting this critical step. In response to this gap, we employed RI-NMR techniques to thoroughly investigate the influence of the ligands and halides on the reaction rate. By synthesizing oxidative addition complexes via various routes, we bypassed the oxidative addition step in our kinetic studies, allowing us to isolate and focus on the transmetalation and reductive elimination sequence. Our findings, which demonstrate how the structure of oxidative addition complexes influences transmetalation rates, clearly establish transmetalation as the rate-limiting step, with reductive elimination occurring more rapidly. The effect of structural differences among phosphine ligands was further elucidated by DFT-D3 calculations by modeling the transmetalation transition states for the representative ligands (SPhos, RuPhos, and CPhos). The computational study suggested that the superior reactivity of the CPhos system is attributed to its higher electrophilicity of the Pd center and more favorable transition state geometry (square-pyrimidal). Notably, the tabulated kinetic data were directly applied to the development of Kumada catalyst-transfer polymerization (KCTP), underscoring the critical role of the transmetalation rate in the polymerization process. The KCTP with CPhos (**14a**) as a ligand, which showed the fastest rate on transmetalation, proved that transmetalation is crucial for obtaining polymers with high molar mass and narrow dispersity. The living chain growth character of this KCTP will also bring unlimited advantages to well-controlled polymerization. The significance of transmetalation is further amplified in Pd-catalyzed catalyst-transfer polymerization, which benefits from superior ring-walk efficiency—an advantage often lacking in Ni-catalyzed systems. This work establishes a robust framework for rapid ligand screening and catalyst optimization, paving the way for the development of next-generation Pd catalysts for precise polymer synthesis.

## 7 Methods

### 7.1 Preparation of Oxidative Addition Complexes

#### 7.1.1 General Procedure A (from Cámpora’s Palladacycle)

An oven-dried 1-dram vial was taken into glovebox and equipped with a magnetic stir bar. To the vial was added palladacycle **S2** (0.30 mmol, 1.0 equiv), phosphine ligand (0.315 mmol, 1.05 equiv), *n*-hexane (3.0 mL), and 1-bromo-4-fluorobenzene (33 μL, 0.3 mmol, 1.0 equiv) in order. The vial was then placed in a preheated aluminum block (60 °C). The reaction mixture was allowed to stir at 60 °C for 2 h. After 2 h, the vial was removed from the glovebox and cooled to room temperature. The vial was open to air and the reaction mixture was diluted with *n*-pentane (10 mL) to assist with precipitation. The precipitate was collected on filter paper and rinsed with *n*-pentane (10 mL x 3) and dried *in vacuo* on the Schlenk line overnight (∼16 h), yielding the desired complex.

#### 7.1.2 General Procedure B (from *trans*-[(4-F-C_6_H_4_)­Pd­(Ph_3_P)_2_Br] **5a**)

An oven-dried 1-dram vial was taken into the glovebox and equipped with a magnetic stir bar. To the vial was added *trans*-[(4-F-C_6_H_4_)­Pd­(Ph_3_P)_2_Br] **5a** (80.6 mg, 0.1 mmol, 1.0 equiv), phosphine ligand (0.1 mmol, 1.0 equiv), and toluene or THF (2.0 mL) in order. The reaction mixture was allowed to stir at 25 °C for 2 h. After 2 h, the vial was removed from the glovebox and cooled down to room temperature. The vial was opened to the air and concentrated to dryness under reduced pressure. The reaction mixture was diluted with pentane (10 mL) to assist with precipitation. The precipitate was collected on filter paper and rinsed with *n*-pentane (10 mL × 3) and dried *in vacuo* on a Schlenk line overnight (∼16 h) yielding the desired complex.

### 7.2 Rapid Injection Procedure for Kinetic Experiment

Inside the glovebox, a 5 mL volumetric flask was charged with 1,2-difluorobenzene (5.0 μL, 50.7 μmol) followed by dissolving with THF to the 5 mL mark generating a 0.01 M of internal standard stock solution. An oven-dried (150 °C), 5 mm, NMR tube was taken into the glovebox and charged with corresponding oxidative addition complexes (10 μmol) and 500 μL of the freshly prepared internal standard stock solution. The tube was capped with a septum. An oven-dried (60 °C) rapid injection barrel was taken into the glovebox and charged with (3-fluorophenyl)­magnesium bromide solution (500 μL, 1.33 M in THF). The glass capillary of the barrel was capped with a septum. The sample and barrel were removed from the glovebox and the sample was placed into NMR probe set to −10 °C with the cap off. The (3-fluorophenyl)­magnesium bromide **1** (120 μmol) in THF (90 μL) was injected by rapid injector apparatus. The progress of the reaction was monitored by a fluorine channel in comparison with the internal reference 1,2-difluorobenzene.

### 7.3 General Method of CTP: Synthesis of Poly­(3-hexylthiphene), P3HT

Inside the glovebox, a 25 mL oven-dried (150 °C) Schlenk flask equipped with a magnetic stir bar was charged with precatalyst (0.015 mmol, 1.0 equiv) and THF (6.86 mL) and then capped with a rubber septum. The flask was removed from the glovebox and connected to a Schlenk line under argon flow. Thiophene monomer **S5** (3.14 mL, 0.287 M in THF, 0.9 mmol, 60.0 equiv) was added via syringe and stirred for 90 min at 25 °C. The reaction was quenched with aq HCl (5 M, 10 mL), extracted with CHCl_3_ (3 × 20 mL), dried over Na_2_SO_4_, and filtered, and the solvent was removed under reduced pressure. The resulting purple solid was dissolved in a minimal amount of CHCl_3_ and precipitated into a 250 mL beaker containing MeOH (200 mL) under vigorous stirring (600 rpm). The precipitate was collected and dried under vacuum overnight, yielding P3HT **S6**. (SEC sample preparation: 10 mg of polymer sample was dissolved in THF (HPLC grade, 1.5 mL) and allowed to sit for 6 h to ensure polymer chain disentanglement. Just before filtration, the solution was gently heated using a heat gun, then filtered through a 0.2 μm PTFE filter into a sample vial for SEC analysis.)

### 7.4 Computational Methods

All geometry optimizations of intermediates and transition states were conducted using the spin restricted M06L^32^-D3 ^33^/6-31G­(d,p)^34^-LANL2DZ­(Pd,Br)^35^ method as implemented in Gaussian16.^36^ Moreover, the solvation effects were considered with THF as the solvent using the CPCM solvent model.^37^ Frequency calculations were also conducted at the same level of theory to obtain vibrational frequencies to determine the identity of stationary points as intermediates (no imaginary frequencies) or transition states (only one imaginary frequency) as well as obtain thermal correction to enthalpy and free energy at 298 K. Intrinsic reaction coordinate (IRC) calculations were done on the transition states to verify the correct transition state associated with the reaction. The end point geometries obtained from the IRC calculations were further optimized to verify the authenticity of the transition state. Also, an extensive conformational search was performed for all the intermediates and transition states, and only the lowest-energy species were shown and discussed. Finally, we performed single-point calculations on the optimized geometries by using the following levels of theory:

1. RM06L-D3/6-311+g­(d,p)-SDD­(Pd,Br)-CPCM­(THF)//RM06L-D3/6-31g­(d,p)-LANL2DZ­(Pd,Br)-CPCM­(THF)
2. RM06L-D3/def2tzvpp-CPCM­(THF)//RM06L-D3/6-31g­(d,p)-LANL2DZ­(Pd,Br)-CPCM­(THF)

All structural figures were generated with CYLview.^38^ Distances in structural figures are shown in Å, and energies are in kcal/mol. NBO analyis was done using Gaussian NBO Version 3.1.^39^ Conceptual density functional theory was used to calculate electrophilicty indices using Multiwfn.^40^

## Supplementary Material



## Data Availability

The data underlying this study are available in the published article and its online Supporting Information.

## Notes

Kinetic experiments, synthetic procedures, characterization data, kinetic data, other data, NMR spectra of these synthesized compounds, structures, NBO interactions, and Cartesian coordinates and energies (PDF)
