# Supplementary material for: Mechanistically Driven Development of Kumada Catalyst-Transfer Polymerizations: A Rapid Injection NMR Study
Source: ACS Catal. 2025 Dec 2;15(24):20847–59. doi: 10.1021/acscatal.5c07930 (PMC12723679; doi:10.1021/acscatal.5c07930)
Supplement: Supplementary file 1 [file cs5c07930_si_001.pdf]

Supporting Information for

Mechanistically Driven Development of Kumada Catalyst-Transfer Polymerizations: A Rapid Injection NMR Study

Seokmin Kang,† Wentao Cen,† Achyut Ranjan Gogoi,§ Jeanette Piña,† Adhya Suresh,§ Fernando Ramirez,† Osvaldo Gutierrez§*, and Andy A. Thomas†*

*Correspondence to: andythomas@tamu.edu†
*Correspondence to: o.gutierrez@ucla.edu§

This PDF file includes:
Materials and Methods
Figs. **S1** to **S120**
Tables **S1** to **S99**

Mechanistically Driven Development of Kumada Catalyst-Transfer Polymerizations: A Rapid Injection NMR Study

Seokmin Kang,[†] Wentao Cen,[†] Achyut Ranjan Gogoi,[§] Jeanette Piña,[†] Adhya Suresh,[§] Fernando Ramirez,[†] Osvaldo Gutierrez[§]*, and Andy A. Thomas[†]*

[†]Department of Chemistry, Texas A&M University, College Station, Texas, USA

[§]Department of Chemistry and Biochemistry, University of California, Los Angeles, Los Angeles, California, USA

*Correspondence to: andythomas@tamu.edu[†]

*Correspondence to: o.gutierrez@ucla.edu[§]

Supporting Information

Table of Contents

1. **Materials and Methods** S3
   - 1.1. **Materials** S3
   - 1.2. **NMR Spectroscopy** S4
   - 1.3. **Rapid Injection NMR (RI-NMR)** S4
   - 1.4 **Size Exclusion Chromatography** S4
2. **Experimental Procedures** S5
   - 2.1. **Preparation of Campora's Palladacycle** S5
   - 2.2. **Preparation of Ph_3P-OAC** S6
   - 2.3. **Preparation of Oxidative Addition Complexes and Grignard Reagent** S7
   - 2.4. **Index of Kinetic Experiments** S27
   - 2.5. **Catalyst Transfer Polymerization (CTP)** S136
3. **NMR Spectra** S143
4. **Computational Studies** S199
5. **References** S238

1. Materials and Methods

Reactions were conducted using glassware that had been oven-dried (150 °C) for a minimum of 1 hour unless otherwise noted. All reactions were conducted under an inert atmosphere using argon-filled glove box or standard Schlenk technique with argon connected to a drying tube equipped with phosphorous pentoxide, calcium sulfate, and sodium hydroxide, unless otherwise noted.

1.1. Materials

Tetrahydrofuran (Sigma-Aldrich, HPLC grade) and 2-methyl tetrahydrofuran (Sigma-Aldrich, contains 250 ppm BHT as stabilizer) were distilled over sodium/benzophenone. Pentane (Fisher Scientific, ACS grade) was distilled over sodium. *n*-Hexane (VWR, HPLC grade), eiethyl ether (VWR, Inhibitor-free, for spectroscopy), and toluene (Fischer Scientific, HPLC grade) were purified by passage through alumina. Methanol (Sigma-Aldrich, HPLC grade) was passed through 4 Å molecular sieves. Chloroform (VWR, ACS grade) was used without purification.

All deuterated solvents for NMR samples were purchased from Cambridge Isotope Labs. THF-d_8 was distilled over NaK (sodium/potassium alloy). Chloroform-*d* was distilled over CaH_2.

Compound suppliers:

2-Dicyclohexylphosphino-2′-(N,N-dimethylamino)biphenyl (DavePhos), 2-dicyclohexylphosphino-2′,6′-diisopropoxybiphenyl (RuPhos), diphenyl(2',4',6'-triisopropyl-[1,1'-biphenyl]-2-yl)phosphine (PhXPhos), 2-dicyclohexylphosphino-2′-methylbiphenyl (MePhos), 1,3-bis(diphenylphosphino)propane (dppp), 1,2-bis(diphenylphosphino)ethane (dppe), 2-dicyclohexylphosphino-2′,4′,6′-triisopropylbiphenyl (XPhos), 2-dicyclohexylphosphino-2′,6′-dimethoxybiphenyl (SPhos), 1-bromo-3-fluorobenzene, 4-fluoroiodobenzene, and 2-hydroxybenzaldehyde phenylhydrazone were purchased from Ambeed. 2-Dicyclohexylphosphino-2′,6′-bis(N,N-dimethylamino)biphenyl (CPhos), 2-di-tert-butylphosphino-2′,4′,6′-triisopropylbiphenyl (*t*BuXPhos), 2,2-dimethyl-1,3-dichloropropane, 1,2-difluorobenzene, tetrakis(triphenylphosphine)palladium ($(PPh_3)_4Pd$) were purchased from Combi-Blocks. Diphenylphosphine, 1,4-difluorobenzene, 1-bromo-4-fluorobenzene, 1-chloro-4-fluorobenzene, and zinc chloride solution, 1.9 M in 2-methyltetrahydrofuran were purchased from Oakwood. Triisopropylphosphine was purchased from STREM. *(**Caution**: Triisopropylphosphine spontaneously ignites under air atmosphere. It needs to be handled under inert atmosphere. Thus, Triisopropylphosphine was kept and used in the argon-filled glovebox in this study.)* 2-Methyl-2-phenylpropylmagnesium chloride and 1,5-cyclooctadiene were purchased from ThermoScientific. Palladium chloride ($PdCl_2$) and magnesium turnings (Mg) were purchased from STREM.

1.2. NMR Spectroscopy

^{1}H, ^{19}F, and ^{31}P NMR spectra for synthesized compounds were recorded on Bruker Avance Neo 400 MHz spectrometer (^{1}H, 400 MHz; ^{19}F, 376 MHz; ^{31}P, 162 MHz). ^{13}C NMR spectra for synthesized compounds were recorded on Bruker Avance III 500 MHz spectrometer, using cold probe (^{13}C, 126 MHz). ^{19}F NMR spectra for rapid injection kinetic experiments were recorded on Bruker Avance Neo 500 MHz NMR spectrometer (^{19}F, 471 MHz). Spectra for kinetics were referenced to 1,2-difluorobenzene as internal standard (–140.71 ppm). Spectra are referenced to residual chloroform (7.26 ppm, ^{1}H; 77.16 ppm, ^{13}C), residual benzene (7.16 ppm, ^{1}H; 128.37 ppm, ^{13}C), residual THF (1.72 ppm, ^{1}H; 67.21 ppm, ^{13}C), external fluorobenzene (−113.15 ppm, ^{19}F), and external triphenylphosphine (–6.50 ppm, ^{31}P). Chemical shifts are reported in ppm, multiplicities are indicated by s (singlet), d (doublet), t (triplet), q (quartet), p (pentet), h (hextet), hept (heptet), m (multiplet), and br (broad). Coupling constants, *J*, are reported in Hertz.

1.3. Rapid Injection NMR (RI-NMR)

The rapid injection apparatus consists of a gastight syringe connected to a long injection capillary with a perforated tip. Additions of Grignard solution were performed using air pressure, delivering an average of 90 µL of Grignard solution into the NMR tube containing the oxidative addition complex solution.

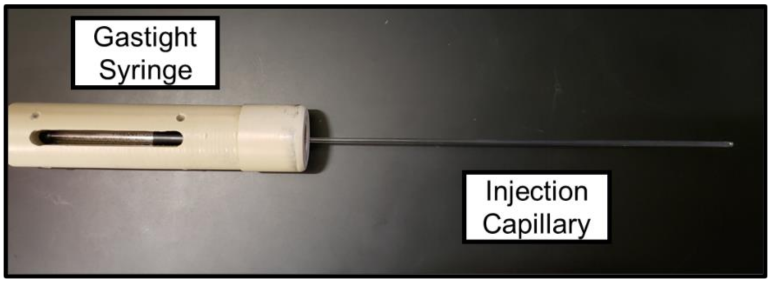

1.4 Size Exclusion Chromatography

The size exclusion chromatography (SEC) for polymer molar masses (M_{n} and M_{w}) and dispersity analysis was carried out with TOSOH Ambient Temperature GPC (refractive index detector) eluted with THF (HPLC grade). Polymer molar masses were determined by comparison with polystyrene standards. Samples were dissolved in THF (with mild heating) and passed through a 0.2 µm PTFE filter prior to analysis.

2. Experimental Procedures

2.1. Preparation of Campora's Palladacycle

(cod)PdCl$_2$ (S1)

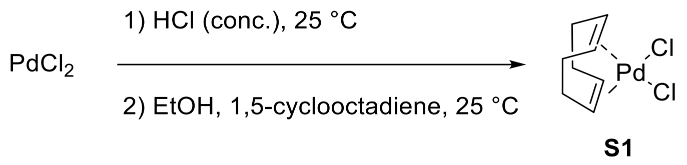

S1 was made according to a modified literature procedure.[i] To a 100 mL round-bottom flask with a magnetic stir bar was added $PdCl_2$ (3.55 g, 20.0 mmol, 1.0 equiv). The flask was charged with concentrated HCl (3.5 mL). The red-colored suspension was allowed to stir for 10 min at 25 °C. After 10 min, the suspension was filtered through a plug of Celite® into a 500 mL round-bottom flask. The plug of Celite® was further rinsed with EtOH (3 x 100 mL). To the flask was added a magnetic stir bar, followed by 1,5-cyclooctadiene (9.8 mL, 80 mmol, 4.0 equiv.). The reaction mixture was allowed to stir for 30 min under air. The precipitate was collected on the filter paper and rinsed with Et_2O (3 x 100 mL) yielding **S1** (5.4 g, 95%) as a bright yellow solid. Spectral data were in accordance with literature reports.[1]

^{1}H NMR (400 MHz, $CDCl_3$) δ 6.32 (s, 4H), 2.91 (m, 4H), 2.58 (m, 4H).

^{13}C NMR (101 MHz, $CDCl_3$) δ 116.79, 31.11.

(cod)Pd($CH_2CMe_2C_6H_4$) (S2), Cámpora's Palladacycle

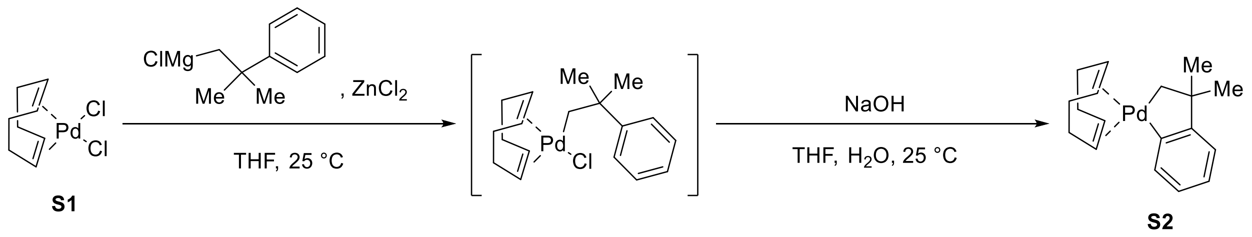

S2 was made according to a modified literature procedure.[1] A 500 mL Schlenk flask with a magnetic stir bar was connected to a dual manifold Schlenk line and placed under vacuum. The flask was flame-dried and charged with argon. The flask was charged with $ZnCl_2$ (1.9 M in 2-MeTHF, 10 mL, 1.93 mmol, 1.1 equiv) followed by THF (sodium/benzophenone, 200 mL). To the solution was added 2-methyl-2-phenylpropylmagnesium chloride (0.5 M in Et_2O, 35 mL, 17.5 mmol, 1.0 equiv) dropwise over 5 min to form a white precipitate. The suspension was allowed to stir for 30 min. In the presence of argon counterflow, **(cod)PdCl$_2$ (S1)** (5.0 g, 17.50 mmol, 1.0 equiv.) was added in one portion. The slurry was allowed to stir at 25 °C overnight (~16 h). The reaction mixture was filtered through a plug of Celite® into a 1.0 L round-bottom flask. The plug was further rinsed with THF (sodium/benzophenone, 350 mL). A magnetic stir bar was added to the flask containing the filtrate, followed by NaOH (freshly ground, 2.8 g)

solution in water (5.0 mL). The reaction mixture was stirred for 3 h at 25 °C. The solution was then concentrated to dryness under reduced pressure. The residue was suspended in Et_2O (from SDS, 350 mL) and filtered through a plug of Celite®. The plug was further rinsed with Et_2O (from SDS, 180 mL). The filtrate was concentrated to dryness under reduced pressure. The resulting residue was suspended in cold pentane (~ 0 °C, 42 mL), and the suspension was triturated with the assistance of sonication with ice-cold water. The resulting powder was collected on the filter paper and rinsed with cold pentane (~0 °C, 84 mL) yielding off-white powder. (4.3 g, 71%) Spectral data are in accordance with literature reports.[1]

^{1}H NMR (400 MHz, C_6D_6) δ 7.28 (dd, J = 7.5 Hz, 1H), 7.24 (td, J = 7.3 Hz, 1H), 7.18 (m, 1H), 7.14 (s, 1H), 5.68 (s, 2H), 5.17 (s, 2H), 2.55 (s, 2H), 1.85 (m, 8H), 1.58 (s, 6H).

^{13}C NMR (101 MHz, C_6D_6) δ 168.17, 163.18, 135.32, 125.57, 125.23, 123.94, 113.83, 110.49, 54.17, 50.41, 34.69, 29.61, 28.84.

2.2. Preparation of Ph_3P-OAC

***trans*-[(4-F-C_6H_4)Pd(Ph_3P)$_2$Br] (5a)**

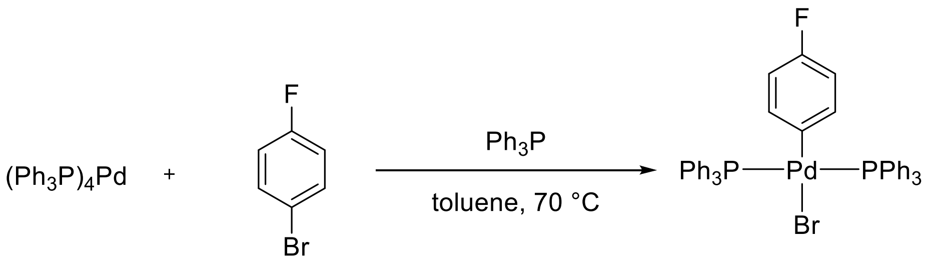

5a

An oven-dried (150 °C) 10-mL Schlenk flask was brought into glovebox The flask was charged with $(Ph_3P)_4Pd$ (500 mg, 0.43 mmol, 1.0 equiv.) and Ph_3P **5** (169 mg, 0.65 mmol, 1.5 equiv), followed by degassed toluene (5.0 mL). The flask was removed from the glove box, then connected to the Schlenk line with a reflux condenser equipped. Under argon atmosphere, the reaction mixture was allowed to stir overnight (~16 h) at 75 °C to form white precipitate. The precipitate was collected on the filter paper and rinsed with Et_2O (30 mL x 3) yielding (235 mg, 67%) as an off-white solid. Spectral data are in accordance with literature reports.[ii]

^{1}H NMR (400 MHz, $CDCl_3$): δ 7.57 – 7.47 (m, 12H), 7.34 (t, J = 7.3 Hz, 6H), 7.26 (t, J = 7.3 Hz, 12H), 6.50 (td, J = 6.5, 1.8 Hz, 2H), 6.03 (t, J = 9.2 Hz, 2H).

^{13}C NMR (126 MHz, $CDCl_3$): δ 160.38, 148.81, 136.27, 134.88, 131.52, 130.01, 128.05, 114.50.

^{19}F NMR (376 MHz, $CDCl_3$): δ –125.14.

^{31}P NMR (162 MHz, $CDCl_3$): δ 22.47.

2.3. Preparation of Oxidative Addition Complexes and Grignard Reagent

General Procedure A: From Cámpora's Palladacycle[1]

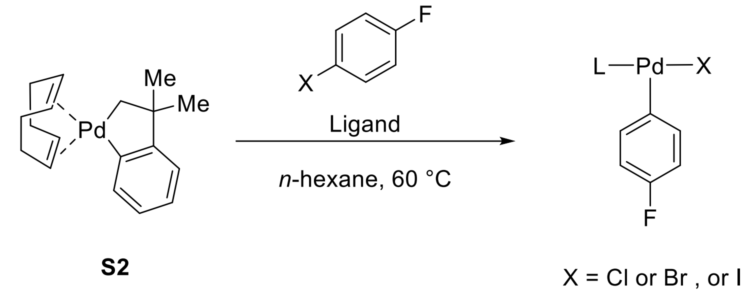

An oven-dried 1-dram vial was taken into glove box and equipped with a magnetic stir bar. To the vial was added palladacycle **S2** (0.30 mmol, 1.0 equiv.), ligand (0.315 mmol, 1.05 equiv), hexane (3.0 mL), and aryl halide (33 µL, 0.3 mmol, 1.0 equiv.) in order. The vial was then placed in a pre-heated aluminum block (60 °C). The reaction mixture was allowed to stir at 60 °C for 2 h. After 2 h, the vial was removed from the glove box and cooled down to room temperature. The vial was open to air and the reaction mixture was diluted with pentane (10 mL) to assist with precipitation. The precipitate was collected on a filter paper and rinsed with pentane (10 mL x 3) and dried *in vacuo* on a Schlenk line overnight (~16 h) yielding the desired complex.

General Procedure B: From *trans*-[(4-F-C_6H_4)Pd(Ph_3P)$_2$Br] **5a**

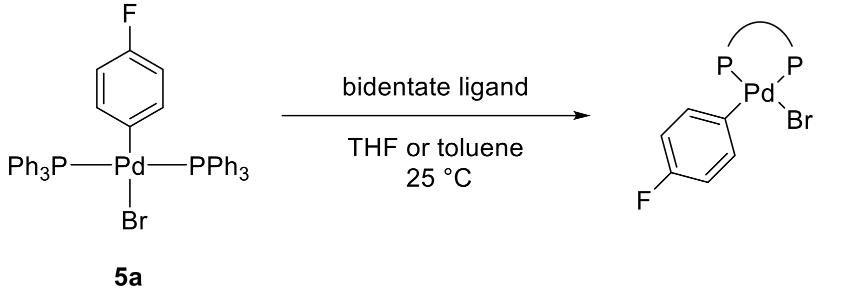

An oven-dried 1-dram vial was taken into glove box and equipped with a magnetic stir bar. To the vial was added *trans*-[(4-F-C_6H_4)Pd(Ph_3P)$_2$Br] **5a** (80.6 mg, 0.1 mmol, 1.0 equiv.), ligand (0.1 mmol, 1.0 equiv.), and toluene or THF (2.0 mL) in order. The reaction mixture was allowed to stir at 25 °C for 2 h. After 2 h, the vial was removed from the glove box and cooled down to room temperature. The vial was open to air and concentrated to dryness under reduced pressure. The reaction mixture was diluted with pentane (10 mL) to assist with precipitation. The precipitate was collected on filter paper and rinsed with pentane (10 mL x 3) and dried *in vacuo* on a Schlenk line overnight (~16 h) yielding the desired complex.

Preparation of Grignard Reagent, 3-Fluorophenylmagnesium bromide **(1)**

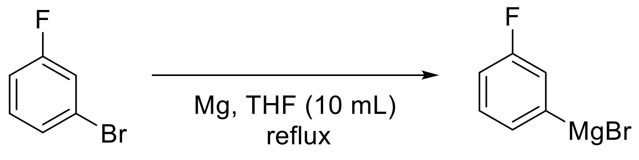

A three-necked round-bottom flask was equipped with magnetic stirrer, reflux condenser, rubber septa, and addition funnel. The reflux condenser was connected to a Schlenk line via rubber tubing with a gas adapter. The assembly was flame-dried under vacuum and subsequently backfilled with argon gas. To introduce magnesium turnings (438 mg, 18.0 mmol, 1.2 equiv), the dropping funnel was briefly removed and then reattached. The system was evacuated and backfilled with argon three times to ensure an inert atmosphere. Iced water was circulated through the reflux condenser. Under an argon atmosphere, THF (3.0 mL) was added via syringe, followed by addition of 3-fluorophenylbromide (0.30 mL, 2.68 mmol). A heat gun was used to gently warm the reaction mixture, initiating the magnesium insertion, which was indicated by a gentle reflux. Once initiation was observed, a solution of 3-fluorophenyl bromide (12.3 mmol in 7.0 mL THF) was added dropwise via the dropping funnel. After spontaneous reflux subsided, the flask was submerged in a pre-heated oil bath (75 °C) and allowed to stir for 2 h. The flask was removed from the oil bath and cooled down to room temperature yielding 3-fluorophenylmagnesium bromide **1.** The concentration of **1** (−118.77 ppm) was determined by ^{19}F NMR against 1,2-fluorobenzene (-140.09 ppm) as an internal standard. The Grignard reagent was stored in an argon filled glove box for rapid injection experiments.

(SPhos)Pd[(4-F)Ph]Br (3a)

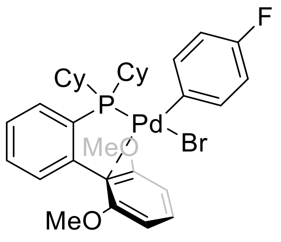

3a

3a was prepared according to the General Procedure **A** using palladacycle **S2** (104.0 mg, 0.30 mmol, 1.0 equiv), SPhos **3** (129 mg, 0.315 mmol, 1.05 equiv), 4-fluorobromobenzene (33 μL, 0.30 mmol, 1.0 equiv), and *n*-hexane (3.0 mL). The precipitate was collected on a filter paper yielding **3a** (182 mg, 88%) as a pale-yellow powder. Spectral data are in accordance with literature reports.[iii]

^{1}H NMR (400 MHz, THF-d_8) δ 7.33 (t, *J* = 7.6 Hz, 1H), 7.23 (t, *J* = 8.4 Hz, 1H), 7.16 – 7.02 (m, 2H), 6.79 (s, 3H), 6.70 – 6.56 (d, 2H), 6.38 (t, *J* = 8.8 Hz, 2H), 3.77 (br s, 6H), 2.95 (br s, 2H), 2.02 – 1.94 (m, 2H), 1.87 – 1.51 (m, 10H), 1.51 – 1.35 (m, 2H), 1.35 – 1.18 (m, 2H), 1.14 – 0.91 (m, 4H).

^{13}C NMR (126 MHz, THF-d_8) δ 162.17, 160.27, 158.63, 140.58, 140.51, 137.26, 134.95, 134.88, 133.56, 129.94, 129.39, 129.34, 129.09, 129.02, 126.30, 126.24, 119.26, 113.10, 112.95, 104.75, 56.13, 39.18, 33.20, 31.24, 31.18, 29.01, 28.88, 28.72, 28.66.

^{19}F NMR (376 MHz, THF-d_8) δ –124.75.

^{31}P NMR (162 MHz, THF-d_8) δ 38.71.

(*t*-BuXPhos)Pd[(4-F)Ph]Br (4a)

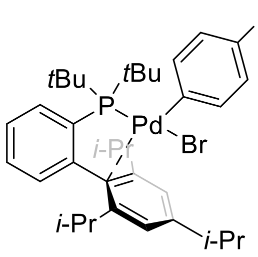

4a

4a was prepared according to the General Procedure **A** using palladacycle **S1** (104 mg, 0.30 mmol, 1.0 equiv), *t*BuXPhos **4** (134 mg, 0.315 mmol, 1.05 equiv), 4-fluorobromobenzene (33 µL, 0.3 mmol, 1.0 equiv), and *n*-hexane (3.0 mL). The precipitate was collected on a filter paper yielding **4a** (194 mg, 96%) as pale-yellow powder

^{1}H NMR (400 MHz, $CDCl_3$) δ 7.92 (m, 1H), 7.34 – 7.29 (m, 2H), 7.04 (s, 2H), 7.01 – 6.92 (m, 2H), 6.73 (m, 1H), 6.59 (t, *J* = 9.0 Hz, 2H), 3.02 (hept, *J* = 6.7 Hz, 1H), 2.48 (hept, *J* = 6.7 Hz, 2H), 1.52 (d, *J* = 6.8 Hz, 6H), 1.36 (–*t*Bu, s, 9H), 1.34 – 1.30 (–*t*Bu and –$CH(CH_3)_2$, 15H), 0.84 (d, *J* = 6.7 Hz, 6H).

^{13}C NMR (126 MHz, $CDCl_3$) Complex spectrum with two isomers, see below

^{19}F NMR (376 MHz, $CDCl_3$) δ –124.15. (major isomer)

^{31}P NMR (162 MHz, $CDCl_3$) δ 48.46. (major isomer)

Note: (*t*-BuXPhos)Pd[(4-F)Ph]Br exist as two monomeric species in $CDCl_3$. The ^{1}H NMR spectrum, along with ^{19}F and ^{31}P NMR analysis, confirms the presence of two isomers in an approximate 1.0:2.8 ratio. Only major peaks were picked and integrated in the ^{1}H spectrum. This observation was in accordance with literature report investigating analogue without fluorine atom.[iv]

(MePhos)Pd[(4-F)Ph]Br (6a)

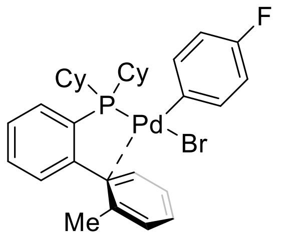

6a

6a was prepared according to the General Procedure **A** using palladacycle **S1** (104 mg, 0.30 mmol, 1.0 equiv), MePhos **6** (115 mg, 0.315 mmol, 1.05 equiv), 4-fluorobromobenzene (33 µL, 0.3 mmol, 1.0 equiv), and *n*-hexane (3.0 mL). The precipitate was collected on a filter paper yielding **6a** (173 mg, 89%) as a pale-yellow powder

^{1}H NMR (400 MHz, THF-d_8) Complex spectrum with peak broadening, see below

^{13}C NMR (126 MHz, THF-d_8) δ 161.21, 145.37, 142.57, 141.78, 137.04, 133.46, 131.50, 129.59, 128.68, 126.97, 113.49, 113.33, 38.05, 31.31, 30.81, 28.62, 28.28, 27.04, 26.91, 21.30.

^{19}F NMR (376 MHz, THF-d_8) δ –124.49.

^{31}P NMR (162 MHz, THF-d_8) δ 36.13.

Note: Significant peak broadening was observed in ^{1}H NMR.

(dppe)Pd[(4-F)Ph]Br (7a)

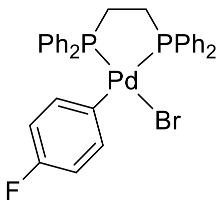

7a

7a was prepared according to the modified General Procedure **B** using *trans*-[(4-F-C_6H_4)Pd(Ph_3P)$_2$Br] **5a** (80.6 mg, 0.1 mmol, 1.0 equiv), dppe **7** (43.8 mg, 0.1 mmol, 1.0 equiv), and THF (4.0 mL) with 6 hours of reaction time. The precipitate was collected on a filter paper yielding **7a** (19 mg, 28%) as a off-white powder. Spectral data are in accordance with literature reports.[v]

^{1}H NMR (400 MHz, $CDCl_3$): δ 7.96–7.91 (br, 4H), 7.46–7.32 (m, 16H), 6.99–6.94 (m, 2H), 6.54–6.50 (m, 2H), 2.52–2.39 (m, 2H), 2.26–2.14 (m, 2H).

^{13}C NMR (126 MHz, $CDCl_3$): δ 161.7 & 159.8 (d, *J* = 239.4 Hz, C–F, 1C), 151.5 & 150.5 (d, *J* = 134.5 Hz, C–Pd, 1C), 137.0–136.9 (m, C–H, 2C), 133.6 & 133.5 (d, *J* = 11.8 Hz, 2C), 133.4 & 133.3 (d, *J* = 11.1 Hz, C–H, 2C), 131.5 & 131.1 (dd, *J* = 2.7, 56.2 Hz, 2C), 131.3 & 131.0 (d, *J* = 38.5 Hz, C–Pd, 2C), 129.5 & 129.1 (d, *J* = 50.9 Hz, C–Pd, 2C), 129.1 (d, *J* = 9.8 Hz, C–H, 2C), 129.0 & 128.9 (d, *J* = 11.0 Hz, C–H, 2C), 114.2–114.0 (m, C–H, 2C), 30.4–29.9 (m, CH_2, 1C), 24.0–23.7 (m, CH_2, 1C).

^{19}F NMR (376 MHz, $CDCl_3$): δ –123.30.

^{31}P NMR (162 MHz, $CDCl_3$): δ 52.25 & 52.08 (d, *J* = 26.7 Hz), 33.49 & 33.33 (d, *J* = 26.7 Hz).

(XPhos)Pd[(4-F)Ph]Br (8a)

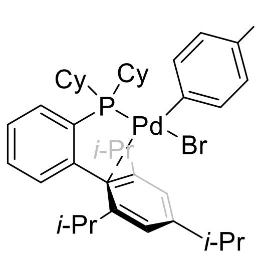

8a

8a was prepared according to the modified General Procedure **A** using palladacycle **S1** (104 mg, 0.30 mmol, 1.0 equiv), XPhos **8** (172 mg, 0.36 mmol, 1.2 equiv), 4-fluorobromobenzene (83 µL, 0.75 mmol, 2.5 equiv), 2-MeTHF (1.25 mL), and THF (2.5 mL). In a glove box, **S1** and the XPhos were dissolved in 2-MeTHF in a 1-dram vial equipped with magnetic stir bar. The solution was allowed to stir for at 60 °C for 30 min, then cooled to room temperature, followed by the addition of 4-fluorobromobenzene. The reaction mixture was then removed from the glove box and concentrated to dryness under reduced pressure. The solid was triturated by adding pentane (20 mL). The precipitate was collected on a filter paper yielding **8a** (183 mg, 80%) as a pale-yellow powder.

^{1}H NMR (400 MHz, $CDCl_3$) δ 7.66 (m, 1H), 7.46 – 7.37 (m, 2H), 7.13 (s, 2H), 6.96 (m, 2H), 6.87 (m, 1H), 6.73 (t, J = 8.8 Hz, 2H), 3.12 (hept, J = 6.9 Hz, 1H), 2.43 (hep, J = 6.7 Hz, 2H), 2.20 (m 2H), 1.96 (s, 2H), 1.85 – 1.56 (m, 16H) 1.39 (d, J = 6.9 Hz, 6H), 1.18 (m,, 6H), 0.90 (d, J = 6.6 Hz, 6H), 0.73 – 0.60 (m, 2H).

^{13}C NMR (126 MHz, $CDCl_3$) δ 162.06, 156.49, 149.62, 148.91, 147.75, 142.25, 136.91, 134.66, 133.66, 131.94, 130.62, 127.01, 126.29, 125.41, 124.96, 114.30, 35.53, 35.32, 34.37, 31.68, 30.36, 28.51, 27.88, 27.78, 27.44, 26.01, 25.65, 24.83, 24.61, 24.24.

^{19}F NMR (376 MHz, $CDCl_3$) δ –122.83.

^{31}P NMR (162 MHz, $CDCl_3$) δ 25.44.

(XPhos)Pd[(4-F)Ph]Cl (8b)

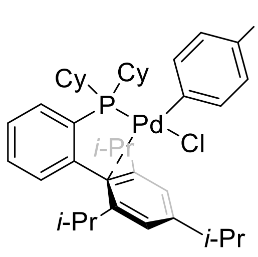

8b

8b was prepared according to the General Procedure **A** using palladacycle **S1** (104 mg, 0.30 mmol, 1.0 equiv), XPhos **8** (150 mg, 0.315 mmol, 1.05 equiv), 4-fluorochlorobenzene (32 μL, 0.30 mmol, 1.0 equiv), and *n*-hexane (3.0 mL). The precipitate was collected on a filter paper yielding **8b** (84 mg, 39%) as a pale-yellow powder.

^{1}H NMR (400 MHz, $CDCl_3$) δ 7.66 (m, 1H), 7.42 (m, 2H), 7.12 (s, 2H), 6.98 (m, 2H), 6.90 (m, 1H), 6.73 (t, J = 9.0 Hz, 2H), 3.04 (hep, 1H), 2.43 (hep, 2H), 2.25 – 2.12 (m, 2H), 1.94 (s, 2H), 1.84 – 1.55 (m, 16H), 1.38 (d, 6H), 1.32 – 1.07 (m, 6H), 0.90 (d, 6H), 0.70 (m, 2H).

^{13}C NMR (126 MHz, $CDCl_3$) δ 162.09, 156.56, 149.57, 148.93, 147.98, 142.26, 136.50, 134.91, 133.65, 131.89, 130.58, 129.31, 127.05, 125.16, 124.71, 114.29, 35.49, 35.27, 34.61, 31.73, 30.36, 28.60, 27.95, 27.93, 27.61, 27.35, 26.04, 25.70, 24.80, 24.54, 24.24.

^{19}F NMR (376 MHz, $CDCl_3$) δ –122.66.

^{31}P NMR (162 MHz, $CDCl_3$) δ 27.43.

(PhXPhos)Pd[(4-F)Ph]Br (9a)

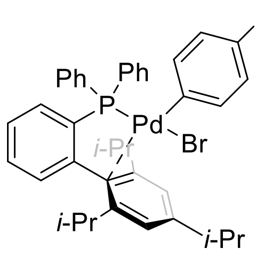

9a

9a was prepared according to the General Procedure **A** using palladacycle **S1** (104 mg, 0.3 mmol, 1.0 equiv), PhXPhos **9** (146 mg, 0.315 mmol, 1.05 equiv), 4-fluorobromobenzene (33 μL, 0.3 mmol, 1.0 equiv), and *n*-hexane (3.0 mL). The precipitate was collected on a filter paper yielding **9a** (199 mg, 89%) as a white powder.

^{1}H NMR (500 MHz, THF-d_8) δ 9.29 (dd, *J* = 16.9, 7.8 Hz, 1H), 7.65 (t, *J* = 7.7 Hz, 1H), 7.49 (t, *J* = 7.6 Hz, 1H), 7.39 – 7.29 (m, 2H), 7.19 (m, 3H), 6.99 (s, 6H), 6.73 (s, 2H), 6.52 (m, 2H), 6.19 (s, 2H), 2.74 (hept, 1H), 2.25 (br s, 2H), 1.20 (d, *J* = 6.9 Hz, 6H), 0.63 (br s, 6H), 0.30 (br s, 6H).

^{13}C NMR (126 MHz, THF-d_8) δ 160.82, 159.92, 149.13, 148.40, 147.19, 146.18, 144.28, 142.75, 137.50, 136.28, 135.76, 134.59, 134.38, 130.54, 130.18, 129.12, 128.01, 126.50, 120.89, 114.09, 113.55, 34.94, 30.81, 25.78, 24.27, 21.83.

^{19}F NMR (376 MHz, THF-d_8) δ –125.13.

^{31}P NMR (202 MHz, THF-d_8) δ 41.47.

(dppp-dimethyl)Pd[(4-F)Ph]Br (10a)

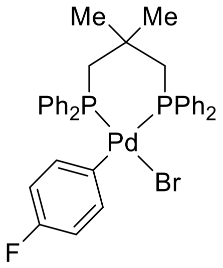

10a

10a was prepared according to the General Procedure **B** using *trans*-[(4-F-C_6H_4)Pd(Ph_3P)$_2$Br] **5a** (80.6 mg, 0.1 mmol, 1.0 equiv), dppp-dimethyl **10** (44.05 mg, 0.1 mmol, 1.0 equiv), and toluene (2.0 mL) with 1 h of reaction time. The precipitate was collected on a filter paper yielding **10a** (62 mg, 86%) as a off-white powder.

^{1}H NMR (400 MHz, $CDCl_3$): δ 7.76 – 7.71 (m, 4H), 7.37 – 7.04 (m, 16H), 6.66 (q, *J* = 6.7 Hz, 2H), 6.17 (t, *J* = 9.0 Hz, 2H), 2.24 (d, *J* = 9.8 Hz, 2H). 2.13 (d, *J* = 9.8 Hz, 2H), 0.57 (s, 6H).

^{13}C NMR (126 MHz, $CDCl_3$): δ 161.19, 159.29, 152.62, 151.57, 136.40, 134.34 (d, *J* = 11.8 Hz), 133.56 (d, *J* = 10.9 Hz), 133.12, 132.82, 132.32, 131.89, 130.68, 130.38, 128.59 (dd, J = 10.2, 6.1 Hz), 113.86 (dd, *J* = 18.5, 10.1 Hz), 41.47 (d, *J* = 24.4 Hz), 38.84 (d, *J* = 12.1 Hz), 35.31 (d, *J* = 4.6 Hz), 33.16 (t, *J* = 7.4 Hz).

^{19}F NMR (376 MHz, $CDCl_3$): δ –124.25.

^{31}P NMR (162 MHz, $CDCl_3$): δ 17.16 (d, *J* = 47.2 Hz), -4.29 (d, *J* = 47.2 Hz).

(RuPhos)Pd[(4-F)Ph]Br (11a)

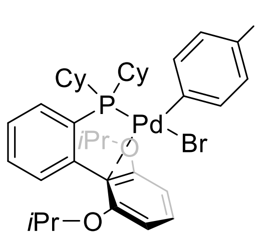

11a

11a was prepared according to the General Procedure **A** using palladacycle **S1** (104 mg, 0.3 mmol, 1.0 equiv), RuPhos **11** (147 mg, 0.315 mmol, 1.05 equiv), 4-fluorobromobenzene (33 µL, 0.3 mmol, 1.0 equiv), and *n*-hexane (3.0 mL). The precipitate was collected on a filter paper yielding **11a** (187 mg, 83%) as a pale-yellow powder.

^{1}H NMR (400 MHz, $CDCl_3$) δ 7.65 (t, *J* = 8.4 Hz, 1H), 7.58 (t, *J* = 7.3, 1H), 7.39 (m, 2H), 7.02 (m, 2H), 6.86 (m, 1H), 6.78 – 6.68 (m, 2H), 6.65 (d, *J* = 8.5 Hz, 2H), 4.60 (hept, *J* = 6.0 Hz, 2H), 2.11 (m, 2H), 1.85 – 1.46 (m, 12H), 1.37 (d, *J* = 6.0 Hz, 6H), 1.29 – 1.05 (m, 6H), 1.01 (d, *J* = 6.0 Hz, 6H), 0.92 – 0.68 (m, 2H).

^{13}C NMR (126 MHz, $CDCl_3$) δ 161.035, 159.06, 144.95, 144.81, 137.51, 134.95, 133.46, 133.18, 132.73, 132.64, 130.90, 130.69, 127.56, 126.54, 126.49, 114.07, 113.91, 111.48, 107.63, 71.06, 34.05, 33.84, 28.30, 27.77, 27.29, 26.95, 26.14, 22.32, 21.71.

^{19}F NMR (376 MHz, $CDCl_3$) δ –122.90.

^{31}P NMR (162 MHz, $CDCl_3$) δ 25.44.

(RuPhos)Pd[(4-F)Ph]Cl (11b)

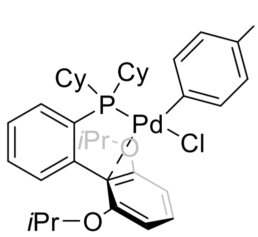

11b

11b was prepared according to the General Procedure **A** using palladacycle **S1** (104 mg, 0.3 mmol, 1.0 equiv), RuPhos **11** (147 mg, 0.315 mmol, 1.05 equiv), 4-fluorochlorobenzene (32 µL, 0.3 mmol, 1.0 equiv), and *n*-hexane (3.0 mL). The precipitate was collected on a filter paper yielding **11b** (175 mg, 83%) as a off-white powder.

^{1}H NMR (400 MHz, $CDCl_3$) δ 7.65 (t, *J* = 8.4 Hz, 1H), 7.58 (t, *J* = 6.5 Hz, 1H), 7.47 – 7.32 (m, 2H), 7.08 – 6.98 (m, 2H), 6.89 (d, *J* = 7.6 Hz, 1H), 6.73 (t, *J* = 9.0 Hz, 2H), 6.66 (d, *J* = 8.5 Hz, 2H), 4.62 (hept, *J* = 6.0 Hz, 2H), 2.17 – 2.01 (m, 2H), 1.83 – 1.47 (m, 12H), 1.36 (d, *J* = 6.1 Hz, 6H), 1.28 – 1.04 (m, 6H), 1.01 (d, *J* = 6.1 Hz, 6H), 0.77 (m, *J* = 12.6 Hz, 2H).

^{13}C NMR (126 MHz, $CDCl_3$) δ 162.03, 160.12, 158.68, 144.90, 136.94, 134.82, 133.18, 132.89, 132.74, 132.65, 130.84, 130.60, 130.18, 126.54, 114.21, 111.94, 107.59, 71.06, 33.93, 33.71, 28.34, 27.78, 27.36, 27.03, 26.94, 26.18, 22.33, 21.64.

^{19}F NMR (376 MHz, $CDCl_3$) δ –123.10.

^{31}P NMR (162 MHz, $CDCl_3$) δ 30.84.

(RuPhos)Pd[(4-F)Ph]I (11c)

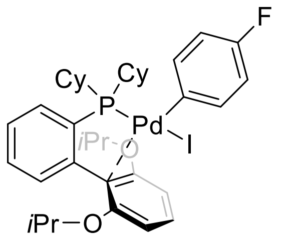

11c

11c was prepared according to the General Procedure **A** using palladacycle **S1** (104 mg, 0.3 mmol, 1.0 equiv), RuPhos **11** (147 mg, 0.315 mmol, 1.05 equiv), 4-fluorochlorobenzene (35 µL, 0.3 mmol, 1.0 equiv), and *n*-hexane (3.0 mL). The precipitate was collected on a filter paper yielding **11c** (194 mg, 81%) as a yellow powder.

^{1}H NMR (400 MHz, $CDCl_3$) δ 7.68 – 7.55 (m, 2H), 7.38 (m, 2H), 6.99 (m, 2H), 6.83 (ddd, J = 7.6, 3.1, 1.5 Hz, 1H), 6.77 – 6.68 (m, 2H), 6.65 (d, J = 8.4 Hz, 2H), 4.64 – 4.51 (hept, J = 6.4 Hz, 2H), 2.12 (m, 2H), 1.86 – 1.49 (m, 12H), 1.38 (d, J = 6.0 Hz, 6H), 1.27 – 1.05 (m, 6H), 1.01 (d, J = 6.0 Hz, 6H), 0.76 (m, 2H).

^{13}C NMR (126 MHz, $CDCl_3$) δ 160.97, 159.41, 144.81, 138.67, 135.00, 133.53, 133.27, 132.77, 132.68, 130.99, 130.70, 126.45, 122.26, 113.64, 111.33, 107.94, 71.14, 34.08, 33.88, 28.23, 27.70, 27.45, 27.35, 27.07, 26.98, 26.13, 22.33, 21.81.

^{19}F NMR (376 MHz, $CDCl_3$) δ –123.48.

^{31}P NMR (162 MHz, $CDCl_3$) δ 25.43.

(dppp)Pd[(4-F)Ph]Br (12a)

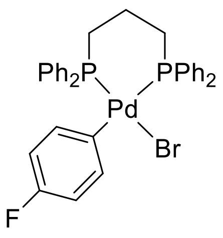

12a

12a was prepared according to the modified General Procedure **B** using *trans*-[(4-F-C_6H_4)Pd(Ph_3P)$_2$Br] **5a** (80.6 mg, 0.1 mmol, 1.0 equiv), dppp **12** (41.2 mg, 0.1 mmol, 1.0 equiv), and toluene (1.7 mL) with 1 h of reaction time. The precipitate was collected on a filter paper yielding **12a** (60.5 mg, 87%) as a off-white powder. Spectral data are in accordance with literature reports.[5]

^{1}H NMR (500 MHz, THF-d_8): δ 7.92–7.88 (m, 4H), 7.40–7.36 (m, 10H) 7.31–7.28 (m, 2H), 7.18–7.15 (m, 4H) 6.78–6.74 (m, 2H), 6.24–6.21 (m, 2H), 2.67–2.62 (m, 2H), 2.46–2.42 (m, 2H), 1.90–1.71 (m, 2H).

^{13}C NMR (126 MHz, THF-d_8): δ 162.0 & 160.1 (d, J = 237.9 Hz, C–F, 1C), 154.6 & 153.6 (d, J = 135.8 Hz, C–Pd, 1C), 137.5 (m, C–H, 2C), 135.0 & 134.9 (d, J =11.0 Hz, C–H), 134.5 & 134.4 (d, J = 10.8 Hz, C–H), 134.3 & 134.0 (d, J = 36.5 Hz, C–P, 2C), 133.0 & 132.6 (d, J = 52.3 Hz, C–P, 2C), 131.1 & 130.8 (dd, J = 2.4, 47.0 Hz, C–H), 129.1 & 129.0 (dd, J = 3.3, 9.3 Hz, C–H), 113.8 & 113.6 (m, C–H, 2C), 29.2 & 29.0 (dd, J = 4.5, 27.5 Hz, CH_2), 27.6 & 27.5 (dd, J = 3.8, 20.4 Hz, CH_2), 20.02 (d, J = 4.2 Hz, CH_2).

^{19}F NMR (376 MHz, THF-d_8): δ –125.85.

^{31}P NMR (162 MHz, THF-d_8): δ 15.29 (d, J = 50.9 Hz), –9.13 (d, J = 50.9 Hz).

(DavePhos)Pd[(4-F)Ph]Br (13a)

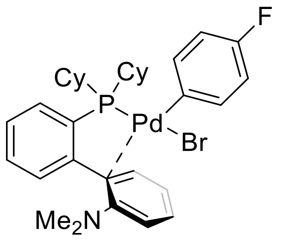

13a

13a was prepared according to the General Procedure **A** using palladacycle **S1** (104 mg, 0.30 mmol, 1.0 equiv) , DavePhos **13** (124 mg, 0.315 mmol, 1.05 equiv), 4-fluorobromobenzene (33 μL, 0.3 mmol, 1.0 equiv), and *n*-hexane (3.0 mL). The precipitate was collected on a filter paper yielding **(13a)** (154 mg, 76%) as a pale-yellow powder

^{1}H NMR (400 MHz, $CDCl_3$) δ 7.63 (t, J = 7.3 Hz, 1H), 7.55 (m, 1H), 7.43 – 7.28 (m, 2H), 7.22 – 6.90 (m, 6H), 6.75 (t, J = 8.7 Hz, 2H), 2.97 (s, 6H), 2.46 (s, 1H), 2.27 (m, 1H), 2.12 (m, 1H), 2.01 – 1.74 (m, 4H) 1.74 – 1.48 (m, 6H), 1.46 – 1.17 (m, 6H), 1.01 (m, 3H), 0.10 (d, J = 12.7 Hz, 1H).

^{13}C NMR (126 MHz, $CDCl_3$) δ 162.04, 160.12, 155.40, 151.34, 138.79, 135.83, 134.34, 132.82, 131.76, 131.26, 130.04, 129.14, 126.86, 125.28, 120.75, 118.51, 117.89, 114.37, 44.18, 36.44, 35.30, 31.11, 27.84, 27.43, 27.30, 27.18, 26.51, 26.19.

^{19}F NMR (376 MHz, $CDCl_3$) δ –122.25.

^{31}P NMR (162 MHz, $CDCl_3$) δ 27.56.

(CPhos)Pd[(4-F)Ph]Br (14a)

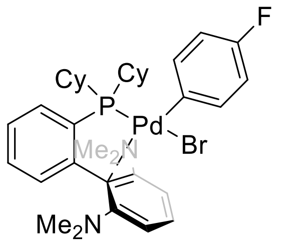

14a

14a was prepared according to the General Procedure **A** using palladacycle **S1** (104 mg, 0.30 mmol, 1.0 equiv), CPhos **14** (138 mg, 0.315 mmol, 1.05 equiv), 4-fluorobromobenzene (33 µL, 0.30 mmol, 1.0 equiv), and *n*-hexane (3.0 mL). The precipitate was collected on a filter paper yielding **14a** (183 mg, 85%) as a pale-yellow powder.

^{1}H NMR (400 MHz, $CDCl_3$) δ 7.68 (t, J = 7.3 Hz, 1H), 7.61 (t, J = 8.1 Hz, 1H), 7.43 (m, 1H), 7.33 (m, 1H), 7.20 – 7.01 (m, 3H), 6.90 (d, J = 8.1 Hz, 2H), 6.73 (t, J = 8.9 Hz, 2H), 2.59 (s, 12H), 28 (m, 2H), 2.01 (br s, 2H), 1.70 (m, 8H), 1.56 – 1.41 (m, 2H), 1.33 – 1.05 (m, 6H), 1.01 – 0.89 (m, 2H).

^{13}C NMR (126 MHz, $CDCl_3$) δ 161.99, 160.07, 156.17, 146.84, 136.78, 134.79, 134.40, 134.12, 134.03, 133.47, 130.79, 127.85, 126.10, 116.78, 115.86, 114.15, 45.15, 36.96, 36.77, 29.45, 27.95, 27.85, 27.69, 27.60, 26.02, 14.23.

^{19}F NMR (376 MHz, $CDCl_3$) δ –122.91.

^{31}P NMR (162 MHz, $CDCl_3$) δ 29.69.

(CPhos)Pd[(4-F)Ph]Cl (14b)

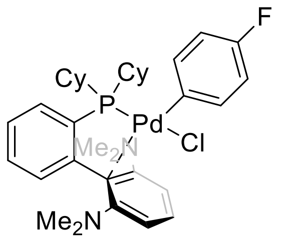

14b

14b was prepared according to the General Procedure **A** using palladacycle **S1** (104 mg, 0.30 mmol, 1.0 equiv), CPhos **14** (138 mg, 0.315 mmol, 1.05 equiv), 4-fluorochlorobenzene (32 μL, 0.3 mmol, 1.0 equiv), and *n*-hexane (3.0 mL). The precipitate was collected on a filter paper yielding **14b** (117 mg, 58%) as a pale-yellow powder.

^{1}H NMR (400 MHz, $CDCl_3$) δ 7.65 (m, 2H), 7.44 (t, J = 7.6 Hz, 1H), 7.38 – 7.30 (m, 1H), 7.15 – 7.01 (m, 3H), 6.92 (d, J = 8.0 Hz, 2H), 6.74 (t, J = 8.9 Hz, 2H), 2.59 (s, 12H), 2.31 – 2.20 (m, 2H), 1.99 (m, 2H), 1.69 (m, 8H), 1.54 – 1.39 (m, 2H), 1.34 – 1.03 (m, 6H), 0.97 – 0.81 (m, 2H).

^{13}C NMR (126 MHz, $CDCl_3$) δ 161.08, 160.12, 155.84, 147.07, 136.38, 134.74, 134.17, 133.29, 130.68, 130.22, 126.20, 117.63, 116.03, 114.16, 45.21, 36.62, 29.32, 27.92, 27.70, 26.04.

^{19}F NMR (376 MHz, $CDCl_3$) δ –122.75.

^{31}P NMR (162 MHz, $CDCl_3$) δ 30.39.

(CPhos)Pd[(4-F)Ph]I (14c)

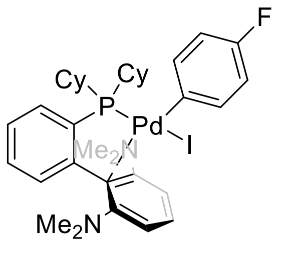

14c

14c was prepared according to the General Procedure **A** using palladacycle **S1** (104 mg, 0.30 mmol, 1.0 equiv), CPhos **14** (138 mg, 0.315 mmol, 1.05 equiv), 4-fluoroiodobenzene (35 µL, 0.3 mmol, 1.0 equiv), and *n*-hexane (3.0 mL). The precipitate was collected on a filter paper yielding **14c** (212 mg, 92%) as a yellow powder

^{1}H NMR (400 MHz, $CDCl_3$) δ 7.68 (m, 1H), 7.55 (t, *J* = 8.1 Hz, 1H), 7.42 (m, 1H), 7.31 (m, 1H), 7.09 – 6.97 (m, 3H), 6.88 (d, *J* = 8.1 Hz, 2H), 6.79 – 6.67 (m, 2H), 2.59 (s, 12H), 2.30 (m, 2H), 2.02 (br s, 2H), 1.79 – 1.64 (m, 8H), 1.46 (m, 2H), 1.28 – 1.03 (m, 6H), 0.93 (m, 2H).

^{13}C NMR (126 MHz, $CDCl_3$) δ 161.91, 160.00, 156.59, 146.82, 137.85, 134.95, 134.71, 134.44, 134.13, 134.04, 133.70, 130.88, 125.95, 122.99, 115.85, 113.70, 113.52, 45.11, 37.06, 36.88, 29.46, 28.01, 27.74, 26.02.

^{19}F NMR (376 MHz, $CDCl_3$) δ –123.56.

^{31}P NMR (162 MHz, $CDCl_3$) δ 25.82.

***trans*-[(4-F-C_6H_4)Pd(*i*-Pr_3P)$_2$Br] (15a)**

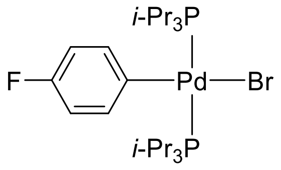

15a

15a was prepared according to the modified General Procedure **A** using palladacycle **S1** (104 mg, 0.3 mmol, 1.0 equiv), *i*Pr_3P **15** (144 mg, 0.9 mmol, 3.0 equiv), 4-fluorobromobenzene (80 µL, 0.75 mmol, 2.5 equiv), 2-MeTHF (1.25 mL), and THF (2.5 mL). In a glovebox, **S1** and the *i*Pr_3P were dissolved in 2-MeTHF in a 1-dram vial equipped with magnetic stir bar. The solution was allowed to stir for at 60 °C for 30 min, then cooled to room temperature, followed by the addition of 4-fluorobromobenzene. The reaction mixture was concentrated to dryness under reduced pressure in the glovebox. The solid was precipitated by adding pentane (1.0 mL) and cooling the mixture to –30 °C in a freezer inside the glove box. The precipitate was collected on a fritted funnel yielding **15a** (91 mg, 50%) as a pale-yellow powder.

^{1}H NMR (400 MHz, THF-*d*$_8$) δ 7.42 – 7.33 (m, 2H), 6.78 – 6.67 (m, 2H), 2.40 (m, 6H), 1.25 (m, 36H).

^{13}C NMR (126 MHz, THF-*d*$_8$) δ 162.01, 160.09, 143.45, 139.98, 114.11, 25.67, 20.29.

^{19}F NMR (376 MHz, THF-*d*$_8$) δ –123.54.

^{31}P NMR (162 MHz, THF-*d*$_8$) δ 29.06.

(dCypp)Pd[(4-F)Ph]Br (16a)

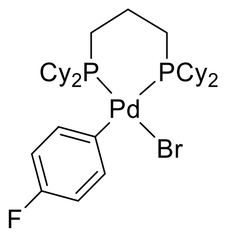

16a

16a was prepared according to the modified General Procedure **B** using *trans*-[(4-F-C_6H_4)Pd(Ph_3P)$_2$Br] **5a** (80.6 mg, 0.1 mmol, 1.0 equiv), dCypp **16** (45.0 mg, 0.10 mmol, 1.0 equiv), and toluene (1.7 mL) with 5 h of reaction time. The precipitate was collected on a filter paper yielding **16a** (64.0 mg, 89%) as a off-white powder. Spectral data are in accordance with literature reports.[vi]

^{1}H NMR (500 MHz, $CDCl_3$): δ 7.34–7.30 (q, J = 7.3 Hz, 2H), 6.83 (m, 2H), 2.35 (4H), 2.20 (m, 2H), 2.03–1.92 (m, 2H), 1.81–1.18 (m, 40H), 1.01–0.97 (m, 2H).

^{13}C NMR (126 MHz, $CDCl_3$): δ 161.9 & 160.0 (d, J = 239.2 Hz, 1C, C–F), 151.7 & 150.6 (d, J = 129.6 Hz, 1C, C-Pd), 137.2 (m, 2C, aryl–C), 114.1–113.9 (m, 2C, aryl–C), 36.37 & 36.15 (d, J = 27.7 Hz, 2C, C–H), 35.62 & 35.46 (d, J = 20.1 Hz, 2C, C–H), 30.6 (d, J = 4.7 Hz, CH_2), 30.5 (d, J = 2.1 Hz, CH_2), 29.0 (d, J = 2.4 Hz, CH_2), 28.7 (d, J = 3.6 Hz, CH_2), 27.7 – 27.5 (m, CH_2), 27.2 – 27.0 (m, CH_2), 26.4 & 26.1 (d, J = 29.4 Hz, CH_2), 22.7 & 22.6 (d, J = 6.24 Hz, CH_2), 19.6 – 19.3 (m, CH_2), 18.1 & 18.0 (dd, J = 3.1, 13.9 Hz, CH_2).

^{19}F NMR (376 MHz, $CDCl_3$): δ –122.97.

^{31}P NMR (162 MHz, $CDCl_3$): δ 23.01 (d, J = 40.0 Hz), 4.19 (d, J = 40.0 Hz).

2.4. Index of Kinetic Experiments

2.4.1. Ligand Effects

Experiment 1: Kinetic Study of the (SPhos)Pd[(4-F)Ph]Br (**3a**) S33
Experiment 2: Kinetic Study of the *(t*-BuXPhos)Pd[(4-F)Ph]Br (**4a**) S50
Experiment 3: Kinetic Study of the *trans*-[(4-F-C_6H_4)Pd(Ph_3P)$_2$Br] (**5a**) S53
Experiment 4: Kinetic Study of the (MePhos)Pd[(4-F)Ph]Br (**6a**) S59
Experiment 5: Kinetic Study of the (dppe)Pd[(4-F)Ph]Br (**7a**) S63
Experiment 6: Kinetic Study of the (XPhos)Pd[(4-F)Ph]Br (**8a**).. S66
Experiment 7: Kinetic Study of the (PhXPhos)Pd[(4-F)Ph]Br (**9a**) S69
Experiment 8: Kinetic Study of the (dppp-dimethyl)Pd[(4-F)Ph]Br (**10a**)............................ S72
Experiment 9: Kinetic Study of the (RuPhos)Pd[(4-F)Ph]Br (**11a**).. S75
Experiment 10: Kinetic Study of the (dppp)Pd[(4-F)Ph]Br (**12a**) S78
Experiment 11: Kinetic Study of the (DavePhos)Pd[(4-F)Ph]Br (**13a**) S81
Experiment 12: Kinetic Study of the (CPhos)Pd[(4-F)Ph]Br (**14a**)....................................... S84
Experiment 13: Kinetic Study of the *trans*-[(4-F-C_6H_4)Pd(i-Pr3P)2Br] (**15a**) S88
Experiment 14: Kinetic Study of the (dCypp)Pd[(4-F)Ph]Br (**16a**)....................................... S93

2.4.2. Halide Effects

Experiment 15: Kinetic Study of the (XPhos)Pd[(4-F)Ph]Cl (**8b**)... S96
Experiment 16: Kinetic Study of the (RuPhos)Pd[(4-F)Ph]I (**11c**).. S99
Experiment 17: Kinetic Study of the (RuPhos)Pd[(4-F)Ph]Cl (**11b**).................................. S102
Experiment 18: Kinetic Study of the (CPhos)Pd[(4-F)Ph]I (**14c**)....................................... S105
Experiment 19: Kinetic Study of the (CPhos)Pd[(4-F)Ph]Cl (**14b**).................................... S108
Experiment 20: Kinetic Study of the (CPhos)Pd[(4-F)Ph]Br (**14a**).................................... S111

2.4.3. Eyring Analysis

Experiment 21: Kinetic Study of the (SPhos)Pd[(4-F)Ph]Br (**3a**) at 0 °C S121
Experiment 22: Kinetic Study of the (SPhos)Pd[(4-F)Ph]Br (**3a**) at 10 °C S124
Experiment 23: Kinetic Study of the *trans*-[(4-F-C_6H_4)Pd(Ph_3P)$_2$Br] (**5a**) at 0 °C S127
Experiment 24: Kinetic Study of the *trans*-[(4-F-C_6H_4)Pd(Ph_3P)$_2$Br] (**5a**) at 10 °C S130
Experiment 25: Kinetic Study of the (CPhos)Pd[(4-F)Ph]Br (**14a**) at –20 °C..................... S133

Equation 1. First-order rate equation

$$[P] = [A]_{\circ}(1 - e^{-kt})$$

Table S1. Summary of Kinetic Data for Transmetalation (Ligand Effect) at –10 °C.

Entry	Ligand	Complex	Run	form k_{obs} ($10^{-2}s^{-1}$)	form k_{avg}, ($10^{-2}s^{-1}$)	k_{rel}
1	SPhos (**3**)	**3a**	1	0.038 ± 0.0009	0.041 ± 0.006	1.00
			2	0.043 ± 0.0003		
2	*t*-BuXPhos (**4**)	**4a**	1	0.08 ± 0.008	0.085 ± 0.005	2.10
			2	0.09 ± 0.009		
3	Ph_3P (**5**)	**5a**	1	0.12 ± 0.002	0.12 ± 0.005	2.84
			2	0.11 ± 0.003		
4	MePhos (**6**)	**6a**	1	1.88 ± 0.12	1.63 ± 0.26	40.07
			2	1.37 ± 0.10		
5	dppe (**7**)	**7a**	1	5.42 ± 0.60	5.09 ± 0.33	125.51
			2	4.76 ± 0.39		
6	XPhos (**8**)	**8a**	1	6.88 ± 0.92	6.22 ± 0.66	153.37
			2	5.56 ± 0.79		
7	PhXPhos (**9**)	**9a**	1	8.59 ± 1.14	7.73 ± 0.86	190.61
			2	6.87 ± 1.10		
8	dppp-dimethyl (**10**)	**10a**	1	8.59 ± 1.95	8.18 ± 0.41	201.70
			2	7.77 ± 0.76		
9	RuPhos (**11**)	**11a**	1	11.39 ± 3.31	11.79 ± 0.40	290.59
			2	12.18 ± 2.59		
10	dppp (**12**)	**12a**	1	15.04 ± 3.35	14.76 ± 0.28	363.95
			2	14.48 ± 7.61		
11	DavePhos (**13**)	**13a**	1	34.28 ± 15.54	32.40 ± 1.88	798.92
			2	30.52 ± 6.92		
12	CPhos (**14**)	**14a**	1	31.61 ± 5.13	38.07 ± 6.46	938.60
			2	44.52 ± 6.00		

Table S2. Summary of Kinetic Data for Transmetalation (Ligand Effect) at 50 °C.

Entry	Ligand	Complex	Run	form k_{obs} ($10^{-2}s^{-1}$)	form k_{avg}, ($10^{-2}s^{-1}$)	k_{rel}
1	*i*Pr_3P (**15**)	**15a**	1	0.005751 ± 0.00012	0.005604 ± 0.0001	1.00
			2	0.005457 ± 0.00016		
2	dCypp (**16**)	**16a**	1	0.0572 ± 0.0048	0.05975 ± 0.0026	10.66
			2	0.0623 ± 0.006		

Table S3. Summary of Kinetic Data for Transmetalation (Halide Effect) at –10 °C.

Entry	Ligand	Complex (Halide)	Run	form k_{obs} ($10^{-2}s^{-1}$)	form k_{avg}, ($10^{-2}s^{-1}$)	k_{rel}
1	RuPhos (**11**)	**11c** (I)	1	5.62 ± 0.67	4.50 ± 1.13	1.00
			2	3.37 ± 0.58		
2	RuPhos (**11**)	**11a** (Br)	1	11.39 ± 3.31	11.79 ± 0.40	2.62
			2	12.18 ± 2.59		
3	RuPhos (**11**)	**11b** (Cl)	1	18.59 ± 0.083	19.61 ± 1.02	4.36
			2	20.62 ± 4.72		
4	XPhos (**8**)	**8a** (Br)	1	6.88 ± 0.92	6.22 ± 0.66	1.00
			2	5.56 ± 0.79		
5	XPhos (**8**)	**8b** (Cl)	1	22.00 ± 8.78	21.58 ± 0.43	3.47
			2	21.15 ± 1.26		

Table S4. Summary of Kinetic Data for Transmetalation (Halide Effect) at –30 °C.

Entry	Ligand	Complex (Halide)	Run	form k_{obs} ($10^{-2}s^{-1}$)	form k_{avg}, ($10^{-2}s^{-1}$)	k_{rel}
1	CPhos (**14**)	**14c** (I)	1	3.02 ± 0.31	3.55 ± 0.53	1.00
			2	4.07 ± 0.48		
2	CPhos (**14**)	**14b** (Cl)	1	13.72 ± 2.71	12.69 ± 1.03	3.58
			2	11.66 ± 2.22		
3	CPhos (**14**)	**14a** (Br)	1	21.65 ± 1.68	19.93 ± 1.73	5.62
			2	18.20 ± 3.25		

Table S5. Summary of Kinetic Data for Transmetalation (Eyring Anylsis)

Entry	Temp (°C)	Ligand	Complex	Run	form k_{obs} ($10^{-2}s^{-1}$)	form k_{avg}, ($10^{-2}s^{-1}$)
1	–10	SPhos (**3**)	**3a**	1	0.038 ± 0.0009	0.041 ± 0.006
				2	0.043 ± 0.0003	
2	0	SPhos (**3**)	**3a**	1	0.27 ± 0.0178	0.28 ± 0.01
				2	0.29 ± 0.0393	
3	10	SPhos (**3**)	**3a**	1	1.16 ± 0.149	1.21 ± 0.05
				2	1.25 ± 0.311	

Table S6. Summary of Kinetic Data for Transmetalation (Eyring Anylsis)

**Entry**	**Temp (°C)**	**Ligand**	**Complex**	**Run**	**form k_{obs} ($10^{-2}s^{-1}$)**	**form k_{avg}, ($10^{-2}s^{-1}$)**
1	–10	Ph_3P (**5**)	**5a**	1	0.12 ± 0.002	0.12 ± 0.005
				2	0.11 ± 0.003	
2	0	Ph_3P (**5**)	**5a**	1	0.32 ± 0.018	0.35 ± 0.03
				2	0.38 ± 0.016	
3	10	Ph_3P (**5**)	**5a**	1	0.93 ± 0.206	0.90 ± 0.04
				2	0.86 ± 0.108	

Table S7. Summary of Kinetic Data for Transmetalation (Eyring Anylsis)

**Entry**	**Temp (°C)**	**Ligand**	**Complex**	**Run**	**form k_{obs} ($10^{-2}s^{-1}$)**	**form k_{avg}, ($10^{-2}s^{-1}$)**
1	–30	CPhos (**14**)	**14a**	1	21.65 ± 1.68	19.93 ± 1.73
				2	18.20 ± 3.25	
2	–20	CPhos (**14**)	**14a**	1	26.99 ± 9.23	28.83 ± 1.84
				2	30.66 ± 7.65	
3	–10	CPhos (**14**)	**14a**	1	31.61 ± 5.13	38.07 ± 6.46
				2	44.52 ± 6.00	

Determination of Order in Oxidative Addition Complex with (CPhos)Pd[(4-F)Ph]Br (**14a**)

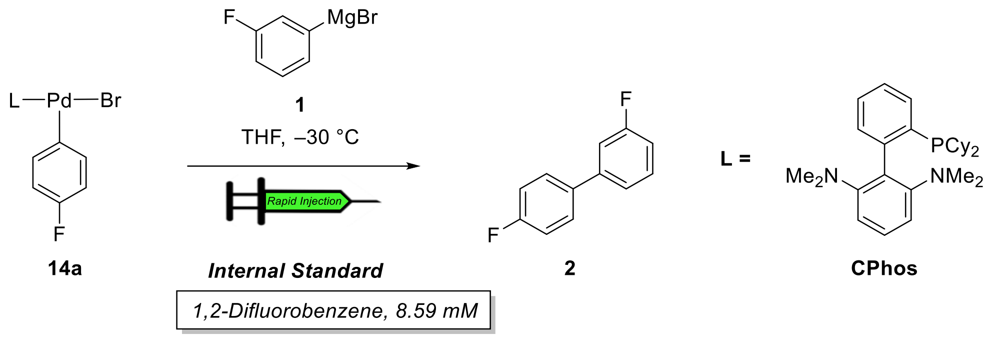

A 5-mL volumetic flask was charged with 1,2-Difluorobenzene (5.0 µL, 50.7 µmol) followed by dissolving with THF to the 5-mL mark generating a 0.01 M of stock solution. An oven-dried (150 °C), 5 mm, NMR tube was taken into the dry box and charged with **14a** (**xx** mg, **xx** µmol) and 500 µL of the freshly prepared solution. The tube was capped with a septum. An oven-dried (60 °C) rapid injection barrel was taken into the glove box and charged with 3-Fluorophenylmagnesium bromide solution (500 µL, 1.33 M in THF). The glass capillary of the barrel was capped with a septum. The sample and the barrel were removed from the glove box and the sample was placed into the NMR probe set to –30 °C with the cap off. Then 3-Fluorophenylmagnesium bromide **1** (120 µmol) in THF (90 µL) was injected (RI-NMR).

Using the fluorine channel to collect a spectrum every 1.6 s the progress of the reaction was monitored by the formation of cross-coupling product in comparison with the internal reference 1,2-difluorobenzene.

**Entry**	**µmol OAC**	**[OAC] (M)**	**Initial rate ($M\ s^{-1}$)**
1	10	0.0169	0.0043
2	20	0.0339	0.0087
3	30	0.0508	0.0142

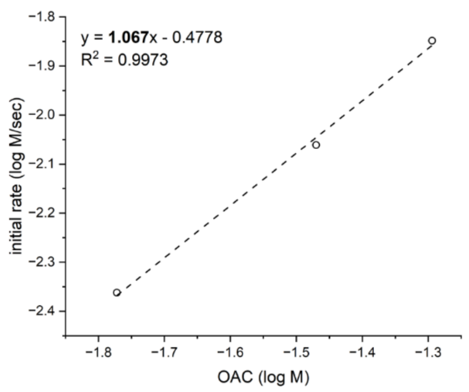

Determination of Order in Grignard Reagent (CPhos)Pd[(4-F)Ph]Br (**14a**)

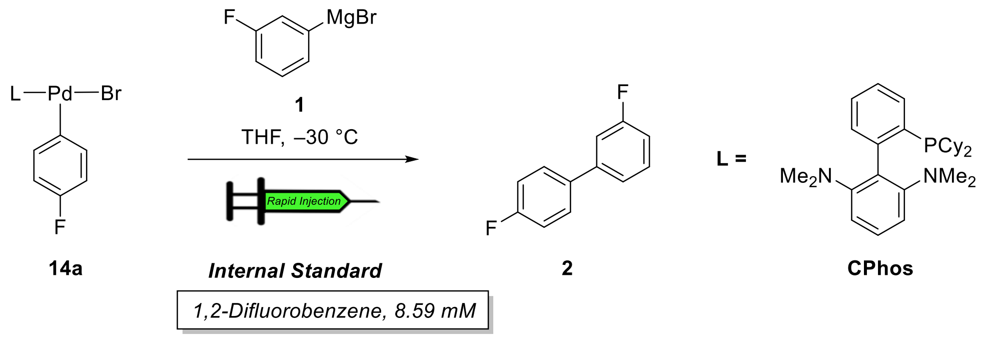

A 5-mL volumetic flask was charged with 1,2-difluorobenzene (5.0 µL, 50.7 µmol) followed by dissolving with THF to the 5-mL mark generating a 0.01 M of stock solution. An oven-dried (150 °C), 5 mm, NMR tube was taken into the dry box and charged with **14a** (7.18 mg, 10.0 µmol) and 500 µL of the freshly prepared solution. The tube was capped with a septum. An oven-dried (60 °C) rapid injection barrel was taken into the glove box and charged with 3-Fluorophenylmagnesium bromide solution (500 µL, 1.0 M or 0.67 M or 0.56 M or 0.44 M or 0.33 M, or 0.22 M in THF). The glass capillary of the barrel was capped with a septum. The sample and the barrel were removed from the glove box and the sample was placed into the NMR probe set to –30 °C with the cap off. Then 3-fluorophenylmagnesium bromide **1** (**xx** µmol) in THF (90 µL) was injected (RI-NMR).

Using the fluorine channel to collect a spectrum every 1.6 s the progress of the reaction was monitored by the formation of cross-coupling product in comparison with the internal reference 1,2-Difluorobenzene.

Entry	µmol ArMgBr	[ArMgBr] (M)	Initial rate (M s^{-1})
1	20	0.034	0.00057
2	30	0.051	0.00084
3	40	0.068	0.00112
4	50	0.085	0.00138
5	60	0.102	0.00168
6	90	0.153	0.00271

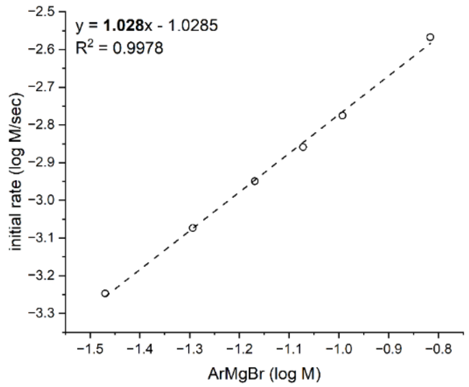

Determination of Order in Grignard Reagent (SPhos)Pd[(4-F)Ph]Br (**3a**)

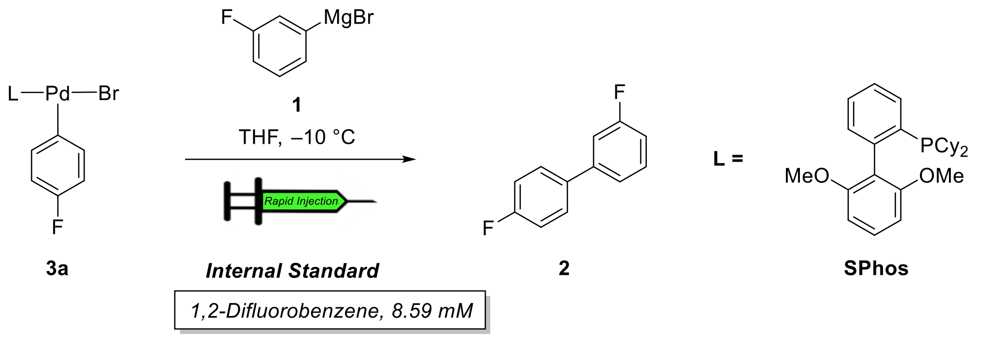

A 5-mL volumetic flask was charged with 1,2-difluorobenzene (5.0 µL, 50.7 µmol) followed by dissolving with THF to the 5-mL mark generating a 0.01 M of stock solution. An oven-dried (150 °C), 5 mm, NMR tube was taken into the dry box and charged with **3a** (6.92 mg, 10.0 µmol) and 500 µL of the freshly prepared solution. The tube was capped with a septum. An oven-dried (60 °C) rapid injection barrel was taken into the glove box and charged with 3-Fluorophenylmagnesium bromide solution (500 µL, 1.0 M or 0.56 M or 0.44 M or 0.33 M in THF). The glass capillary of the barrel was capped with a septum. The sample and the barrel were removed from the glove box and the sample was placed into the NMR probe set to –10 °C with the cap off. Then 3-fluorophenylmagnesium bromide **1** (**xx** µmol) in THF (90 µL) was injected (RI-NMR).

Using the fluorine channel to collect a spectrum every 16 s the progress of the reaction was monitored by the formation of cross-coupling product in comparison with the internal reference 1,2-Difluorobenzene.

**Entry**	**µmol ArMgBr**	**[ArMgBr] (M)**	**Initial rate ($M\ s^{-1}$)**
1	30	0.051	0.0000211
2	40	0.068	0.0000332
3	50	0.085	0.0000392
4	90	0.153	0.0000693

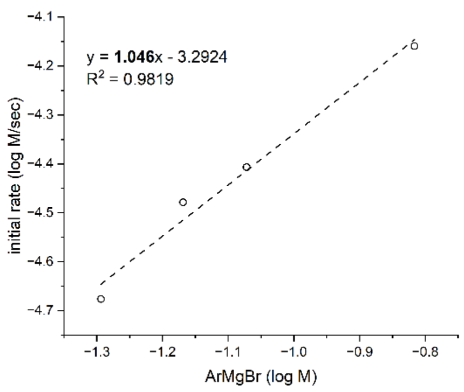

Determination of Order in Grignard Reagent (RuPhos)Pd[(4-F)Ph]Br (**11a**)

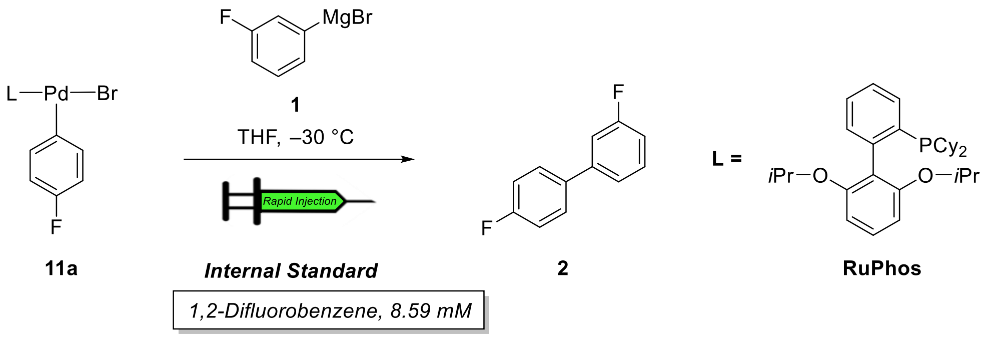

A 5-mL volumetic flask was charged with 1,2-difluorobenzene (5.0 µL, 50.7 µmol) followed by dissolving with THF to the 5-mL mark generating a 0.01 M of stock solution. An oven-dried (150 °C), 5 mm, NMR tube was taken into the dry box and charged with **11a** (7.48 mg, 10.0 µmol) and 500 µL of the freshly prepared solution. The tube was capped with a septum. An oven-dried (60 °C) rapid injection barrel was taken into the glove box and charged with 3-Fluorophenylmagnesium bromide solution (500 µL, 0.67 M or 0.56 M or 0.44 M or 0.22 M in THF). The glass capillary of the barrel was capped with a septum. The sample and the barrel were removed from the glove box and the sample was placed into the NMR probe set to –30 °C with the cap off. Then 3-fluorophenylmagnesium bromide **1** (**xx** µmol) in THF (90 µL) was injected (RI-NMR).

Using the fluorine channel to collect a spectrum every 1.6 s the progress of the reaction was monitored by the formation of cross-coupling product in comparison with the internal reference 1,2-Difluorobenzene.

Entry	µmol ArMgBr	[ArMgBr] (M)	Initial rate ($M\ s^{-1}$)
1	20	0.034	0.000349
3	40	0.068	0.000635
4	50	0.085	0.000788
5	60	0.102	0.001060

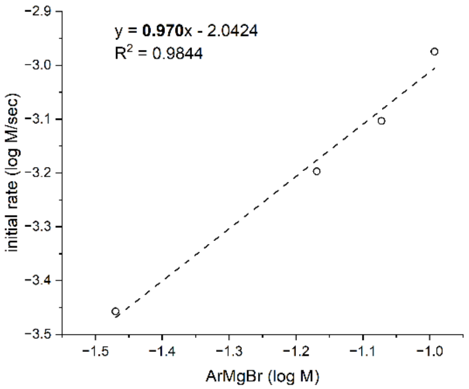

2.4.1. Ligand Effects

Experiment 1: Kinetic Study of the (SPhos)Pd[(4-F)Ph]Br **(3a)**

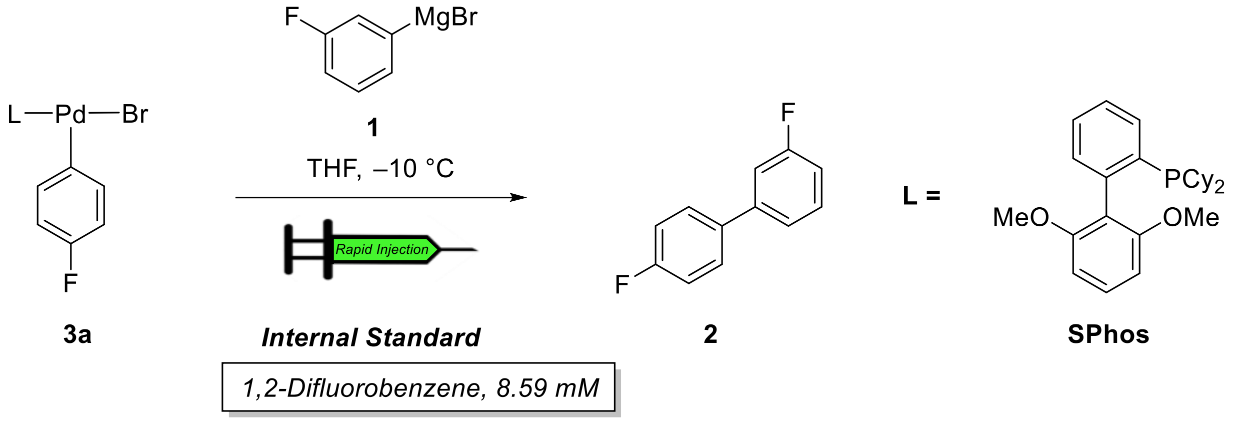

A 5-mL volumetic flask was charged with 1,2-Difluorobenzene (5.0 µL, 50.7 µmol) followed by dissolving with THF to the 5-mL mark generating a 0.01 M of stock solution. An oven-dried (150 °C), 5 mm, NMR tube was taken into the dry box and charged with **3a** (6.92 mg, 10.0 µmol) and 500 µL of the freshly prepared solution. The tube was capped with a septum. An oven-dried (60 °C) rapid injection barrel was taken into the glove box and charged with 3-Fluorophenylmagnesium bromide solution (500 µL, 1.33 M in THF). The glass capillary of the barrel was capped with a septum. The sample and the barrel were removed from the glove box and the sample was placed into the NMR probe set to –10 °C with the cap off. Then 3-Fluorophenylmagnesium bromide **1** (120 µmol) in THF (90 µL) was injected (RI-NMR).

Using the fluorine channel to collect a spectrum every 15 s the progress of the reaction was monitored by the formation of cross-coupling product in comparison with the internal reference 1,2-Difluorobenzene. The first order formation profile was fitted with the Curve Fitter Toolbox in Matlab using Equation 1.

Table S8. Summary of fits for the formation of **2**

**Run**	**R^2 of fit**	***k* ($10^{-2}s^{-1}$) (form CCP[a])**	**$[OAC]_o$ (mM)**
1	0.9668	0.038 ± 0.0009	0.015
2	0.9975	0.043 ± 0.0003	0.016

[a]CCP = cross coupling product

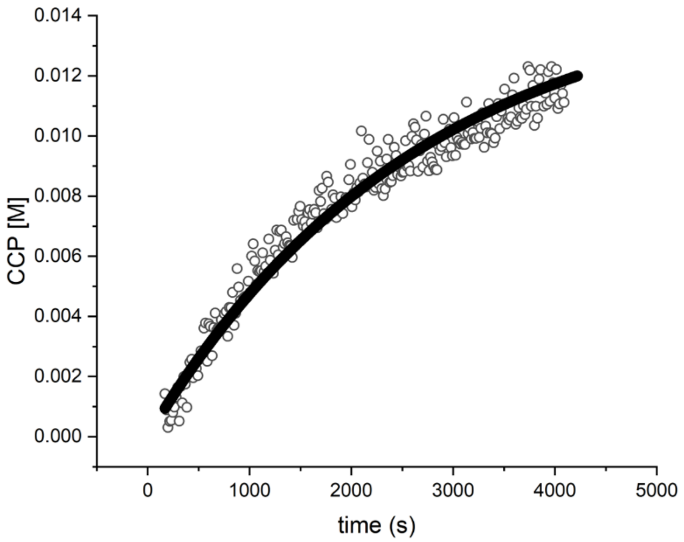

Figure S1. Formation of **2** from complex **3a** at –10 °C (Run 1)

Table S9. Data for the formation of **2** from complex **3a** at –10 °C (Run 1)

Time (s)	Integral IS (−140 ppm)	Integral CCP (−116 ppm)	[CCP] (M)
171	1168530	259330	0.003295
186	1157490	215383	0.002763
201	1160100	170301	0.002180
217	1156950	185535	0.002381
232	1167340	190008	0.002417
248	1142410	205726	0.002674
263	1155770	222138	0.002854
279	1139090	244500	0.003187
294	1147880	271380	0.003510
309	1158100	186008	0.002385
325	1163750	277969	0.003546
340	1142590	230458	0.002995
355	1155180	300604	0.003864
371	1141720	278034	0.003616
386	1186360	227667	0.002849
401	1159880	303951	0.003891
416	1136570	332358	0.004342
432	1109950	332128	0.004443
447	1154680	298106	0.003833
462	1130340	311993	0.004098
477	1131830	315626	0.00414

493	1138730	299147	0.003900
508	1158860	345840	0.004431
523	1132550	360456	0.004725
539	1151670	356653	0.004598
554	1133820	418572	0.005481
569	1072950	408305	0.00565
585	1176140	346321	0.004372
601	1192050	451263	0.005621
617	1113910	415533	0.005539
633	1027740	315990	0.004565
648	1138540	421531	0.005497
663	1176390	474004	0.005983
678	1158640	421615	0.005403
694	1138460	420898	0.005489
709	1121420	413373	0.005473
724	1165390	451152	0.005748
740	1154140	433796	0.005581
755	1181940	474246	0.005957
771	1166590	473620	0.006028
787	1134740	397736	0.005204
802	1165990	484509	0.006170
818	1073500	445239	0.006158
834	1141990	512570	0.006664
849	1139780	427745	0.005572
864	1145380	460382	0.005968
880	1117080	561315	0.007461
895	1129320	520721	0.006846
911	1136760	490379	0.006405
926	1195100	509176	0.006326
943	1092100	477332	0.006489
959	1091810	480079	0.006529
974	1147610	501546	0.006489
989	1050380	498081	0.007041
1004	1170730	525256	0.006661
1021	1066750	565541	0.007871
1036	1102930	615185	0.008282
1052	1092090	567182	0.007711
1067	1101960	514394	0.006931
1082	1135030	564607	0.007386
1099	1119430	554286	0.007352
1114	1135490	565322	0.007392

1129	1105290	594319	0.007984
1144	1129590	559835	0.007359
1159	1117320	566766	0.007531
1175	1136220	559265	0.007308
1190	1089930	619580	0.008440
1206	1119250	583259	0.007737
1221	1110130	552579	0.00739
1236	1160470	570654	0.007301
1252	1122130	609568	0.008065
1267	1073440	631932	0.008741
1283	1096330	584422	0.007915
1298	1151110	674489	0.00870
1313	1081520	636811	0.008742
1329	1148030	631628	0.008169
1345	1107660	618332	0.008288
1360	1094100	627748	0.008519
1375	1076370	602971	0.008317
1390	1099910	609617	0.008229
1406	1143280	632563	0.008215
1421	1125510	593293	0.007827
1436	1090970	665975	0.009064
1451	1116260	617779	0.008217
1467	1097400	672532	0.009099
1482	1076760	678742	0.009359
1497	1113510	714452	0.009526
1512	1117920	683950	0.009084
1527	1145470	685671	0.008888
1543	1168190	709891	0.009023
1560	1108650	658943	0.008825
1575	1153380	723163	0.009309
1591	1128190	715681	0.009419
1606	1127380	681714	0.008978
1621	1119150	698453	0.009266
1637	1124200	713563	0.009424
1652	1144500	717852	0.009313
1667	1149170	682633	0.00882
1683	1123960	760976	0.010052
1698	1097740	716342	0.009689
1713	1077940	735879	0.010136
1730	1111090	694716	0.009283
1745	1104640	676878	0.009098

1760	1108050	785698	0.010528
1775	1134090	789598	0.010337
1791	1121740	695516	0.009206
1807	1102070	699923	0.00943
1822	1150150	767673	0.00991
1838	1146690	756993	0.009802
1853	1124550	693502	0.009156
1868	1130440	723304	0.0095
1884	1113670	703527	0.009379
1899	1160480	769531	0.009846
1916	1157910	724297	0.009287
1931	1124880	732884	0.009673
1947	1126740	744991	0.009817
1962	1147320	756318	0.009788
1978	1144210	802123	0.010408
1993	1079990	794300	0.01092
2008	1140610	730759	0.009512
2024	1098360	737925	0.009975
2040	1133410	743779	0.009743
2055	1109750	759483	0.010161
2070	1078150	726268	0.010002
2085	1089270	755754	0.010301
2100	1068600	865927	0.012031
2115	1118190	788247	0.010466
2131	1127240	780447	0.01028
2146	1160070	794882	0.010174
2161	1113970	823836	0.01098
2176	1032960	817770	0.011754
2192	1122750	777983	0.010288
2207	1168210	838280	0.010654
2223	1135540	769891	0.010067
2238	1122630	767937	0.010156
2254	1117360	855361	0.011366
2269	1147070	794206	0.01028
2285	1112270	826394	0.011031
2301	1132280	771219	0.010113
2316	1152670	767391	0.009885
2332	1142910	778249	0.01011
2347	1149720	859606	0.011101
2363	1128060	892758	0.01175
2379	1136420	790219	0.010324

2394	1153180	803786	0.010349
2409	1125930	799674	0.010545
2424	1102640	853410	0.011491
2440	1146000	865626	0.011215
2456	1129010	805382	0.010591
2471	1145630	835227	0.010825
2487	1131820	803444	0.01054
2502	1128270	809660	0.010655
2518	1124960	808540	0.010671
2533	1163310	917452	0.01171
2549	1166310	852263	0.01085
2564	1126930	826143	0.010885
2580	1120720	807667	0.0107
2595	1095180	874528	0.011856
2610	1135310	938660	0.012276
2625	1124530	920794	0.012157
2641	1109490	876866	0.011734
2656	1160320	835368	0.010689
2672	1136630	870830	0.011375
2688	1135810	886117	0.011583
2703	1153540	841811	0.010835
2719	1080560	868475	0.011933
2734	1127880	951410	0.012524
2749	1120650	832764	0.011033
2764	1141460	823528	0.010712
2779	1113040	836316	0.011156
2795	1130820	838109	0.011004
2810	1134180	828998	0.010852
2825	1181980	855630	0.010748
2841	1170960	847508	0.010746
2857	1130090	879076	0.01155
2872	1145990	878957	0.011388
2887	1102480	854834	0.011512
2903	1089740	911751	0.012422
2918	1144820	897647	0.011642
2934	1135160	854188	0.011172
2949	1082640	875337	0.012004
2964	1112270	905911	0.012093
2979	1102200	852656	0.011486
2994	1146380	865493	0.01121
3010	1128520	948490	0.012479

3025	1130180	854417	0.011225
3040	1131380	900226	0.011814
3055	1143840	903139	0.011723
3071	1117170	872840	0.0116
3086	1166560	916168	0.011661
3101	1157350	903214	0.011587
3117	1137360	887078	0.01158
3132	1114940	975407	0.012989
3147	1131900	911099	0.011951
3162	1137420	928886	0.012125
3179	1161870	921088	0.011771
3194	1109580	895306	0.01198
3209	1112880	883717	0.01179
3225	1119800	889324	0.011792
3241	1160330	965552	0.012355
3258	1154930	919629	0.011822
3273	1131060	923747	0.012126
3288	1117040	950826	0.012638
3305	1130430	874626	0.011488
3320	1123130	922397	0.012194
3336	1121860	904773	0.011974
3351	1125320	980749	0.01294
3366	1155870	932174	0.011974
3381	1106380	889791	0.011941
3397	1142270	895798	0.011644
3412	1136060	902921	0.011801
3427	1138920	959818	0.012513
3443	1119960	976502	0.012946
3459	1110580	906066	0.012113
3474	1087950	930272	0.012696
3490	1122780	977593	0.012928
3506	1112780	1006050	0.013423
3522	1133790	936373	0.012262
3537	1104990	926525	0.012449
3553	1114530	930762	0.012399
3569	1113820	937640	0.012499
3584	1121760	999713	0.013232
3599	1100390	1022060	0.013791
3615	1122010	926375	0.012259
3630	1092210	908904	0.012356
3645	1096590	946144	0.01281

3660	1130520	951203	0.012492
3675	1101980	923914	0.012448
3691	1103540	957558	0.012883
3706	1092300	925498	0.01258
3721	1104270	948628	0.012755
3737	1032720	985870	0.014174
3753	1039160	983723	0.014055
3769	1094040	947570	0.01286
3784	1105080	1008460	0.013549
3799	1108400	912589	0.012224
3815	1095000	948441	0.01286
3830	1092520	916634	0.012457
3845	1072870	994291	0.01376
3860	1080320	1023610	0.014068
3875	1114380	1005550	0.013397
3891	1092630	945901	0.012854
3907	1086630	972694	0.013291
3922	1093160	950739	0.012913
3937	1089030	1027010	0.014002
3953	1103170	967551	0.013022
3968	1082160	1033110	0.014174
3983	1081940	993663	0.013636
3999	1084920	959332	0.013129
4014	1063600	1008780	0.014082
4029	1075530	926146	0.012785
4044	1081260	942169	0.012938
4060	1070370	978504	0.013573
4075	1066030	953397	0.013279

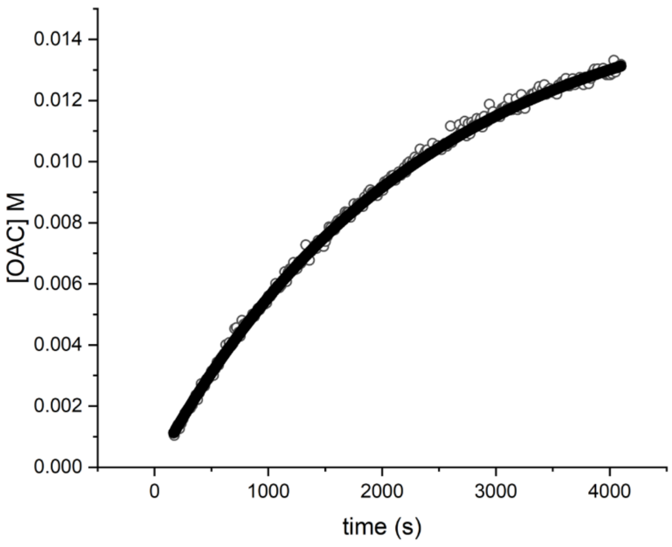

Figure S2. Formation of **2** from complex **3a** at –10 °C (Run 2)

Table S10. Data for the formation of **2** from complex **3a** at –10 °C (Run 2)

Time (s)	Integral IS (−140 ppm)	Integral CCP (−116 ppm)	[CCP] (M)
175	2163730	287180	0.00213259
190	2166390	319604	0.002370456
206	2170480	335406	0.002482969
221	2212610	324432	0.002355999
237	2189780	344409	0.002527146
252	2246970	373252	0.002669078
269	2206530	393248	0.002863604
284	2225890	407046	0.0029383
300	2252420	423874	0.003023735
316	2178160	410482	0.003028033
332	2184290	423624	0.003116209
348	2218060	458715	0.003322967
364	2219030	476036	0.003446934
380	2178080	447362	0.00330021
395	2184740	479633	0.003527489
411	2180650	518737	0.003822237
426	2152220	500554	0.003736978
441	2176990	507245	0.003743843
457	2168080	535692	0.003970052
472	2161160	534885	0.003976764
488	2201790	553076	0.004036131

503	2152210	567758	0.004238722
518	2223410	566219	0.004091864
534	2182180	584381	0.004302906
550	2170830	611631	0.004527099
565	2205990	608913	0.004435147
582	2180070	630224	0.004644948
597	2182740	640141	0.004712268
613	2149150	641447	0.004795682
628	2143800	679361	0.005091816
643	2159270	665570	0.004952712
659	2161500	694199	0.00516042
674	2229140	700724	0.005050867
691	2205700	704739	0.005133791
706	2201250	769659	0.005618046
722	2208800	777291	0.005654362
739	2197020	752458	0.005503064
754	2222890	743525	0.005374449
770	2166170	794550	0.005893659
785	2154520	773658	0.005769721
801	2162650	778752	0.005785878
816	2169730	789213	0.005844466
832	2179590	800539	0.005901522
847	2161970	799496	0.005941867
862	2140650	812088	0.006095562
878	2238690	839375	0.006024464
894	2155880	825249	0.00615059
910	2191850	857406	0.006285387
925	2192290	851911	0.006243851
941	2177090	859381	0.006342576
956	2171720	867865	0.00642103
972	2157740	863117	0.006427275
987	2184230	878953	0.00646582
1003	2171920	904885	0.006694311
1018	2206400	916700	0.006675738
1034	2208540	928947	0.00675837
1050	2204400	945412	0.006891076
1066	2138760	944911	0.007098804
1082	2178610	951790	0.007019691
1098	2214940	959771	0.006962448
1114	2196020	962171	0.007039994
1130	2193740	972914	0.007125997

1145	2156680	1004530	0.007483996
1161	2190510	977265	0.00716842
1176	2138900	995198	0.007476104
1192	2145090	1012910	0.007587202
1207	2159910	1024060	0.00761809
1222	2168330	1050400	0.007783692
1238	2202010	1036280	0.007561608
1253	2195650	1035830	0.007580218
1268	2171300	1055620	0.007811674
1284	2160320	1042450	0.007753423
1299	2162430	1051910	0.007816149
1315	2213370	1091120	0.007920905
1331	2189320	1140010	0.00836673
1347	2215940	1089550	0.007900335
1363	2197150	1075270	0.007863468
1378	2136220	1084160	0.00815462
1394	2185420	1123550	0.008260642
1409	2150490	1117250	0.008347747
1424	2182750	1119740	0.008242701
1440	2166040	1146700	0.00850628
1455	2165880	1137070	0.008435467
1471	2204260	1155350	0.008421842
1487	2206490	1141910	0.008315459
1503	2229880	1175740	0.008472004
1519	2163660	1162520	0.008633119
1535	2169430	1208160	0.008948189
1551	2157660	1199000	0.008928788
1566	2124950	1173060	0.008870086
1582	2190720	1207930	0.008859541
1597	2152100	1213680	0.009061458
1612	2126810	1213280	0.009166186
1628	2175270	1236570	0.009134018
1644	2123470	1219670	0.009228955
1660	2191650	1250930	0.00917103
1676	2122650	1247970	0.009446743
1692	2130920	1248730	0.009415811
1707	2109840	1239180	0.009437157
1722	2170820	1251310	0.009261843
1738	2144730	1256450	0.009413018
1753	2144870	1295630	0.009705912
1769	2163850	1280120	0.009505607

1785	2196690	1326240	0.009700847
1802	2146370	1299700	0.009729597
1817	2129500	1290380	0.009736353
1832	2172090	1300430	0.009619788
1848	2151090	1329480	0.009930693
1864	2191860	1345570	0.009863926
1881	2170170	1362950	0.010091193
1897	2155960	1363660	0.010162995
1913	2128130	1335710	0.010084871
1928	2118700	1331080	0.010094644
1944	2134530	1337730	0.010069839
1959	2176240	1351180	0.009976145
1975	2163730	1359740	0.01009739
1992	2131500	1360250	0.010253915
2007	2166130	1367940	0.010147028
2023	2161160	1402980	0.010430878
2039	2119160	1373030	0.010410524
2054	2131460	1392810	0.010499558
2070	2140920	1391520	0.010443482
2085	2119860	1401920	0.010626063
2101	2104800	1393980	0.01064148
2116	2131860	1390290	0.010478595
2132	2153430	1421300	0.010605016
2147	2119230	1418130	0.010752124
2163	2175770	1441000	0.01064161
2179	2168660	1445100	0.010706876
2195	2131400	1445570	0.010897591
2210	2173130	1452780	0.010741637
2226	2119720	1455820	0.011035335
2241	2176450	1500700	0.011079024
2257	2173610	1487240	0.010994001
2273	2132380	1475760	0.011120068
2289	2118910	1481380	0.011233376
2305	2133020	1479160	0.011142344
2320	2139350	1491590	0.011202732
2336	2082080	1489490	0.01149467
2351	2148750	1498810	0.011207713
2367	2134210	1503020	0.011315765
2382	2109400	1500080	0.011426463
2398	2129990	1521330	0.011476308
2414	2139970	1513470	0.011363771

2429	2157740	1524670	0.011353586
2445	2133320	1550980	0.011681713
2460	2126770	1515530	0.011449864
2475	2121270	1513730	0.011465917
2491	2119400	1531250	0.011608858
2507	2133600	1532660	0.011542215
2522	2134480	1524710	0.011477611
2537	2136600	1544880	0.011617906
2553	2148950	1562810	0.011685201
2569	2191570	1581530	0.011595205
2585	2125390	1543920	0.011671925
2601	2107050	1607230	0.012256304
2616	2149010	1567210	0.011717773
2632	2150600	1585090	0.011842697
2648	2146480	1587470	0.011883244
2664	2163390	1604790	0.011918997
2679	2131020	1630320	0.012292541
2695	2146660	1613330	0.01207581
2711	2136180	1616190	0.012156566
2726	2079510	1606090	0.012409812
2742	2162670	1606060	0.011932401
2758	2125690	1632820	0.01234226
2773	2148550	1604320	0.011997807
2789	2099580	1616870	0.012373683
2804	2091180	1599070	0.012286619
2819	2139370	1635950	0.012286847
2835	2168310	1687090	0.012501819
2851	2144420	1651700	0.012375924
2866	2114890	1644570	0.012494558
2882	2141570	1628380	0.012217429
2897	2140960	1674410	0.012566362
2913	2148800	1655110	0.012376196
2929	2120440	1638200	0.012413586
2944	2100960	1695730	0.012968664
2960	2157140	1690750	0.012593817
2976	2084230	1650000	0.01272022
2991	2144750	1676950	0.012563185
3007	2122060	1664210	0.012601052
3022	2144140	1688520	0.012653463
3037	2174830	1699680	0.012557355
3054	2117100	1690190	0.01282775

3069	2134340	1703180	0.012821926
3085	2100920	1686940	0.012901685
3100	2115530	1688690	0.012825877
3115	2072720	1696160	0.013148691
3131	2162780	1720630	0.012782961
3146	2153810	1714080	0.012787334
3162	2118420	1703260	0.01291889
3177	2102910	1720280	0.013144219
3192	2153210	1713660	0.012787764
3208	2163850	1731180	0.01285498
3224	2154530	1780750	0.013280265
3240	2131300	1729260	0.013036831
3256	2148690	1715810	0.012830742
3271	2127100	1735550	0.013110086
3287	2150920	1758960	0.013139778
3303	2146130	1759260	0.013171351
3318	2129240	1762100	0.013297263
3334	2152080	1782140	0.013305761
3349	2139820	1755980	0.013185562
3365	2148630	1794340	0.01341836
3380	2125190	1790970	0.013540879
3396	2138470	1766070	0.013269699
3411	2108020	1767130	0.013469457
3427	2153520	1823100	0.013602474
3443	2174350	1798920	0.013293481
3459	2115830	1768300	0.013428624
3474	2125340	1783120	0.013480577
3490	2141360	1788520	0.013420245
3505	2126080	1766650	0.013351413
3521	2143840	1792250	0.013432676
3536	2172050	1797230	0.013295056
3552	2132000	1804030	0.013596054
3567	2136320	1804620	0.013572998
3584	2118010	1806880	0.01370748
3599	2111930	1799060	0.013687447
3615	2110100	1813140	0.013806533
3630	2146810	1821220	0.013630919
3646	2139190	1805780	0.013563501
3661	2144120	1815850	0.013607778
3677	2134760	1832890	0.013795698
3692	2147130	1817520	0.013601199

3708	2106750	1800770	0.013734143
3724	2110540	1806300	0.013751581
3739	2111960	1819850	0.013845423
3755	2143060	1840380	0.013798424
3770	2174370	1840640	0.013601654
3786	2107720	1816830	0.013850253
3802	2170760	1852660	0.013713245
3817	2151970	1826720	0.0136393
3833	2159750	1855720	0.013805918
3850	2147540	1871330	0.014001206
3866	2103810	1848340	0.014116651
3881	2115770	1838210	0.013959922
3896	2104690	1850080	0.014124032
3912	2133230	1856580	0.013984029
3927	2107240	1840860	0.014036638
3943	2130650	1875350	0.014142512
3958	2126000	1854920	0.014019039
3973	2128020	1845020	0.013930981
3989	2143300	1857240	0.013923275
4004	2159150	1871160	0.013924655
4021	2150860	1870390	0.013972572
4036	2084540	1868410	0.01440185
4051	2159740	1884160	0.014017567
4067	2161090	1904210	0.014157883
4084	2122470	1879150	0.014225784

Experiment 2: Kinetic Study of the (t-BuXPhos)Pd[(4-F)Ph]Br **(4a)**

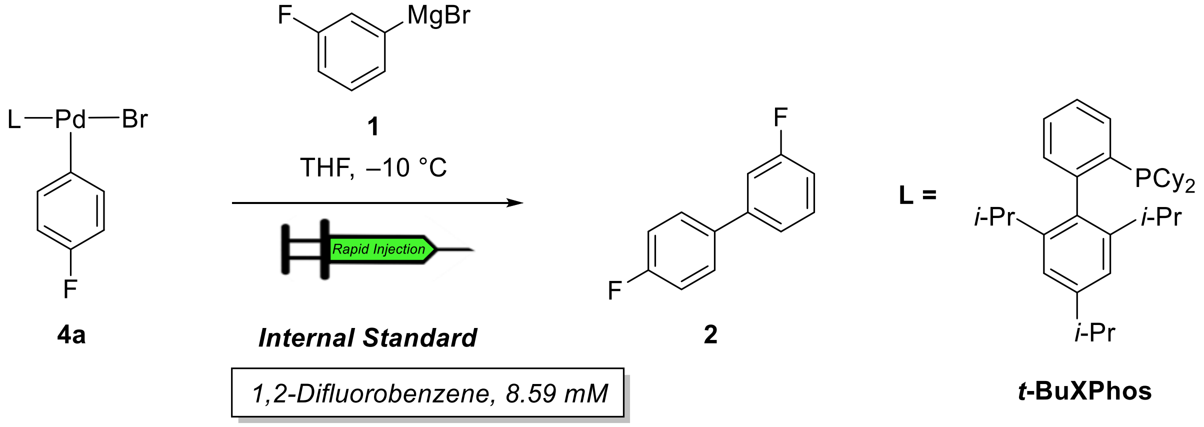

A 5-mL volumetic flask was charged with 1,2-Difluorobenzene (5.0 µL, 50.7 µmol) followed by dissolving with THF to the 5-mL mark generating a 0.01 M of stock solution. An oven-dried (150 °C), 5 mm, NMR tube was taken into the dry box and charged with **4a** (7.1 mg, 10.0 µmol) and 500 µL of the freshly prepared solution. The tube was capped with a septum. An oven-dried (60 °C) rapid injection barrel was taken into the glove box and charged with 3-Fluorophenylmagnesium bromide solution (500 µL, 1.33 M in THF). The glass capillary of the barrel was capped with a septum. The sample and the barrel were removed from the glove box and the sample was placed into the NMR probe set to –10 °C with the cap off. Then 3-Fluorophenylmagnesium bromide **1** (120 µmol) in THF (90 µL) was injected (RI-NMR).

Using the fluorine channel to collect a spectrum every 31 s the progress of the reaction was monitored by the formation of cross-coupling product in comparison with the internal reference 1,2-Difluorobenzene. The first order formation profile was fitted with the Curve Fitter Toolbox in Matlab using Equation 1.

Table S11. Summary of fits for the formation of **2**

Run	R^2 of fit	k ($10^{-2}s^{-1}$) (form CCP)	$[OAC]_o$ (mM)
1	0.9398	0.08 ± 0.008	0.0158 ± 0.00058
2	0.9256	0.09 ± 0.009	0.0157 ± 0.00044

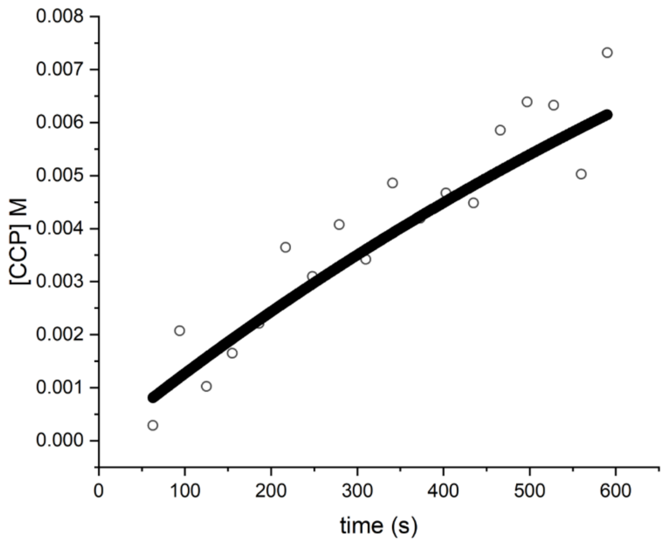

Fig S3. Formation of **2** from complex **4a** at − 10°C in (Run 1).

Table S12. Data for the formation of **2** from complex **4a** at –10 °C (Run 1)

Time (s)	Integral IS (−140 ppm)	Integral CCP (−116 ppm)	[CCP] (M)
63	1343710	123850	0.001368
94	1415340	300790	0.003155
125	1405370	199140	0.002104
155	1307830	240510	0.00273
186	1314680	291553	0.003293
217	1303430	414984	0.004727
248	1329140	373804	0.004176
279	1350530	469007	0.005156
310	1384940	419921	0.004502
341	1321100	528263	0.005937
373	1346900	478534	0.005275
403	1297060	502347	0.00575
435	1380640	517265	0.005563
466	1352510	631956	0.006937
497	1378760	693743	0.007471
528	1383640	690254	0.007407
560	1337070	549868	0.006106
590	1329410	751841	0.008397

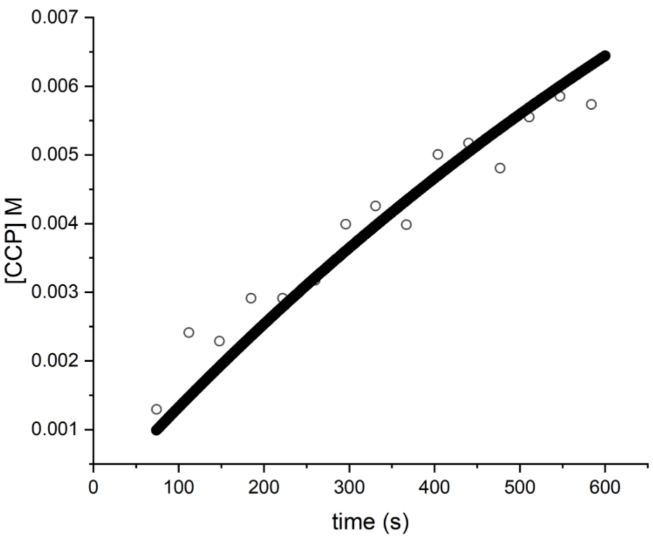

Figure S4. Formation of **2** from complex **4a** at –10 °C (Run 2)

Table S13. Data for the formation of **2** from complex **4a** at –10 °C (Run 2)

**Time (s)**	**Integral IS (−140 ppm)**	**Integral CCP (−116 ppm)**	**[CCP] (M)**
74	1822540	311351	0.002484
112	1935320	479521	0.003603
148	2089570	499475	0.003476
185	2131000	601167	0.004102
222	2111620	595836	0.004103
260	2167810	650583	0.004364
296	2152940	767135	0.005182
331	2224100	832916	0.005446
367	2225960	792167	0.005175
404	2196930	936265	0.006198
440	2255480	986818	0.006363
477	2280220	940264	0.005997
511	2239130	1038200	0.006743
547	2305100	1116130	0.007041
584	2298140	1094610	0.006927

Experiment 3: Kinetic Study of the *trans*-[(4-F-C_6H_4)Pd(Ph_3P)$_2$Br] **(5a)**

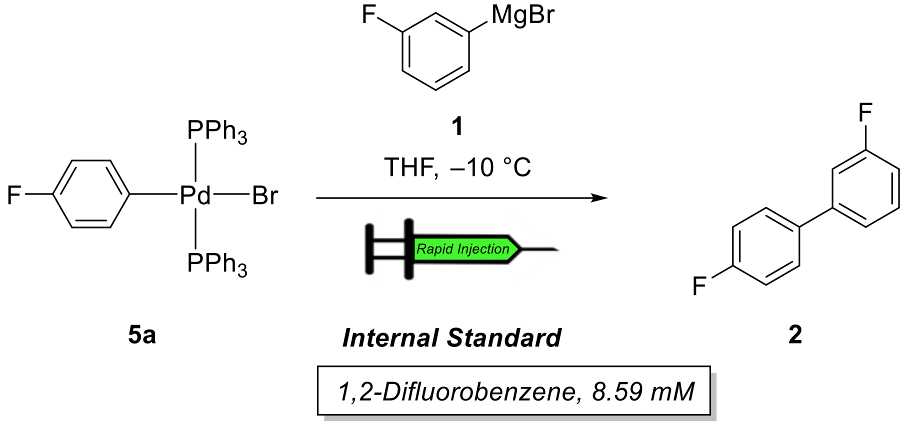

A 5-mL volumetic flask was charged with 1,2-Difluorobenzene (5.0 µL, 50.7 µmol) followed by dissolving with THF to the 5-mL mark generating a 0.01 M of stock solution. An oven-dried (150 °C), 5 mm, NMR tube was taken into the dry box and charged with **5a** (8.06 mg, 10.0 µmol) and 500 µL of the freshly prepared solution. The tube was capped with a septum. An oven-dried (60 °C) rapid injection barrel was taken into the glove box and charged with 3-Fluorophenylmagnesium bromide solution (500 µL, 1.33 M in THF). The glass capillary of the barrel was capped with a septum. The sample and the barrel were removed from the glove box and the sample was placed into the NMR probe set to –10 °C with the cap off. Then 3-Fluorophenylmagnesium bromide **1** (120 µmol) in THF (90 µL) was injected (RI-NMR).

Using the fluorine channel to collect a spectrum every 15 s the progress of the reaction was monitored by the formation of cross-coupling product in comparison with the internal reference 1,2-Difluorobenzene. The first order formation profile was fitted with the Curve Fitter Toolbox in Matlab using Equation 1.

Table S14. Summary of fits for the formation of **2**

**Run**	**R^2 of fit**	***k*** **($10^{-2}s^{-1}$) (form CCP)**	**$[OAC]_o$ (mM)**
1	0.9952	0.12 ± 0.002	0.0159 ± 0.00013
2	0.9927	0.11 ± 0.003	0.0157 ± 0.00016

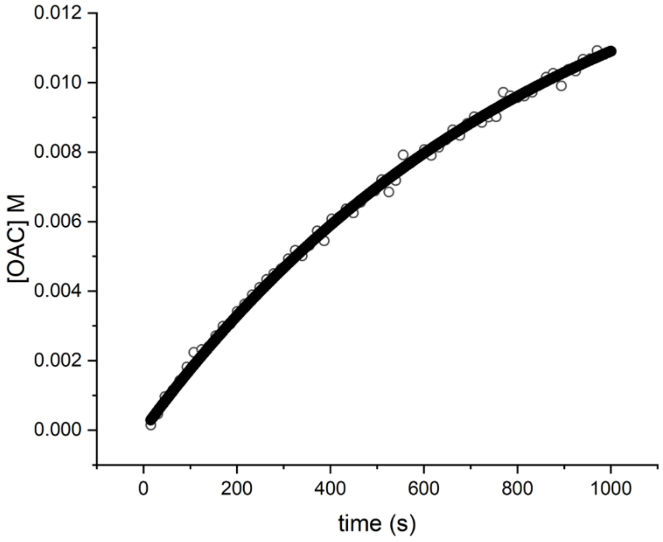

Figure S5. Formation of **2** from complex **5a** at –10 °C (Run 1)

Table S15. Data for the formation of **2** from complex **5a** at –10 °C (Run 1)

**Time (s)**	**Integral IS (−140 ppm)**	**Integral CCP (−116 ppm)**	**[CCP] (M)**
16	1737500	122267	0.00115
31	1755400	157060	0.001462
46	1839860	220657	0.00196
63	1831880	240341	0.002144
78	1841390	273186	0.002424
93	1822860	314105	0.002815
108	1843090	365185	0.003237
125	1884850	382875	0.003319
140	1870620	389737	0.003404
155	1863020	423773	0.003717
170	1843200	449780	0.003987
186	1881780	466607	0.004051
201	1825010	492931	0.004413
217	1883690	533802	0.00463
233	1884060	564394	0.004895
249	1827590	570857	0.005104
264	1822360	595178	0.005336
279	1868180	629590	0.005506
295	1853530	640161	0.005643
310	1827960	663595	0.005931

325	1839960	694735	0.006169
340	1853160	681910	0.006012
355	1832010	708117	0.006315
372	1843700	759589	0.006732
387	1861120	734320	0.006447
403	1872990	811466	0.007079
419	1831590	799532	0.007132
434	1811640	816850	0.007367
449	1822720	808476	0.007247
465	1853870	857612	0.007559
480	1856930	882687	0.007767
495	1838760	887191	0.007883
510	1841270	924898	0.008207
525	1856270	891899	0.007851
540	1858370	930776	0.008183
556	1798020	980964	0.008914
571	1851380	982619	0.008672
586	1820550	984995	0.00884
601	1810000	1004610	0.009069
616	1847860	1006980	0.008904
632	1850660	1034860	0.009137
647	1831460	1047950	0.009349
662	1808280	1066880	0.00964
677	1841950	1068760	0.00948
693	1819340	1093430	0.00982
708	1806530	1107400	0.010016
724	1876680	1131910	0.009855
739	1830200	1120810	0.010006
755	1878050	1151380	0.010017
770	1786080	1171880	0.01072
785	1808770	1175360	0.010617
800	1821920	1178390	0.010568
815	1827770	1187180	0.010613
832	1832210	1202560	0.010724
847	1825000	1221900	0.01094
862	1792350	1223260	0.011151
877	1815430	1251540	0.011264
894	1899220	1267220	0.010902
909	1841260	1281720	0.011374
925	1855940	1286860	0.011329
941	1810360	1293370	0.011673

956	1851400	1323680	0.011682
971	1822110	1329140	0.011918
986	1843880	1332840	0.011811
1001	1824320	1328080	0.011895

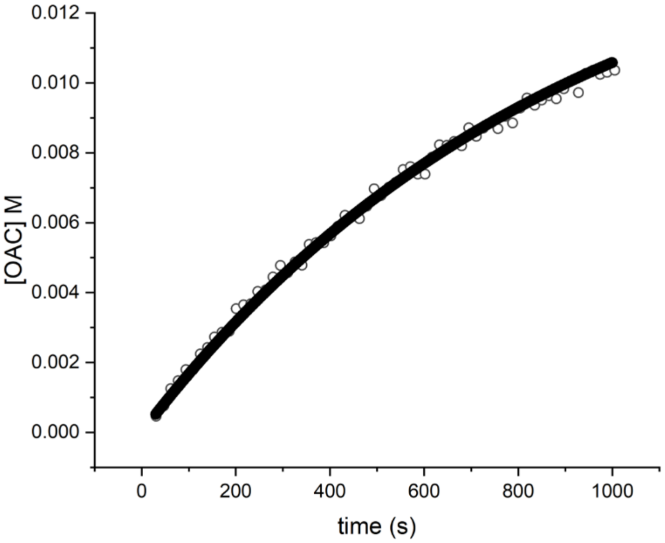

Figure S6. Formation of **2** from complex **5a** at –10 °C (Run 2)

Table S16. Data for the formation of **2** from complex **5a** at –10 °C (Run 2)

Time (s)	Integral IS (−140 ppm)	Integral CCP (−116 ppm)	[CCP] (M)
16	1503490	96763	0.001052
31	1521750	155471	0.001669
47	1572320	189640	0.001971
62	1575930	237412	0.002461
78	1591030	261867	0.002689
94	1600470	294040	0.003002
109	1597580	293407	0.003001
125	1592250	336436	0.003452
140	1586580	353451	0.00364
155	1628840	392986	0.003942
171	1614910	402156	0.004069

186	1677010	420943	0.004101
201	1612330	468781	0.004751
217	1583420	470802	0.004858
232	1588670	475017	0.004885
247	1611950	517230	0.005243
264	1597180	516609	0.005285
279	1604830	555696	0.005658
295	1615980	592043	0.005986
310	1573790	556693	0.00578
326	1596550	595235	0.006092
341	1606720	589222	0.005992
356	1564700	631106	0.00659
371	1592930	647351	0.00664
387	1591670	646313	0.006635
402	1588230	664197	0.006833
417	1562910	679539	0.007104
432	1577500	716291	0.007419
448	1606130	725931	0.007385
463	1607140	720520	0.007325
478	1564770	735390	0.007679
494	1583670	792488	0.008176
509	1583390	773684	0.007984
525	1574830	792174	0.008219
540	1582170	809689	0.008362
556	1546320	826537	0.008733
571	1556620	839408	0.008811
587	1597680	840734	0.008598
602	1604480	844340	0.008598
617	1566820	870869	0.009082
633	1568430	906071	0.009439
649	1545390	891488	0.009425
664	1590710	927743	0.009529
680	1577730	908441	0.009408
695	1530640	929270	0.00992
711	1591990	943441	0.009683
726	1586110	962738	0.009917
742	1575340	969969	0.01006
757	1605370	972794	0.009901
773	1568020	984276	0.010256
788	1595400	982276	0.01006
804	1562070	1002820	0.010489

819	1582550	1043570	0.010774
835	1572560	1016980	0.010566
850	1561430	1024470	0.01072
866	1599670	1061490	0.010842
881	1585700	1043690	0.010754
897	1597850	1079460	0.011038
912	1579170	1088300	0.01126
928	1594380	1066280	0.010927
943	1523180	1069640	0.011474
958	1573810	1113760	0.011563
974	1606820	1126030	0.01145
989	1591940	1121870	0.011514
1005	1563970	1107710	0.011572

Experiment 4: Kinetic Study of the (MePhos)Pd[(4-F)Ph]Br **(6a)**

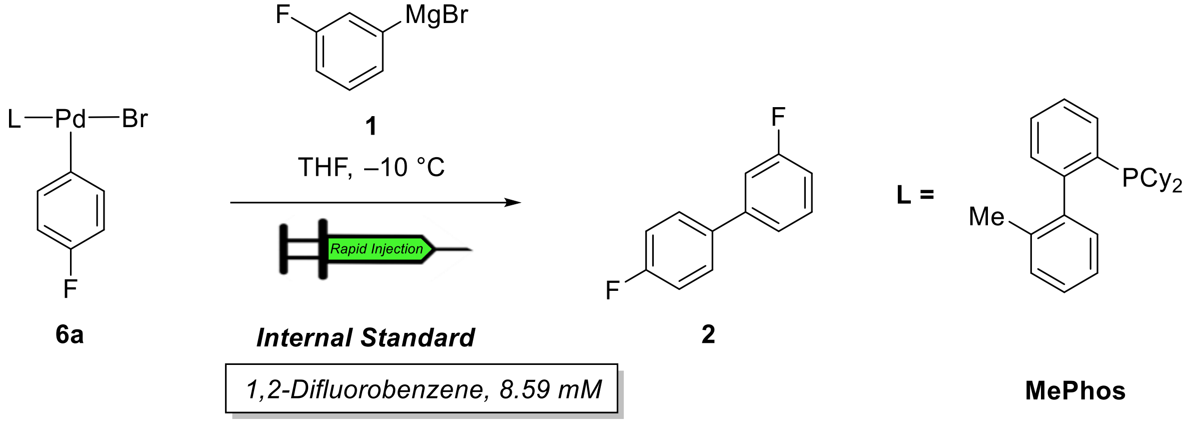

A 5-mL volumetic flask was charged with 1,2-Difluorobenzene (5.0 µL, 50.7 µmol) followed by dissolving with THF to the 5-mL mark generating a 0.01 M of stock solution. An oven-dried (150 °C), 5 mm, NMR tube was taken into the dry box and charged with **6a** (6.46 mg, 10 µmol) and 500 µL of the freshly prepared solution. The tube was capped with a septum. An oven-dried (60 °C) rapid injection barrel was taken into the glove box and charged with 3-Fluorophenylmagnesium bromide solution (500 µL, 1.33 M in THF). The glass capillary of the barrel was capped with a septum. The sample and the barrel were removed from the glove box and the sample was placed into the NMR probe set to –10 °C with the cap off. Then 3-Fluorophenylmagnesium bromide **1** (120 µmol) in THF (90 µL) was injected (RI-NMR).

Using the fluorine channel to collect a spectrum every 1.6 s the progress of the reaction was monitored by the formation of cross-coupling product in comparison with the internal reference 1,2-Difluorobenzene. The first order formation profile was fitted with the Curve Fitter Toolbox in Matlab using Equation 1.

Table S17. Summary of fits for the formation of **2**

**Run**	**R^2 of fit**	***k* ($10^{-2}s^{-1}$) (form CCP)**	**$[OAC]_o$ (mM)**
1	0.9831	1.88 ± 0.12	0.0166 ± 0.00032
2	0.9654	1.37 ± 0.10	0.0163 ± 0.00037

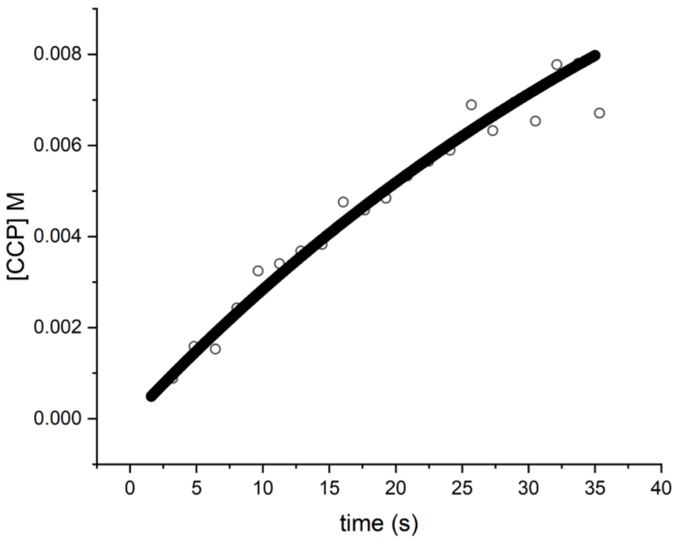

Figure S7. Formation of **2** from complex **6a** at –10 °C (Run 1)

Table S18. Data for the formation of **2** from complex **6a** at –10 °C (Run 1)

Time (s)	Integral IS (−140 ppm)	Integral CCP (−116 ppm)	[CCP] (M)
1.61	389922	21333.7	0.000829
3.21	370813	29907.4	0.001222
4.82	376464	47840.6	0.001926
6.43	371606	45821.6	0.001868
8.03	364076	66573.2	0.002771
9.64	352746	83426.6	0.003584
11.25	350662	86645.1	0.003744
12.85	345790	91757	0.004021
14.46	375108	103234	0.00417
16.07	335458	112791	0.005095
17.67	363136	117919	0.00492
19.28	354423	121200	0.005182
20.89	347897	130117	0.005667
22.49	344466	136154	0.005989
24.10	340448	140014	0.006232
25.71	314379	150057	0.007232
27.31	357270	157182	0.006666
28.92	339402	163181	0.007285
30.53	354267	160756	0.006876
32.13	327866	175532	0.008112

33.74	345397	185540	0.00814
35.35	372963	173543	0.007051

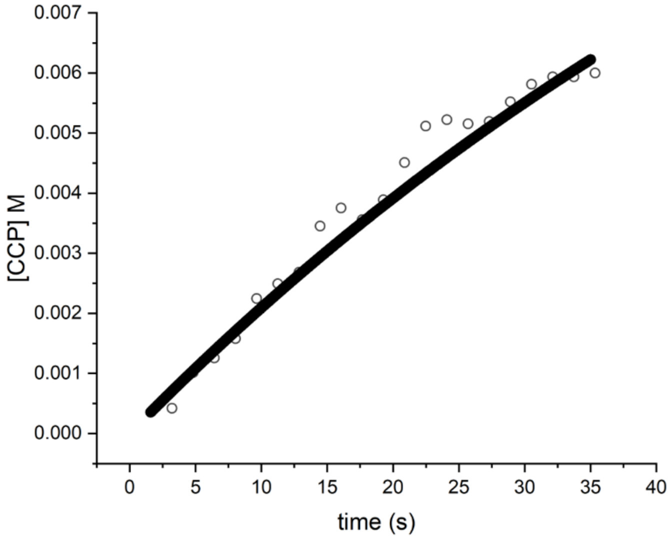

Figure 8. Formation of **2** from complex **6a** at –10 °C (Run 2)

Table S19. Data for the formation of **2** from complex **6a** at –10 °C (Run 2)

**Time (s)**	**Integral IS (−140 ppm)**	**Integral CCP (−116 ppm)**	**[CCP] (M)**
1.61	385142	5406.29	0.000217
3.21	371458	24006.9	0.000999
4.82	370613	38235.7	0.001595
6.43	363836	43236.2	0.001837
8.03	349481	48867.2	0.002161
9.64	353318	64560.4	0.002825
11.25	341523	67837.9	0.00307
12.85	332536	70217.3	0.003264
14.46	356867	93098.5	0.004033
16.07	337158	94539.3	0.004334
17.67	329818	88287.8	0.004138
19.28	325997	94272.9	0.00447
20.89	329032	108279	0.005087

22.49	328508	121054	0.005696
24.10	323895	121545	0.005801
25.71	340113	126215	0.005736
27.31	333004	124396	0.005774
28.92	337377	133161	0.006101
30.53	342426	141559	0.00639
32.13	344911	145348	0.006514
33.74	349025	147093	0.006514
35.35	342860	145919	0.006579

Experiment 5: Kinetic Study of the (dppe)Pd[(4-F)Ph]Br **(7a)**

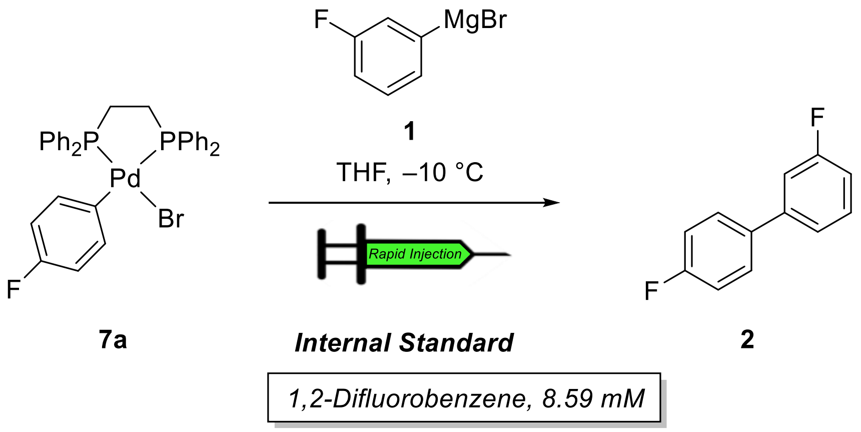

A 5-mL volumetic flask was charged with 1,2-Difluorobenzene (5.0 µL, 50.7 µmol) followed by dissolving with THF to the 5-mL mark generating a 0.01 M of stock solution. An oven-dried (150 °C), 5 mm, NMR tube was taken into the dry box and charged with **7a** (6.8 mg, 10 µmol) and 500 µL of the freshly prepared solution. The tube was capped with a septum. An oven-dried (60 °C) rapid injection barrel was taken into the glove box and charged with 3-Fluorophenylmagnesium bromide solution (500 µL, 1.33 M in THF). The glass capillary of the barrel was capped with a septum. The sample and the barrel were removed from the glove box and the sample was placed into the NMR probe set to –10 °C with the cap off. Then 3-Fluorophenylmagnesium bromide **1** (120 µmol) in THF (90 µL) was injected (RI-NMR).

Using the fluorine channel to collect a spectrum every 1.6 s the progress of the reaction was monitored by the formation of cross-coupling product in comparison with the internal reference 1,2-Difluorobenzene. The first order formation profile was fitted with the Curve Fitter Toolbox in Matlab using Equation 1.

Table S20. Summary of fits for the formation of **2**

Run	R^2 of fit	k ($10^{-2}s^{-1}$) (form CCP)	$[OAC]_o$ (mM)
1	0.9826	5.42 ± 0.60	0.0171 ± 0.00068
2	0.9890	4.76 ± 0.39	0.0174 ± 0.00050

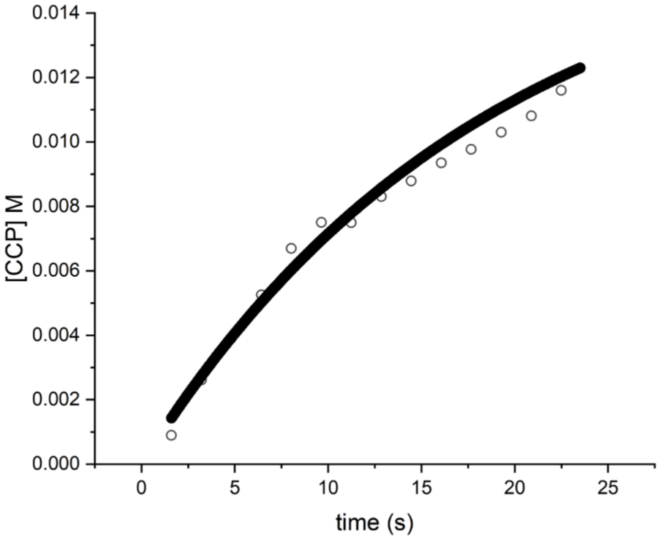

Figure S9. Formation of **2** from complex **7a** at –10 °C (Run 1)

Table S21. Data for the formation of **2** from complex **7a** at –10 °C (Run 1)

Time (s)	Integral IS (−140 ppm)	Integral CCP (−116 ppm)	[CCP] (M)
1.61	380550	18090.4	0.000735
3.21	365383	57916.8	0.00245
4.82	368094	89111.8	0.003742
6.43	353731	116580	0.005094
8.03	337705	142854	0.006539
9.64	343327	163039	0.007341
11.25	348934	165580	0.007335
12.85	336425	177315	0.008147
14.46	342872	191394	0.008629
16.07	343844	204422	0.00919
17.67	342785	213093	0.009609
19.28	334365	219412	0.010143
20.89	332774	229161	0.010645
22.49	321624	237963	0.011437

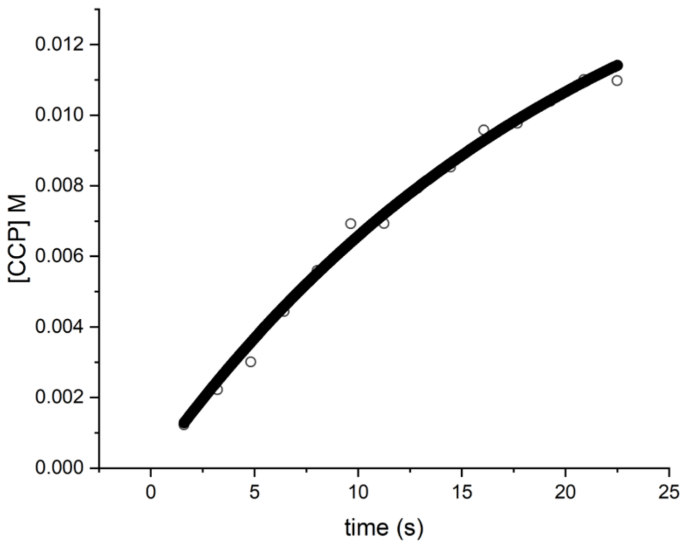

Figure S10. Formation of **2** from complex **7a** at –10 °C (Run 2)

Table S22. Data for the formation of 2 from complex **7a** at –10 °C (Run 2)

Time (s)	Integral IS (−140 ppm)	Integral CCP (−116 ppm)	[CCP] (M)
1.61	362064	17819.3	0.000776
3.21	368659	41440.8	0.001772
4.82	339735	55146	0.002559
6.43	354799	89765.3	0.003988
8.03	338680	110667	0.005151
9.64	323642	132979	0.006477
11.25	339753	139644	0.006479
12.85	323956	153674	0.007477
14.46	335123	171701	0.008076
16.07	331452	192045	0.009133
17.67	315668	186634	0.009319
19.28	310453	195901	0.009947
20.89	327124	219146	0.01056
22.49	312859	209021	0.010531

Experiment 6: Kinetic Study of the (XPhos)Pd[(4-F)Ph]Br **(8a)**

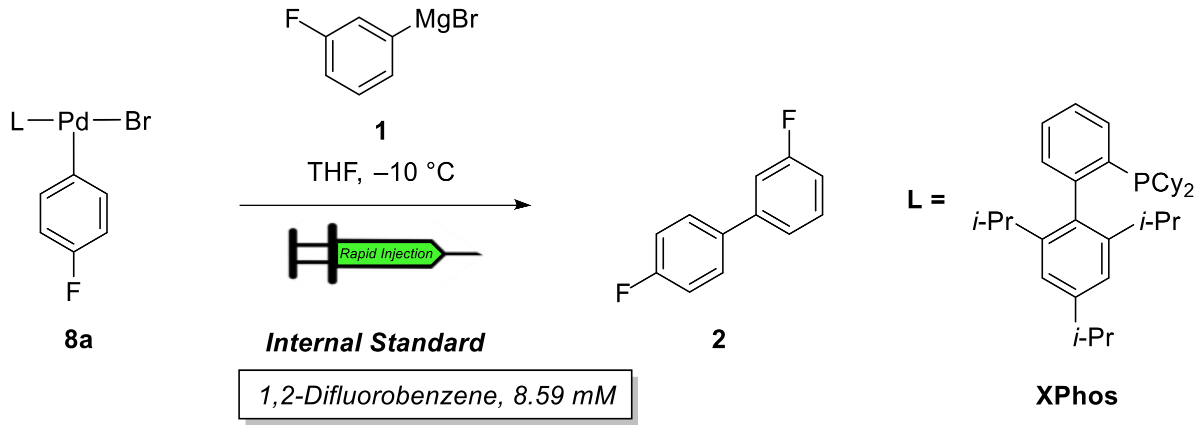

A 5-mL volumetic flask was charged with 1,2-Difluorobenzene (5.0 µL, 50.7 µmol) followed by dissolving with THF to the 5-mL mark generating a 0.01 M of stock solution. An oven-dried (150 °C), 5 mm, NMR tube was taken into the dry box and charged with **8a** (7.6 mg, 10 µmol) and 500 µL of the freshly prepared solution. The tube was capped with a septum. An oven-dried (60 °C) rapid injection barrel was taken into the glove box and charged with 3-Fluorophenylmagnesium bromide solution (500 µL, 1.33 M in THF). The glass capillary of the barrel was capped with a septum. The sample and the barrel were removed from the glove box and the sample was placed into the NMR probe set to –10 °C with the cap off. Then 3-Fluorophenylmagnesium bromide **1** (120 µmol) in THF (90 µL) was injected (RI-NMR).

Using the fluorine channel to collect a spectrum every 1.6 s the progress of the reaction was monitored by the formation of cross-coupling product in comparison with the internal reference 1,2-Difluorobenzene. The first order formation profile was fitted with the Curve Fitter Toolbox in Matlab using Equation 1.

Table S23. Summary of fits for the formation of **2**

**Run**	**R^2 of fit**	***k*** **($10^{-2}s^{-1}$) (form CCP)**	**$[OAC]_o$ (mM)**
1	0.9948	6.88 ± 0.92	0.0168 ± 0.0093
2	0.9898	5.56 ± 0.79	0.0166 ± 0.0006

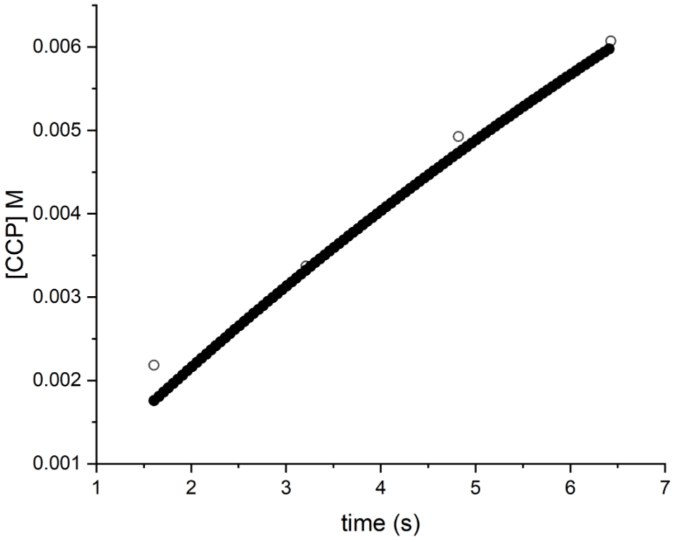

Figure S11. Formation of **2** from complex **8a** at –10 °C (Run 1)

Table S24. Data for the formation of **2** from complex **8a** at –10 °C (Run 1)

Time (s)	Integral IS (−140 ppm)	Integral CCP (−116 ppm)	[CCP] (M)
1.61	214283	31481.2	0.002181
3.21	207161	47023.4	0.00337
4.82	199050	66012.5	0.004924
6.43	196057	80175.1	0.006072

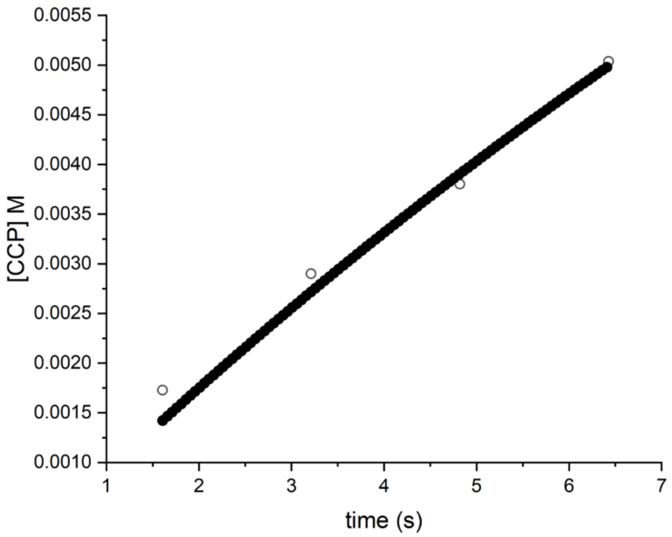

Figure S12. Formation of **2** from complex **8a** at –10 °C (Run 2)

Table S25. Data for the formation of **2** from complex **8a** at –10 °C (Run 2)

**Time (s)**	**Integral IS (−140 ppm)**	**Integral CCP (−116 ppm)**	**[CCP] (M)**
1.61	299705	40943.3	0.002028
3.21	289157	62314.7	0.0032
4.82	264309	72991	0.0041
6.43	251197	90312.1	0.005338

Experiment 7: Kinetic Study of the (PhXPhos)Pd[(4-F)Ph]Br **(9a)**

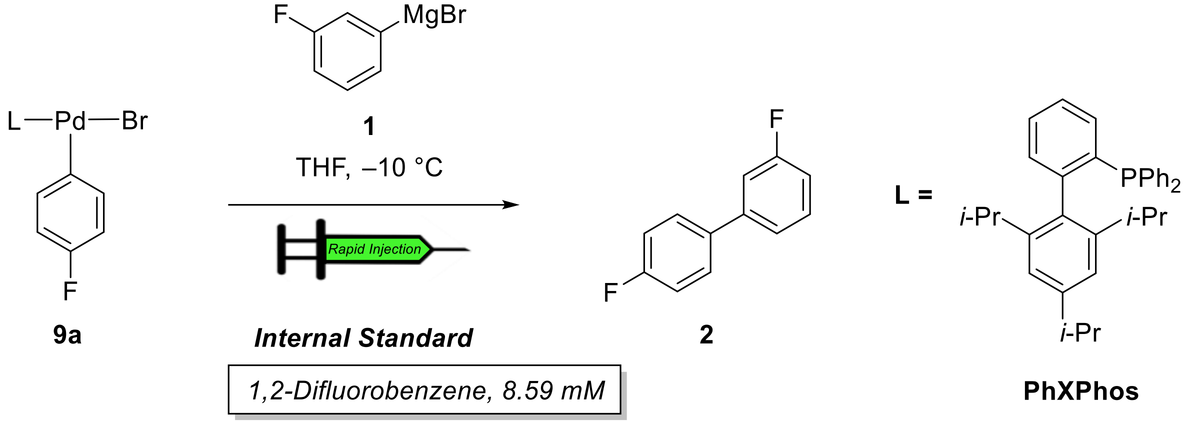

A 5-mL volumetic flask was charged with 1,2-Difluorobenzene (5.0 µL, 50.7 µmol) followed by dissolving with THF to the 5-mL mark generating a 0.01 M of stock solution. An oven-dried (150 °C), 5 mm, NMR tube was taken into the dry box and charged with **9a** (7.46 mg, 10 µmol) and 500 µL of the freshly prepared solution. The tube was capped with a septum. An oven-dried (60 °C) rapid injection barrel was taken into the glove box and charged with 3-Fluorophenylmagnesium bromide solution (500 µL, 1.33 M in THF). The glass capillary of the barrel was capped with a septum. The sample and the barrel were removed from the glove box and the sample was placed into the NMR probe set to –10 °C with the cap off. Then 3-Fluorophenylmagnesium bromide **1** (120 µmol) in THF (90 µL) was injected (RI-NMR).

Using the fluorine channel to collect a spectrum every 1.6 s the progress of the reaction was monitored by the formation of cross-coupling product in comparison with the internal reference 1,2-Difluorobenzene. The first order formation profile was fitted with the Curve Fitter Toolbox in Matlab using Equation 1.

Table S26. Summary of fits for the formation of **2**

**Run**	**R^2 of fit**	***k*** **($10^{-2}s^{-1}$) (form CCP)**	**$[OAC]_o$ (mM)**
1	0.9881	8.59 ± 1.14	0.0174 ± 0.00085
2	0.9823	6.87 ± 1.10	0.0172 ± 0.00087

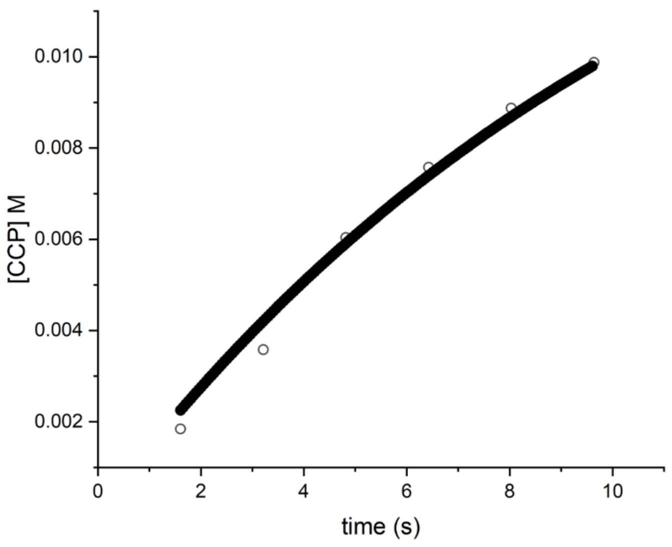

Figure S13. Formation of **2** from complex **9a** at –10 °C (Run 1)

Table S27. Data for the formation of **2** from complex **9a** at –10 °C (Run 1)

Time (s)	Integral IS (−140 ppm)	Integral CCP (−116 ppm)	[CCP] (M)
1.61	333518	29158.5	0.001325
3.21	385452	77780.9	0.003058
4.82	352744	128391	0.005515
6.43	341304	158984	0.007058
8.03	347082	191386	0.008355
9.64	331002	204390	0.009357

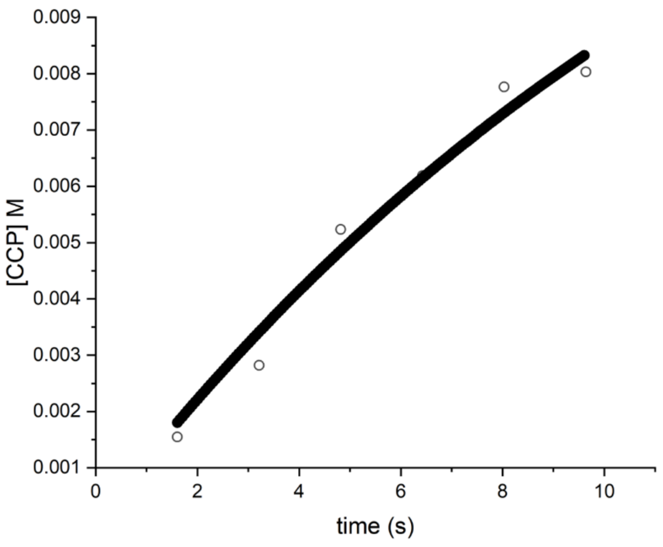

Figure S14. Formation of **2** from complex **9a** at –10 °C (Run 2)

Table S28. Data for the formation of **2** from complex **9a** at –10 °C (Run 2)

Time (s)	Integral IS (−140 ppm)	Integral CCP (−116 ppm)	[CCP] (M)
1.61	382497	30762.1	0.001219
3.21	376163	61746.7	0.002487
4.82	377758	122254	0.004904
6.43	348218	134641	0.005859
8.03	336024	164918	0.007437
9.64	349250	177471	0.0077

Experiment 8: Kinetic Study of the (dppp-dimethyl)Pd[(4-F)Ph]Br **(10a)**

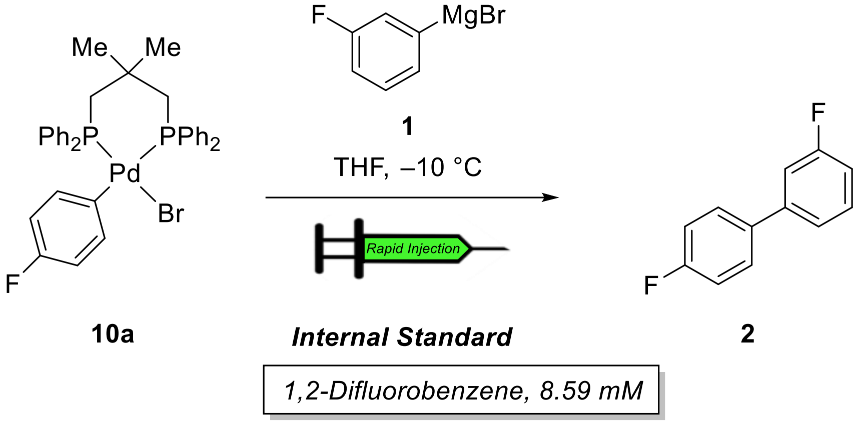

A 5-mL volumetic flask was charged with 1,2-Difluorobenzene (5.0 µL, 50.7 µmol) followed by dissolving with THF to the 5-mL mark generating a 0.01 M of stock solution. An oven-dried (150 °C), 5 mm, NMR tube was taken into the dry box and charged with **10a** (7.22 mg, 10 µmol) and 500 µL of the freshly prepared solution. The tube was capped with a septum. An oven-dried (60 °C) rapid injection barrel was taken into the glove box and charged with 3-Fluorophenylmagnesium bromide solution (500 µL, 1.33 M in THF). The glass capillary of the barrel was capped with a septum. The sample and the barrel were removed from the glove box and the sample was placed into the NMR probe set to –10 °C with the cap off. Then 3-Fluorophenylmagnesium bromide **1** (120 µmol) in THF (90 µL) was injected (RI-NMR).

Using the fluorine channel to collect a spectrum every 1.6 s the progress of the reaction was monitored by the formation of cross-coupling product in comparison with the internal reference 1,2-Difluorobenzene. The first order formation profile was fitted with the Curve Fitter Toolbox in Matlab using Equation 1.

Table S29. Summary of fits for the formation of **2**

Run	R^2 of fit	k ($10^{-2}s^{-1}$) (form CCP)	$[OAC]_o$ (M)
1	0.9754	8.59 ± 1.95	0.0172 ± 0.00127
2	0.9913	7.77 ± 0.76	0.0170 ± 0.00064

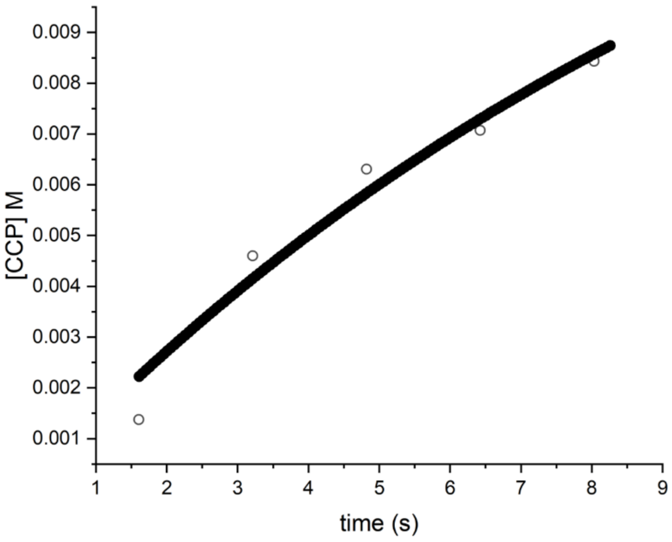

Figure S15. Formation of **2** from complex **10a** at –10 °C (Run 1)

Table S30. Data for the formation of **2** from complex **10a** at –10 °C (Run 1)

**Time (s)**	**Integral IS (−140 ppm)**	**Integral CCP (−116 ppm)**	**[CCP] (M)**
1.61	262567	17714.1	0.001084
3.21	254703	68296.2	0.004308
4.82	252628	94572.8	0.006015
6.43	254508	107426	0.006782
8.03	254536	128941	0.00814

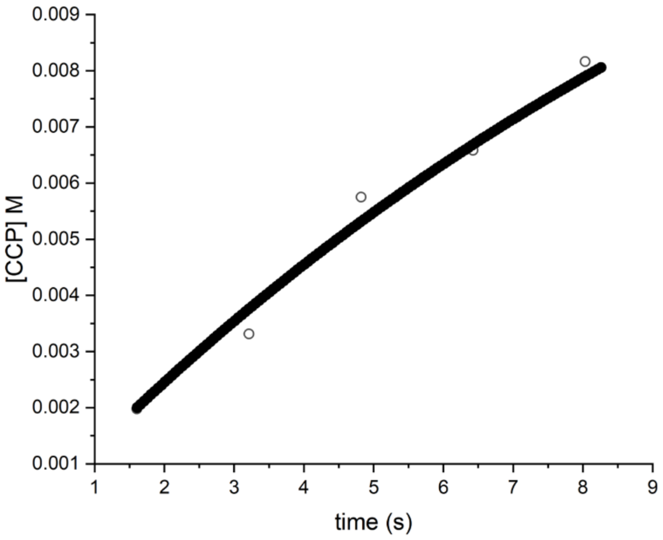

Figure S16. Formation of **2** from complex **10a** at –10 °C (Run 2)

Table S31. Data for the formation of **2** from complex **10a** at –10 °C (Run 2)

**Time (s)**	**Integral IS (−140 ppm)**	**Integral CCP (−116 ppm)**	**[CCP] (M)**
1.61	338150	40235.3	0.001876
3.21	322909	65808.4	0.003212
4.82	314655	112735	0.005647
6.43	296450	121953	0.006484
8.03	309354	158261	0.008064
9.64	309605	165077	0.008404

Experiment 9: Kinetic Study of the (RuPhos)Pd[(4-F)Ph]Br **(11a)**

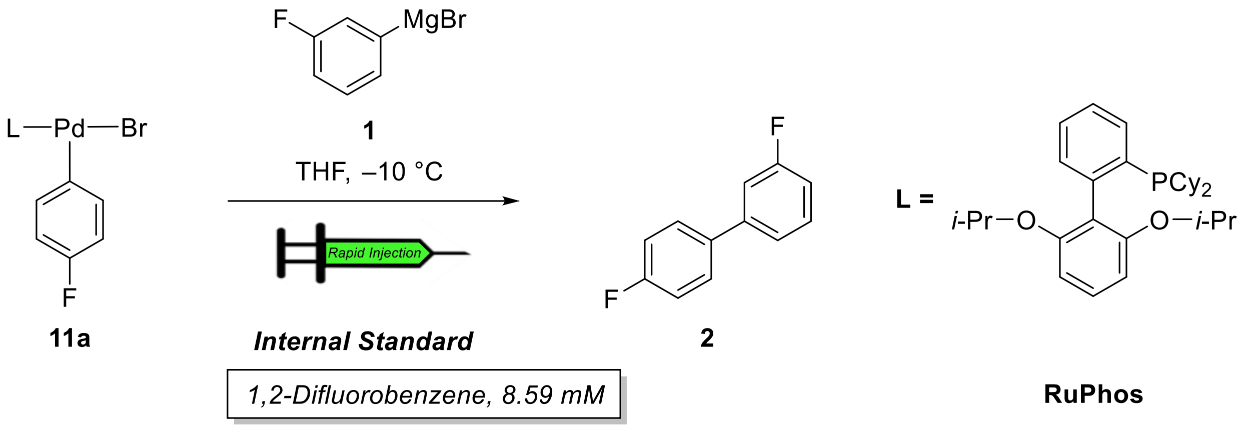

A 5-mL volumetic flask was charged with 1,2-Difluorobenzene (5.0 µL, 50.7 µmol) followed by dissolving with THF to the 5-mL mark generating a 0.01 M of stock solution. An oven-dried (150 °C), 5 mm, NMR tube was taken into the dry box and charged with **11a** (7.5 mg, 10 µmol) and 500 µL of the freshly prepared solution. The tube was capped with a septum. An oven-dried (60 °C) rapid injection barrel was taken into the glove box and charged with 3-Fluorophenylmagnesium bromide solution (500 µL, 1.33 M in THF). The glass capillary of the barrel was capped with a septum. The sample and the barrel were removed from the glove box and the sample was placed into the NMR probe set to –10 °C with the cap off. Then 3-Fluorophenylmagnesium bromide **1** (120 µmol) in THF (90 µL) was injected (RI-NMR).

Using the fluorine channel to collect a spectrum every 1.6 s the progress of the reaction was monitored by the formation of cross-coupling product in comparison with the internal reference 1,2-Difluorobenzene. The first order formation profile was fitted with the Curve Fitter Toolbox in Matlab using Equation 1.

Table S32. Summary of fits for the formation of **2**

Run	R^2 of fit	k ($10^{-2}s^{-1}$) (form CCP)	$[OAC]_o$ (M)
1	0.9625	11.39 ± 3.31	0.0168 ± 0.00192
2	0.9794	12.18 ± 2.59	0.0165 ± 0.00143

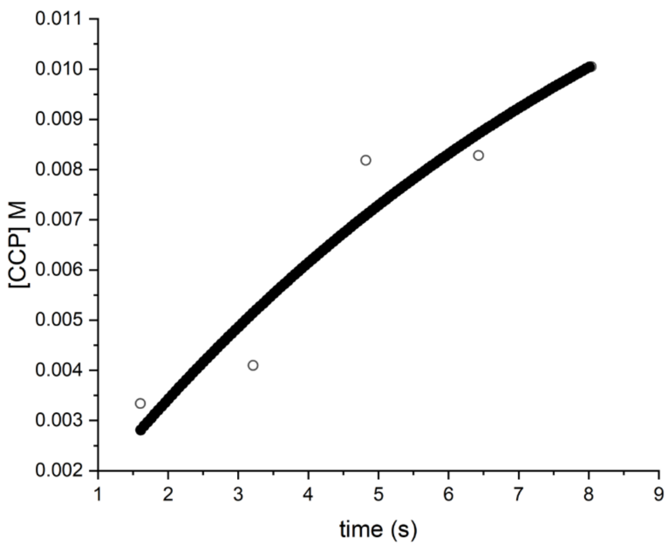

Figure S17. Formation of **2** from complex **11a** at –10 °C (Run 1)

Table S33. Data for the formation of **2** from complex **11a** at –10 °C (Run 1)

**Time (s)**	**Integral IS (−140 ppm)**	**Integral CCP (−116 ppm)**	**[CCP] (M)**
1.61	235578	54837.7	0.003456
3.21	310495	88255.9	0.00422
4.82	312672	174853	0.008303
6.43	301275	170416	0.008398
8.04	291609	199739	0.01017

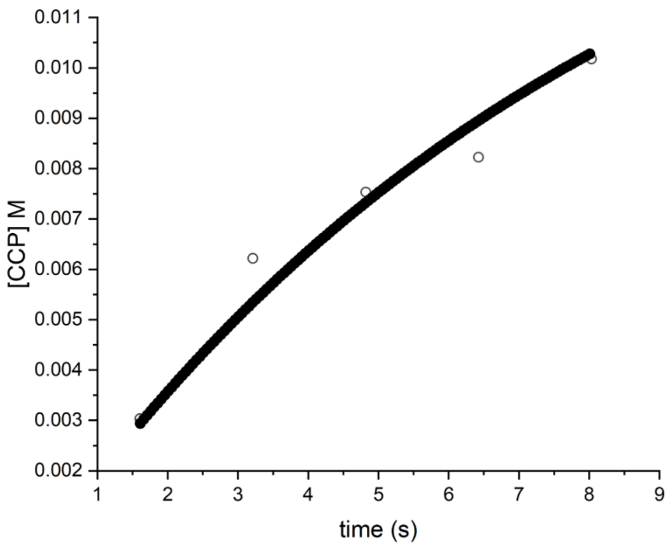

Figure S18. Formation of **2** from complex **11a** at –10 °C (Run 2)

Table S34. Data for the formation of **2** from complex **11a** at –10 °C (Run 2)

**Time (s)**	**Integral IS (−140 ppm)**	**Integral CCP (−116 ppm)**	**[CCP] (M)**
1.61	395576	91777.8	0.003445
3.21	384656	171657	0.006626
4.82	371872	198867	0.00794
6.43	383068	222773	0.008635
8.03	354824	253039	0.010588

Experiment 10: Kinetic Study of the (dppp)Pd[(4-F)Ph]Br **(12a)**

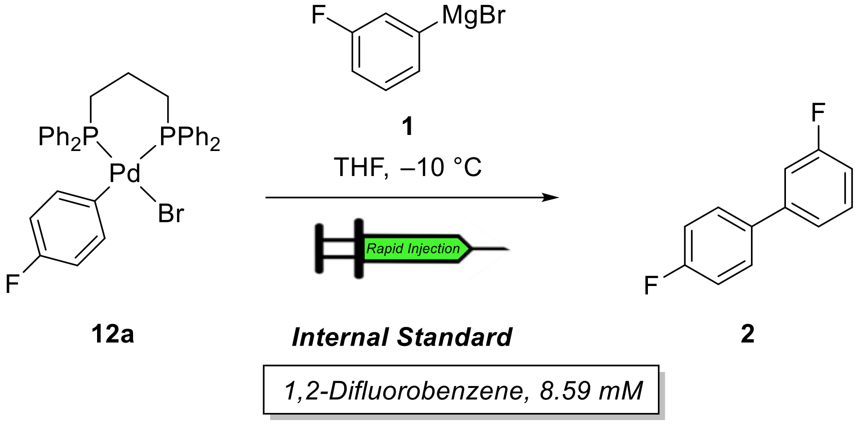

A 5-mL volumetic flask was charged with 1,2-Difluorobenzene (5.0 µL, 50.7 µmol) followed by dissolving with THF to the 5-mL mark generating a 0.01 M of stock solution. An oven-dried (150 °C), 5 mm, NMR tube was taken into the dry box and charged with **12a** (6.94 mg, 10 µmol) and 500 µL of the freshly prepared solution. The tube was capped with a septum. An oven-dried (60 °C) rapid injection barrel was taken into the glove box and charged with 3-Fluorophenylmagnesium bromide solution (500 µL, 1.33 M in THF). The glass capillary of the barrel was capped with a septum. The sample and the barrel were removed from the glove box and the sample was placed into the NMR probe set to –10 °C with the cap off. Then 3-Fluorophenylmagnesium bromide **1** (120 µmol) in THF (90 µL) was injected (RI-NMR).

Using the fluorine channel to collect a spectrum every 1.6 s the progress of the reaction was monitored by the formation of cross-coupling product in comparison with the internal reference 1,2-Difluorobenzene. The first order formation profile was fitted with the Curve Fitter Toolbox in Matlab using Equation 1.

Table S35. Summary of fits for the formation of **2**

**Run**	**R^2 of fit**	***k*** **($10^{-2}s^{-1}$) (form CCP)**	**$[OAC]_o$ (M)**
1	0.9873	15.04 ± 3.35	0.0172 ± 0.00154
2	0.9742	14.48 ± 7.61	0.0174 ± 0.00297

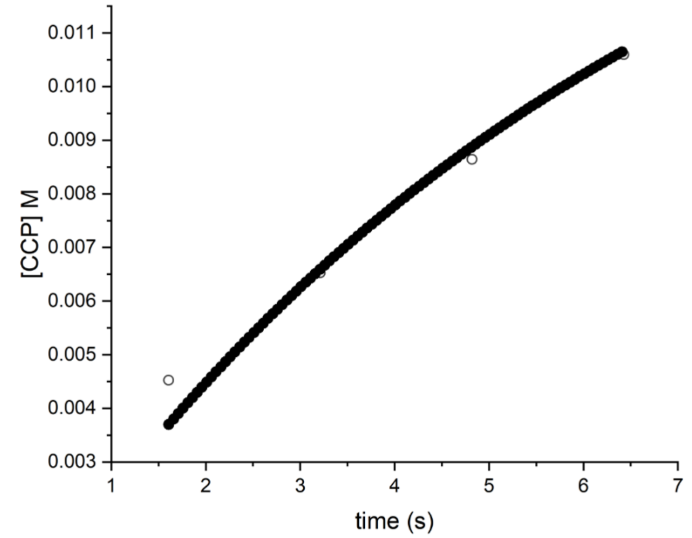

Figure S19. Formation of **2** from complex **12a** at –10 °C (Run 1)

Table S36. Data for the formation of **2** from complex **12a** at –10 °C (Run 1)

**Time (s)**	**Integral IS (−140 ppm)**	**Integral CCP (−116 ppm)**	**[CCP] (M)**
1.61	113524	35408.5	0.004916
3.21	270189	118508	0.006914
4.82	265720	152247	0.009031
6.43	244848	170632	0.010985

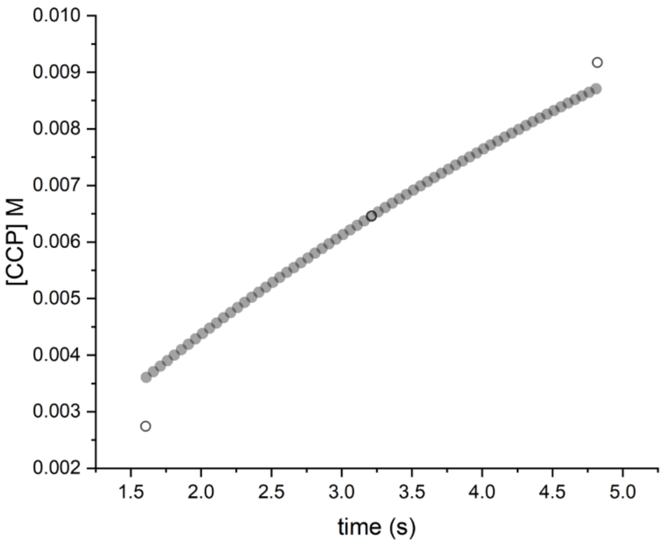

Figure S20. Formation of **2** from complex **12a** at –10 °C (Run 2)

Table S37. Data for the formation of **2** from complex **12a** at –10 °C (Run 2)

Time (s)	Integral IS (−140 ppm)	Integral CCP (−116 ppm)	[CCP] (M)
1.61	341072	49373	0.002282
3.21	333101	126787	0.006
4.82	334770	185060	0.008714

Experiment 11: Kinetic Study of the (DavePhos)Pd[(4-F)Ph]Br **(13a)**

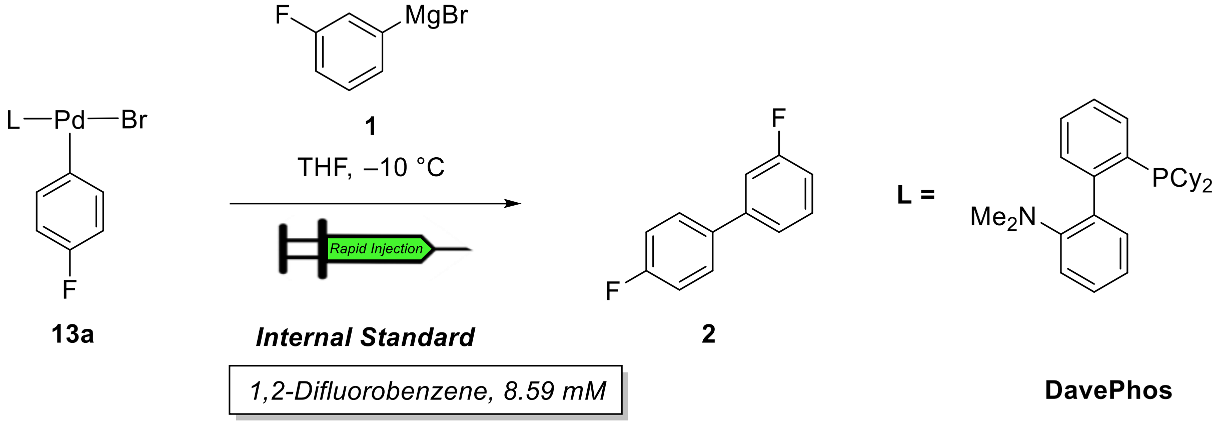

A 5-mL volumetic flask was charged with 1,2-Difluorobenzene (5.0 µL, 50.7 µmol) followed by dissolving with THF to the 5-mL mark generating a 0.01 M of stock solution. An oven-dried (150 °C), 5 mm, NMR tube was taken into the dry box and charged with **13a** (6.75 mg, 10 µmol) and 500 µL of the freshly prepared solution. The tube was capped with a septum. An oven-dried (60 °C) rapid injection barrel was taken into the glove box and charged with 3-Fluorophenylmagnesium bromide solution (500 µL, 1.33 M in THF). The glass capillary of the barrel was capped with a septum. The sample and the barrel were removed from the glove box and the sample was placed into the NMR probe set to –10 °C with the cap off. Then 3-Fluorophenylmagnesium bromide **1** (120 µmol) in THF (90 µL) was injected (RI-NMR).

Using the fluorine channel to collect a spectrum every 1.6 s the progress of the reaction was monitored by the formation of cross-coupling product in comparison with the internal reference 1,2-Difluorobenzene. The first order formation profile was fitted with the Curve Fitter Toolbox in Matlab using Equation 1.

Table S38. Summary of fits for the formation of **2**

Run	R^2 of fit	k ($10^{-2}s^{-1}$) (form CCP)	$[OAC]_o$ (M)
1	0.9696	34.28 ± 15.54	0.0176 ± 0.00409
2	0.9911	30.52 ± 6.92	0.0172 ± 0.00199

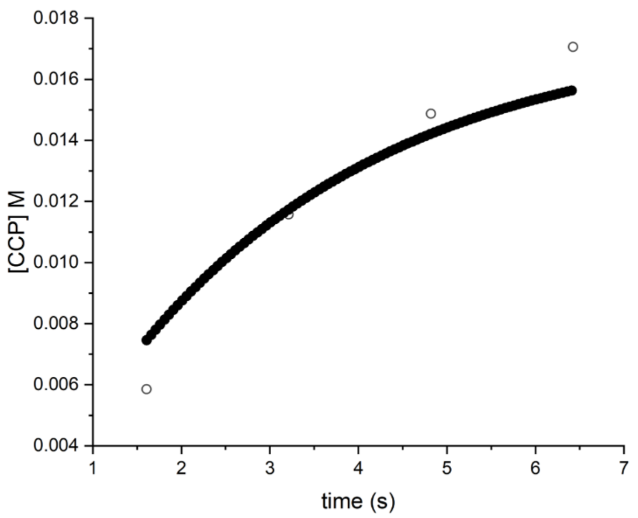

Figure S21. Formation of **2** from complex **13a** at –10 °C (Run 1)

Table S39. Data for the formation of **2** at –10 °C (Run 1)

Time (s)	Integral IS (−140 ppm)	Integral CCP (−116 ppm)	[CCP] (M)
1.61	390549	123712	0.005176
3.21	370551	247091	0.010895
4.82	378632	328807	0.014189
6.43	352909	353805	0.01638

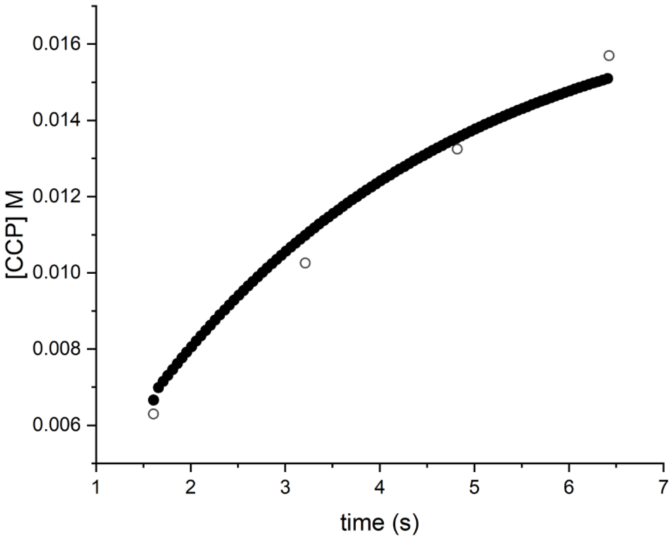

Figure S22. Formation of **2** from complex **13a** at –10 °C (Run 2)

Table S40. Data for the formation of **2** from complex **13a** at –10 °C (Run 2)

**Time (s)**	**Integral IS (−140 ppm)**	**Integral CCP (−116 ppm)**	**[CCP] (M)**
1.61	317460	117478	0.006046
3.21	380973	233266	0.010004
4.82	379649	301953	0.012995
6.43	343534	324733	0.015445

Experiment 12: Kinetic Study of the (CPhos)Pd[(4-F)Ph]Br **(14a)**

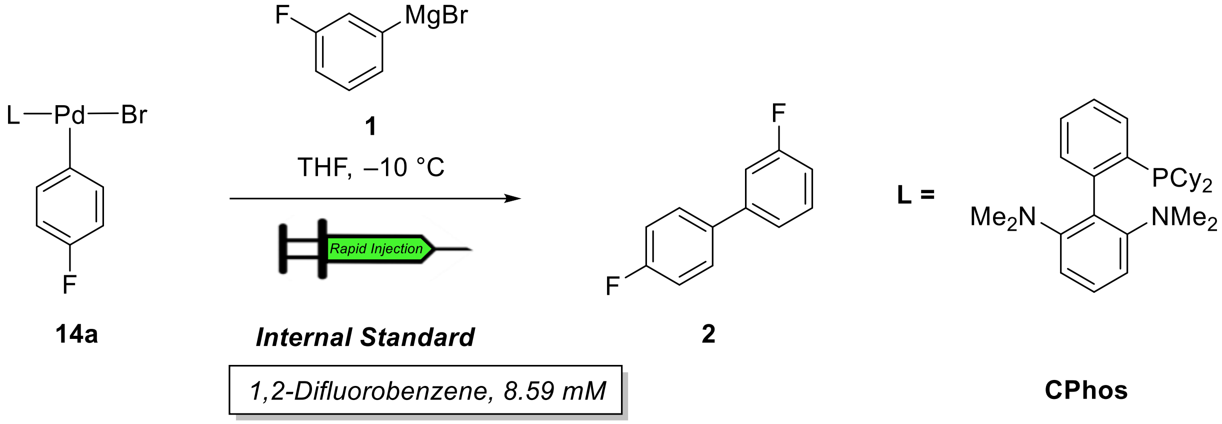

A 5-mL volumetic flask was charged with 1,2-Difluorobenzene (5.0 μL, 50.7 μmol) followed by dissolving with THF to the 5-mL mark generating a 0.01 M of stock solution. An oven-dried (150 °C), 5 mm, NMR tube was taken into the dry box and charged with **14a** (7.18 mg, 10 μmol) and 500 μL of the freshly prepared solution. The tube was capped with a septum. An oven-dried (60 °C) rapid injection barrel was taken into the glove box and charged with 3-Fluorophenylmagnesium bromide solution (500 μL, 1.33 M in THF). The glass capillary of the barrel was capped with a septum. The sample and the barrel were removed from the glove box and the sample was placed into the NMR probe set to –10 °C with the cap off. Then 3-Fluorophenylmagnesium bromide **1** (120 μmol) in THF (90 μL) was injected (RI-NMR).

Using the fluorine channel to collect a spectrum every 1.6 s the progress of the reaction was monitored by the formation of cross-coupling product in comparison with the internal reference 1,2-Difluorobenzene. The first order formation profile was fitted with the Curve Fitter Toolbox in Matlab using Equation 1.

Table S41. Summary of fits for the formation of **2**

Run	R^2 of fit	k ($10^{-2}s^{-1}$) (form CCP)	$[OAC]_o$ (M)
1	0.9905	31.61 ± 5.13	0.0172 ± 0.00154
2	0.9975	44.52 ± 6.00	0.0168 ± 0.00113

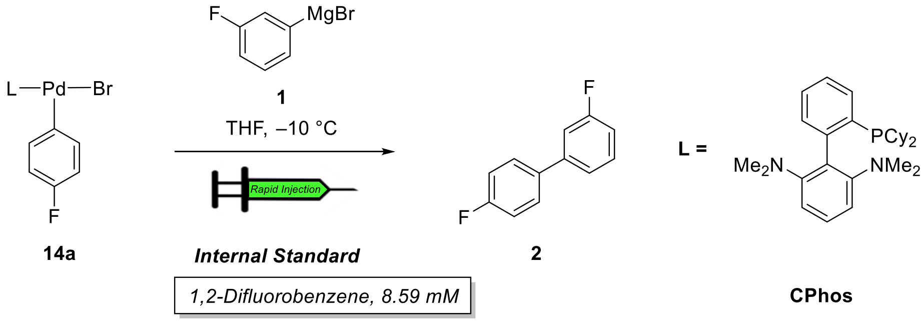

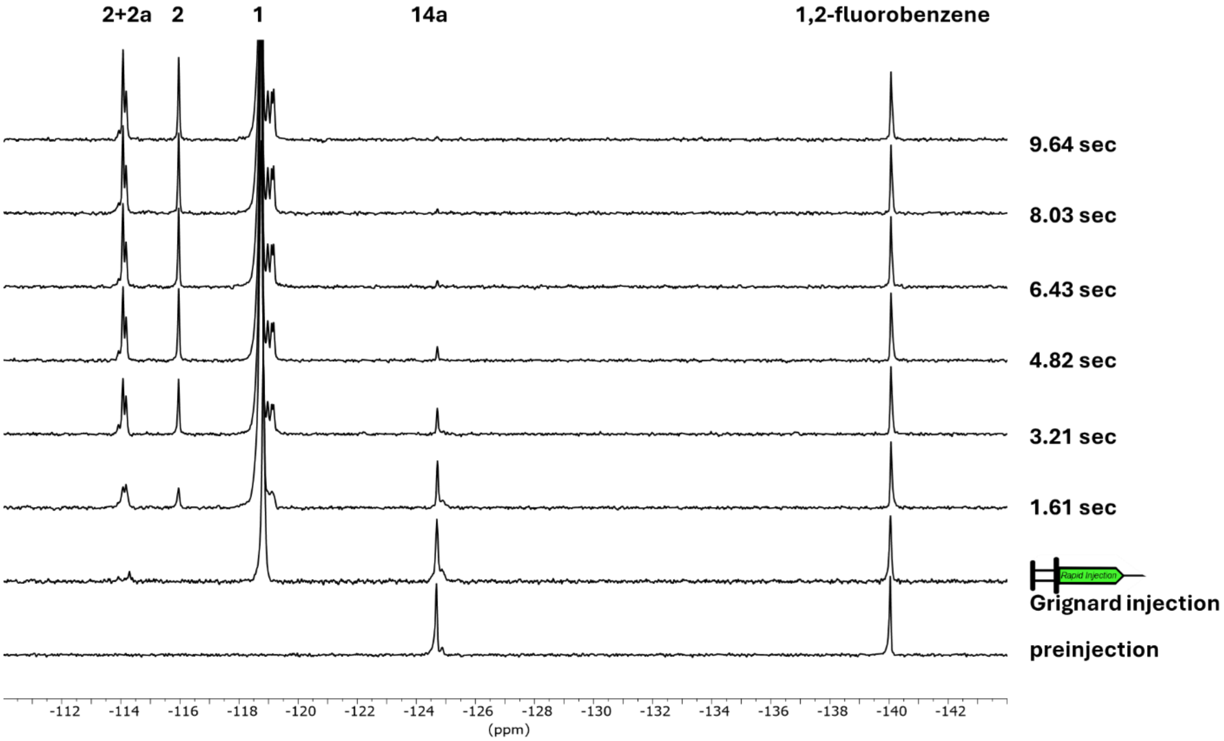

Figure S23. ^{19}F NMR for kinetic array: Formation of **2** from complex **14a** at –10 °C

Corresponding fluorine chemical shifts

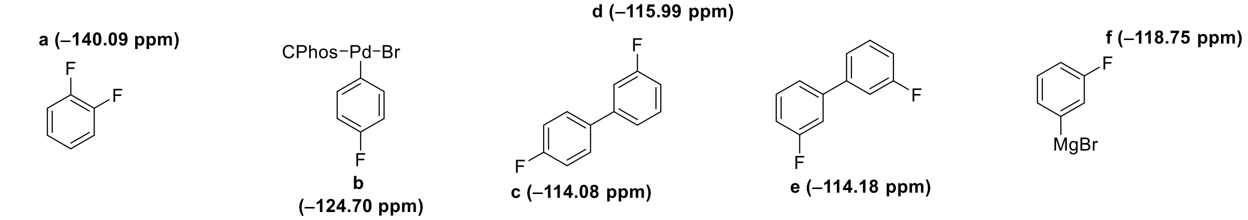

1,2-fluorobenzene (internal standard) | **14a** | **2** | **2a** | **1**

homocoupling product
(From Grignard preparation)

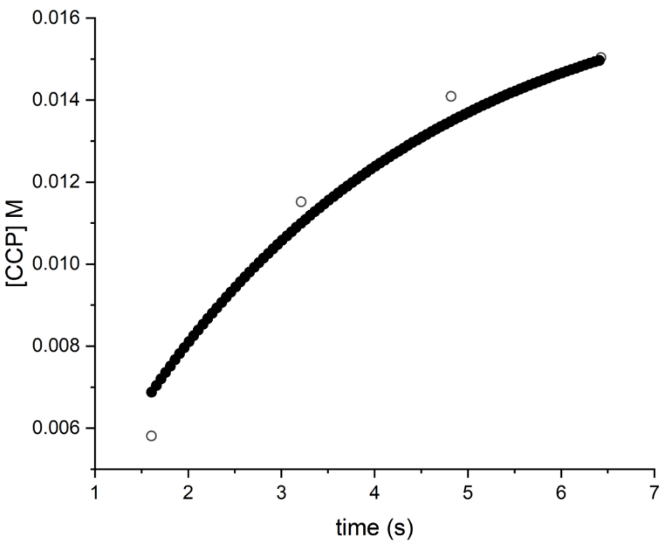

Figure S24. Formation of **2** from complex **14a** at –10 °C (Run 1)

Table S42. Data for the formation of **2** from complex **14a** at –10 °C (Run 1)

**Time (s)**	**Integral IS (−140 ppm)**	**Integral CCP (−116 ppm)**	**[CCP] (M)**
1.61	349860	123782	0.005469
3.21	349010	252420	0.01118
4.82	359509	319746	0.013748
6.43	342632	325729	0.014695

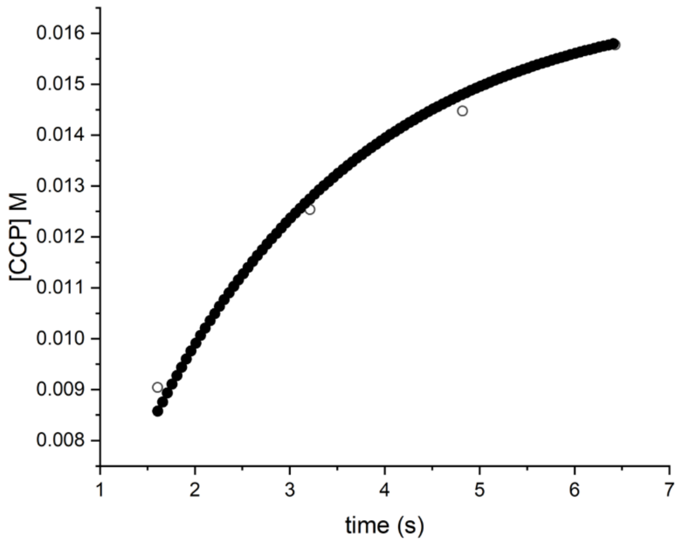

Figure S25. Formation of **2** from complex **14a** at –10 °C (Run 2)

Table S43. Data for the formation of **2** from complex **14a** at –10 °C (Run 2)

**Time (s)**	**Integral IS (−140 ppm)**	**Integral CCP (−116 ppm)**	**[CCP] (M)**
1.61	316496	195708	0.009181
3.21	374372	319624	0.012676
4.82	375687	369798	0.014615
6.43	334113	358037	0.015911

Experiment 13: Kinetic Study of the *trans*-[(4-F-C_6H_4)Pd(*i*-Pr_3P)$_2$Br] **(15a)**

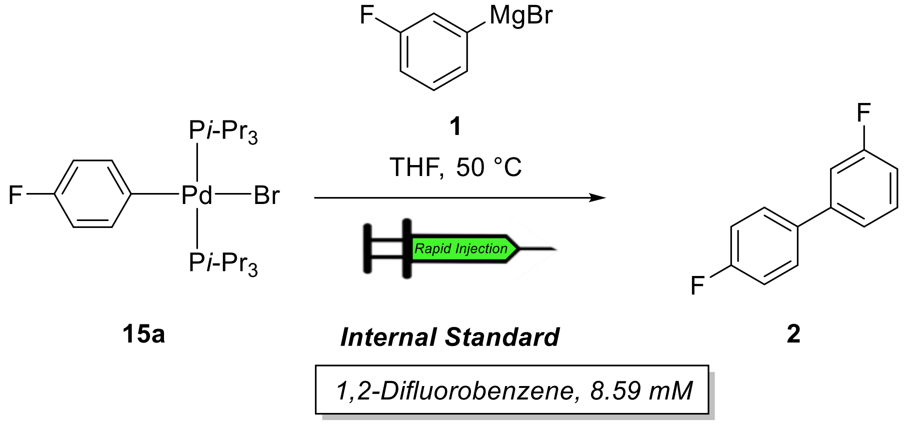

A 5-mL volumetic flask was charged with 1,2-Difluorobenzene (5.0 µL, 50.7 µmol) followed by dissolving with THF to the 5-mL mark generating a 0.01 M of stock solution. An oven-dried (150 °C), 5 mm, NMR tube was taken into the dry box and charged with **15a** (6.02 mg, 10 µmol) and 500 µL of the freshly prepared solution. The tube was capped with a septum. An oven-dried (60 °C) rapid injection barrel was taken into the glove box and charged with 3-Fluorophenylmagnesium bromide solution (500 µL, 1.33 M in THF). The glass capillary of the barrel was capped with a septum. The sample and the barrel were removed from the glove box and the sample was placed into the NMR probe set to –10 °C with the cap off. Then 3-Fluorophenylmagnesium bromide **1** (120 µmol) in THF (90 µL) was injected (RI-NMR).

Using the fluorine channel to collect a spectrum every 107 s the progress of the reaction was monitored by the formation of cross-coupling product in comparison with the internal reference 1,2-Difluorobenzene. The first order formation profile was fitted with the Curve Fitter Toolbox in Matlab using Equation 1.

Table S44. Summary of fits for the formation of **2**

Run	R^2 of fit	k ($10^{-2}s^{-1}$) (form CCP)	[OAC]$_o$ (M)
1	0.9898	0.005751 ± 0.00012	0.0170 ± 0.00011
2	0.9804	0.005457 ± 0.00016	0.0167 ± 0.00014

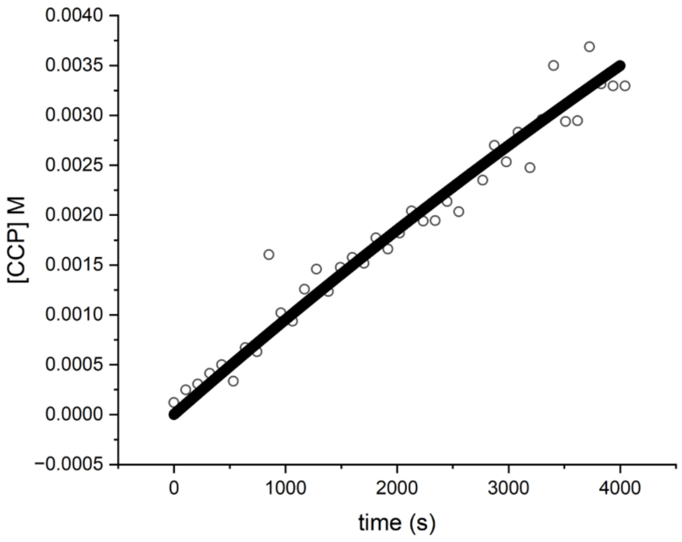

Figure S26. Formation of **2** from complex **15a** at –10 °C (Run 1)

Table S45. Data for the formation of **2** from complex **15a** at –10 °C (Run 1)

Time (s)	Integral IS (−140 ppm)	Integral CCP (−116 ppm)	[CCP] (M)
106	4306900	34276.9	0.000128
213	4348650	50677.6	0.000187
321	4289780	78587.3	0.000294
427	4302710	102275	0.000381
533	4344120	58383.8	0.000215
639	4272290	146981	0.000552
745	4291370	136724	0.000511
852	4266640	394654	0.001483
958	4308620	242215	0.000901
1064	4344950	221494	0.000817
1171	4260170	302315	0.001138
1278	4289520	357949	0.001338
1385	4282670	297638	0.001114
1491	4290250	363198	0.001357
1598	4326340	392664	0.001455
1704	4246170	369850	0.001397
1811	4283450	441105	0.001651
1917	4243000	407334	0.001539
2023	4313680	457114	0.001699
2129	4281740	513375	0.001922
2235	4288460	486497	0.001819

2342	4358380	495961	0.001825
2448	4348430	546793	0.002016
2554	4338090	517564	0.001913
2660	4268760	611671	0.002297
2767	4307530	599193	0.00223
2873	4243460	682796	0.00258
2979	4284590	644683	0.002413
3085	4275880	722863	0.002711
3192	4363860	640631	0.002354
3298	4361570	772016	0.002838
3404	4248770	895343	0.003379
3511	4222250	742140	0.002818
3617	4222480	744237	0.002826
3724	4294410	955420	0.003567
3830	4308600	858785	0.003196
3936	4335900	858685	0.003175
4043	4290980	849859	0.003176

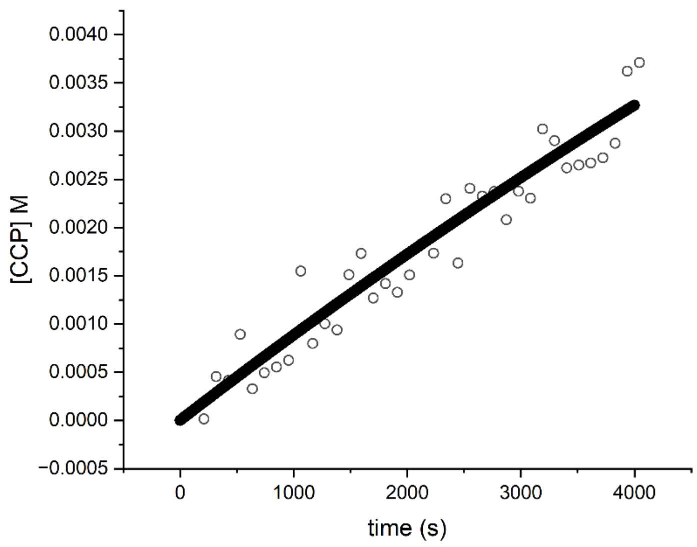

Figure S27. Formation of **2** from complex **15a** at –10 °C (Run 2)

Table S46. Data for the formation of **2** from complex **15a** at –10 °C (Run 2)

Time (s)	Integral IS (−140 ppm)	Integral CCP (−116 ppm)	[CCP] (M)
106	4469460	89141.8	0.000326
212	4344860	64959.9	0.000244
319	4436450	185987	0.000685
425	4433490	174961	0.000645
531	4337800	298093	0.001123
637	4422970	150867	0.000557
743	4325980	191516	0.000723
850	4391530	210775	0.000784
957	4416650	230469	0.000853
1063	4308510	468669	0.001777
1169	4343840	273216	0.001028
1276	4341740	327403	0.001232
1382	4383200	313622	0.001169
1488	4373810	466053	0.001741
1594	4345230	521237	0.00196
1702	4345020	398612	0.001499
1808	4374760	441823	0.00165
1914	4348870	414912	0.001559
2020	4349350	462478	0.001737

2127	4419220	557751	0.002062
2233	4374160	525655	0.001964
2339	4291010	663802	0.002528
2446	4401030	501128	0.00186
2552	4406060	710903	0.002636
2660	4419250	691211	0.002556
2766	4337470	691485	0.002605
2872	4339200	613629	0.002311
2979	4358490	695521	0.002607
3085	4349210	674613	0.002534
3191	4233300	842567	0.003252
3297	4250460	814681	0.003132
3404	4374350	762289	0.002847
3510	4250980	748484	0.002877
3616	4400490	780741	0.002899
3723	4352330	786571	0.002953
3830	4324620	821885	0.003105
3936	4242180	999852	0.003851
4043	4306610	1038660	0.003941

Experiment 14: Kinetic Study of the (dCypp)Pd[(4-F)Ph]Br **(16a)**

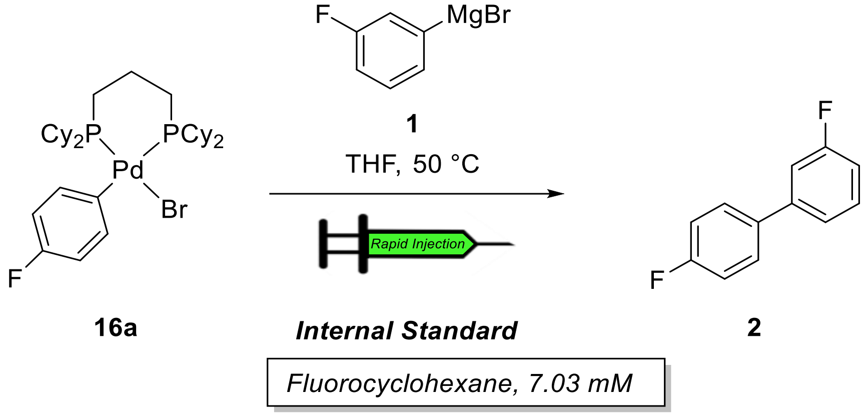

A 5-mL volumetic flask was charged with Fluorocyclohexane (4.51 µL, 41.5 µmol) followed by dissolving with THF to the 5-mL mark generating a 0.0083 M of stock solution. An oven-dried (150 °C), 5 mm, NMR tube was taken into the dry box and charged with **16a** (7.18 mg, 10 µmol) and 500 µL of the freshly prepared solution. The tube was capped with a septum. An oven-dried (60 °C) rapid injection barrel was taken into the glove box and charged with 3-Fluorophenylmagnesium bromide solution (500 µL, 1.33 M in THF). The glass capillary of the barrel was capped with a septum. The sample and the barrel were removed from the glove box and the sample was placed into the NMR probe set to –10 °C with the cap off. Then 3-Fluorophenylmagnesium bromide **1** (120 µmol) in THF (90 µL) was injected (RI-NMR).

Using the fluorine channel to collect a spectrum every 250 s the progress of the reaction was monitored by the decay of dCypp-Pd-Br in comparison with the internal reference 1,2-Difluorobenzene. The first order formation profile was fitted with the Curve Fitter Toolbox in Matlab using Equation 1.

Table S47. Summary of fits for the decay of **16a**

**Run**	**R^2 of fit**	***k*** **($10^{-2}s^{-1}$) (decay OAC)**	**[OAC]₀ (M)**
1	0.9898	0.0572 ± 0.0048	0.0193 ± 0.00107
2	0.9891	0.0623 ± 0.0060	0.0253 ± 0.00119

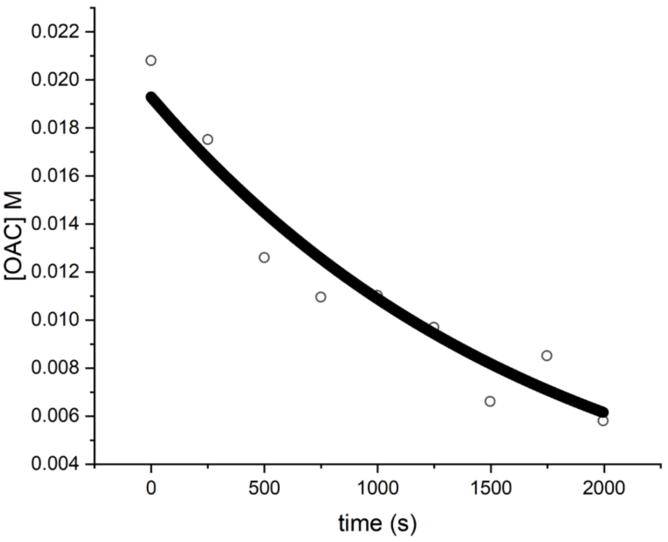

Figure S28. Decay of **16a** at 50 °C (Run 1)

Table S48. Data for the decay of **16a** at 50 °C (Run 1)

**Time (s)**	**Integral IS (−174 ppm)**	**Integral OAC (−125 ppm)**	**[OAC] (M)**
0	1680560	5031260	0.020804
250	1317350	3321420	0.017521
500	1472800	2670210	0.012599
749	1443270	2275650	0.010957
999	1313350	2084890	0.011031
1248	1343320	1875270	0.009701
1497	1532000	1457830	0.006613
1747	1232680	1509830	0.008512
1996	1239000	1034150	0.0058

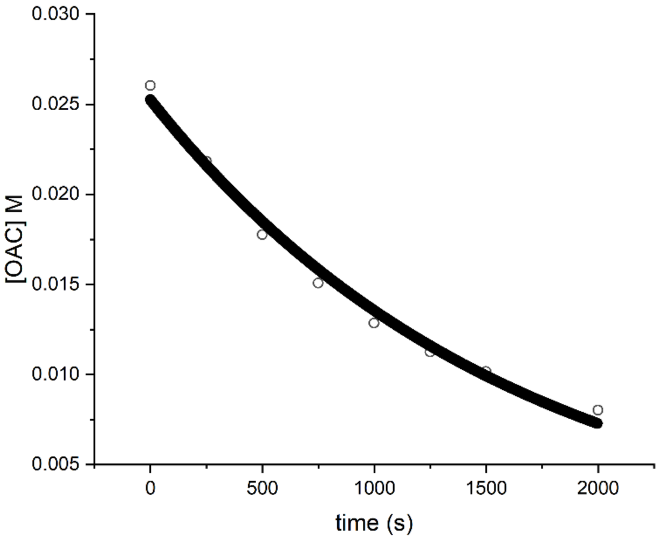

Figure S29. Decay of **16a** at 50 °C (Run 2)

Table S49. Data for the decay of **16a** at 50 °C (Run 2)

**Time (s)**	**Integral IS (−174 ppm)**	**Integral OAC (−125 ppm)**	**[OAC] (M)**
0	6866090	6417060	0.026042
250	6539270	5124850	0.021837
500	6495040	4140610	0.017764
749	6434520	3481700	0.015077
999	6435070	2968930	0.012856
1249	6383430	2579150	0.011258
1499	6422420	2342900	0.010165
1749	6692290	2044370	0.008512
1999	6621650	1907570	0.008027

2.4.1. Halide Effects

Experiment 15: Kinetic Study of the (XPhos)Pd[(4-F)Ph]Cl **(8b)**

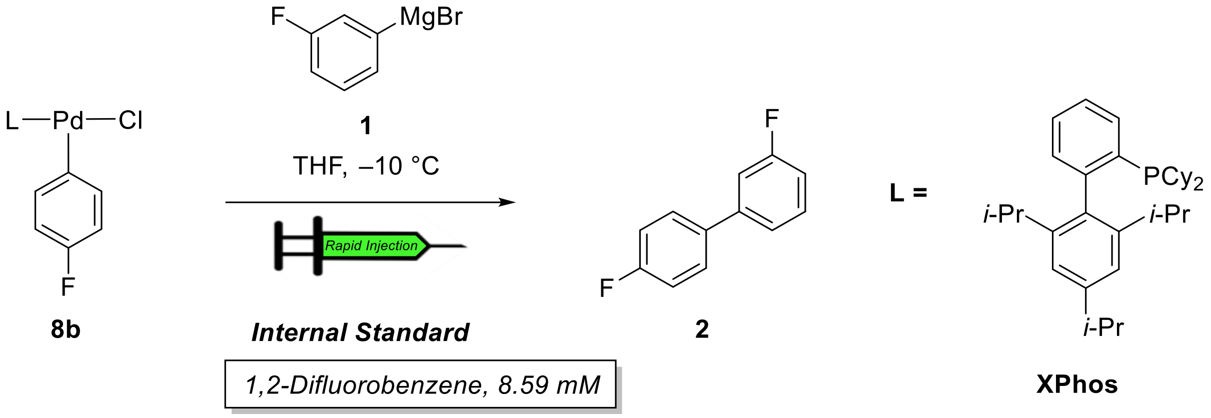

A 5-mL volumetic flask was charged with 1,2-Difluorobenzene (5.0 µL, 50.7 µmol) followed by dissolving with THF to the 5-mL mark generating a 0.01 M of stock solution. An oven-dried (150 °C), 5 mm, NMR tube was taken into the dry box and charged with **8b** (7.14 mg, 10 µmol) and 500 µL of the freshly prepared solution. The tube was capped with a septum. An oven-dried (60 °C) rapid injection barrel was taken into the glove box and charged with 3-Fluorophenylmagnesium bromide solution (500 µL, 1.33 M in THF). The glass capillary of the barrel was capped with a septum. The sample and the barrel were removed from the glove box and the sample was placed into the NMR probe set to –10 °C with the cap off. Then 3-Fluorophenylmagnesium bromide **1** (120 µmol) in THF (90 µL) was injected (RI-NMR).

Using the fluorine channel to collect a spectrum every 1.6 s the progress of the reaction was monitored by the formation of cross-coupling product in comparison with the internal reference 1,2-Difluorobenzene. The first order formation profile was fitted with the Curve Fitter Toolbox in Matlab using Equation 1.

Table S50. Summary of fits for the formation of **2**

Run	R^2 of fit	k ($10^{-2}s^{-1}$) (form CCP)	$[OAC]_o$ (M)
1	0.9861	22.00 ± 8.78	0.0171 ± 0.0029
2	0.9992	21.15 ± 1.26	0.0169 ± 0.0005

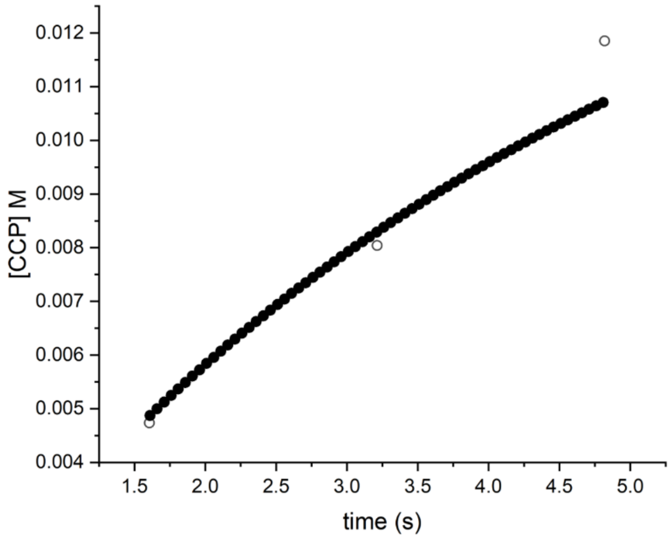

Figure S30. Formation of **2** from complex **8b** at –10 °C (Run 1)

Table S51 Data for the formation of **2** from complex **8b** at –10 °C (Run 1)

**Time (s)**	**Integral IS (−140 ppm)**	**Integral CCP (−116 ppm)**	**[CCP] (M)**
1.61	229506	67642.4	0.004496
3.21	409048	209213	0.007802
4.82	343346	261406	0.011614

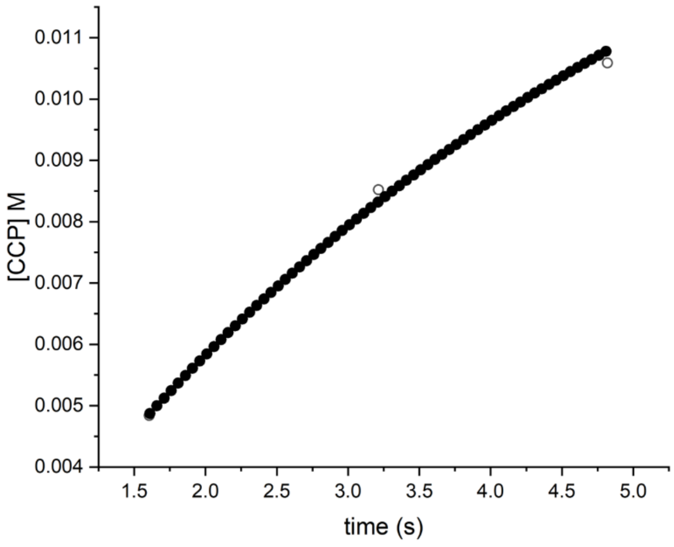

Figure S31. Formation of **2** from complex **8b** at –10 °C (Run 2)

Table S52. Data for the formation of **2** from complex **8b** at –10 °C (Run 2)

**Time (s)**	**Integral IS (−140 ppm)**	**Integral CCP (−116 ppm)**	**[CCP] (M)**
1.61	373348	117420	0.004862
3.21	371539	205243	0.008539
4.82	353238	242460	0.01061

Experiment 16: Kinetic Study of the (RuPhos)Pd[(4-F)Ph]I **(11c)**

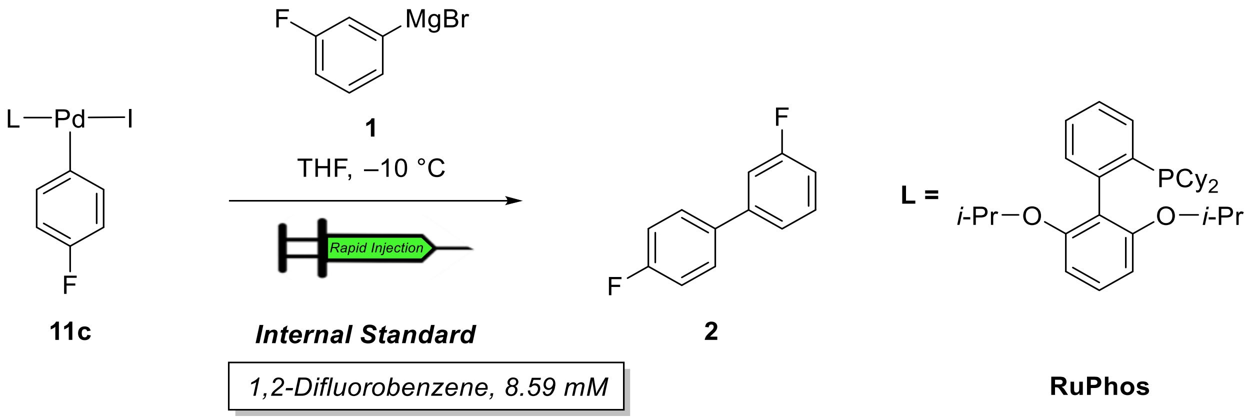

A 5-mL volumetic flask was charged with 1,2-Difluorobenzene (5.0 µL, 50.7 µmol) followed by dissolving with THF to the 5-mL mark generating a 0.01 M of stock solution. An oven-dried (150 °C), 5 mm, NMR tube was taken into the dry box and charged with **11c** (7.95 mg, 10 µmol) and 500 µL of the freshly prepared solution. The tube was capped with a septum. An oven-dried (60 °C) rapid injection barrel was taken into the glove box and charged with 3-Fluorophenylmagnesium bromide solution (500 µL, 1.33 M in THF). The glass capillary of the barrel was capped with a septum. The sample and the barrel were removed from the glove box and the sample was placed into the NMR probe set to –10 °C with the cap off. Then 3-Fluorophenylmagnesium bromide **1** (120 µmol) in THF (90 µL) was injected (RI-NMR).

Using the fluorine channel to collect a spectrum every 1.6 s the progress of the reaction was monitored by the formation of cross-coupling product in comparison with the internal reference 1,2-Difluorobenzene. The first order formation profile was fitted with the Curve Fitter Toolbox in Matlab using Equation 1.

Table S53. Summary of fits for the formation of **2**

Run	R^2 of fit	k ($10^{-2}s^{-1}$) (form CCP)	$[OAC]_o$ (M)
1	0.9836	5.62 ± 0.67	0.0171 ± 0.00069
2	0.9783	3.34 ± 0.58	0.0167 ± 0.00051

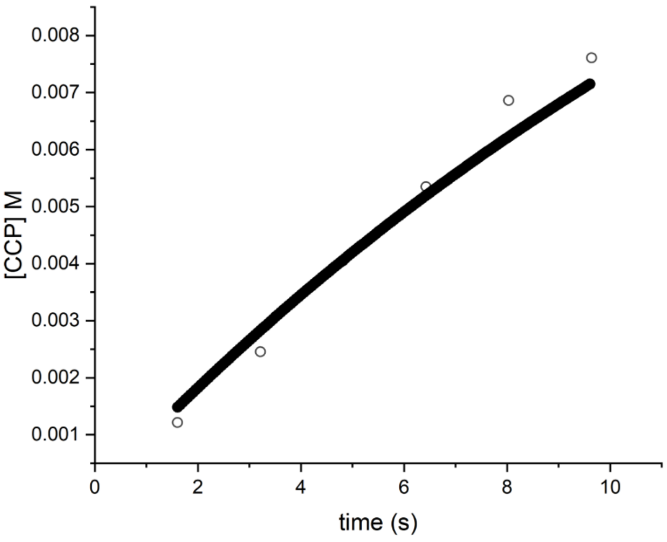

Figure S32. Formation of **2** from complex **11c** at –10 °C (Run 1)

Table S54. Data for the formation of **2** from complex **11c** at –10 °C (Run 1)

**Time (s)**	**Integral IS (−140 ppm)**	**Integral CCP (−116 ppm)**	**[CCP] (M)**
1.61	285578	16503.5	0.000995
3.21	419785	54448.5	0.002234
4.82	410272	91274.1	0.003831
6.43	393685	117245	0.005128
8.03	393564	151808	0.006642
9.64	383846	164686	0.007388

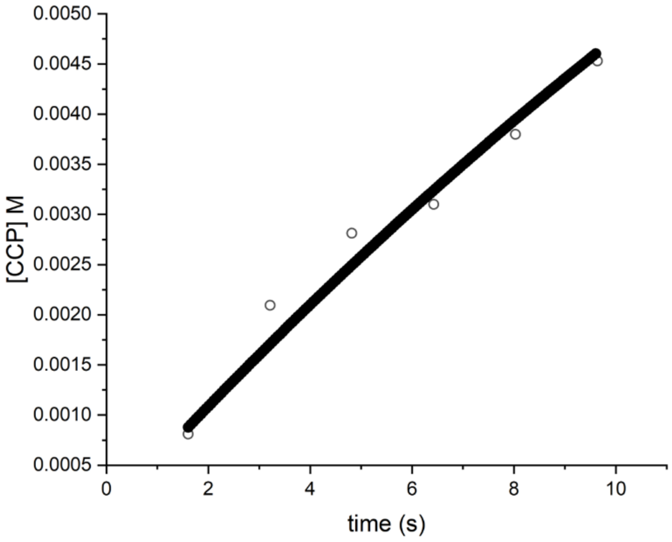

Figure S33. Formation of **2** from complex **11c** at –10 °C (Run 2)

Table S55. Data for the formation of **2** from complex **11c** at –10 °C (Run 2)

**Time (s)**	**Integral IS (−140 ppm)**	**Integral CCP (−116 ppm)**	**[CCP] (M)**
1.61	378803	27624	0.00106
3.21	363243	58535.9	0.002343
4.82	361629	76133.4	0.003062
6.43	378852	87302.8	0.003351
8.03	395088	109984	0.004048
9.64	349176	114730	0.004778

Experiment 17: Kinetic Study of the (RuPhos)Pd[(4-F)Ph]Cl **(11b)**

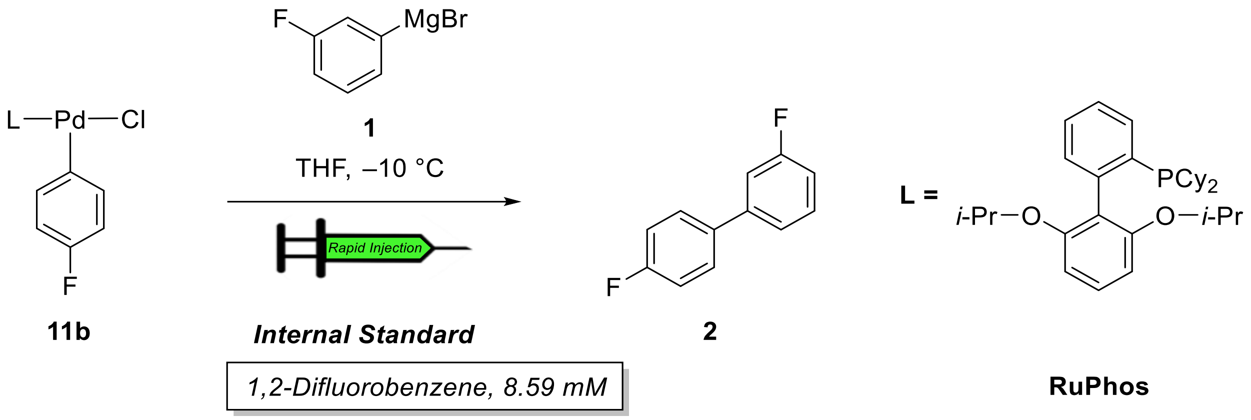

A 5-mL volumetic flask was charged with 1,2-Difluorobenzene (5.0 µL, 50.7 µmol) followed by dissolving with THF to the 5-mL mark generating a 0.01 M of stock solution. An oven-dried (150 °C), 5 mm, NMR tube was taken into the dry box and charged with **11b** (7.04 mg, 10 µmol) and 500 µL of the freshly prepared solution. The tube was capped with a septum. An oven-dried (60 °C) rapid injection barrel was taken into the glove box and charged with 3-Fluorophenylmagnesium bromide solution (500 µL, 1.33 M in THF). The glass capillary of the barrel was capped with a septum. The sample and the barrel were removed from the glove box and the sample was placed into the NMR probe set to –10 °C with the cap off. Then 3-Fluorophenylmagnesium bromide **1** (120 µmol) in THF (90 µL) was injected (RI-NMR).

Using the fluorine channel to collect a spectrum every 1.6 s the progress of the reaction was monitored by the formation of cross-coupling product in comparison with the internal reference 1,2-Difluorobenzene. The first order formation profile was fitted with the Curve Fitter Toolbox in Matlab using Equation 1.

Table S56. Summary of fits for the formation of **2**

Run	R^2 of fit	k ($10^{-2}s^{-1}$) (form CCP)	$[OAC]_o$ (M)
1	0.9823	18.59 ± 0.083	0.0173 ± 0.00293
2	0.9954	20.62 ± 4.72	0.0170 ± 0.00156

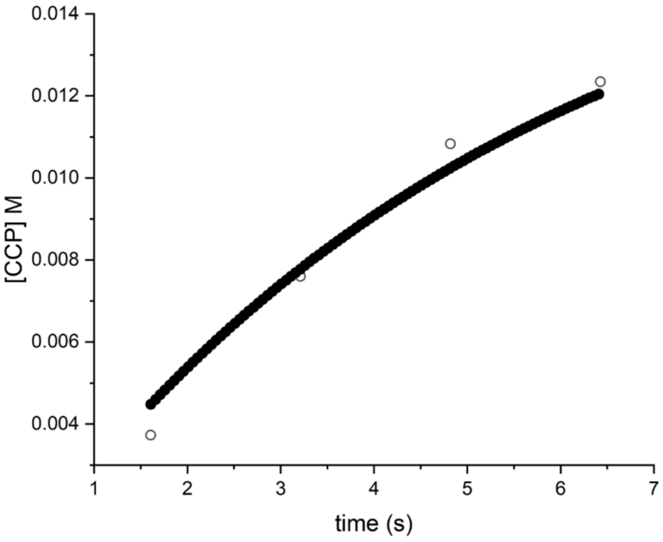

Figure S34. Formation of **2** from complex **11b** at –10 °C (Run 1)

Table S57. Data for the formation of **2** at –10 °C (Run 1)

**Time (s)**	**Integral IS (−140 ppm)**	**Integral CCP (−116 ppm)**	**[CCP] (M)**
1.61	409101	79067.8	0.003328
3.21	405375	169471	0.007199
4.82	381300	231041	0.010434
6.43	374608	259805	0.011943

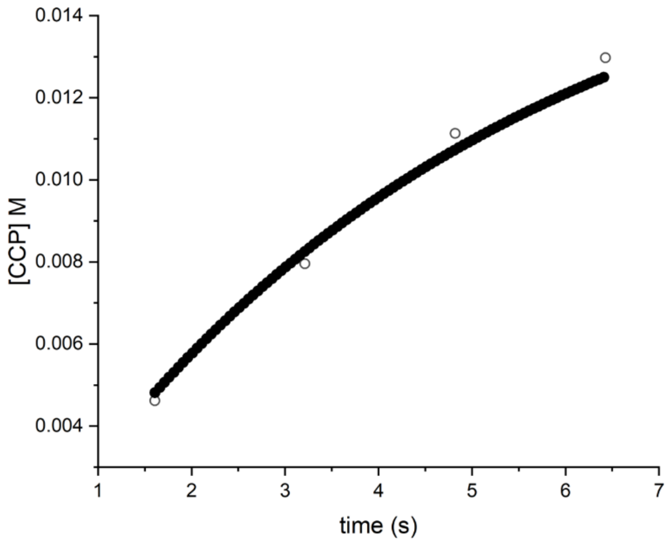

Figure S35. Formation of **2** from complex **11b** at –10 °C (Run 2)

Table S58. Data for the formation of **2** from complex **11b** at –10 °C (Run 2)

**Time (s)**	**Integral IS (−140 ppm)**	**Integral CCP (−116 ppm)**	**[CCP] (M)**
1.61	409400	106383	0.004475
3.21	420934	190978	0.007813
4.82	390343	249185	0.010993
6.43	375793	280096	0.012835

Experiment 18: Kinetic Study of the (CPhos)Pd[(4-F)Ph]I **(14c)**

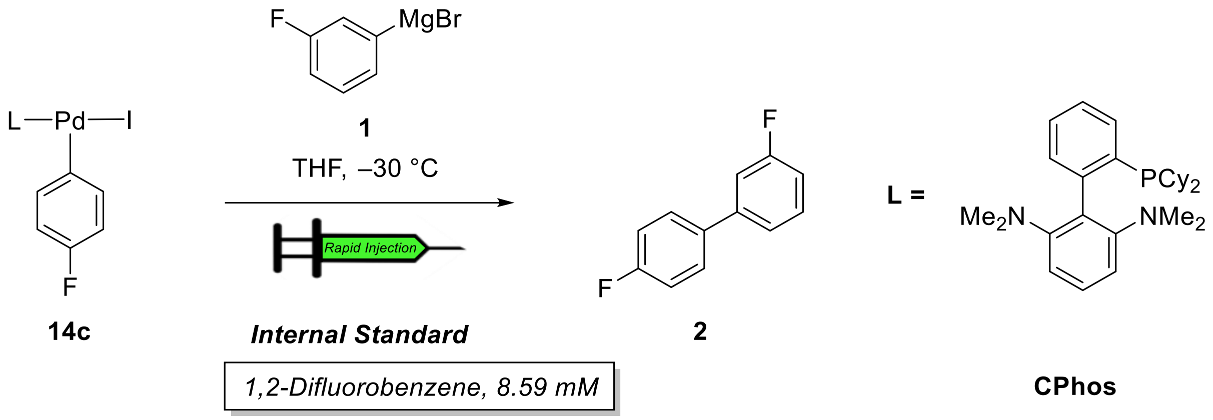

A 5-mL volumetic flask was charged with 1,2-Difluorobenzene (5.0 µL, 50.7 µmol) followed by dissolving with THF to the 5-mL mark generating a 0.01 M of stock solution. An oven-dried (150 °C), 5 mm, NMR tube was taken into the dry box and charged with **14c** (7.65 mg, 10 µmol) and 500 µL of the freshly prepared solution. The tube was capped with a septum. An oven-dried (60 °C) rapid injection barrel was taken into the glove box and charged with 3-Fluorophenylmagnesium bromide solution (500 µL, 1.33 M in THF). The glass capillary of the barrel was capped with a septum. The sample and the barrel were removed from the glove box and the sample was placed into the NMR probe set to –30 °C with the cap off. Then 3-Fluorophenylmagnesium bromide **1** (120 µmol) in THF (90 µL) was injected (RI-NMR).

Using the fluorine channel to collect a spectrum every 1.6 s the progress of the reaction was monitored by the formation of cross-coupling product in comparison with the internal reference 1,2-Difluorobenzene. The first order formation profile was fitted with the Curve Fitter Toolbox in Matlab using Equation 1.

Table S59. Summary of fits for the formation of **2**

Run	R^2 of fit	k ($10^{-2}s^{-1}$) (form CCP)	$[OAC]_o$ (M)
1	0.9535	3.02 ± 0.314	0.0156 ± 0.0007
2	0.9736	4.07 ± 0.483	0.0170 ± 0.0007

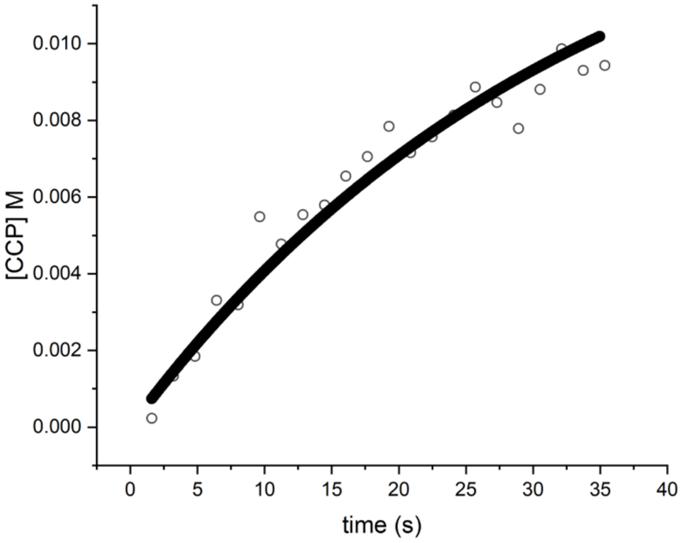

Figure S36. Formation of **2** from complex **14c** at –30 °C (Run 1)

Table S60. Data for the formation of **2** at –30 °C (Run 1)

**Time (s)**	**Integral IS (−140 ppm)**	**Integral CCP (−116 ppm)**	**[CCP] (M)**
1.61	517731	42387.3	0.001499
3.21	500895	71117	0.002599
4.82	513478	87367.5	0.003115
6.43	492210	123067	0.004577
8.03	475847	116007	0.004463
9.64	473260	174717	0.006758
11.25	482047	159139	0.006043
12.85	462302	171926	0.006807
14.46	481526	185714	0.00706
16.07	494948	211240	0.007812
17.67	443040	201512	0.008326
19.28	457213	227544	0.00911
20.89	478239	220122	0.008425
22.49	478937	231377	0.008843
24.10	486693	249981	0.009402
25.71	501133	277577	0.010139
27.31	489524	260449	0.009739
28.92	509665	252248	0.00906
30.53	489055	269249	0.010078
32.13	466139	283665	0.011139
33.74	488724	282363	0.010576

35.35	489698	286361	0.010704

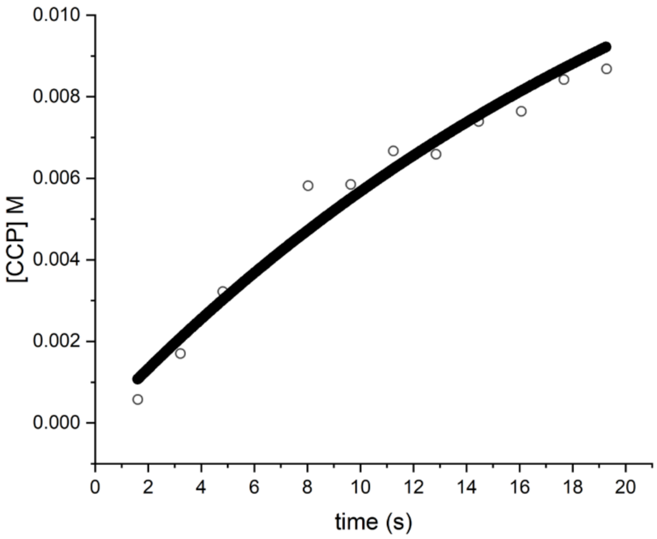

Figure S37. Formation of **2** from complex **14c** at –30 °C (Run 2)

Table S61. Data for the formation of **2** at –30 °C (Run 2)

Time (s)	Integral IS (−140 ppm)	Integral CCP (−116 ppm)	[CCP] (M)
1.61	552337	15546.7	0.000515
3.21	516636	46255.1	0.001639
4.82	504624	87145.5	0.003161
6.43	497011	104030	0.003831
8.03	468004	147178	0.005757
9.64	462581	146276	0.005788
11.25	465596	168117	0.00661
12.85	473859	169067	0.006531
14.46	450848	180523	0.007329
16.07	461777	191304	0.007583
17.67	425626	194383	0.00836
19.28	452662	213240	0.008623

Experiment 19: Kinetic Study of the (CPhos)Pd[(4-F)Ph]Cl **(14b)**

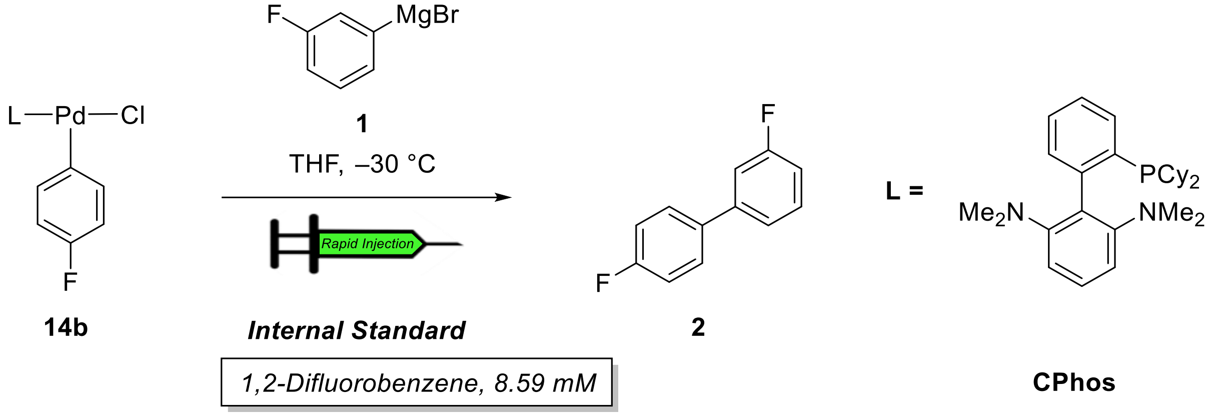

A 5-mL volumetic flask was charged with 1,2-Difluorobenzene (5.0 µL, 50.7 µmol) followed by dissolving with THF to the 5-mL mark generating a 0.01 M of stock solution. An oven-dried (150 °C), 5 mm, NMR tube was taken into the dry box and charged with **14b** (6.74 mg, 10 µmol) and 500 µL of the freshly prepared solution. The tube was capped with a septum. An oven-dried (60 °C) rapid injection barrel was taken into the glove box and charged with 3-Fluorophenylmagnesium bromide solution (500 µL, 1.33 M in THF). The glass capillary of the barrel was capped with a septum. The sample and the barrel were removed from the glove box and the sample was placed into the NMR probe set to –30 °C with the cap off. Then 3-Fluorophenylmagnesium bromide **1** (120 µmol) in THF (90 µL) was injected (RI-NMR).

Using the fluorine channel to collect a spectrum every 1.6 s the progress of the reaction was monitored by the formation of cross-coupling product in comparison with the internal reference 1,2-Difluorobenzene. The first order formation profile was fitted with the Curve Fitter Toolbox in Matlab using Equation 1.

Table S62. Summary of fits for the formation of **2**

**Run**	**R^2 of fit**	***k*** **($10^{-2}s^{-1}$) (form CCP)**	**$[OAC]_o$ (M)**
1	0.9833	13.72 ± 2.71	0.0175 ± 0.0015
2	0.9837	11.66 ± 2.22	0.0169 ± 0.0013

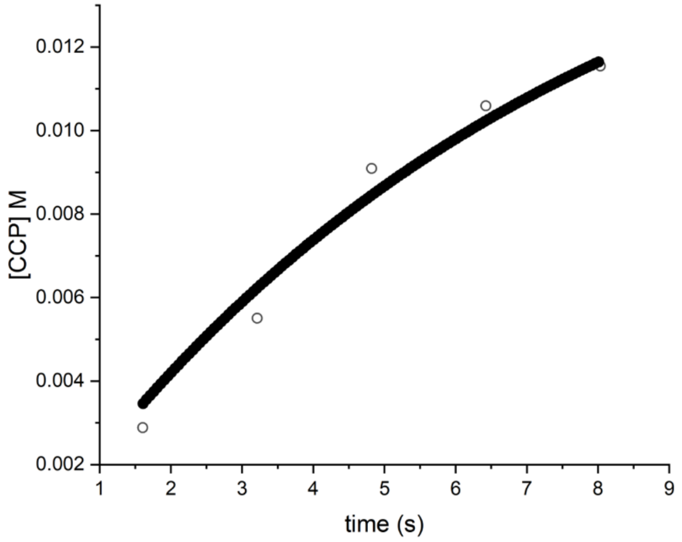

Figure S38. Formation of **2** from complex **14b** at –30 °C (Run 1)

Table S63. Data for the formation of **2** from complex **14b** at –30 °C (Run 1)

Time (s)	Integral IS (−140 ppm)	Integral CCP (−116 ppm)	[CCP] (M)
1.61	318556	40434.4	0.002323
3.21	498953	134812	0.004946
4.82	446082	207947	0.008533
6.43	433402	237538	0.010033
8.03	458245	274917	0.010982

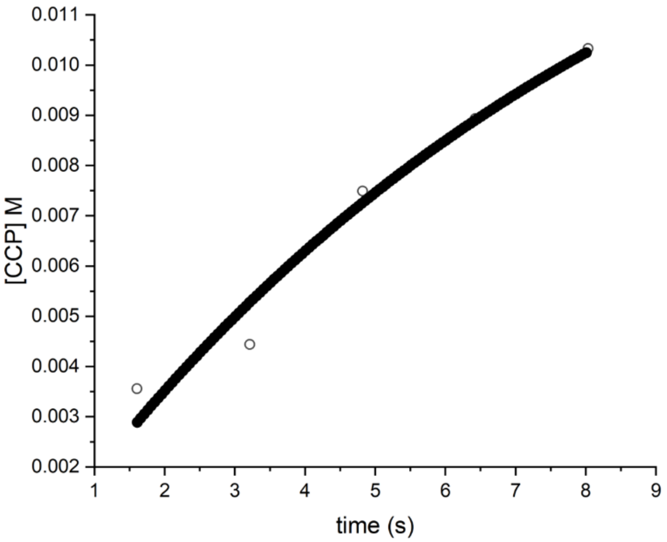

Figure S39. Formation of **2** from complex **14b** at –30 °C (Run 2)

Table S64. Data for the formation of **2** from complex **14b** at –30 °C (Run 2)

**Time (s)**	**Integral IS (−140 ppm)**	**Integral CCP (−116 ppm)**	**[CCP] (M)**
1.61	366155	71541	0.003577
3.21	508961	124053	0.004462
4.82	511612	209900	0.00751
6.43	500084	244528	0.008951
8.03	503376	284626	0.01035

Experiment 20: Kinetic Study of the (CPhos)Pd[(4-F)Ph]Br **(14a)**

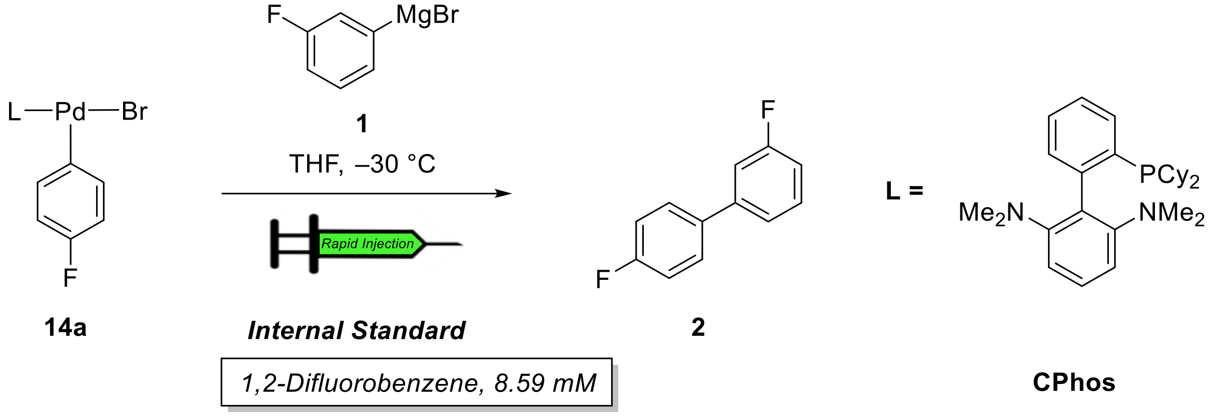

A 5-mL volumetic flask was charged with 1,2-Difluorobenzene (5.0 µL, 50.7 µmol) followed by dissolving with THF to the 5-mL mark generating a 0.01 M of stock solution. An oven-dried (150 °C), 5 mm, NMR tube was taken into the dry box and charged with **14a** (7.18 mg, 10 µmol) and 500 µL of the freshly prepared solution. The tube was capped with a septum. An oven-dried (60 °C) rapid injection barrel was taken into the glove box and charged with 3-Fluorophenylmagnesium bromide solution (500 µL, 1.33 M in THF). The glass capillary of the barrel was capped with a septum. The sample and the barrel were removed from the glove box and the sample was placed into the NMR probe set to –30 °C with the cap off. Then 3-Fluorophenylmagnesium bromide **1** (120 µmol) in THF (90 µL) was injected (RI-NMR).

Using the fluorine channel to collect a spectrum every 1.6 s the progress of the reaction was monitored by the formation of cross-coupling product in comparison with the internal reference 1,2-Difluorobenzene. The first order formation profile was fitted with the Curve Fitter Toolbox in Matlab using Equation 1.

Table S65. Summary of fits for the formation of **2**

**Run**	**R^2 of fit**	***k*** **($10^{-2}s^{-1}$) (form CCP)**	**$[OAC]_o$ (M)**
1	0.9978	21.65 ± 1.68	0.0170 ± 0.0007
2	0.9873	18.20 ± 3.25	0.0167 ± 0.0015

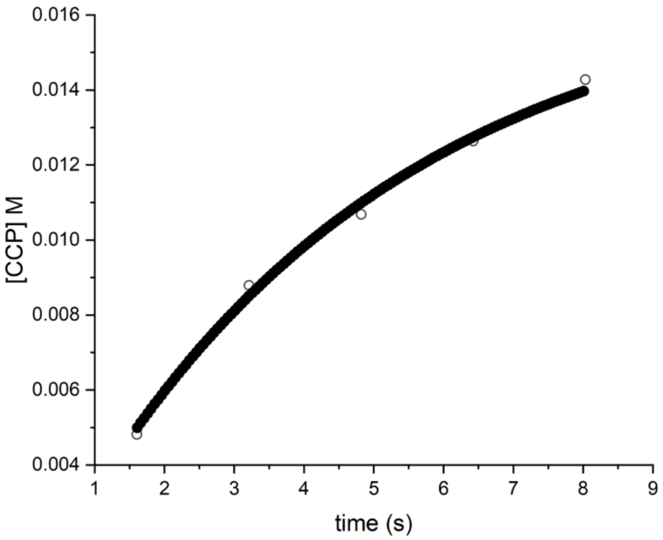

Figure S40. Formation of **2** from complex **14a** at –30 °C (Run 1)

Table S66. Data for the formation of **2** from complex **14a** at –30 °C (Run 1)

**Time (s)**	**Integral IS (−140 ppm)**	**Integral CCP (−116 ppm)**	**[CCP] (M)**
1.61	462687	144225	0.004755
3.21	455117	260378	0.008727
4.82	422462	294268	0.010625
6.43	413002	340541	0.012578
8.03	393636	366921	0.014219

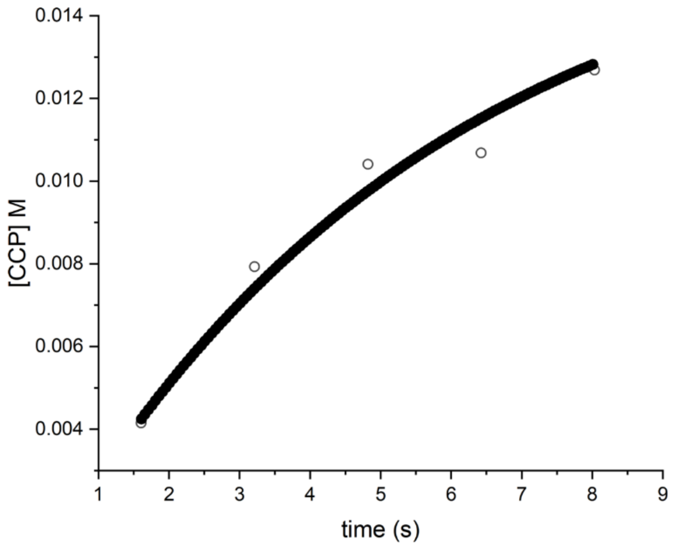

Figure S41. Formation of **2** from complex **14a** at –30 °C (Run 2)

Table S67. Data for the formation of **2** from complex **14a** at –30 °C (Run 2)

**Time (s)**	**Integral IS (−140 ppm)**	**Integral CCP (−116 ppm)**	**[CCP] (M)**
1.61	473481	134775	0.004342
3.21	438249	233360	0.008123
4.82	442977	307702	0.010596
6.43	440960	314249	0.010871
8.03	431645	364472	0.01288

2.4.3. Eyring Analysis

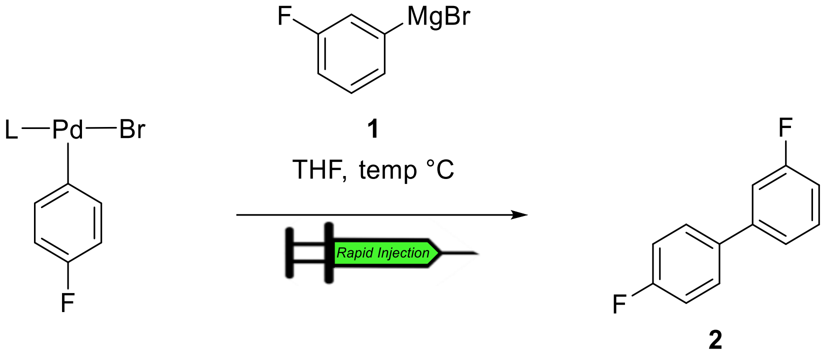

L = SPhos or Ph_3P, or CPhos

A 5-mL volumetic flask was charged with 1,2-Difluorobenzene (5.0 µL, 50.7 µmol) followed by dissolving with THF to the 5-mL mark generating a 0.01 M of stock solution. An oven-dried (150 °C), 5 mm, NMR tube was taken into the dry box and charged with corresponding oxidative addition complex (xx mg, 10 µmol) and 500 µL of the freshly prepared solution. The tube was capped with a septum. An oven-dried (60 °C) rapid injection barrel was taken into the glove box and charged with 3-Fluorophenylmagnesium bromide solution (500 µL, 1.33 M in THF). The glass capillary of the barrel was capped with a septum. The sample and the barrel were removed from the glove box and the sample was placed into the NMR probe set to –30 °C or –20 °C or -10 °C or 0 °C, or 10 °C with the cap off. Then 3-Fluorophenylmagnesium bromide **1** (120 µmol) in THF (90 µL) was injected (RI-NMR).

Using the fluorine channel to collect a spectrum the progress of the reaction was monitored by the formation of cross-coupling product in comparison with the internal reference 1,2-Difluorobenzene. The first order formation profile was fitted with the Curve Fitter Toolbox in Matlab using Equation 1. Then using the Eyring equation 2 $\Delta G^‡$, $\Delta H^‡$, and $\Delta S^‡$ were calculated.

Equation 2. Eyring equation

$$\ln\frac{k}{T} = \frac{-\Delta H^{\ddagger}}{R}\frac{1}{T} + \ln\frac{k_B}{h} + \frac{\Delta S^{\ddagger}}{R}$$

Table S68. Activation Parameters for the Transmetalation

Entry	Ligand	Complex	$\Delta G^{\ddagger}_{263.15}$, kcal/mol	$\Delta H^{\ddagger}$, kcal/mol	$\Delta S^{\ddagger}$, kcal/mol·K
1	SPhos	**3a**	19.44 ± 3.31	24.74 ± 1.55	0.020 ± 0.0057
2	Ph_3P	**5a**	18.91 ± 0.84	14.68 ± 0.43	-0.016 ± 0.0016
3	CPhos	**14a**	15.88 ± 0.50	3.620 ± 0.25	-0.047 ± 0.00097

Table S69. Results from the cross-coupling reaction with **3a**

Entry	Temp. (K)	*k* (10^{-2} s^{-1}) (Form 2)	$[OAC]_0$ M
1	263.15	0.038 ± 0.0009	0.015 ± 0.0002
2	263.15	0.043 ± 0.0003	0.016 ± 0.00007
3	273.15	0.27 ± 0.000178	0.0159 ± 0.0004
4	273.15	0.29 ± 0.000393	0.0160 ± 0.0006
5	283.15	1.16 ± 0.15	0.0166 ± 0.0009
6	283.15	1.25 ± 0.31	0.0173 ± 0.0017

Table S70. Averages of results from cross-coupling reaction with **3a**

Entry	Temp. (K)	*k* (10^{-2} s^{-1}) (Form 2)
1	263.15	0.041 ± 0.006
2	273.15	0.280 ± 0.010
3	283.15	1.210 ± 0.050

Table S71. Data for Eyring analysis with **3a**

Entry	Temp. (K)	*k* (10^{-2} s^{-1}) (Form 2)	ln(k/T)	1/T
1	263.15	0.041 ± 0.006	-13.40	0.00380
2	273.15	0.280 ± 0.010	-11.49	0.00366
3	283.15	1.210 ± 0.050	-10.06	0.00353

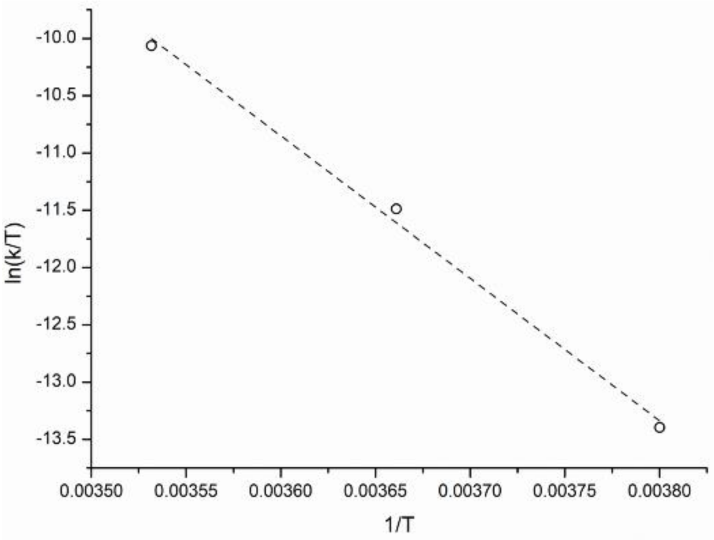

Figure S42. Eyring plot with **3a**

Table S72. Results from Eyring analysis with **3a**

**equation**	y = –(12430±780)*x + (33.898±2.86)
**R^2**	0.9961

	Value
**$\Delta G^‡$**	19.44 ± 3.31 (kcal/mol) at –10 °C
**$\Delta H^‡$**	24.7 ± 1.55 (kcal/mol)
**$\Delta S^‡$**	0.02 ± 0.0057 (kcal/mol·K)

Table S73. Results from the cross-coupling reaction with **5a**

**Entry**	**Temp. (K)**	***k*** **(10^{-2} s^{-1}) (Form 2)**	**$[OAC]_0$ M**
1	263.15	0.12 ± 0.002	0.0159 ± 0.00013
2	263.15	0.11 ± 0.003	0.0157 ± 0.00016
3	273.15	0.32 ± 0.018	0.0163 ± 0.0003
4	273.15	0.38 ± 0.016	0.0172 ± 0.0003
5	283.15	0.93 ± 0.209	0.0172 ± 0.0013
6	283.15	0.86 ± 0.108	0.0168 ± 0.0007

Table S74. Averages of results from cross-coupling reaction with **5a**

**Entry**	**Temp. (K)**	***k*** **(10^{-2} s^{-1}) (Form 2)**
1	263.15	0.12 ± 0.005
2	273.15	0.35 ± 0.03
3	283.15	0.90 ± 0.04

Table S75. Data for Eyring analysis with **5a**

**Entry**	**Temp. (K)**	***k*** **(10^{-2} s^{-1}) (Form 2)**	**ln(k/T)**	**1/T**
1	263.15	0.12 ± 0.005	-12.34	0.00380
2	273.15	0.35 ± 0.03	-11.27	0.00366
3	283.15	0.90 ± 0.04	-10.36	0.00353

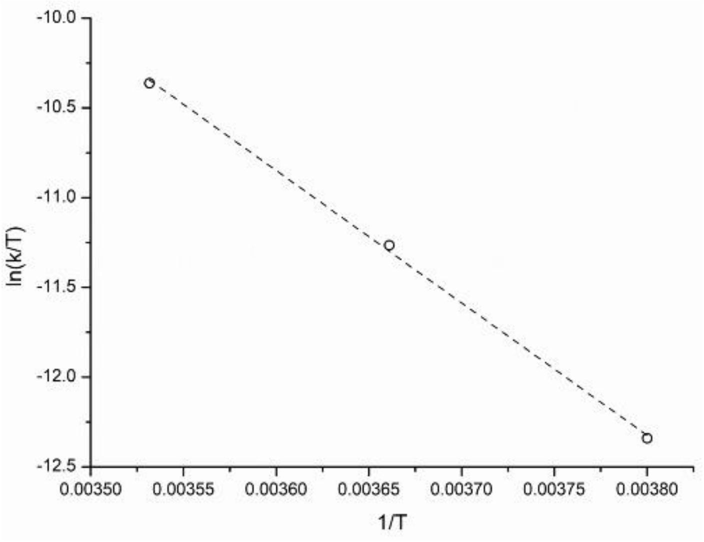

Figure S43. Eyring plot with **5a**

Table S76. Results from Eyring analysis with 5**a**

**equation**	y = –(7376.1±215)*x + (15.705±0.79)
**R^2**	0.9991

	Value
**ΔG‡**	18.91±0.10 (kcal/mol) at –10 °C
**ΔH‡**	14.68±0.43 (kcal/mol)
**ΔS‡**	-0.016±0.002 (kcal/mol·K)

Table S77. Results from the cross-coupling reaction with **14a**

Entry	Temp. (K)	*k* (10^{-2} s^{-1}) (Form 2)	$[OAC]_0$ M
1	243.15	21.65 ± 1.68	0.0170 ± 0.00070
2	243.15	18.20 ± 3.25	0.0167 ± 0.00150
3	253.15	26.99 ± 9.23	0.0160 ± 0.00290
4	253.15	30.66 ± 7.65	0.0167 ± 0.00210
5	263.15	31.61 ± 5.13	0.0172 ± 0.00154
6	263.15	44.52 ± 6.00	0.0168 ± 0.00113

Table S78. Averages of results from cross-coupling reaction with **14a**

Entry	Temp. (K)	*k* (10^{-2} s^{-1}) (Form 2)
1	243.15	19.93 ± 1.73
2	253.15	28.83 ± 1.84
3	263.15	38.07 ± 6.46

Table S79. Data for Eyring analysis with **14a**

Entry	Temp. (K)	*k* (10^{-2} s^{-1}) (Form 2)	ln(k/T)	1/T
1	243.15	19.93 ± 1.73	-7.11	0.00411
2	253.15	28.83 ± 1.84	-6.78	0.00395
3	263.15	38.07 ± 6.46	-7.11	0.00380

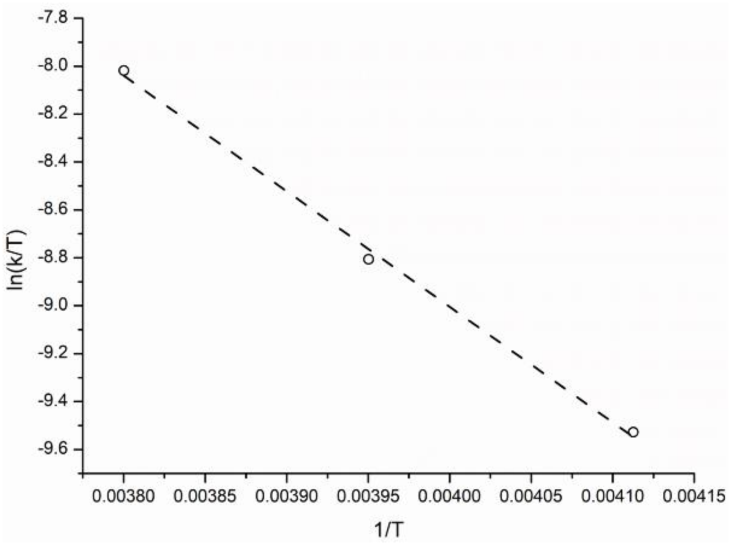

Figure S44. Eyring plot with **14a**

Table S80. Results from Eyring analysis with **14a**

**equation**	y = –(1821±124)*x + (0.39±0.048)
**R^2**	0.9954

	Value
**$\Delta G^‡$**	15.87±0.50 (kcal/mol) at –10 °C
**$\Delta H^‡$**	3.62±0.25 (kcal/mol)
**$\Delta S^‡$**	–0.0466±0.00097 (kcal/mol·K)

Experiment 21: Kinetic Study of the (SPhos)Pd[(4-F)Ph]Br **(3a)** at 0 °C

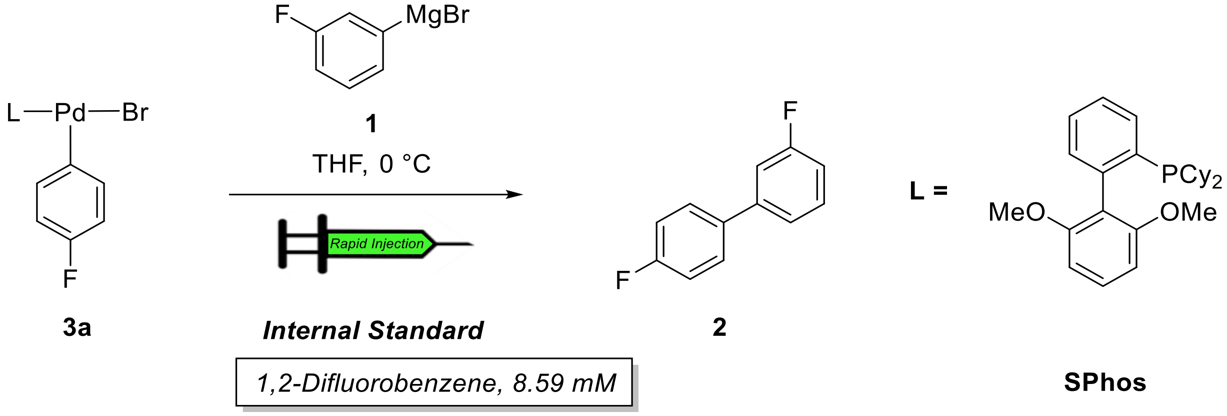

A 5-mL volumetic flask was charged with 1,2-Difluorobenzene (5.0 µL, 50.7 µmol) followed by dissolving with THF to the 5-mL mark generating a 0.01 M of stock solution. An oven-dried (150 °C), 5 mm, NMR tube was taken into the dry box and charged with **3a** (6.92 mg, 10 µmol) and 500 µL of the freshly prepared solution. The tube was capped with a septum. An oven-dried (60 °C) rapid injection barrel was taken into the glove box and charged with 3-Fluorophenylmagnesium bromide solution (500 µL, 1.33 M in THF). The glass capillary of the barrel was capped with a septum. The sample and the barrel were removed from the glove box and the sample was placed into the NMR probe set to 0 °C with the cap off. Then 3-Fluorophenylmagnesium bromide **1** (120 µmol) in THF (90 µL) was injected (RI-NMR).

Using the fluorine channel to collect a spectrum every 19 s the progress of the reaction was monitored by the formation of cross-coupling product in comparison with the internal reference 1,2-Difluorobenzene. The first order formation profile was fitted with the Curve Fitter Toolbox in Matlab using Equation 1.

Table S81. Summary of fits for the formation of **2**

**Run**	**R^2 of fit**	***k*** **($10^{-2}s^{-1}$) (form CCP)**	**$[OAC]_o$ (M)**
1	0.9852	0.27 ± 0.000178	0.0159 ± 0.0004
2	0.9663	0.29 ± 0.000393	0.0160 ± 0.0006

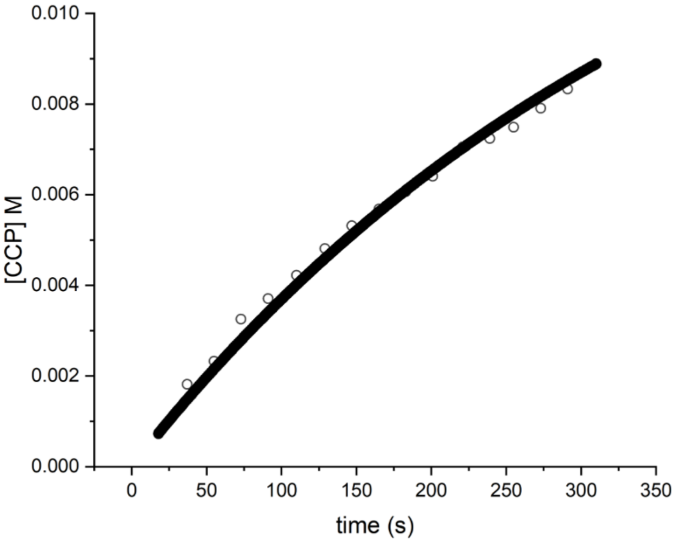

Figure S45.*1* Formation of **2** from complex **3a** at 0 °C (Run 1)

Table S82. Data for the formation of **2** from complex **3a** at 0 °C (Run 1)

Time (s)	Integral IS (−140 ppm)	Integral CCP (−116 ppm)	[CCP] (M)
18	2066990	236829	0.001771
37	1996220	370445	0.002869
55	2036900	444933	0.003377
73	2125910	591782	0.004303
91	2090920	643521	0.004757
110	2070350	705800	0.00527
129	2013600	763733	0.005863
147	2095610	862683	0.006363
165	2099120	913927	0.00673
183	2095100	964337	0.007115
201	2108910	1017470	0.007458
221	1970330	1033030	0.008104
239	2053260	1101070	0.008289
255	2114590	1168210	0.00854
273	2087190	1209440	0.008957
291	2078680	1261450	0.00938
310	2027670	1304320	0.009943

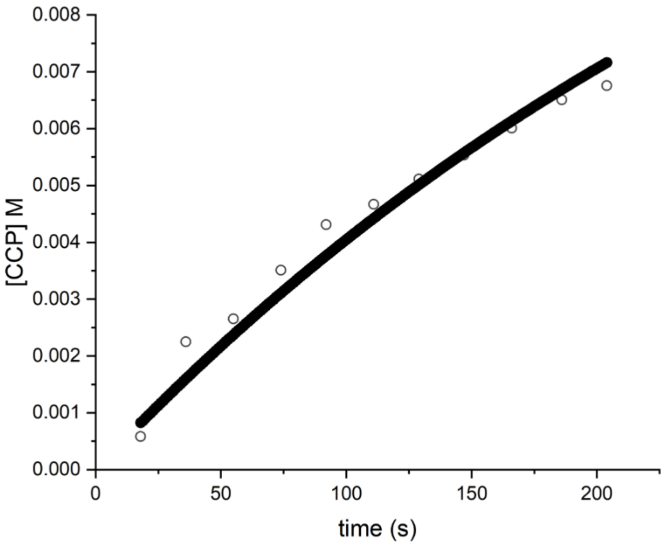

Figure S46. Formation of **2** from complex **3a** at 0 °C (Run 2)

Table S83. Data for the formation of **2** from complex **3a** at 0 °C (Run 2)

**Time (s)**	**Integral IS (−140 ppm)**	**Integral CCP (−116 ppm)**	**[CCP] (M)**
18	2019200	193898	0.001484
36	1986520	404735	0.003149
55	1940060	445963	0.003553
74	1955440	557762	0.004409
92	2041980	688420	0.005211
111	2018190	726828	0.005567
129	2023770	786985	0.006011
147	2061830	858098	0.006433
166	2042900	912912	0.006908
186	2009790	962983	0.007406
204	2009460	995089	0.007655

Experiment 22: Kinetic Study of the (SPhos)Pd[(4-F)Ph]Br **(3a)** at 10 °C

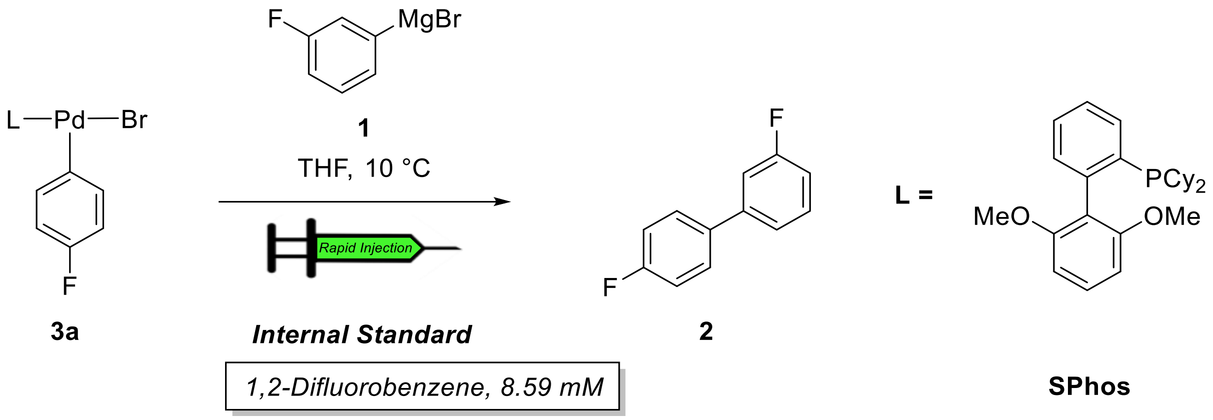

A 5-mL volumetic flask was charged with 1,2-Difluorobenzene (5.0 µL, 50.7 µmol) followed by dissolving with THF to the 5-mL mark generating a 0.01 M of stock solution. An oven-dried (150 °C), 5 mm, NMR tube was taken into the dry box and charged with **3a** (6.92 mg, 10 µmol) and 500 µL of the freshly prepared solution. The tube was capped with a septum. An oven-dried (60 °C) rapid injection barrel was taken into the glove box and charged with 3-Fluorophenylmagnesium bromide solution (500 µL, 1.33 M in THF). The glass capillary of the barrel was capped with a septum. The sample and the barrel were removed from the glove box and the sample was placed into the NMR probe set to 10 °C with the cap off. Then 3-Fluorophenylmagnesium bromide **1** (120 µmol) in THF (90 µL) was injected (RI-NMR).

Using the fluorine channel to collect a spectrum every 19 s the progress of the reaction was monitored by the formation of cross-coupling product in comparison with the internal reference 1,2-Difluorobenzene. The first order formation profile was fitted with the Curve Fitter Toolbox in Matlab using Equation 1.

Table S84. Summary of fits for the formation of **2**

**Run**	**R^2 of fit**	***k*** **($10^{-2}s^{-1}$) (form CCP)**	**$[OAC]_o$ (M)**
1	0.9852	1.16 ± 0.15	0.0166 ± 0.0009
2	0.9844	1.25 ± 0.31	0.0173 ± 0.0017

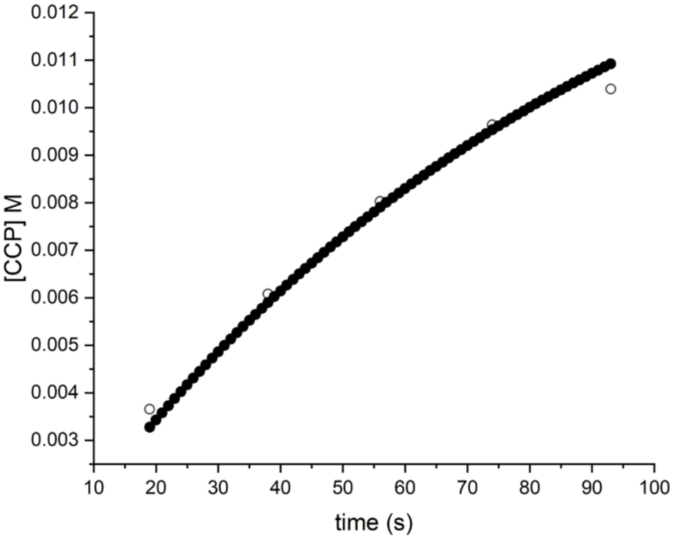

Figure S47. Formation of **2** from complex **3a** at 10 °C (Run 1)

Table S85. Data for the formation of **2** from complex **3a** at 10 °C (Run 1)

**Time (s)**	**Integral IS (−140 ppm)**	**Integral CCP (−116 ppm)**	**[CCP] (M)**
19	1731570	448823	0.004007
38	1696710	706061	0.006432
56	1724240	934151	0.008375
74	1704640	1101830	0.009991
93	1731620	1203280	0.010741

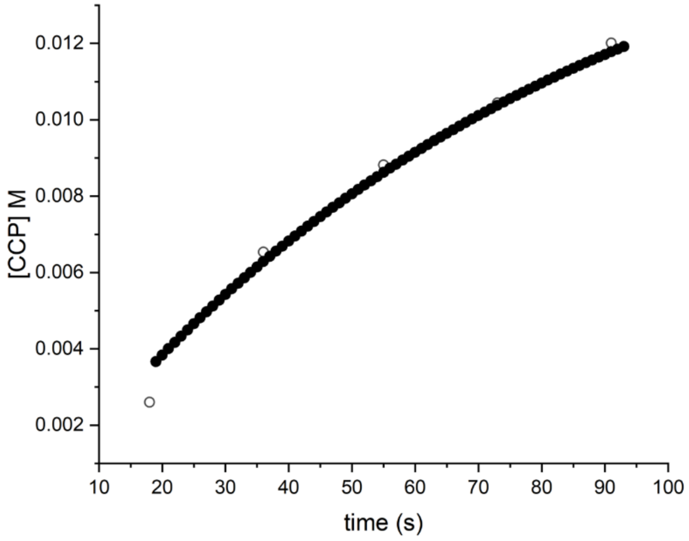

Figure S48. Formation of **2** from complex **3a** at 10 °C (Run 2)

Table S86. Data for the formation of **2** from complex **3a** at 10 °C (Run 2)

**Time (s)**	**Integral IS (−140 ppm)**	**Integral CCP (−116 ppm)**	**[CCP] (M)**
18	1811590	253618	0.002164
36	1699580	670144	0.006095
55	1715530	929986	0.00838
73	1715010	1109940	0.010004
91	1693170	1267440	0.011571

Experiment 23: Kinetic Study of the *trans*-[(4-F-C_6H_4)Pd(Ph_3P)$_2$Br] **(5a)** at 0 °C

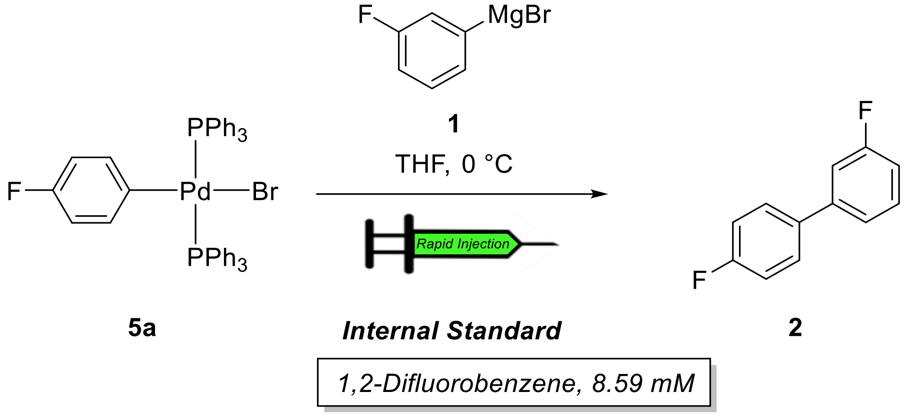

A 5-mL volumetic flask was charged with 1,2-Difluorobenzene (5.0 µL, 50.7 µmol) followed by dissolving with THF to the 5-mL mark generating a 0.01 M of stock solution. An oven-dried (150 °C), 5 mm, NMR tube was taken into the dry box and charged with **5a** (8.06 mg, 10 µmol) and 500 µL of the freshly prepared solution. The tube was capped with a septum. An oven-dried (60 °C) rapid injection barrel was taken into the glove box and charged with 3-Fluorophenylmagnesium bromide solution (500 µL, 1.33 M in THF). The glass capillary of the barrel was capped with a septum. The sample and the barrel were removed from the glove box and the sample was placed into the NMR probe set to 0 °C with the cap off. Then 3-Fluorophenylmagnesium bromide **1** (120 µmol) in THF (90 µL) was injected (RI-NMR).

Using the fluorine channel to collect a spectrum every 15 s the progress of the reaction was monitored by the formation of cross-coupling product in comparison with the internal reference 1,2-Difluorobenzene. The first order formation profile was fitted with the Curve Fitter Toolbox in Matlab using Equation 1.

Table S87. Summary of fits for the formation of **2**

**Run**	**R^2 of fit**	***k*** **($10^{-2}s^{-1}$) (form CCP)**	**$[OAC]_o$ (M)**
1	0.9916	0.32 ± 0.018	0.0163 ± 0.0003
2	0.9954	0.38 ± 0.016	0.0172 ± 0.0003

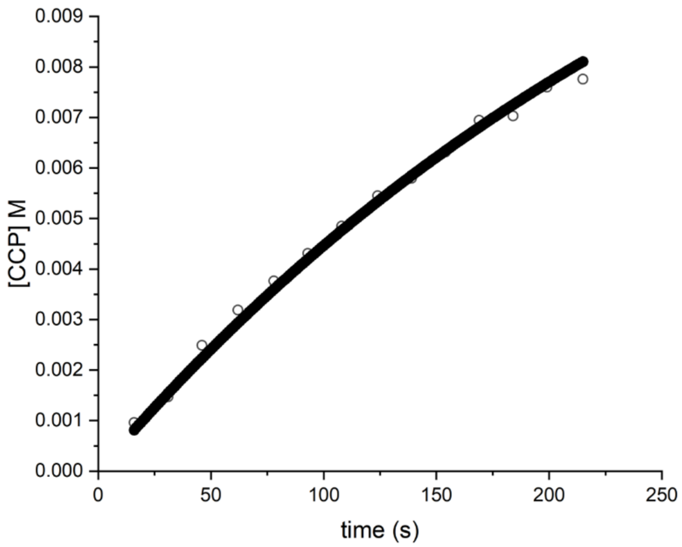

Figure S49. Formation of **2** from complex **5a** at 0 °C (Run 1)

Table S88. Data for the formation of **2** from complex **5a** at 0 °C (Run 1)

Time (s)	Integral IS (−140 ppm)	Integral CCP (−116 ppm)	[CCP] (M)
16	1909280	185861	0.001561
31	1927560	248705	0.002069
46	1931990	372232	0.003089
62	1890340	446531	0.003787
78	1938040	527612	0.004365
93	1921580	588174	0.004908
108	1907150	648260	0.00545
124	1927490	727422	0.006051
139	1933570	771641	0.006399
154	1949120	840747	0.006916
169	1962650	923141	0.007542
184	1923740	915548	0.007631
199	1899940	971979	0.008203
215	1897010	989102	0.00836

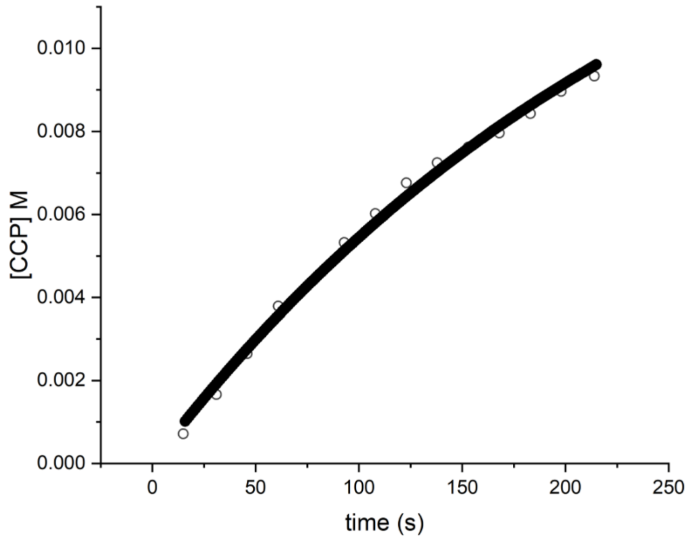

Figure S50. Formation of **2** from complex **5a** at 0 °C (Run 2)

Table S89. Data for the formation of **2** from complex **5a** at 0 °C (Run 2)

Time (s)	Integral IS (−140 ppm)	Integral CCP (−116 ppm)	[CCP] (M)
15	2006270	56571.3	0.000452
31	1925630	168157	0.0014
46	1860100	276404	0.002383
61	1859030	409163	0.003529
77	1865450	477999	0.004108
93	1949050	615152	0.005061
108	1870370	671797	0.005759
123	1880900	762443	0.0065
138	1871870	815437	0.006985
153	1897880	871489	0.007363
168	1921290	923117	0.007704
183	1874960	955417	0.00817
198	1889620	1025880	0.008705
214	1878240	1063050	0.009075

Experiment 24: Kinetic Study of the trans-[(4-F-C_6H_4)Pd(Ph_3P)$_2$Br] **(5a)** at 10 °C

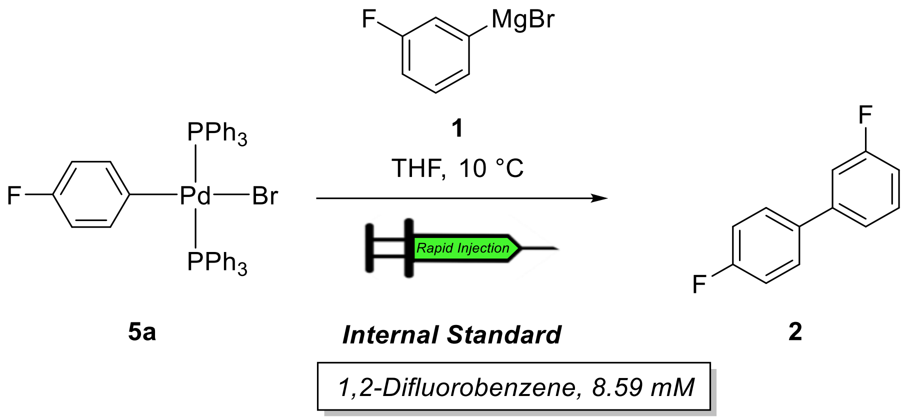

A 5-mL volumetic flask was charged with 1,2-Difluorobenzene (5.0 µL, 50.7 µmol) followed by dissolving with THF to the 5-mL mark generating a 0.01 M of stock solution. An oven-dried (150 °C), 5 mm, NMR tube was taken into the dry box and charged with **5a** (8.06 mg, 10 µmol) and 500 µL of the freshly prepared solution. The tube was capped with a septum. An oven-dried (60 °C) rapid injection barrel was taken into the glove box and charged with 3-Fluorophenylmagnesium bromide solution (500 µL, 1.33 M in THF). The glass capillary of the barrel was capped with a septum. The sample and the barrel were removed from the glove box and the sample was placed into the NMR probe set to 10 °C with the cap off. Then 3-Fluorophenylmagnesium bromide **1** (120 µmol) in THF (90 µL) was injected (RI-NMR).

Using the fluorine channel to collect a spectrum every 15 s the progress of the reaction was monitored by the formation of cross-coupling product in comparison with the internal reference 1,2-Difluorobenzene. The first order formation profile was fitted with the Curve Fitter Toolbox in Matlab using Equation 1.

Table S90. Summary of fits for the formation of **2**

Run	R^2 of fit	k ($10^{-2}s^{-1}$) (form CCP)	[OAC]$_o$ (M)
1	0.9763	0.93 ± 0.209	0.0172 ± 0.0013
2	0.9922	0.86 ± 0.108	0.0168 ± 0.0007

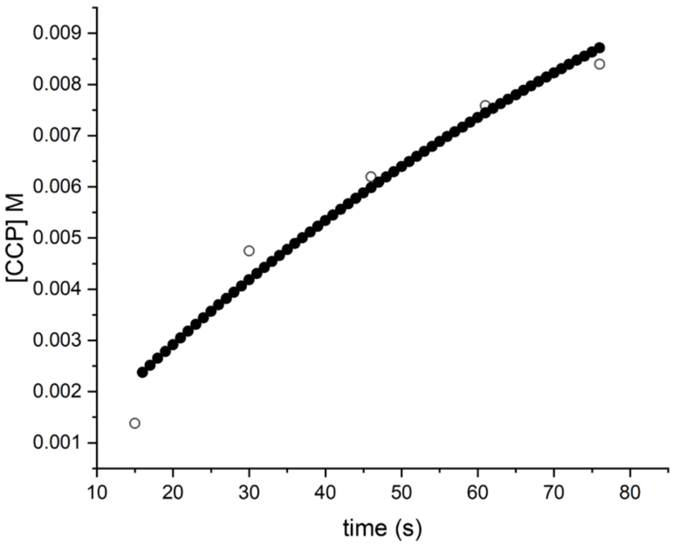

Figure S51. Formation of **2** from complex **5a** at 10 °C (Run 1)

Table S91. Data for the formation of **2** from complex **5a** at 10 °C (Run 1)

**Time (s)**	**Integral IS (−140 ppm)**	**Integral CCP (−116 ppm)**	**[CCP] (M)**
15	1739000	119449	0.001101
30	1687630	470352	0.004469
46	1634790	603021	0.005914
61	1670390	761036	0.007305
76	1666230	843170	0.008114

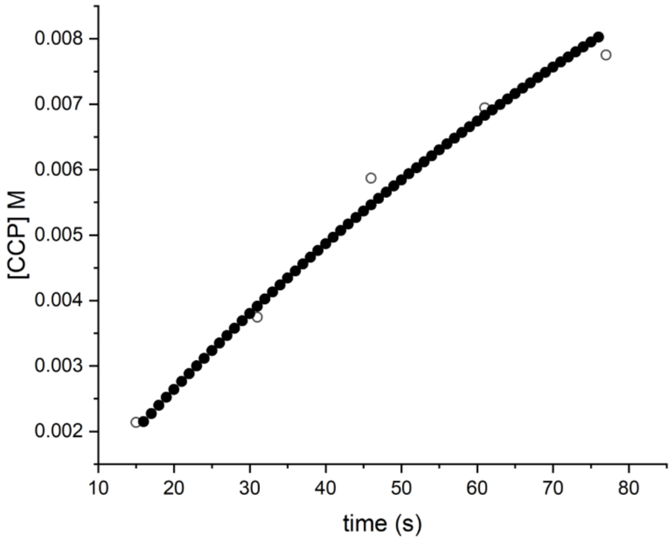

Figure S52. Formation of **2** from complex **5a** at 10 °C (Run 2)

Table S92 Data for the formation of **2** from complex **5a** at 10 °C (Run 2)

**Time (s)**	**Integral IS (−140 ppm)**	**Integral CCP (−116 ppm)**	**[CCP] (M)**
15	1626580	230081	0.002268
31	1628660	393770	0.003877
46	1611010	602828	0.006
61	1590830	701723	0.007073
77	1601800	787467	0.007882

Experiment 25: Kinetic Study of the (CPhos)Pd[(4-F)Ph]Br **(14a)** at –20 °C

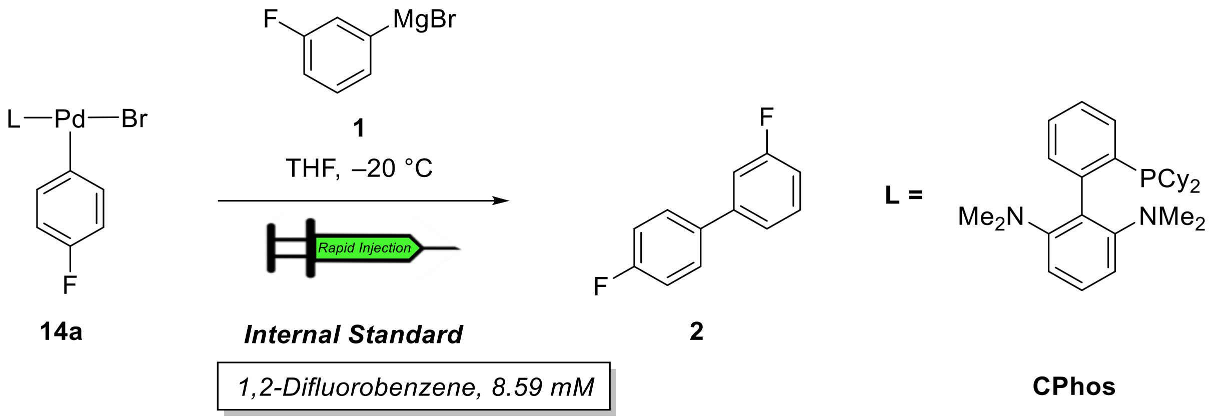

A 5-mL volumetic flask was charged with 1,2-Difluorobenzene (5.0 µL, 50.7 µmol) followed by dissolving with THF to the 5-mL mark generating a 0.01 M of stock solution. An oven-dried (150 °C), 5 mm, NMR tube was taken into the dry box and charged with **14a** (7.18 mg, 10 µmol) and 500 µL of the freshly prepared solution. The tube was capped with a septum. An oven-dried (60 °C) rapid injection barrel was taken into the glove box and charged with 3-Fluorophenylmagnesium bromide solution (500 µL, 1.33 M in THF). The glass capillary of the barrel was capped with a septum. The sample and the barrel were removed from the glove box and the sample was placed into the NMR probe set to –20 °C with the cap off. Then 3-Fluorophenylmagnesium bromide **1** (120 µmol) in THF (90 µL) was injected (RI-NMR).

Using the fluorine channel to collect a spectrum every 1.61 s the progress of the reaction was monitored by the formation of cross-coupling product in comparison with the internal reference 1,2-Difluorobenzene. The first order formation profile was fitted with the Curve Fitter Toolbox in Matlab using Equation 1.

Table S93. Summary of fits for the formation of **2**

**Run**	**R^2 of fit**	***k*** **($10^{-2}s^{-1}$) (form CCP)**	**$[OAC]_o$ (M)**
1	0.9619	26.99 ± 9.23	0.0160 ± 0.0029
2	0.9883	30.66 ± 7.65	0.0167 ± 0.0021

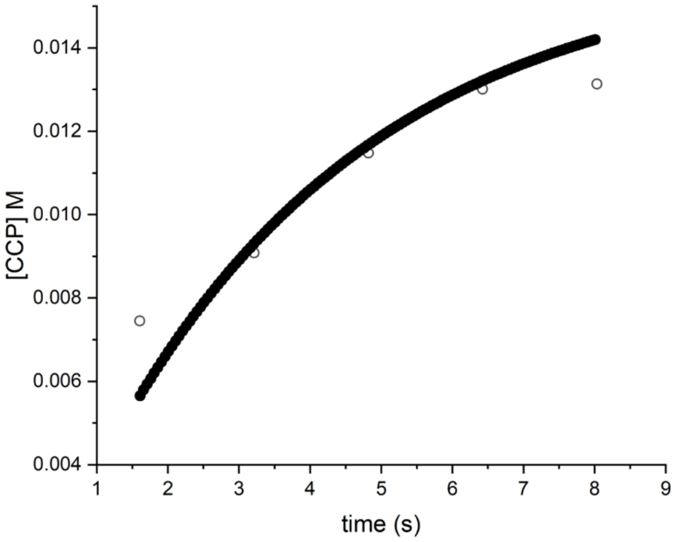

Figure S53. Formation of **2** from complex **14a** at –20 °C (Run 1)

Table S94 Data for the formation of **2** from complex **14a** at –20 °C (Run 1)

Time (s)	Integral IS (−140 ppm)	Integral CCP (−116 ppm)	[CCP] (M)
1.61	276707	169593	0.00831
3.21	340664	249649	0.009937
4.82	334007	303894	0.012337
6.43	325221	332578	0.013866
8.03	327584	338049	0.013992

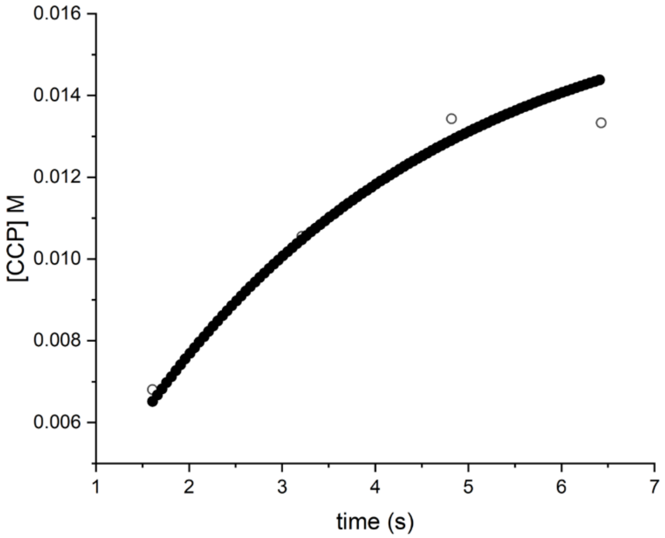

Figure S54. Formation of **2** from complex **14a** at –20 °C (Run 2)

Table S95. Data for the formation of **2** from complex **14a** at –20 °C (Run 2)

Time (s)	Integral IS (−140 ppm)	Integral CCP (−116 ppm)	[CCP] (M)
1.61	359363	185171	0.006987
3.21	332458	263193	0.010734
4.82	303190	304271	0.013608
6.43	303052	302003	0.013512

2.5. Catalyst Transfer Polymerization (CTP)

Preparation for Thiophene Monomer: Grignard Metathesis Method (GRIM)

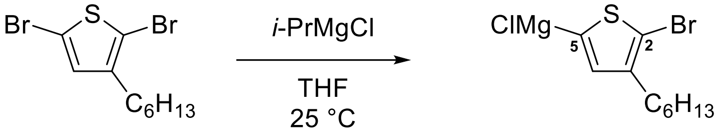

S4 **S5**

MgCl at **C5** : MgCl at **C2** = 4:1
determined by ^{1}H NMR

In the glovebox, **S4** (0.28 mL, 1.30 mmol, 1.0 equiv.) was dissolved in THF (3.5 mL) in a 20 mL vial containing a magnetic stir bar. *i*-PrMgCl (0.62 mL, 1.20 mmol, 1.94 M, 0.9 equiv) was then added via syringe, the vial was capped, and the reaction mixture was stirred for at 25 °C 30 min.

Titration method: In a 1-dram vial equipped with magnetic stir bar, salicylaldehyde phenylhydrazone (10.0 mg, 0.047 mmol) was dissolved in 0.5 mL of THF. ArMgCl was added dropwise using a 500 μL syringe. The initially yellow solution turned bright orange indicating the end-point. The average concentration of ArMgCl was determined to be 0.29 M.[vii]

General Method of CTP: Synthesis of poly(3-hexylthiophene), P3HT (For Table S96)

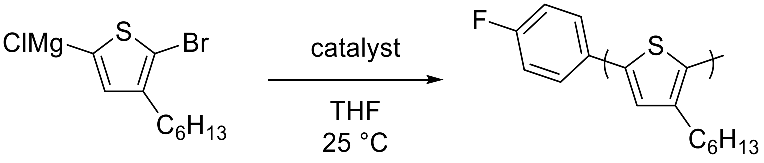

S5 **S6**

Inside the glovebox, a 25 mL oven-dried (150 °C) Schlenk flask equipped with a magnetic stir bar was charged with precatalyst (10.8 mg, 0.015 mmol, 1.0 equiv) and THF (6.86 mL), then capped with rubber septum. The flask was removed from the glovebox and connected to a the Schlenk line under argon flow. **S5** (3.14 mL, 0.287 M, 0.9 mmol, 60 equiv) was added via syringe, and stirred for at 25 °C 90 min. The reaction was quenched with aq. HCl (5 M, 10 mL), extracted with $CHCl_3$ (3 × 20 mL), dried over Na_2SO_4, filtered, and the solvent was removed under reduced pressure. The resulting purple solid was dissolved in a minimal amount of $CHCl_3$ and precipitated into 250 mL beaker containing MeOH (200 mL) under vigorous stirring (600 rpm). The precipitate was collected and dried under vacuum overnight yielding P3HT **S6**.

SEC sample preparation: 5~10 mg of polymer sample was dissolved in THF (HPLC grade, 1.5 mL) and allowed to sit for 6 hours to ensure polymer chain disentanglement. Just before filtration, the solution was gently heated using a heat gun, then filtered through a 0.2 μm PTFE filter into a sample vial for SEC analysis.

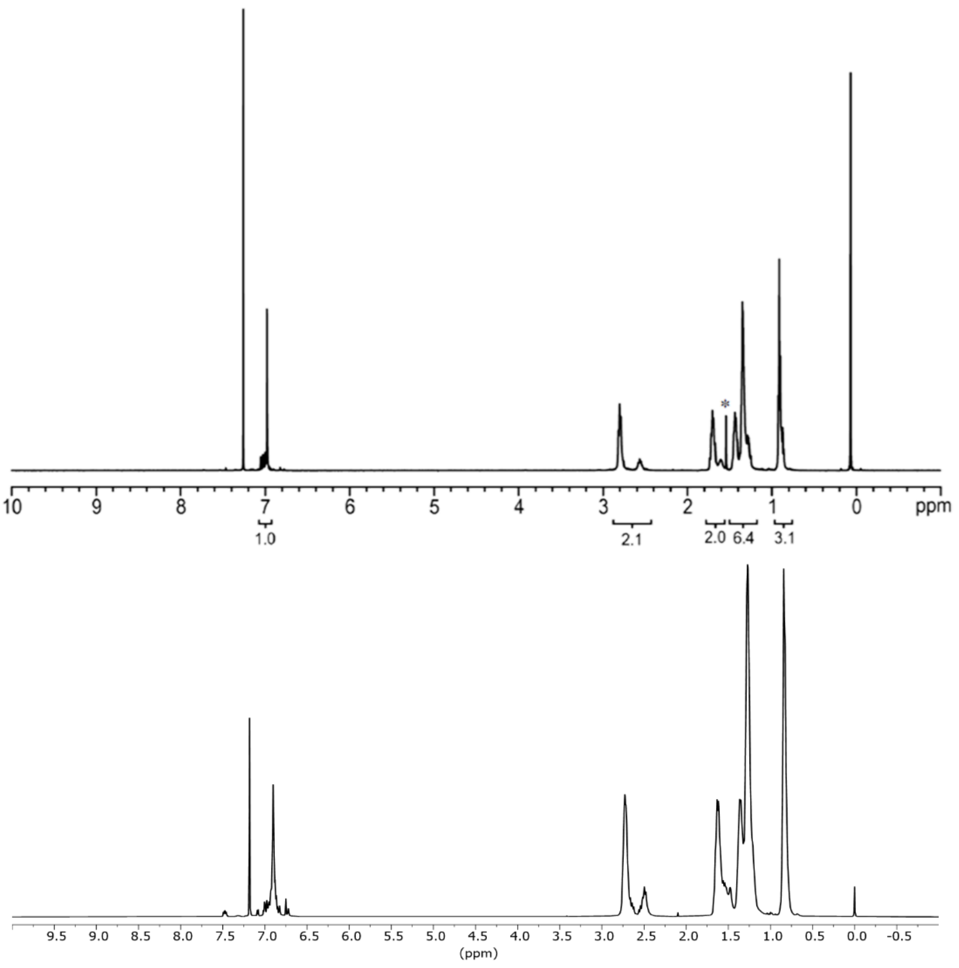

Figure S55. Comparison of the ^1H NMR spectra of synthesized P3HT (bottom) and reported P3HT (top).[7a]

Catalyst Transfer Polymerization: Effect of k_{obs} on M_n and dispersity

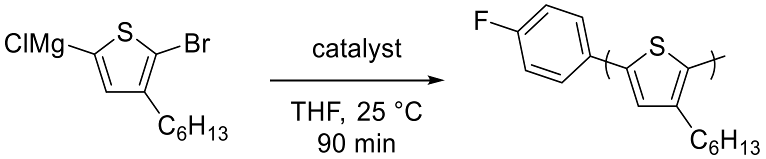

S5 **S6**

Table S96. Ligand and Halide Effect on Catalyst Transfer Polymerization

Entry	Catalyst	form k_{obs}[b], 10^{-2} s^{-1}	M/I	M_n ($Đ$)	dispersity
1	**3a**	0.041 ± 0.003	60	5.9 k	1.57
2	**5a**	0.12 ± 0.005	60	-	
3	**6a**	1.63 ± 0.26	60	-	
4	**8a**	6.22 ± 0.66	60	2.9 k	1.61
5	**8b**	12.69 ± 1.03	60	3.1 k	1.63
6	**11c**	4.50 ± 1.13	60	8.8 k	1.62
7	**11a**	11.79 ± 0.40	60	10.8 k	1.42
8	**11b**	19.61 ± 1.02	60	12.9 k	1.47
9	**12a**	14.76 ± 0.28	60	1.5 k	1.20
10	**14a**	38.07 ± 6.46	60	13.9 k	1.37
11[a]	**14a**	38.07 ± 6.46	60	14.1 k	1.43
12[a]	**14a**	38.07 ± 6.46	120	14.0 k	1.58
13	PEPPSI-Ipr	-	60	17.0 k	1.25

Reaction time was 15 h. [b] k_{obs} values were obtained in rapid injection experiments.

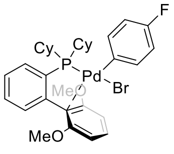

3a

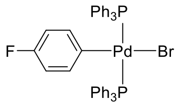

5a

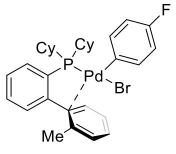

6a

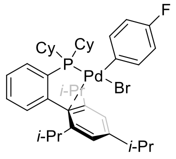

8a

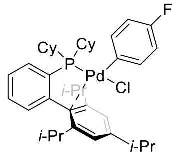

8b

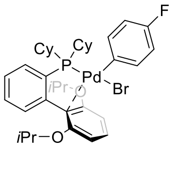

11a

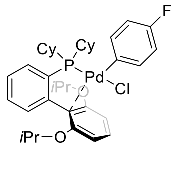

11b

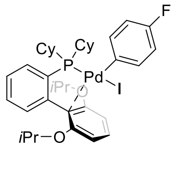

11c

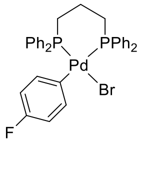

12a

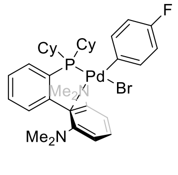

14a

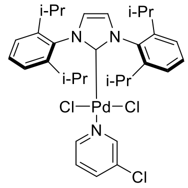

PEPPSI-Ipr

Screening of the Ring-walking Process in Small Molecule:

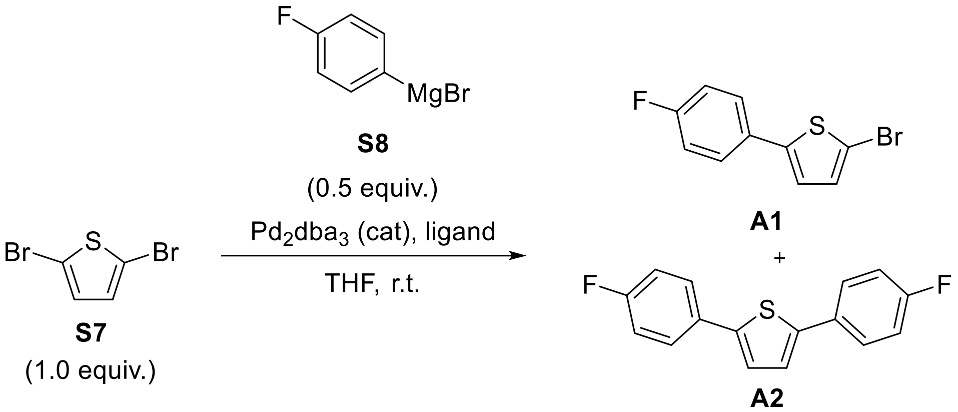

To a dram vial equipped with a magnetic stir bar, 2,5-dibromothiophene **S7** (48.4 mg, 0.2 mmol, 1.0 equiv), ligand (0.016 mmol), Pd_2dba_3 (0.004 mmol, 3.66 mg) were added inside the argon-filled glove box, followed by addition of THF (Nak, degassed, 3.0 mL). The mixture was heated and stirred at 60 °C for 1 h, then cooled to room temperature. Afterward, 4-fluorophenylmagnesium bromide **S8** (0.1 mmol, 1.26 M, 79 μL) was then added to the reaction mixture, and the reaction was left to stir at room temperature overnight. The crude reaction mixture was taken out from the glove box and quenched by 2.0 mL brine. Crude mixture was extracted by Et_2O (3 x 5 mL) then dried under reduced pressure. Internal standard, 1,4-difluorobenzene, from stock solution with $CDCl_3$ was added to analyze ^{19}F NMR yield.

Table S97. Product distribution experiment.

Entry	Pd (equiv)	ligand (equiv)	**A1:A2**[a]
1	Pd_2dba_3 (0.02)	SPhos (0.08)	6:94
2	Pd_2dba_3 (0.02)	XPhos (0.08)	15:85
3	Pd_2dba_3 (0.02)	RuPhos (0.08)	3:97
4	Pd_2dba_3 (0.02)	dppp (0.08)	96:4
5	Pd_2dba_3 (0.02)	CPhos (0.08)	9:91

[a]**A1:A2** ratio was determined by ^{19}F NMR spectroscopy against 1,4-difluorobenzene as an internal standard

Chain Extension Experiment:

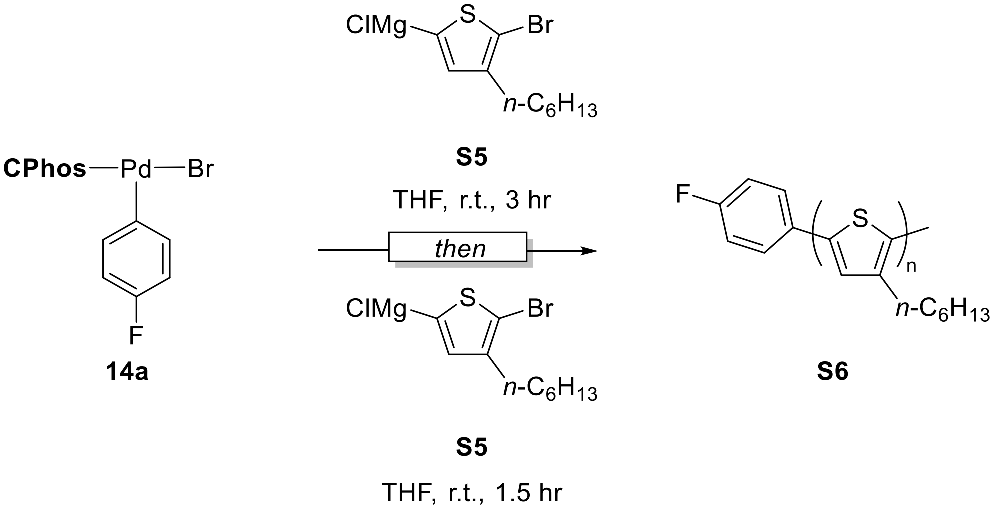

Inside the glovebox, a 25 mL oven-dried (150 °C) Schlenk flask equipped with a magnetic stir bar was charged with **14a** (10.8 mg, 0.015 mmol, 1.0 equiv) and THF (6.0 mL), then capped with rubber septum. The flask was removed from the glovebox and connected to a the Schlenk line under argon flow. **S5** (2.0 mL, 0.186 M, 0.37 mmol, 25.0 equiv) was added via syringe, and stirred for at 25 °C 3 hours. After 3 hours, an aliquot was taken via syringe and quenched with a. HCl (12 M, 1.0 mL). **S5** (1.5 mL, 0.246 M, 0.37 mmol, 25.0 equiv) was then added and stirred for 1.5 hours at 25 °C. After 60 min, an aliquot was taken and quenched with aq. HCl (12 M, 1 mL). Each aliquot was extracted with $CHCl_3$ (3 × 1 mL), dried over Na_2SO_4, filtered, and concentrated under reduced pressure.

SEC sample preparation: Each aliquot was dissolved in THF (HPLC grade, 1.5 mL) and allowed to sit for 6 hours to ensure polymer chain disentanglement. Just before filtration, the solution was gently heated using a heat gun, then filtered through a 0.2 µm PTFE filter into a sample vial for SEC analysis.

Block 1: M_n = 5.5 k, dispersity = 1.19 / Block 2: M_n = 8.1 k, dispersity = 1.32

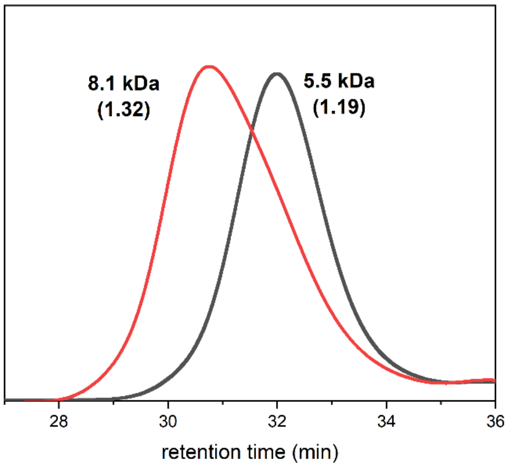

Figure S56. chain extension of P3HT

Plot of M_n and dispersity versus conversion and plot of ln([M]$_0$/[M]) versus time:

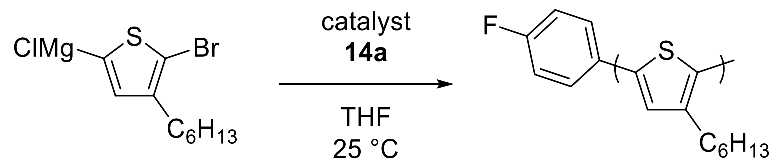

S5 **S6**

Inside the glovebox, a 25 mL oven-dried (150 °C) Schlenk flask equipped with a magnetic stir bar was charged with precatalyst **14a** (10.8 mg, 0.015 mmol, 1.0 equiv) and THF (6.56 mL), then capped with rubber septum. The flask was removed from the glovebox and connected to a the Schlenk line under argon flow. **S5** (3.44 mL, 0.262 M, 0.9 mmol, 60 equiv), with tridecane added as an internal standard was added via syringe, and allowed to stir for at 25 °C. The aliquots were taken every 10 min and quenched with aq. HCl (12 M, 1.0 ml). Each aliquot was extracted with Et_2O (3 × 1.0 mL) and $CHCl_3$ (3 × 1.0 mL), dried over Na_2SO_4, filtered. The Et_2O layer was directly used for GC-FID analysis to get monomer conversion, whereas the $CHCl_3$ layer was dried under reduced pressure to be used for SEC analysis.

SEC sample preparation: The resulting polymer sample in each aliquot was dissolved in THF (HPLC grade, 1.5 mL) and allowed to sit for 6 hours to ensure polymer chain disentanglement. Just before filtration, the solution was gently heated using a heat gun, then filtered through a 0.2 µm PTFE filter into a sample vial for SEC analysis.

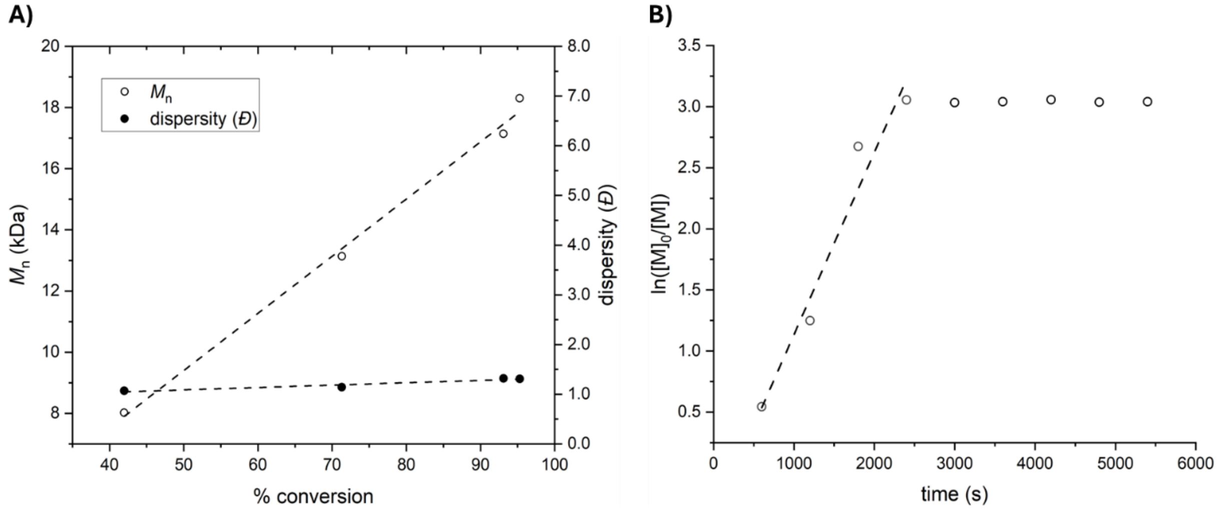

Figure S57. Plot of A) M_n and dispersity versus conversion of monomer and B) logarithm of [M]$_0$/[M] versus time.

Table S98. Data for Figure S56.

Time (s)	Monomer conversion (%)	M_n (kDa)	Dispersity (*Đ*)	ln([M]$_0$/[M])
0	0	-	-	0
600	42	8.023	1.07	0.54
1200	71	13.1	1.14	1.25
1800	93	17.1	1.32	2.67
2400	95	18.3	1.31	3.06
3000	95	17.8	1.32	3.03
3600	95	18.3	1.31	3.04
4200	95	-	-	3.06
4800	95	-	-	3.04
5400	95	-	-	3.04

3. NMR Spectra

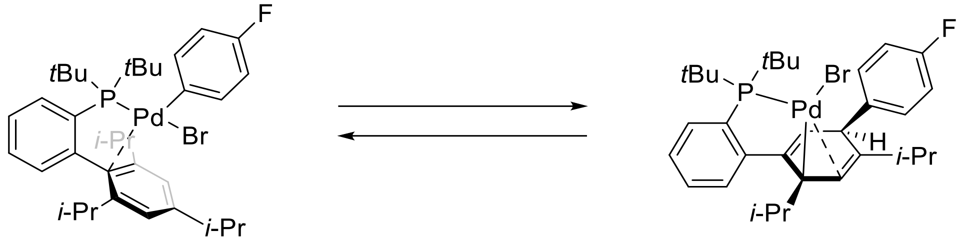

4a

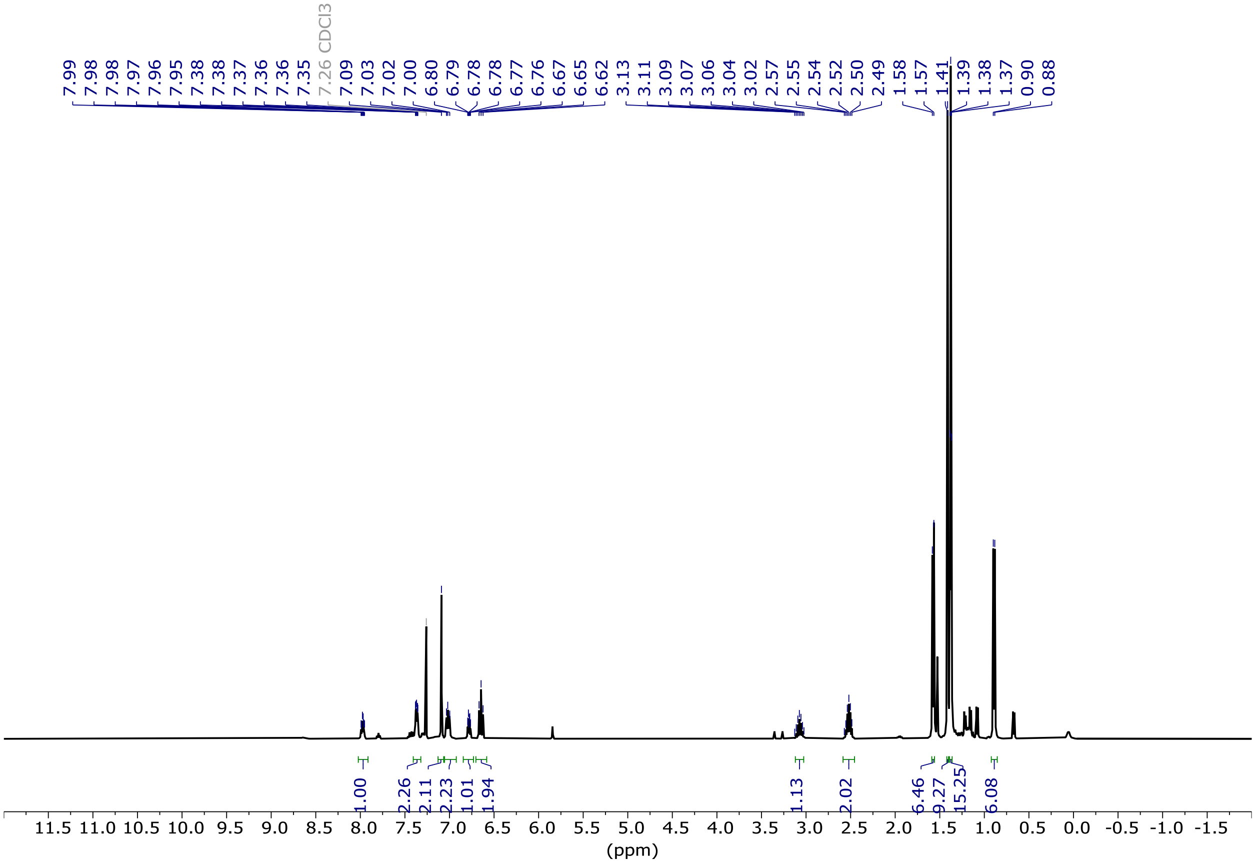

Figure S58. ^{1}H NMR spectrum of **4a** and isomer, referenced to $CDCl_3$ (7.26 ppm).

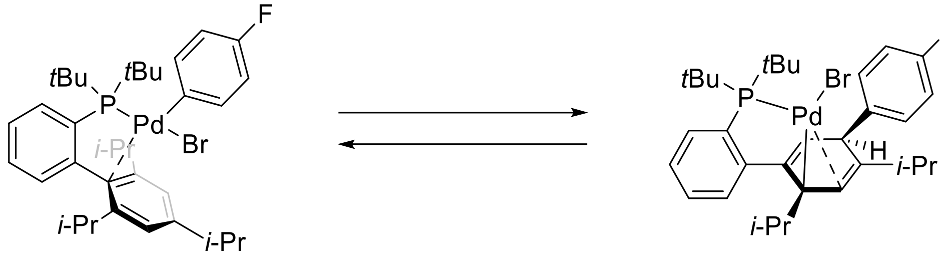

4a

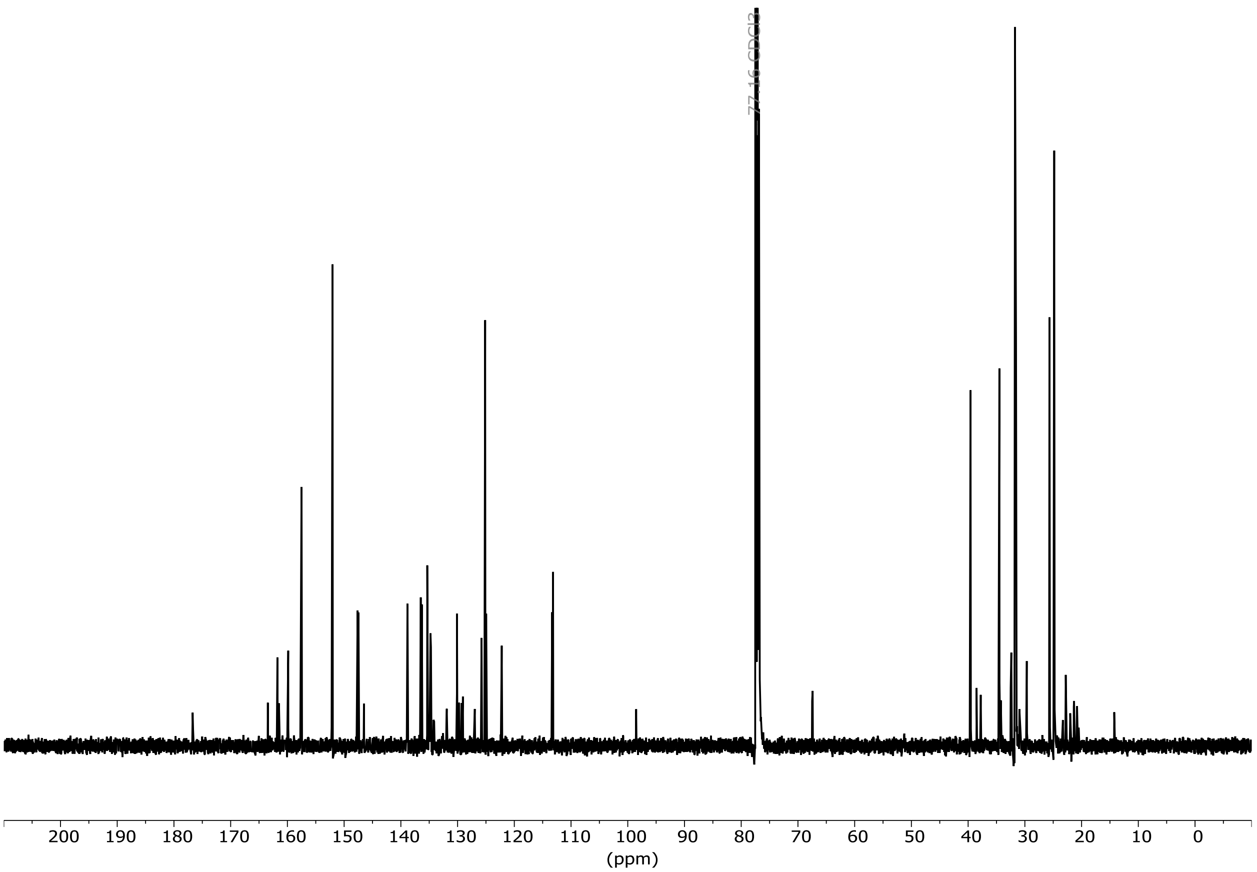

Figure S59. ^{13}C NMR spectrum of **4a** and isomer, referenced to $CDCl_3$ (77.16 ppm).

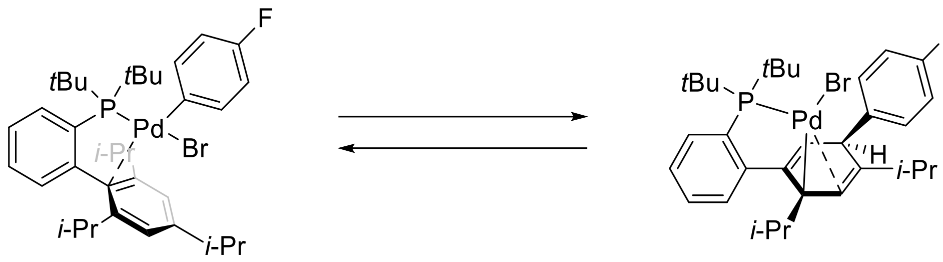

4a

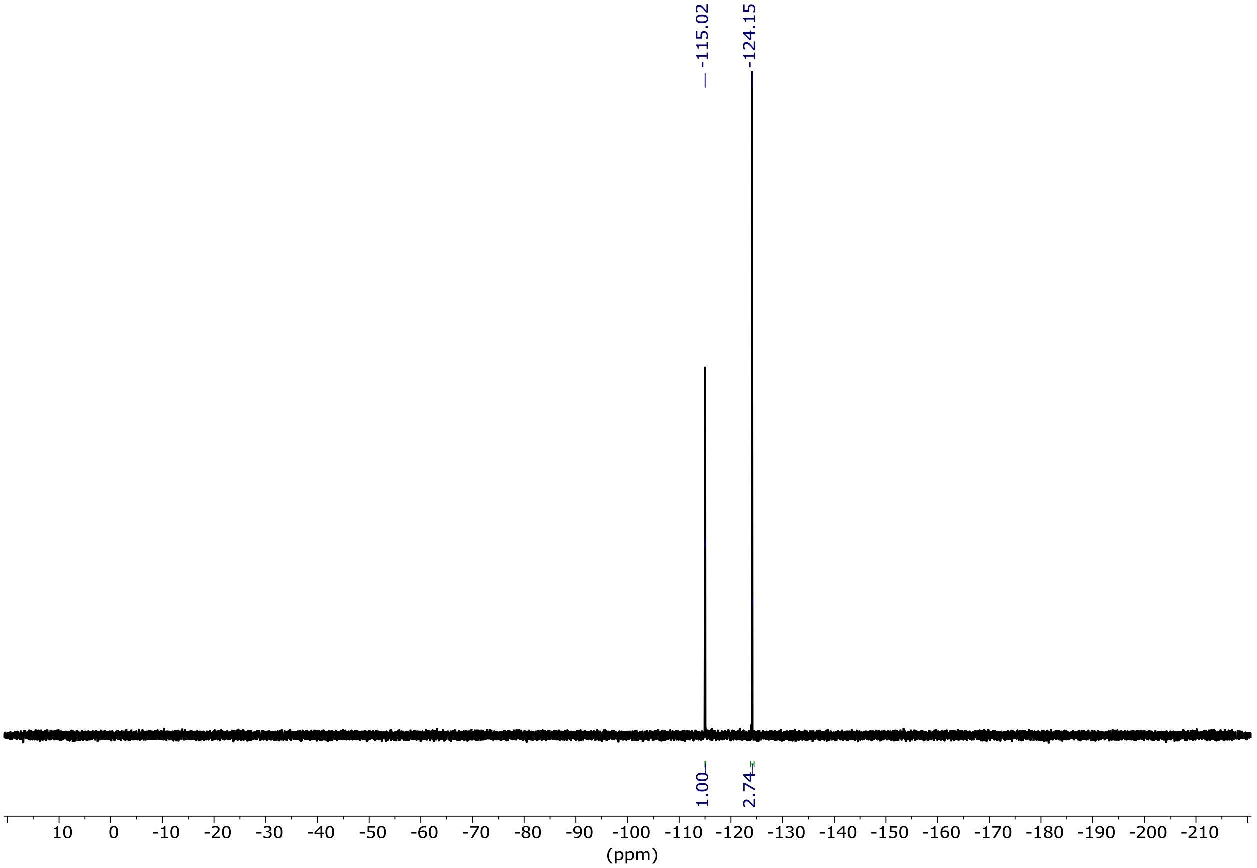

Figure S60. ^{19}F NMR spectrum of **4a** and isomer, externally referenced to fluorobenzene (-113.15 ppm).

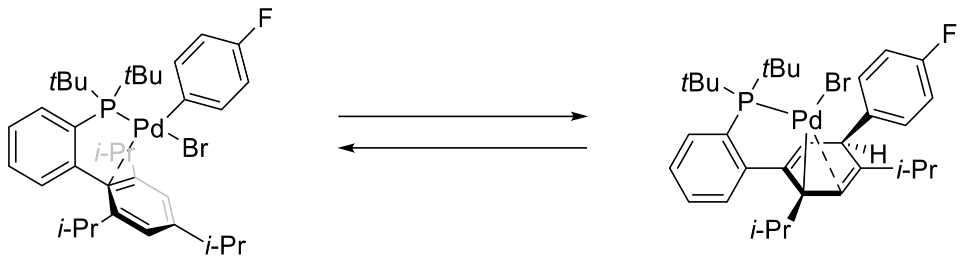

4a

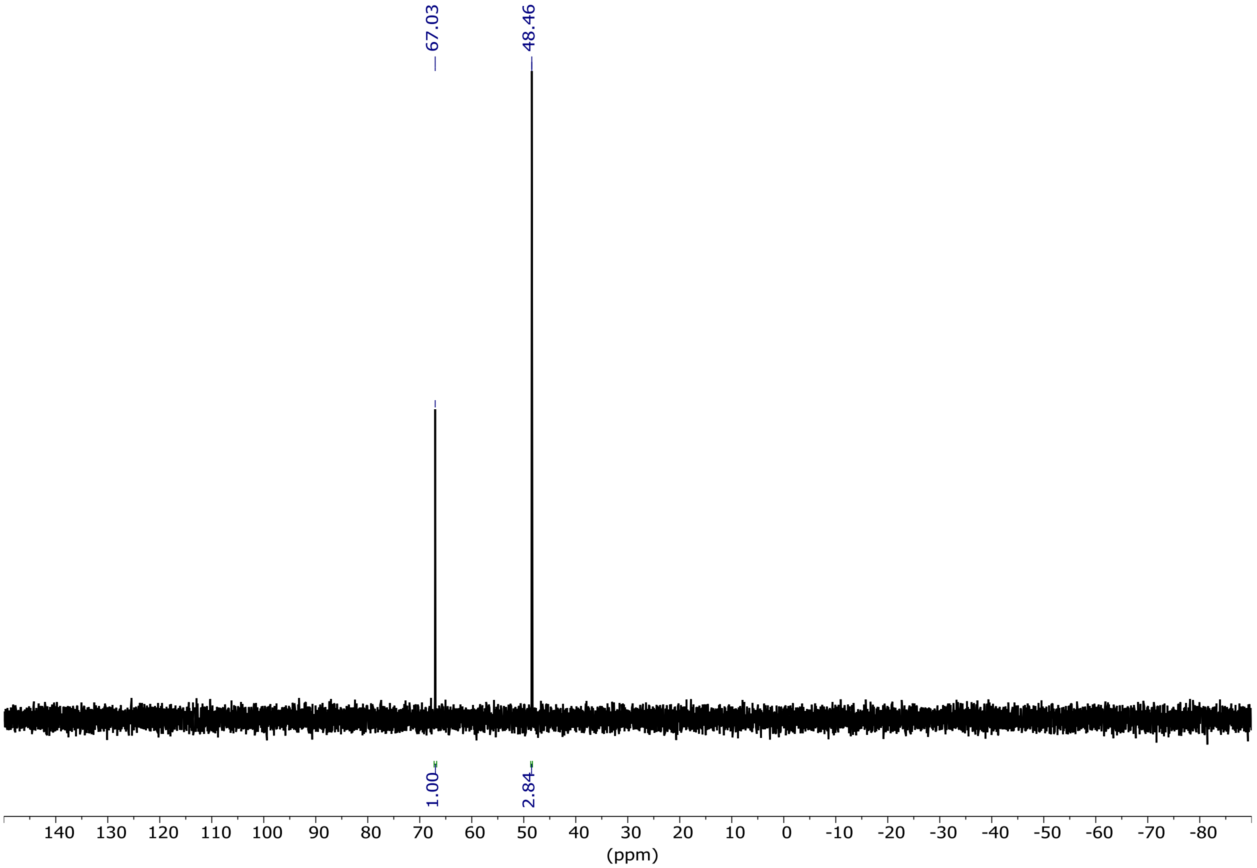

Figure S61. ^{31}P NMR spectrum of **4a** and isomer, externally referenced to triphenylphosphine (–6.5 ppm).

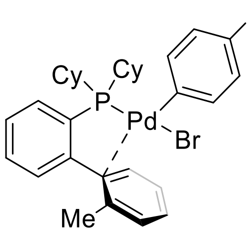

6a

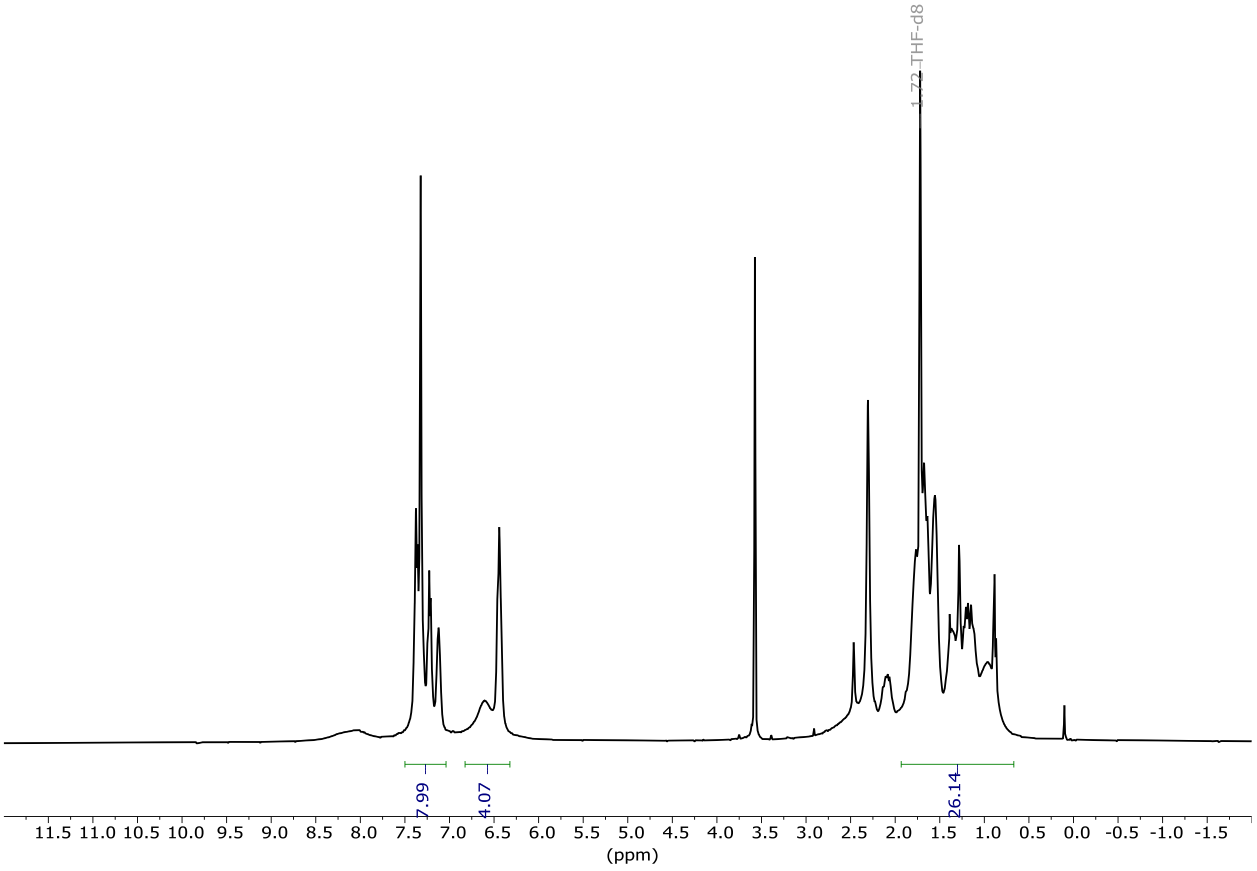

Figure S62. ^{1}H NMR spectrum of **6a**, referenced to THF-d_8 (1.72 ppm).

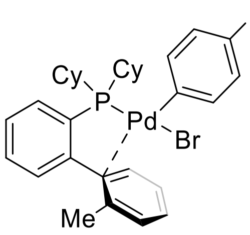

6a

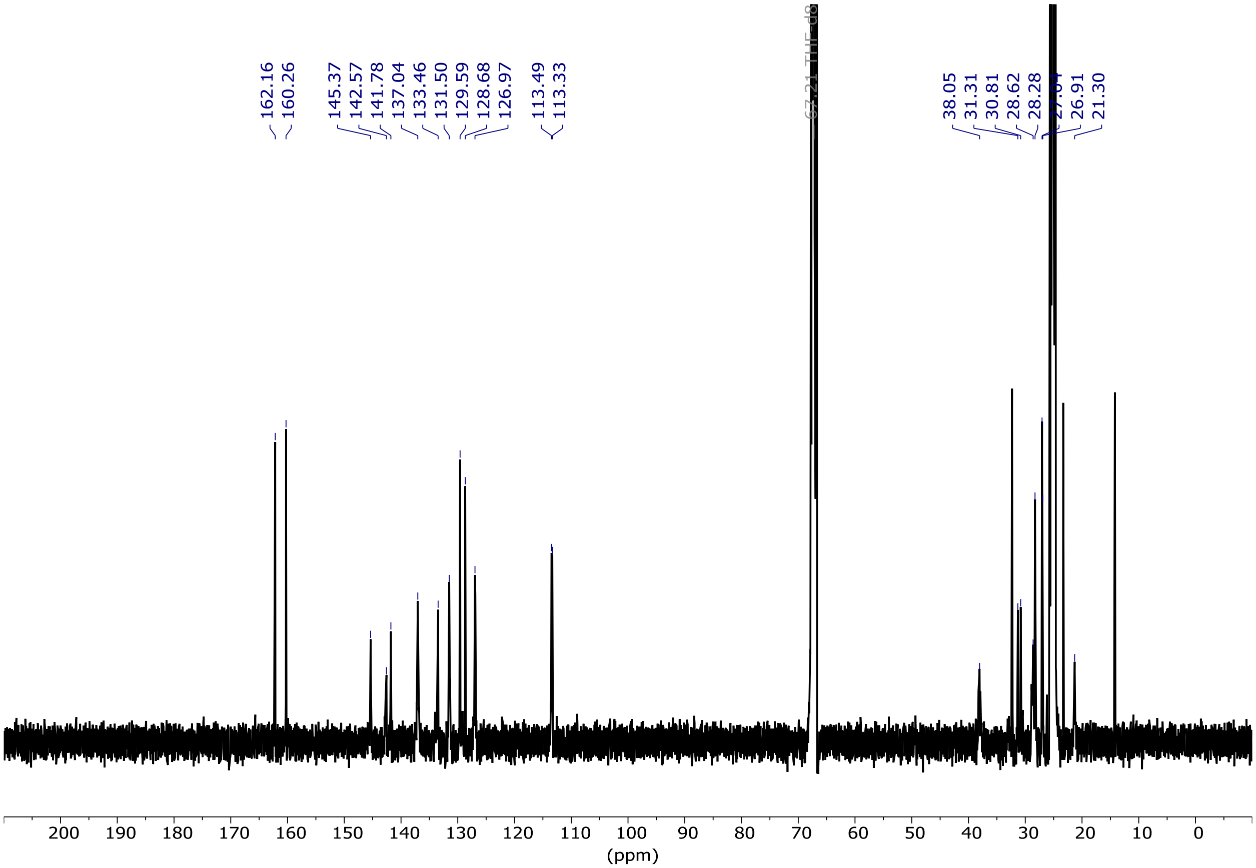

Figure S63. ^{13}C NMR spectrum of **6a**, referenced to THF-d_8 (67.21 ppm).

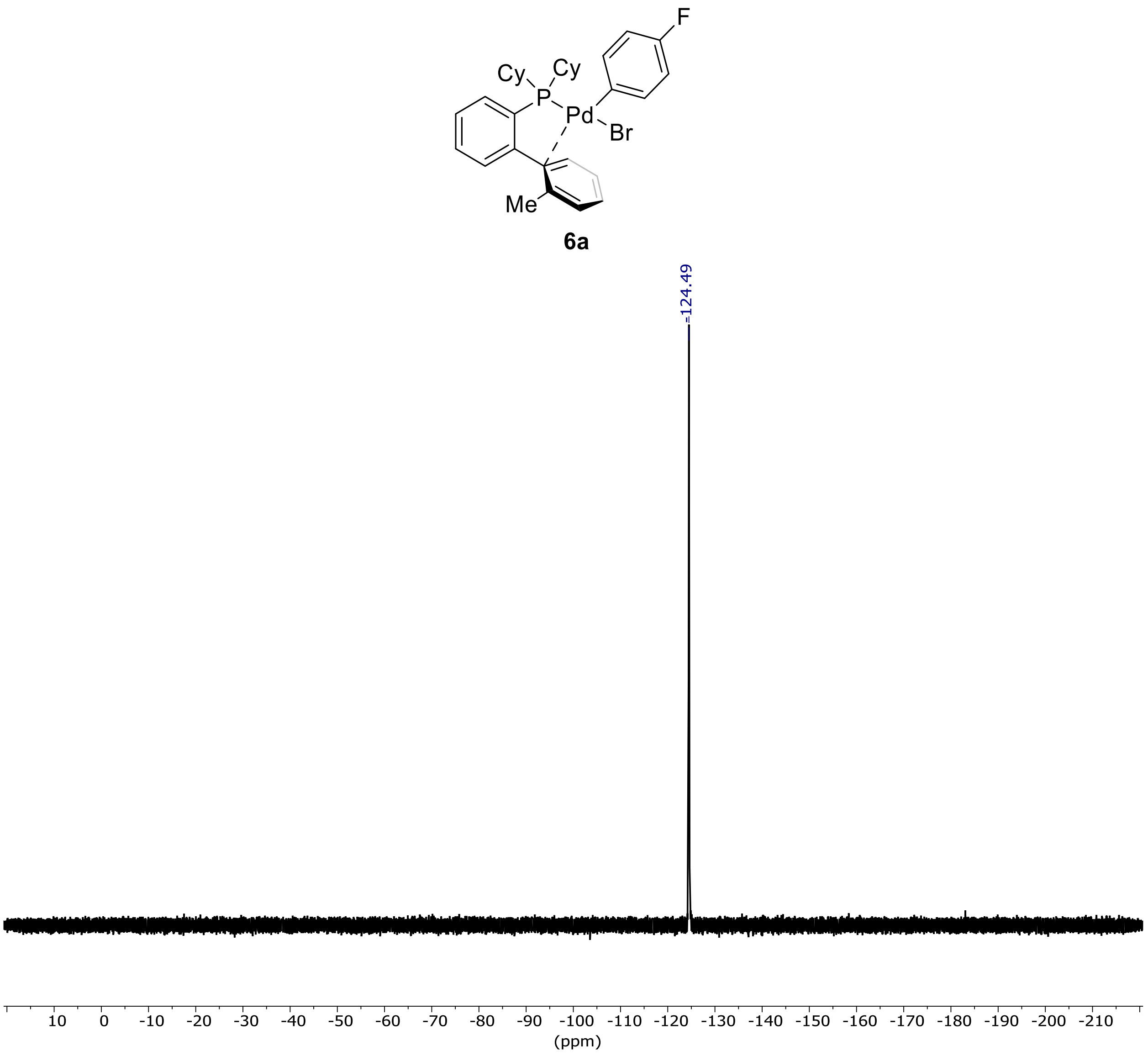

Figure S64. ^{19}F NMR spectrum of **6a**, externally referenced to fluorobenzene (-113.15 ppm).

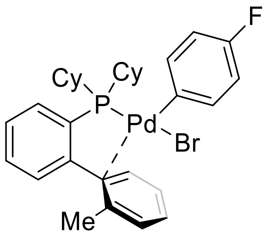

6a

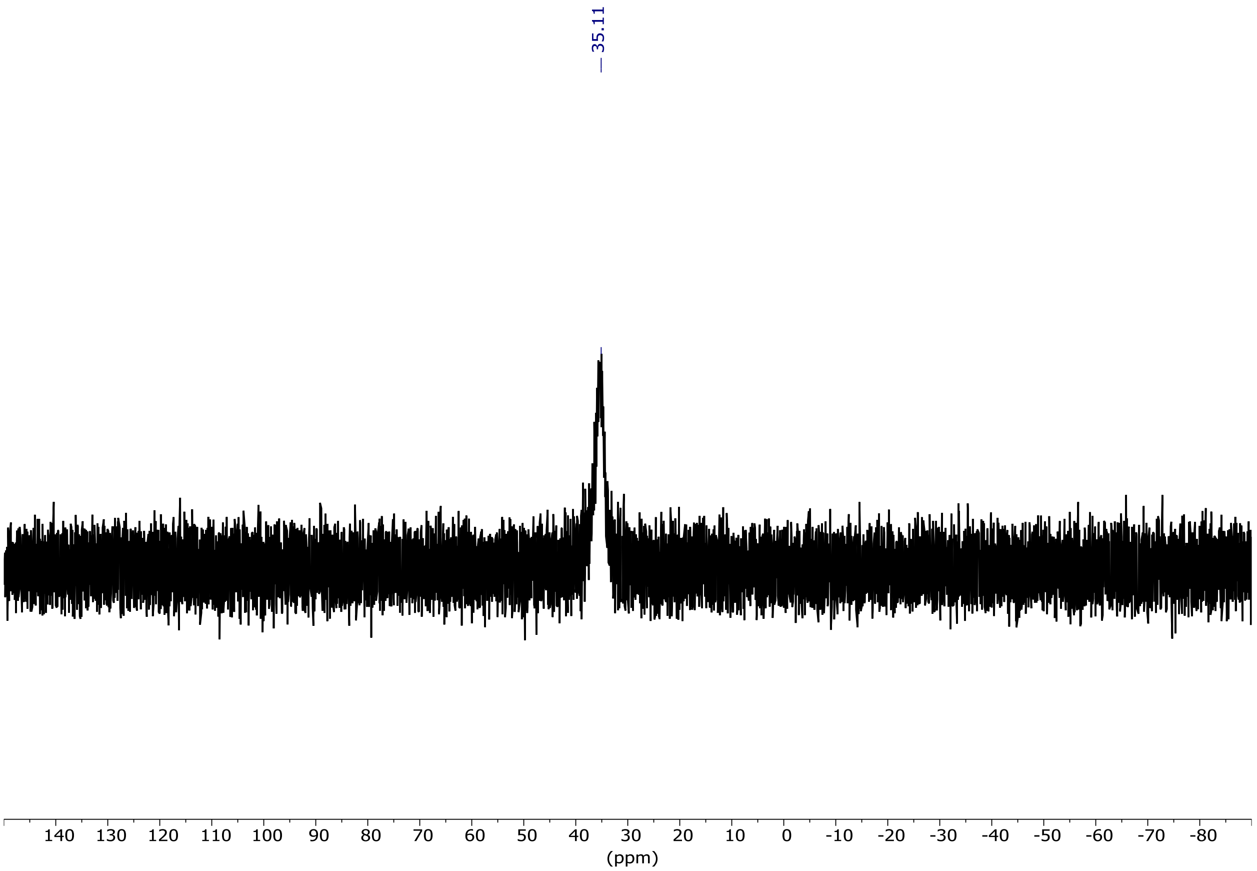

Figure S65. ^{31}P NMR spectrum of **6a**, externally referenced to triphenylphosphine (– 6.5 ppm).

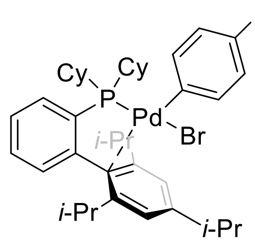

8a

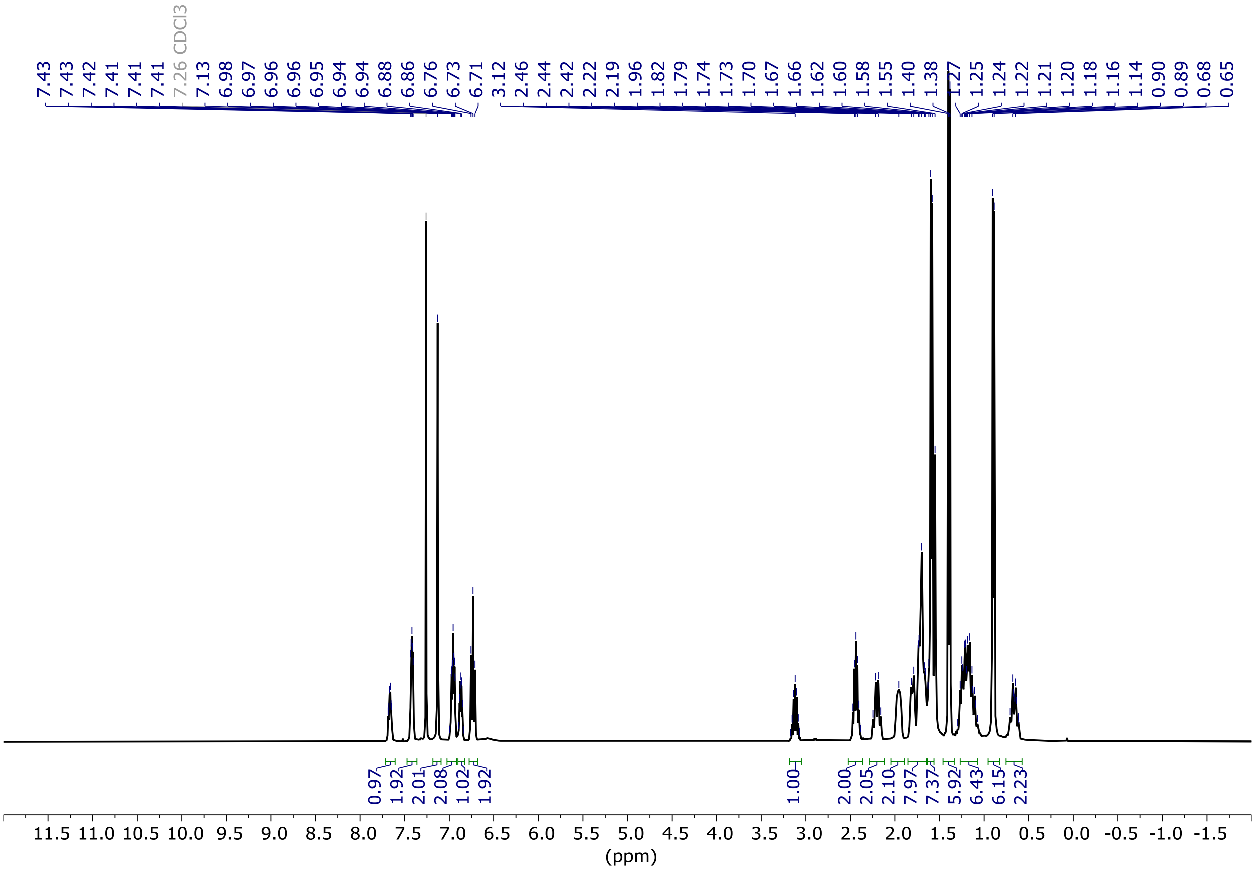

Figure S66. ^{1}H NMR spectrum of **8a**, referenced to $CDCl_3$ (7.26 ppm).

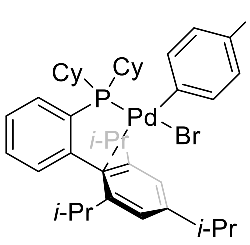

8a

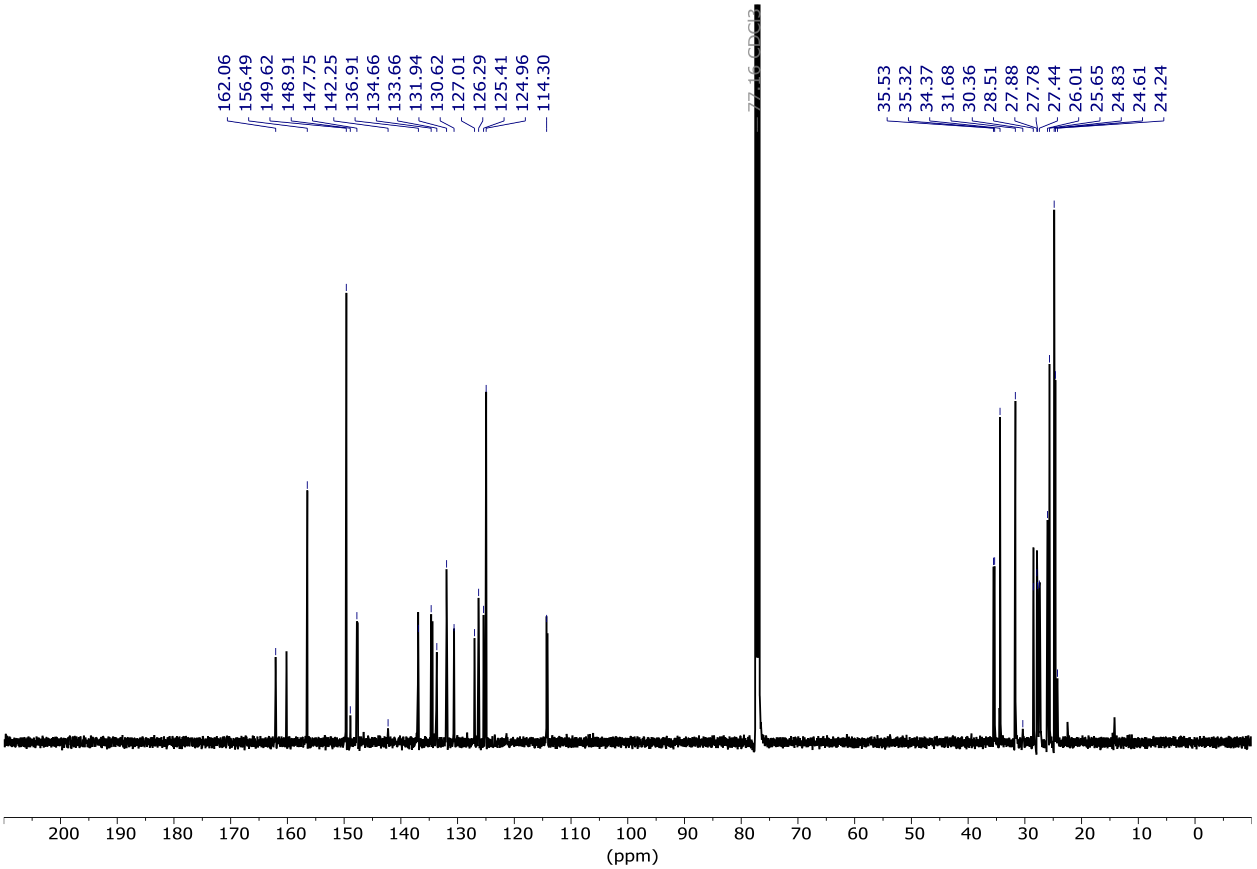

Figure S67. ^{13}C NMR spectrum of **8a**, referenced to $CDCl_3$ (77.16 ppm).

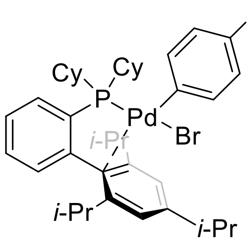

8a

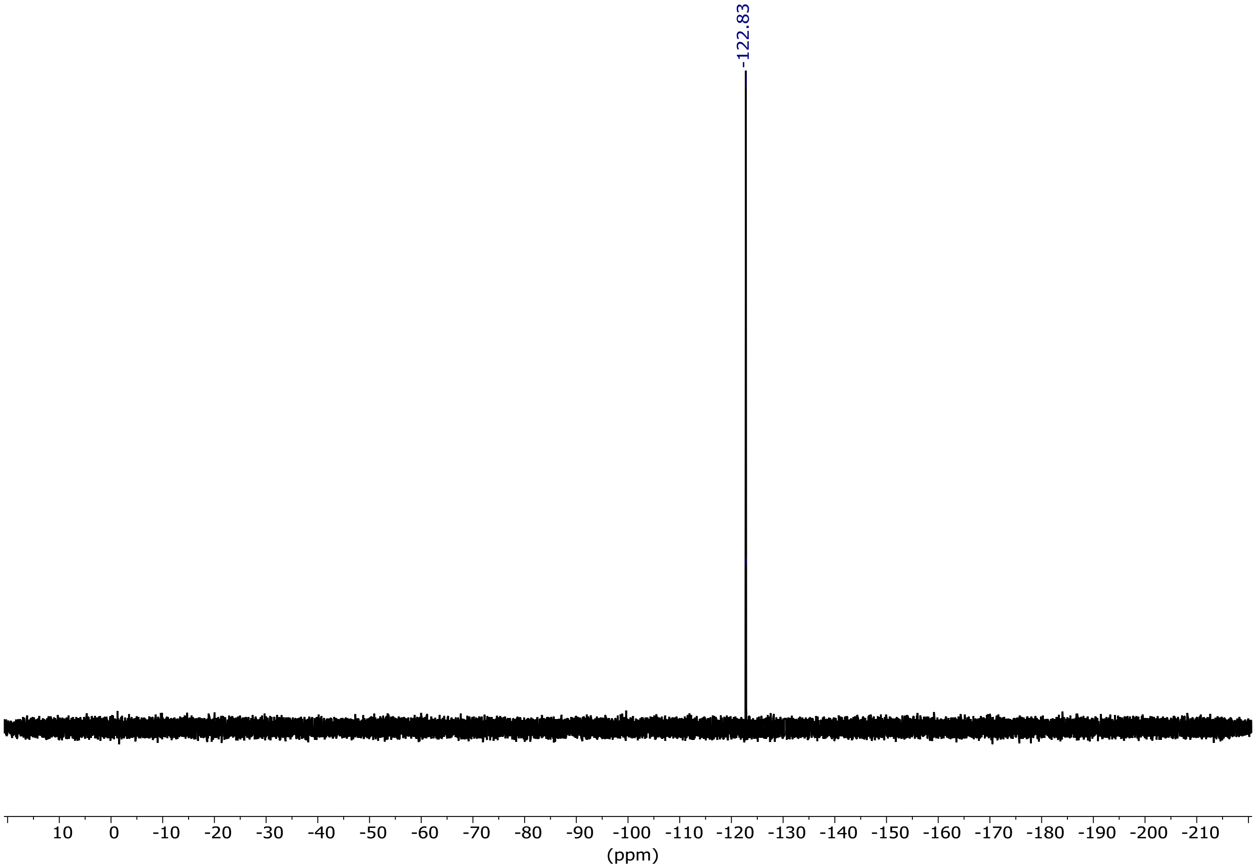

Figure S68. ^{19}F NMR spectrum of **8a**, externally referenced to fluorobenzene (-113.15 ppm).

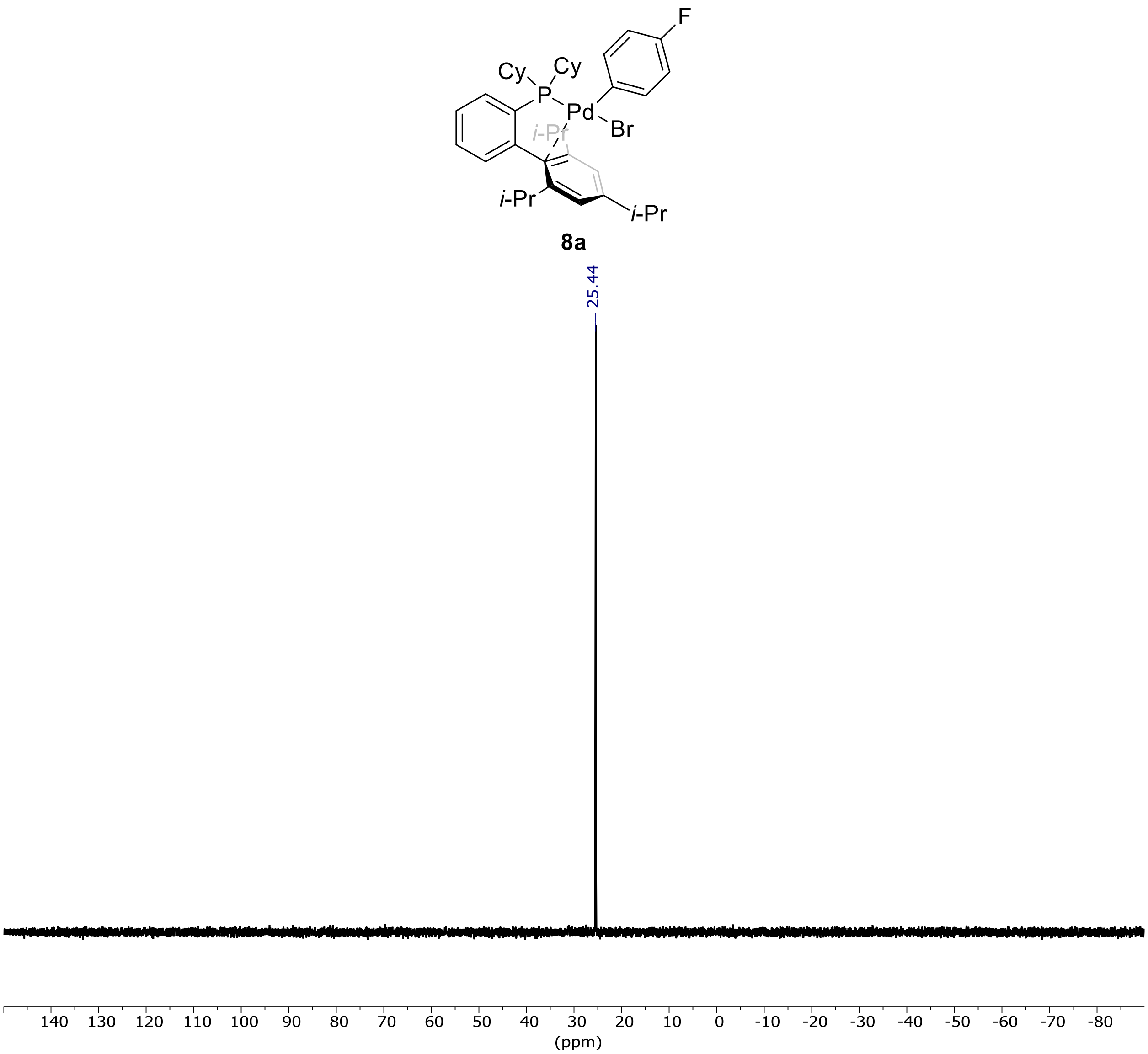

Figure S69. ^{31}P NMR spectrum of **8a**, externally referenced to triphenylphosphine (– 6.5 ppm).

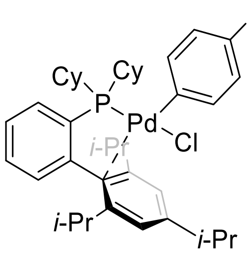

8b

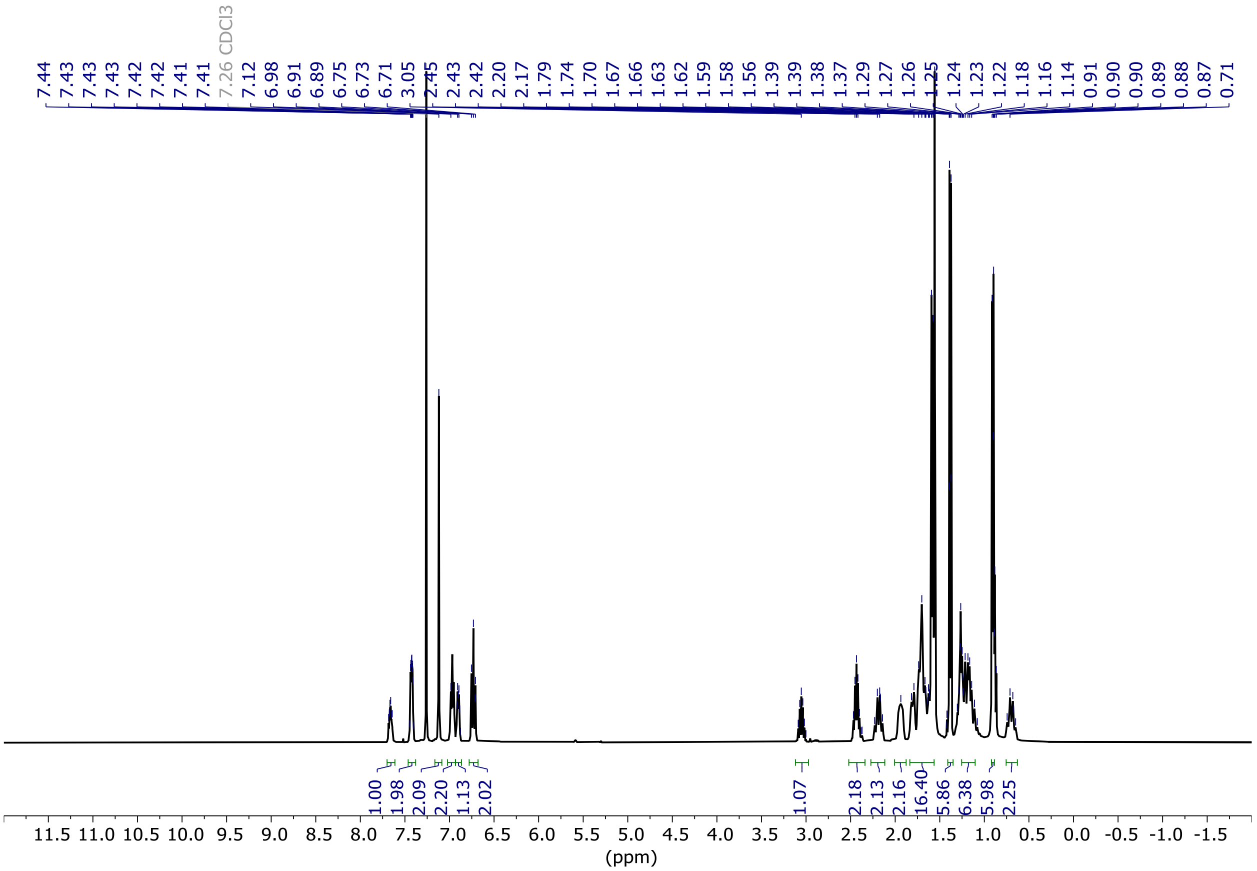

Figure S70. ^{1}H NMR spectrum of **8b**, referenced to $CDCl_3$ (7.26 ppm).

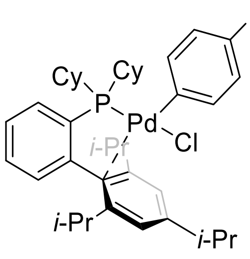

8b

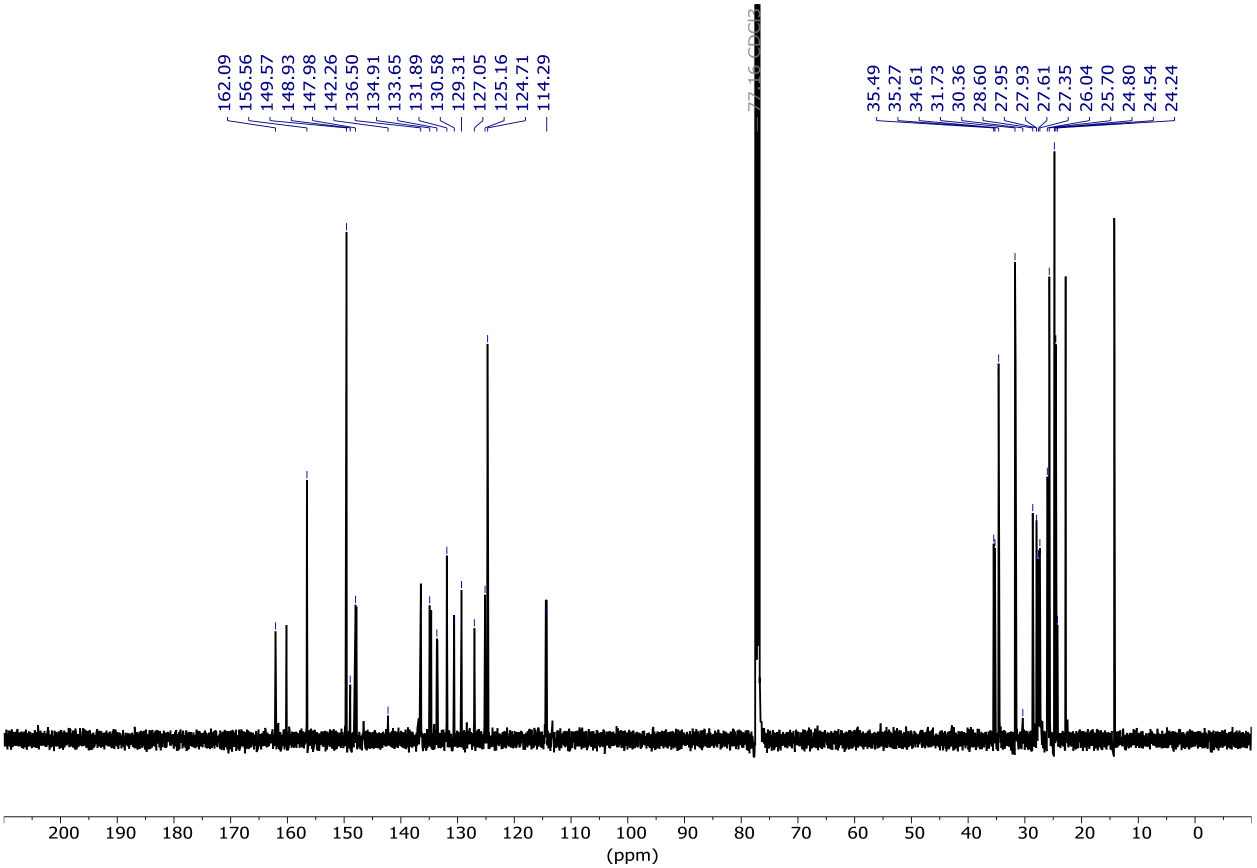

Figure S71. ^{13}C NMR spectrum of **8b**, referenced to $CDCl_3$ (77.16 ppm).

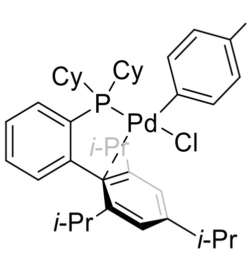

8b

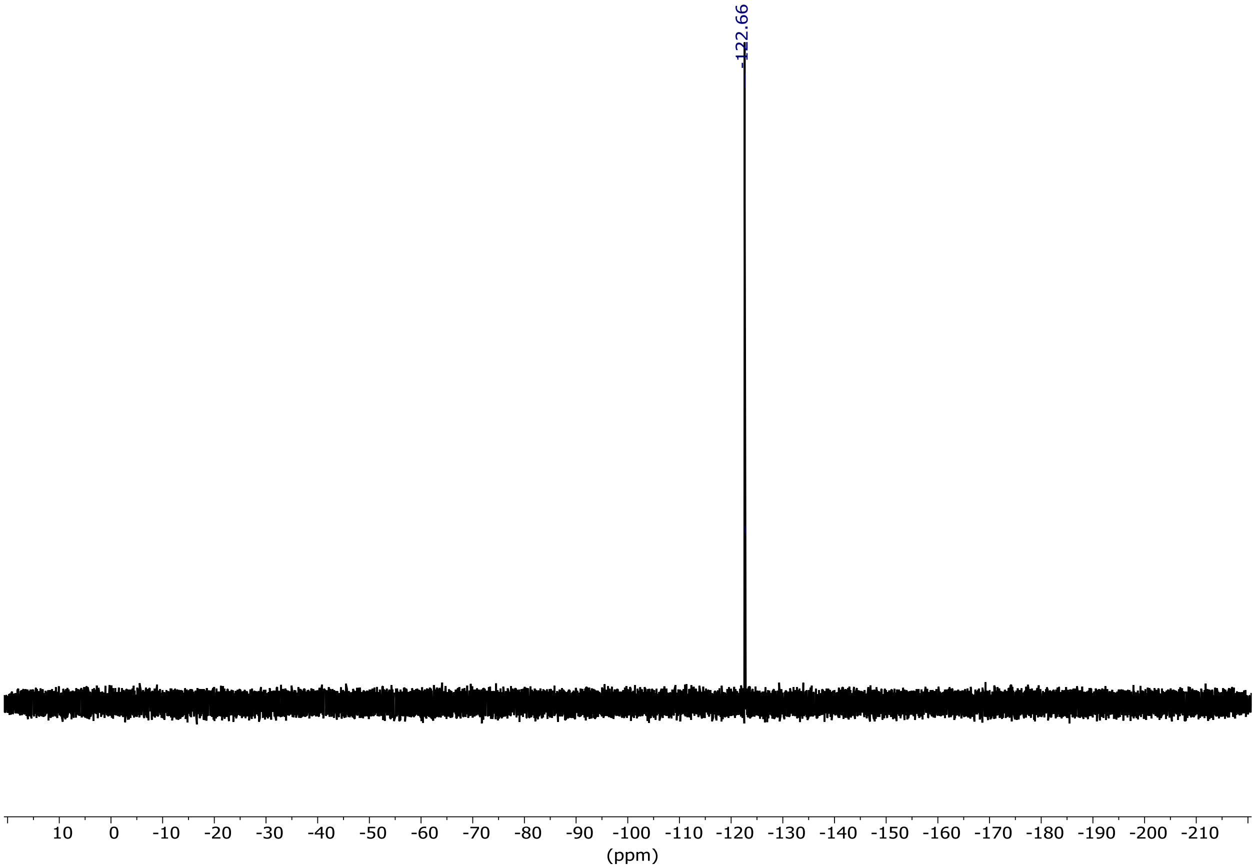

Figure S72. ^{19}F NMR spectrum of **8b**, externally referenced to fluorobenzene (–113.15 ppm).

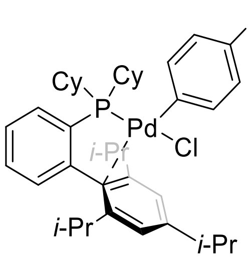

8b

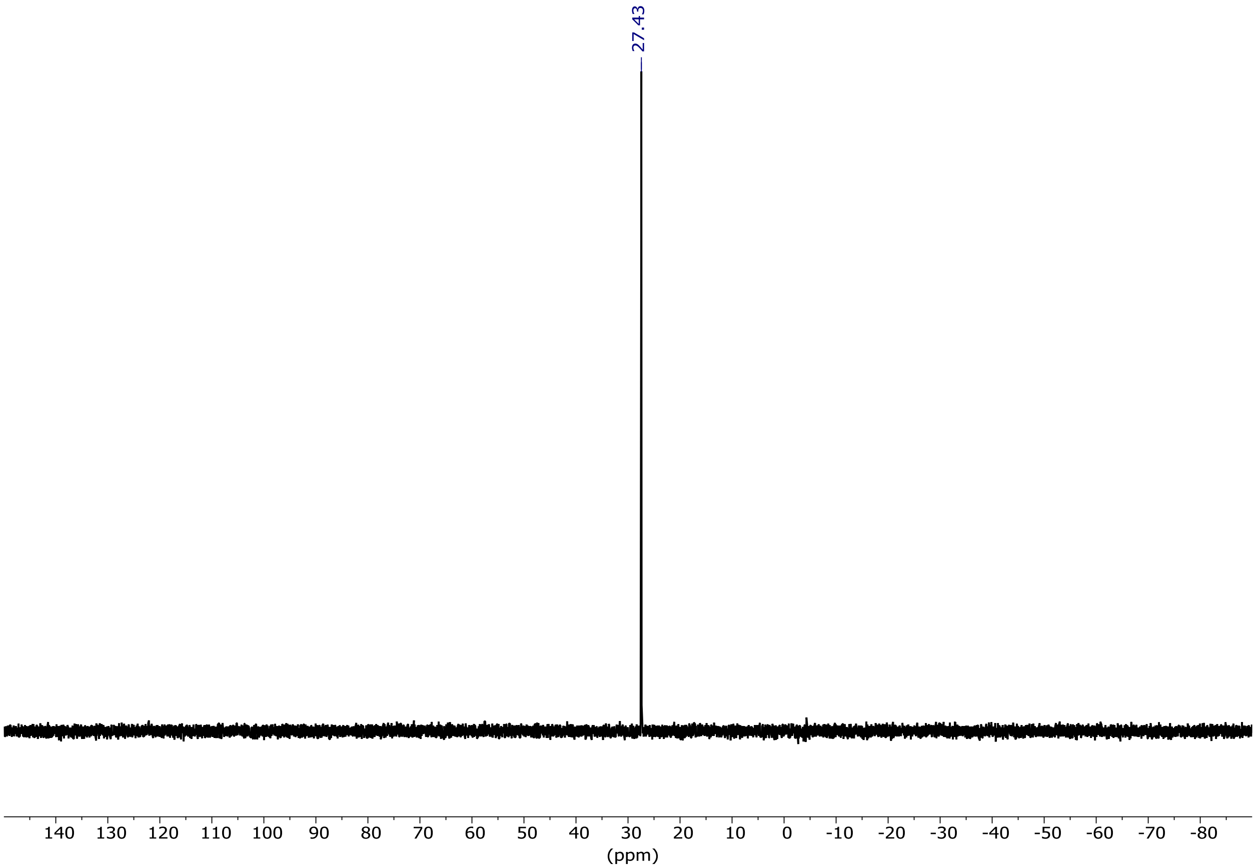

Figure S73. ^{31}P NMR spectrum of **8b**, externally referenced to triphenylphosphine (– 6.5 ppm).

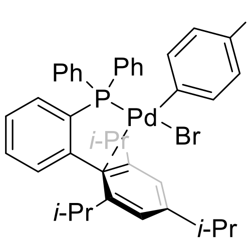

9a

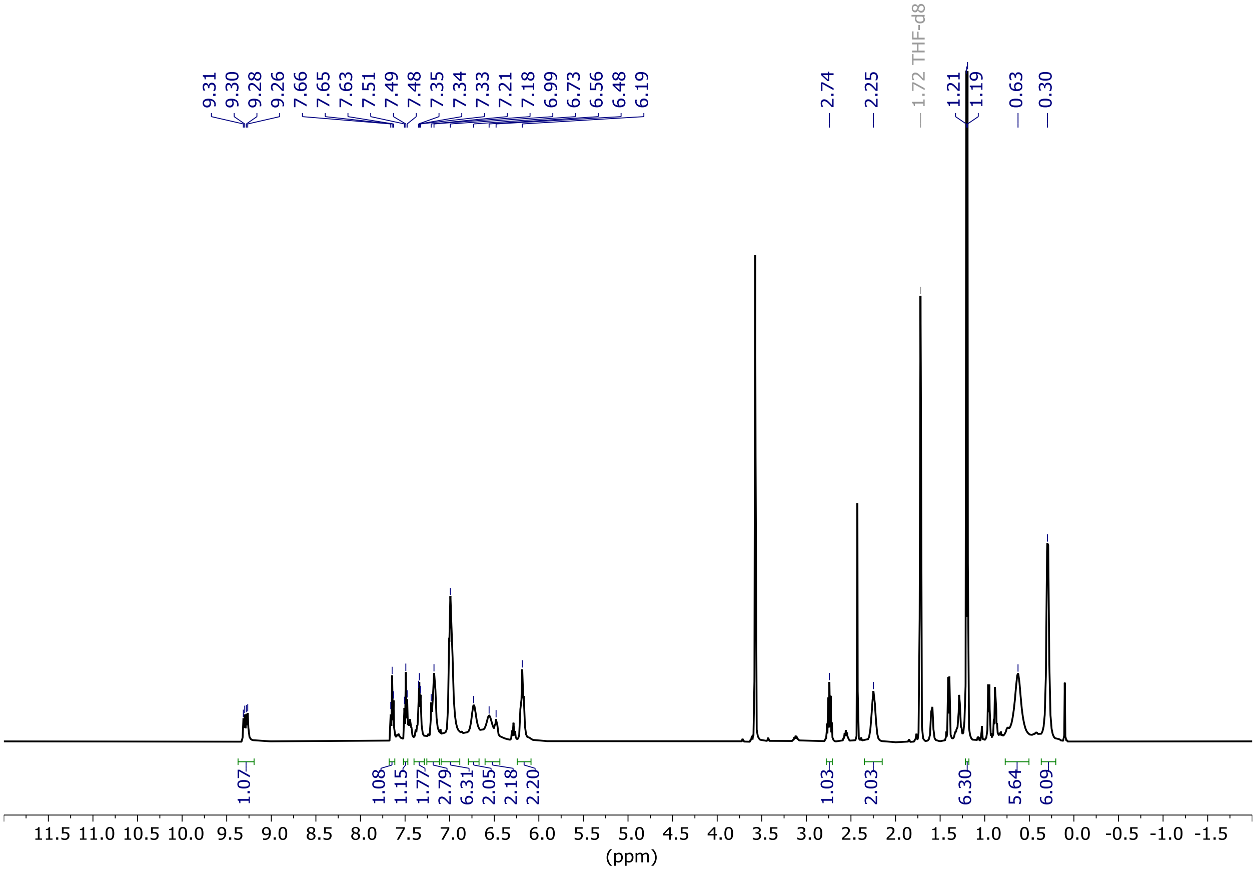

Figure S74. ^{1}H NMR spectrum of **9a**, referenced to THF-d_8 (1.72 ppm).

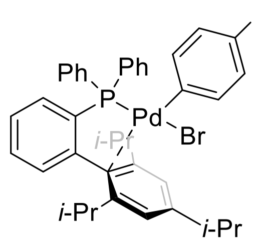

9a

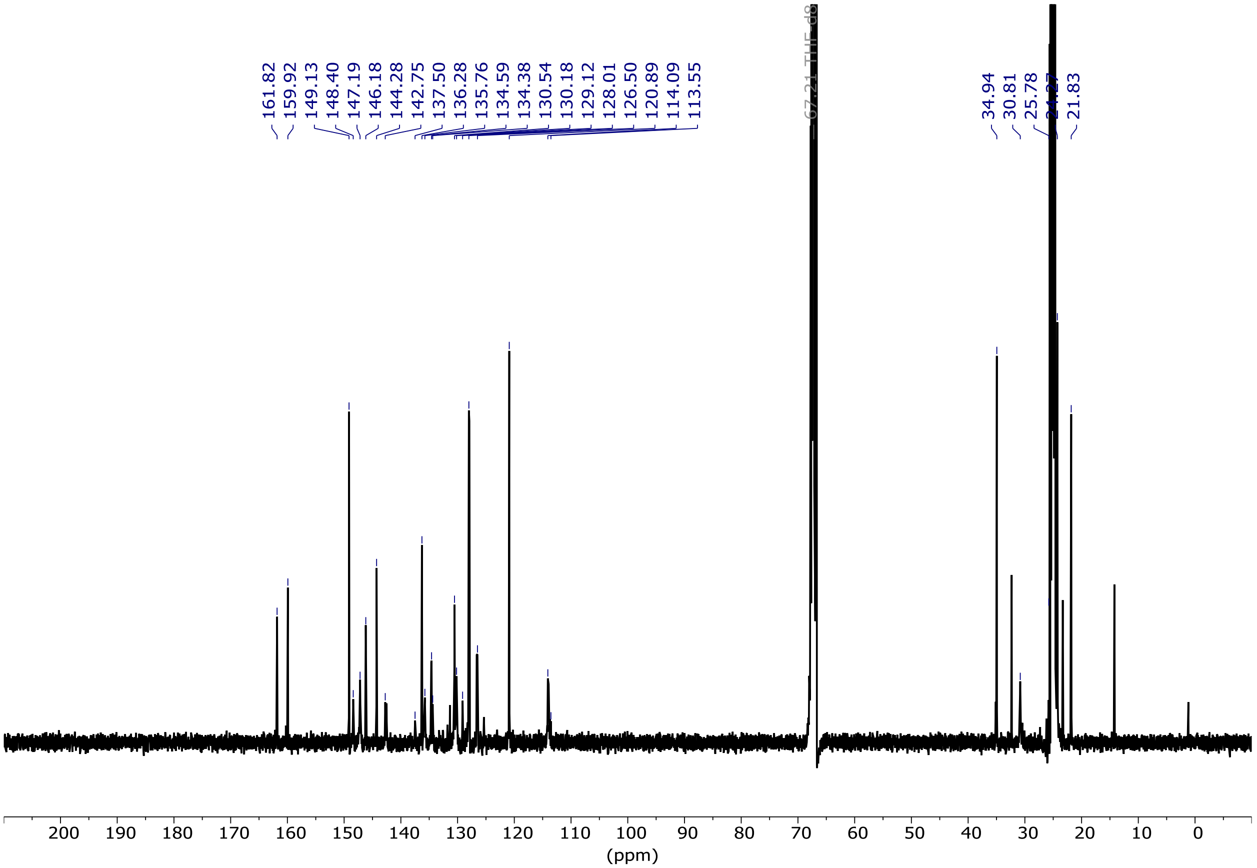

Figure S75. ^{13}C NMR spectrum of **9a**, referenced to THF-d_8 (67.21 ppm).

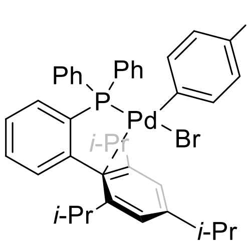

9a

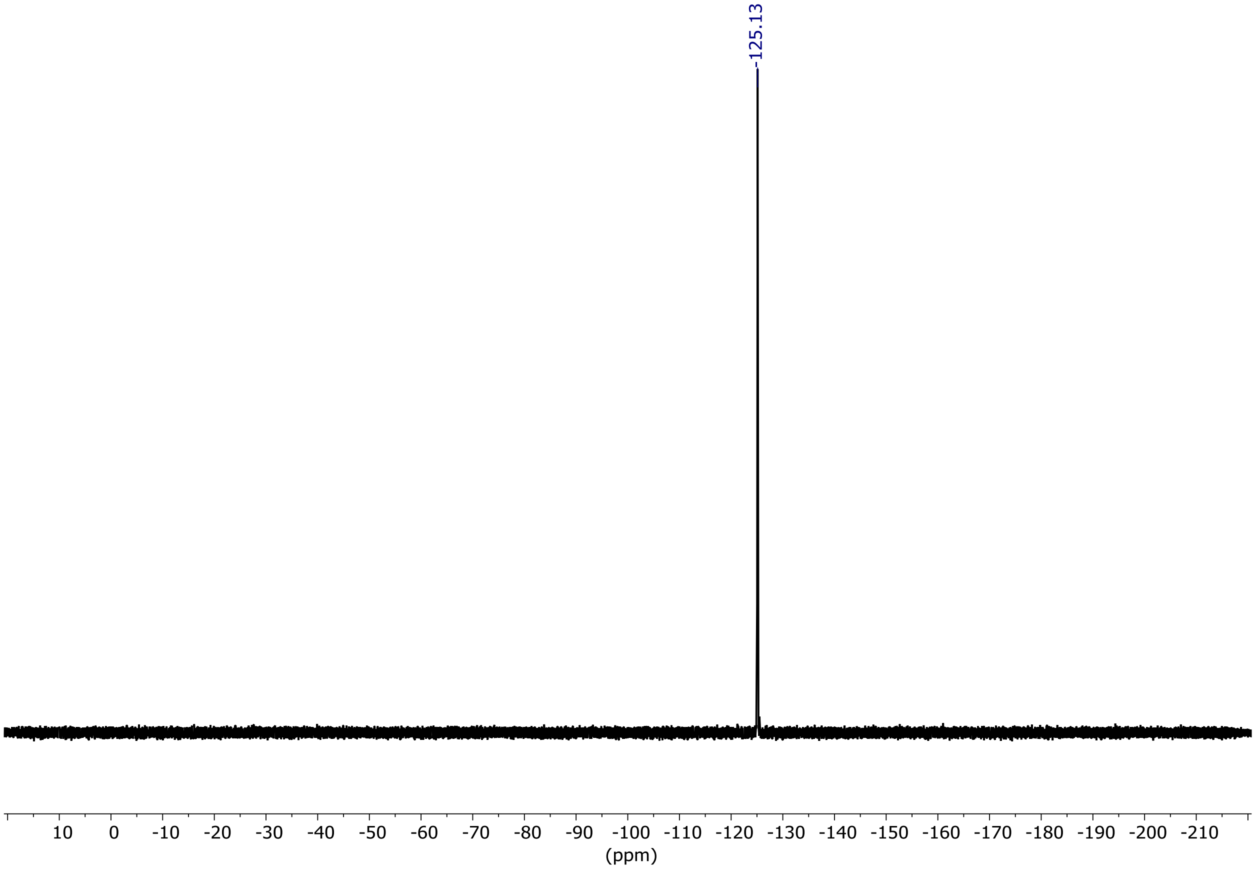

Figure S76. ^{19}F NMR spectrum of **9a**, externally referenced to fluorobenzene (-113.15 ppm).

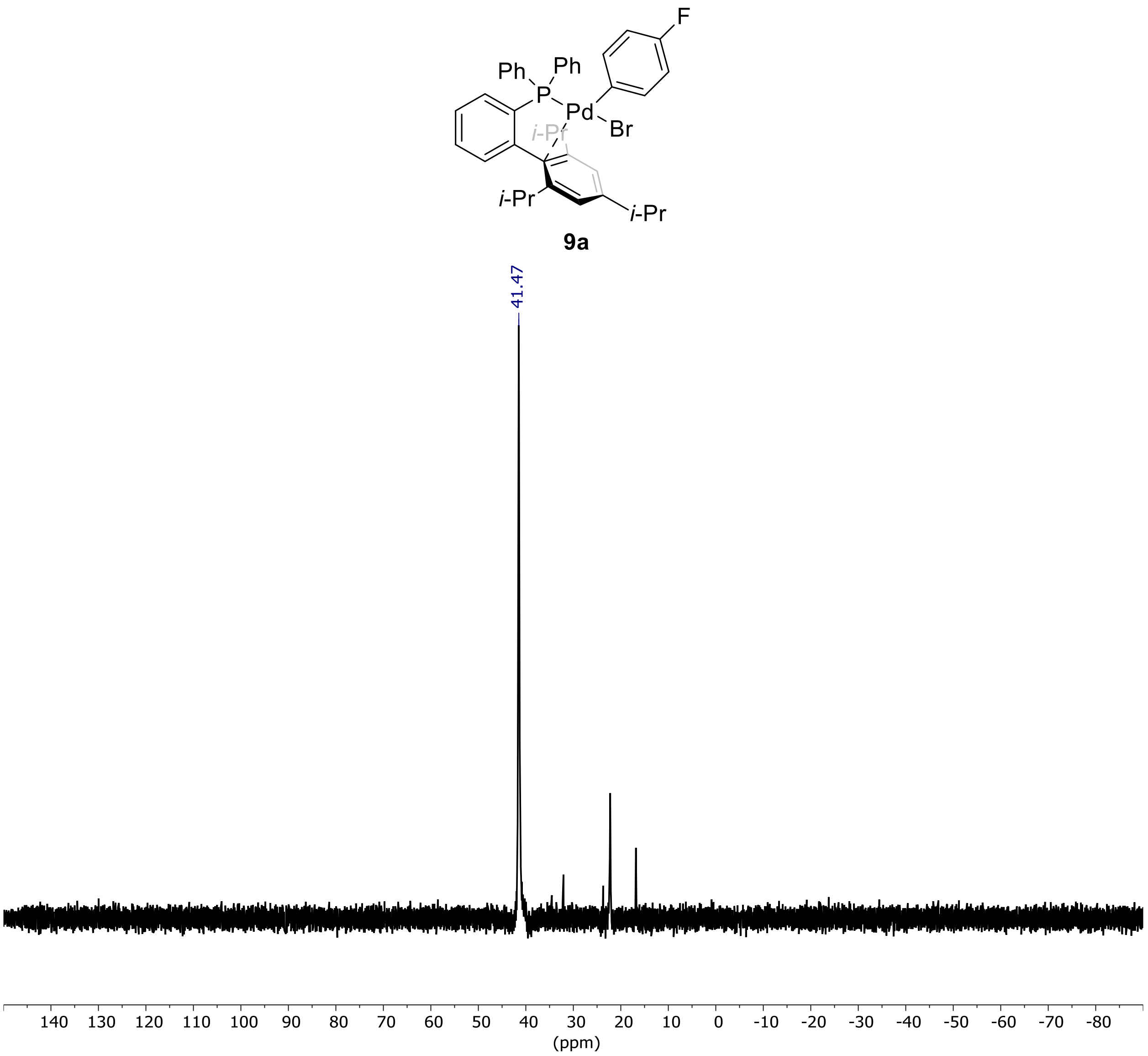

Figure S77. ^{31}P NMR spectrum of **9a**, externally referenced to triphenylphosphine (– 6.5 ppm).

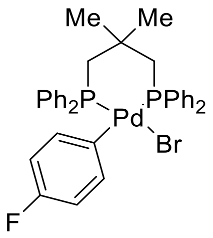

10a

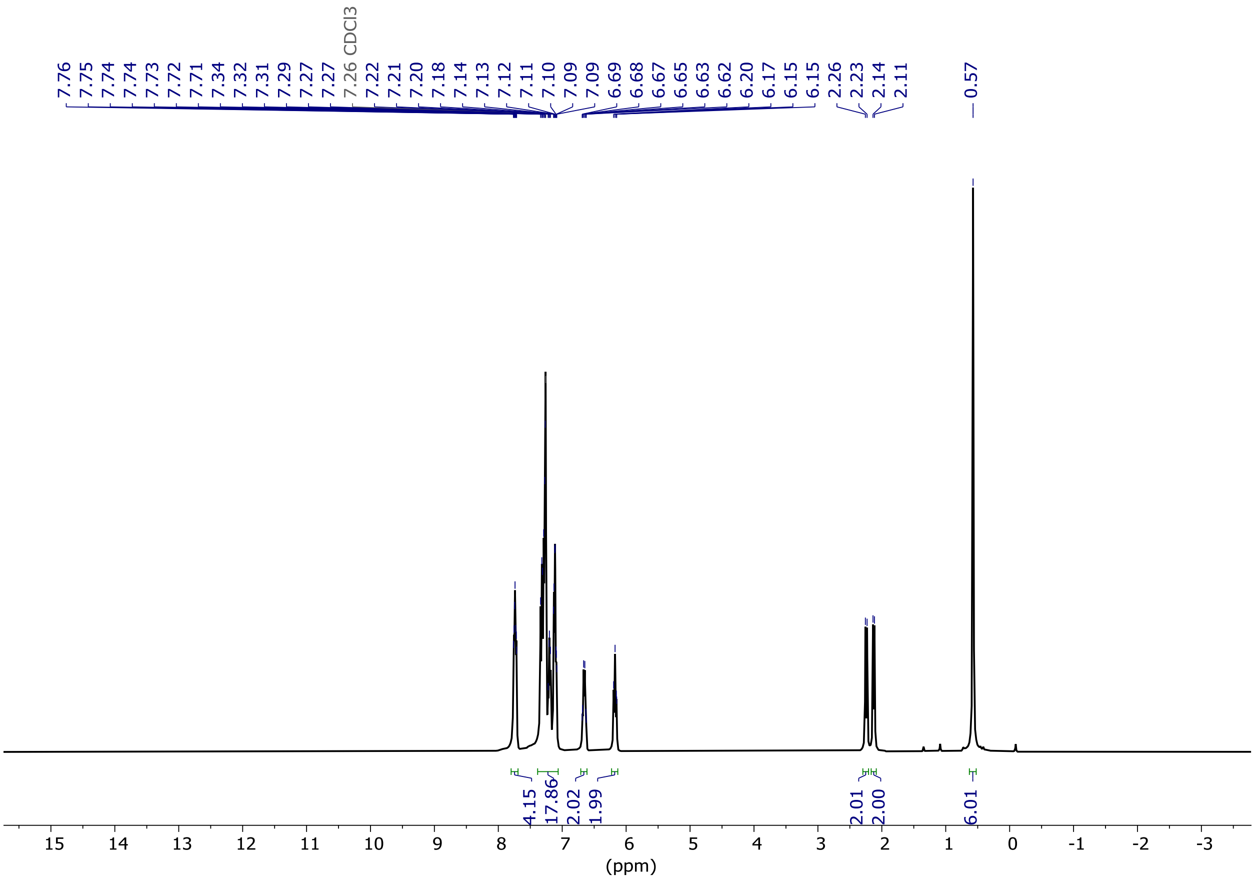

Figure S78. ^{1}H NMR spectrum of **10a**, referenced to $CDCl_3$ (7.26 ppm).

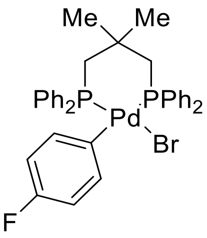

10a

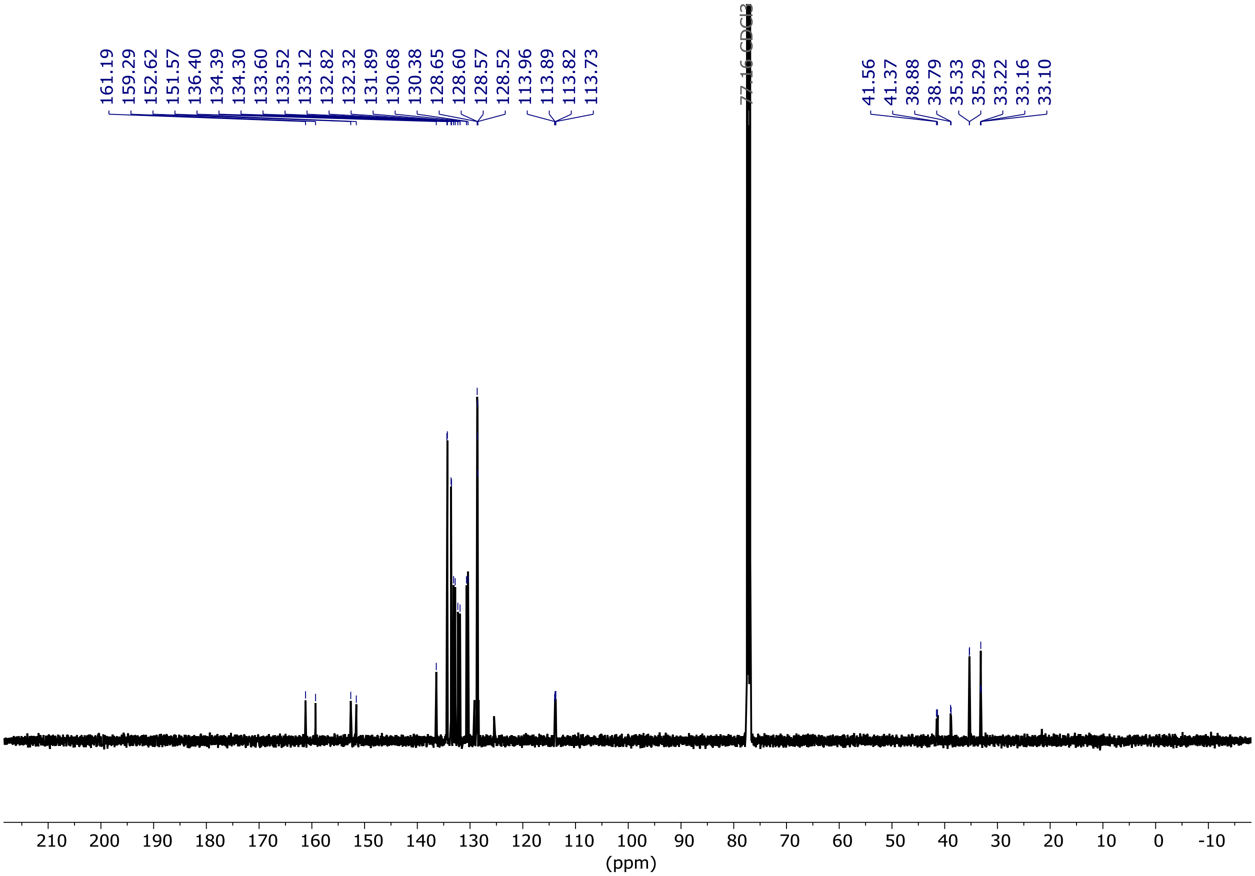

Figure S79. ^{13}C NMR spectrum of **10a**, referenced to $CDCl_3$ (77.16 ppm).

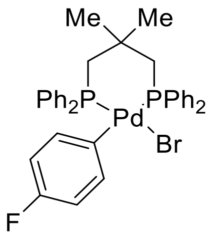

10a

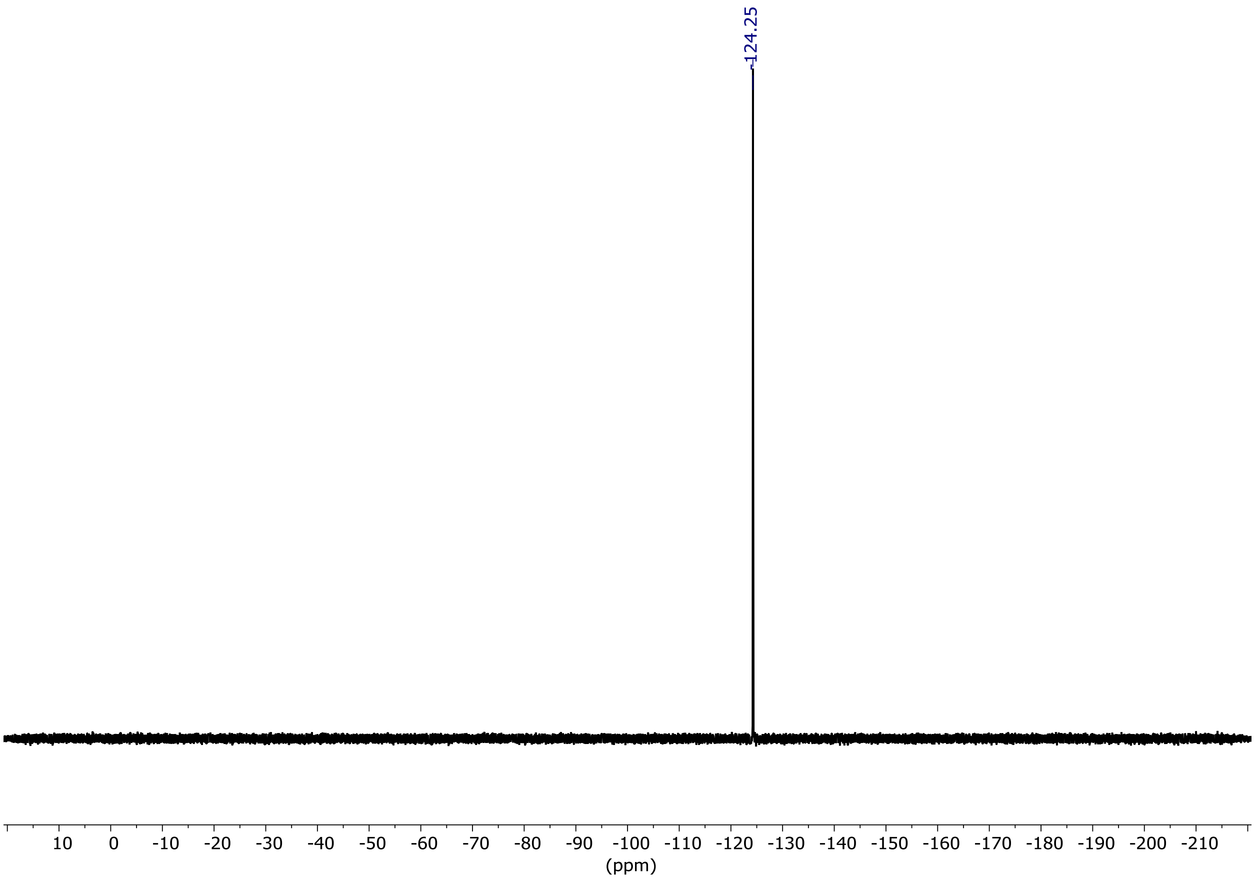

Figure S80. ^{19}F NMR spectrum of **10a**, externally referenced to fluorobenzene (–113.15 ppm).

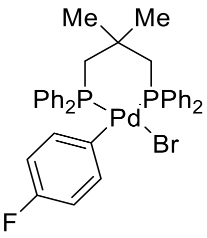

10a

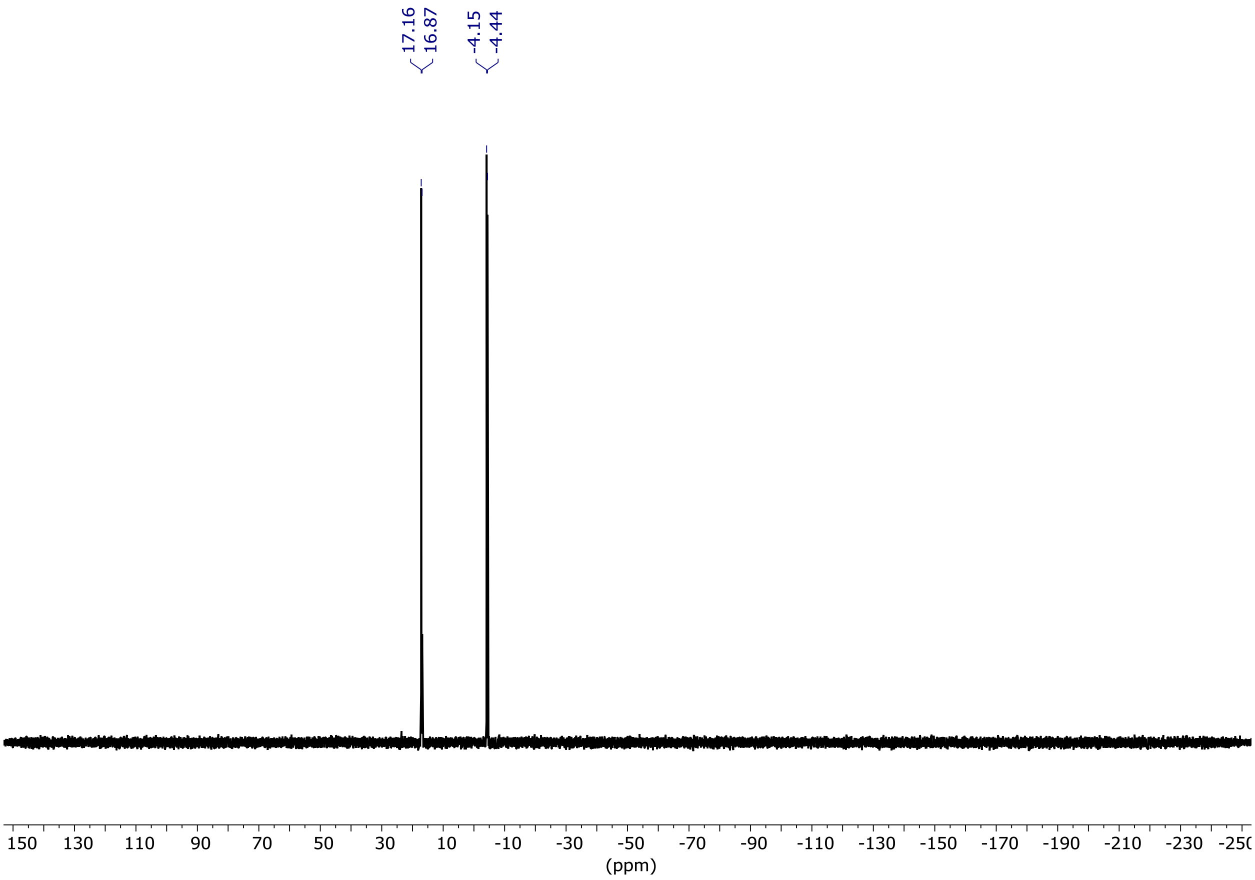

Figure S81. ^{31}P NMR spectrum of **10a**, externally referenced to triphenylphosphine (– 6.5 ppm).

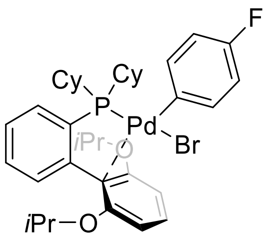

11a

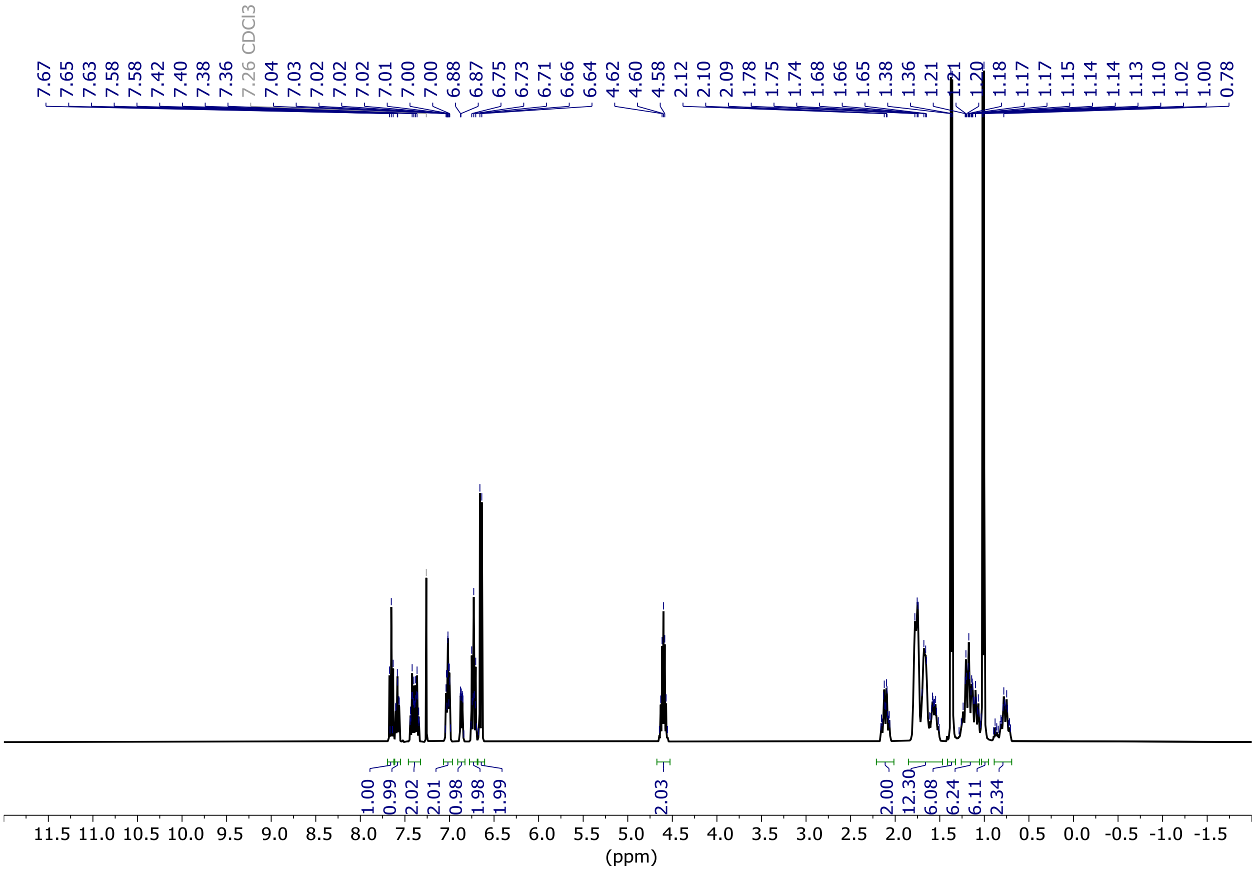

Figure S82. ^{1}H NMR spectrum of **11a**, referenced to $CDCl_3$ (7.26 ppm).

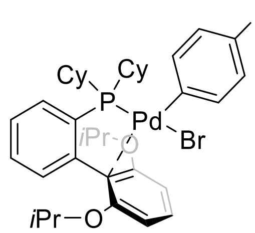

11a

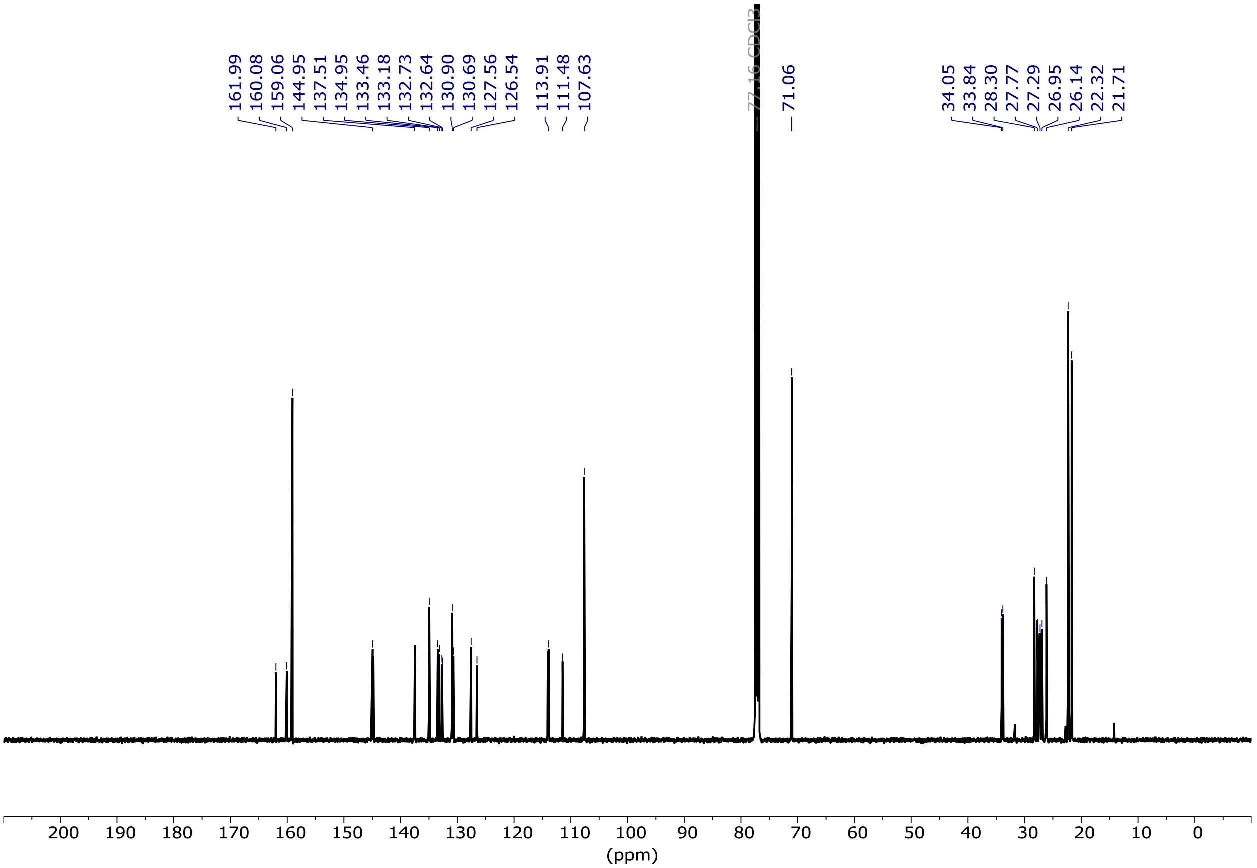

Figure S83. ^{13}C NMR spectrum of **11a**, referenced to $CDCl_3$ (77.16 ppm).

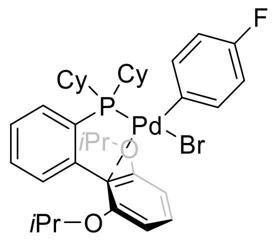

11a

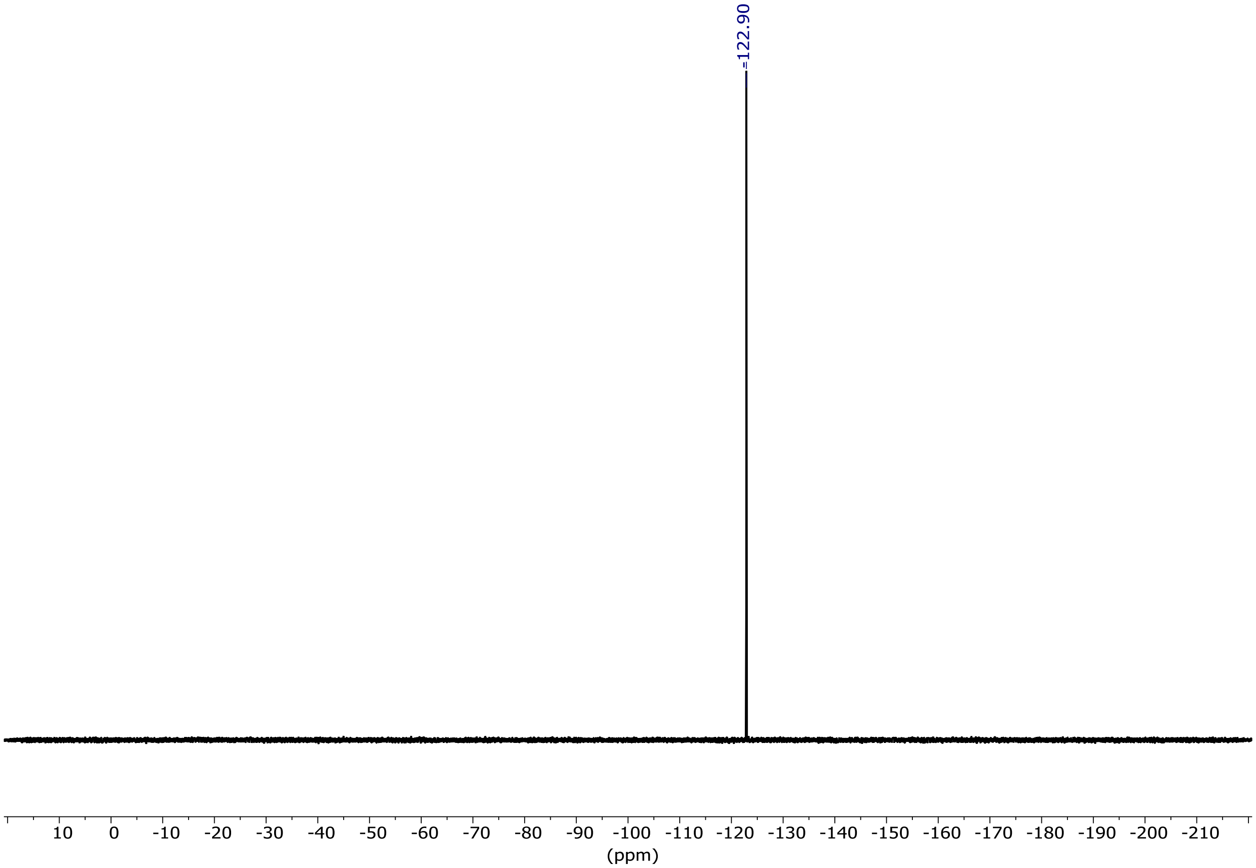

Figure S84. ^{19}F NMR spectrum of **11a**, externally referenced to fluorobenzene (-113.15 ppm).

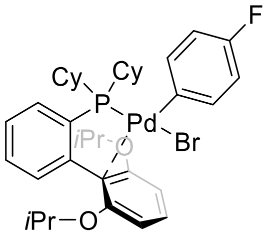

11a

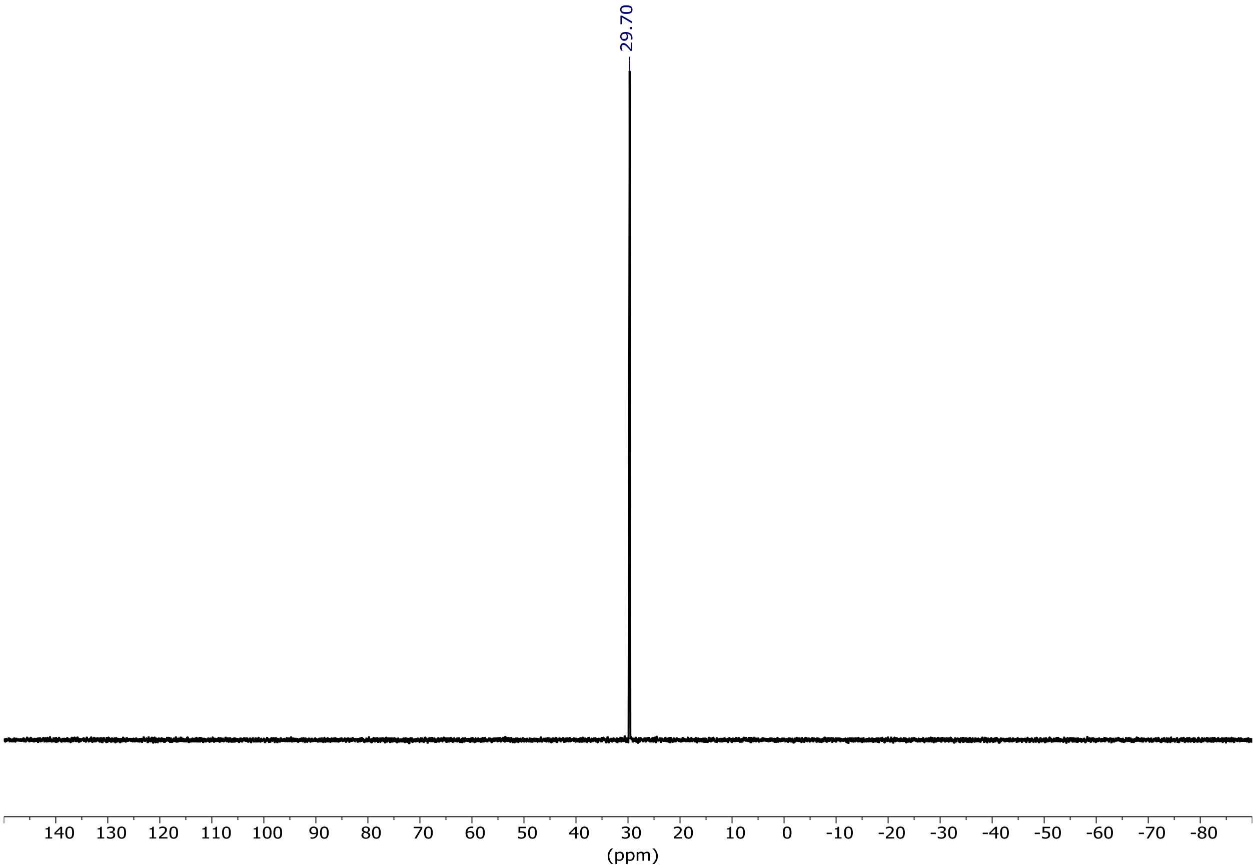

Figure S85. ^{31}P NMR spectrum of **11a**, externally referenced to triphenylphosphine (– 6.5 ppm).

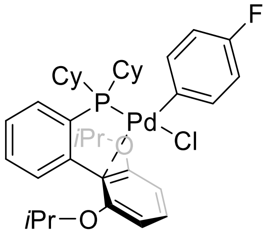

11b

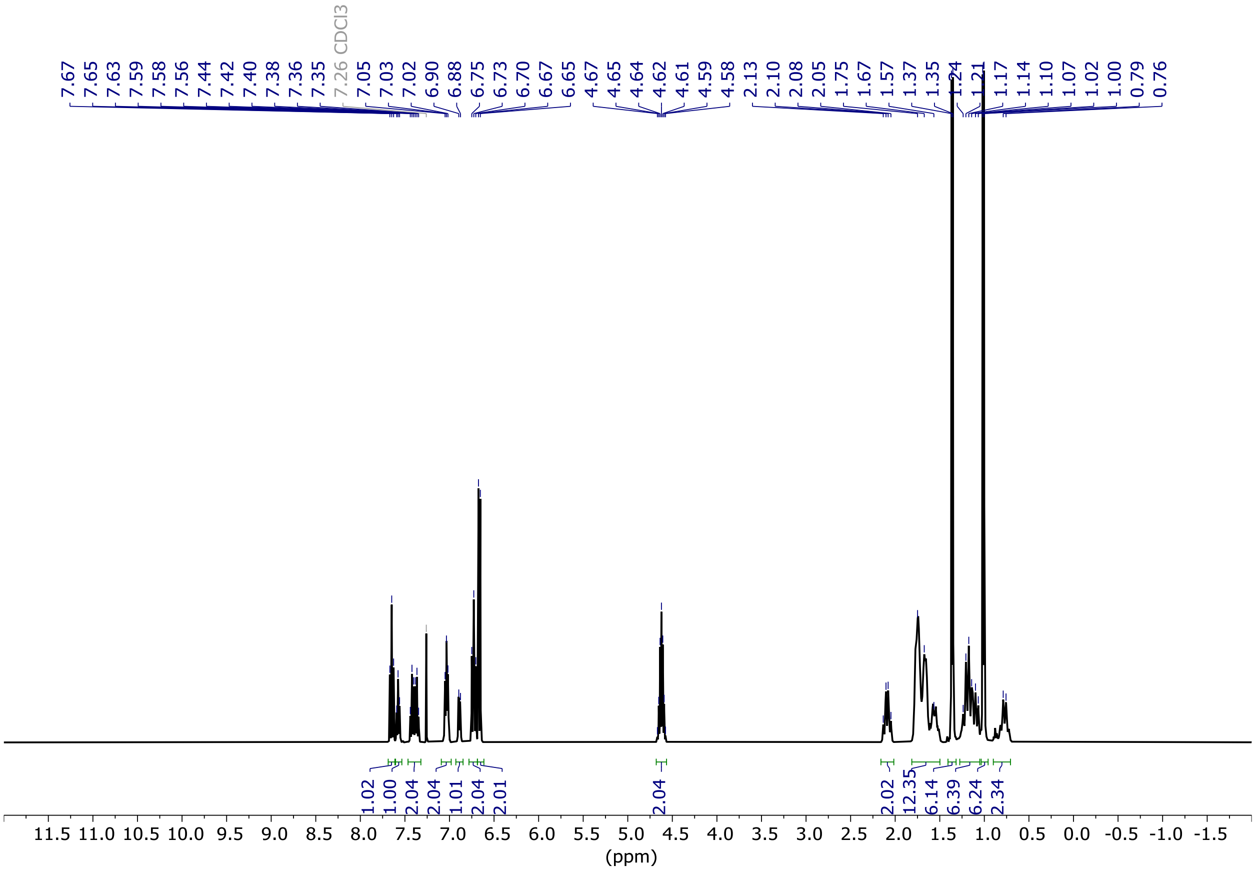

Figure S86. ^{1}H NMR spectrum of **11b**, referenced to $CDCl_3$ (7.26 ppm).

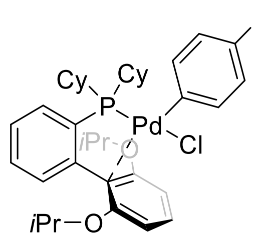

11b

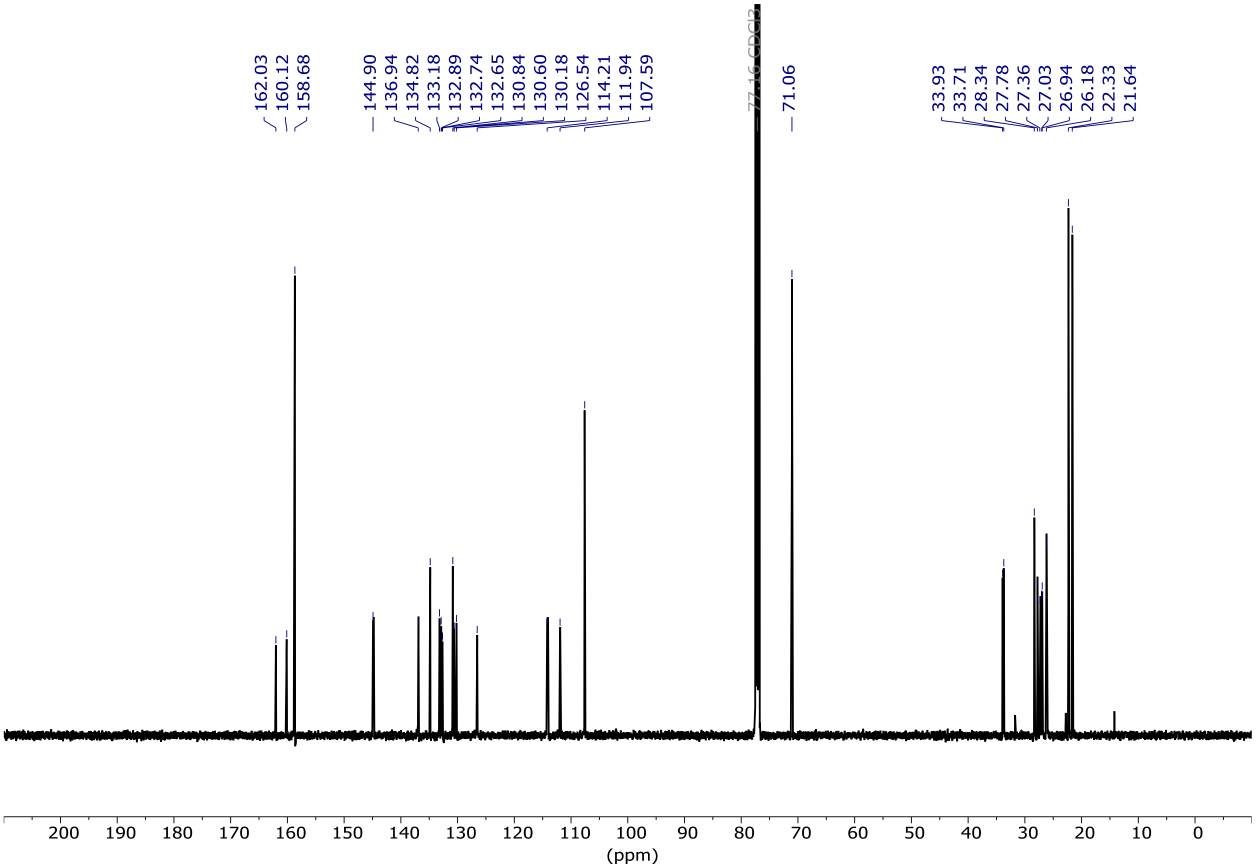

Figure S87. ^{13}C NMR spectrum of **11b**, referenced to $CDCl_3$ (77.16 ppm).

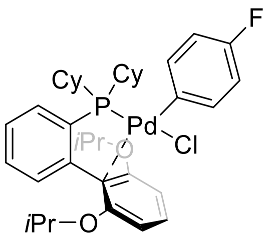

11b

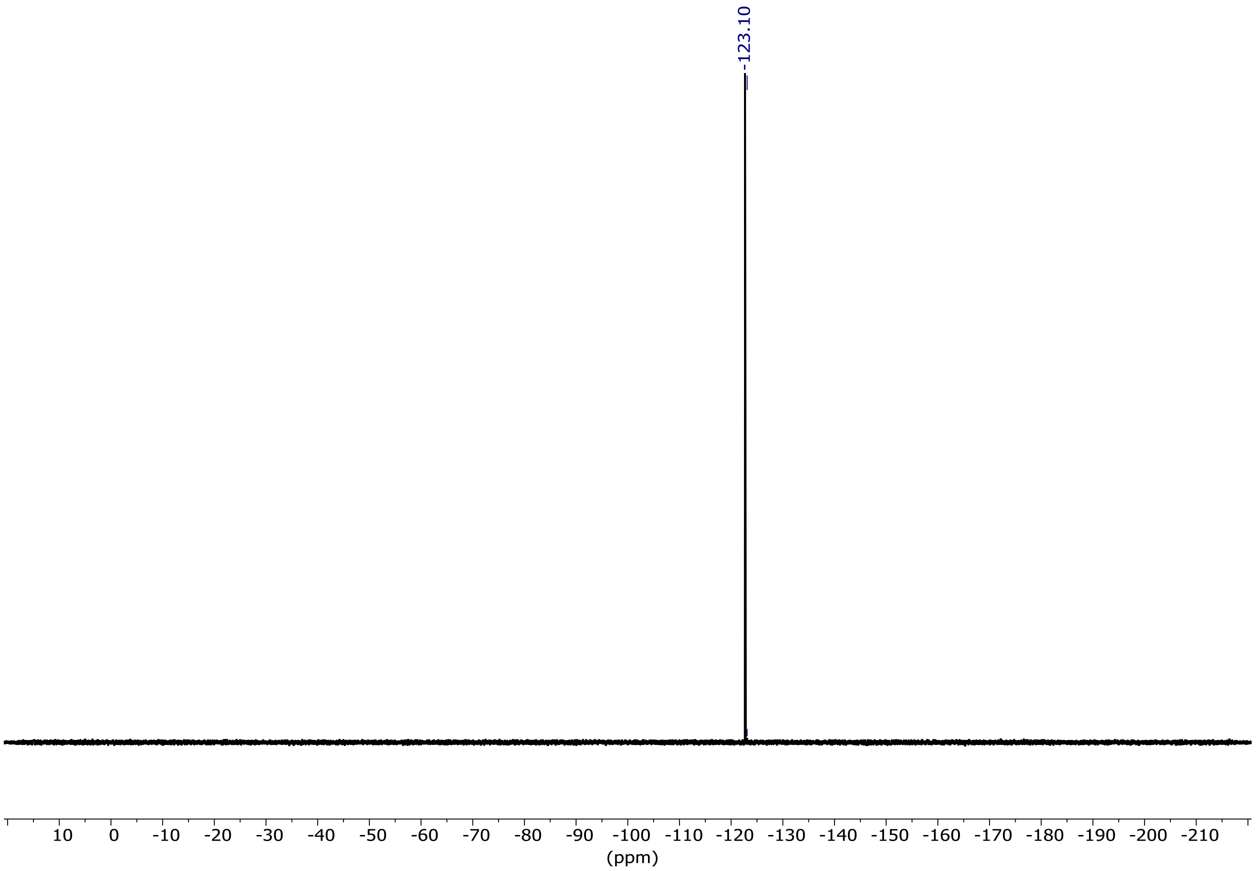

Figure S88. ^{19}F NMR spectrum of **11b**, externally referenced to fluorobenzene (-113.15 ppm).

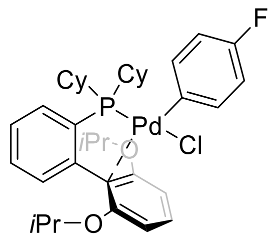

11b

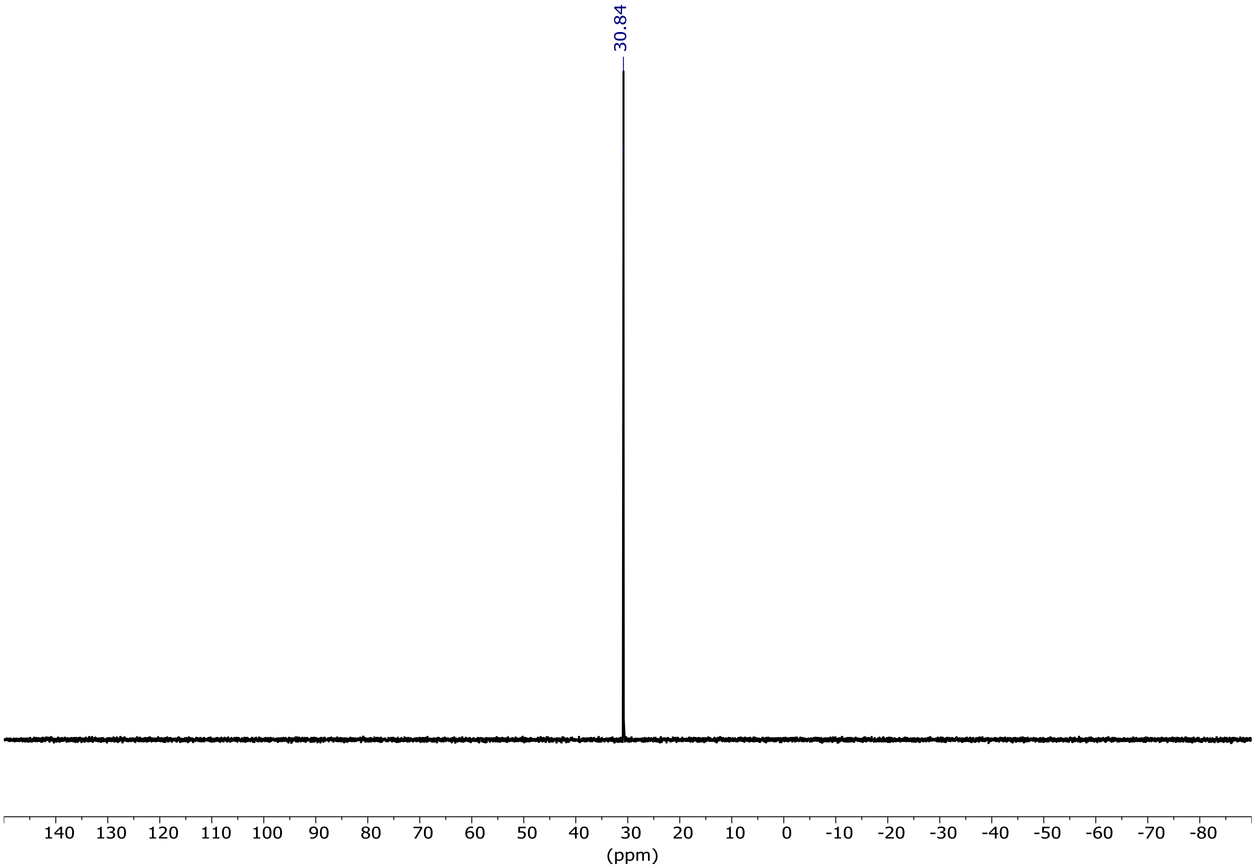

Figure S89. ^{31}P NMR spectrum of **11b**, externally referenced to triphenylphosphine (– 6.5 ppm).

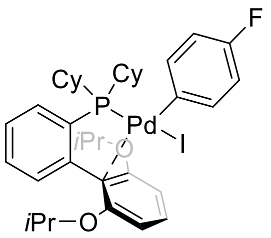

11c

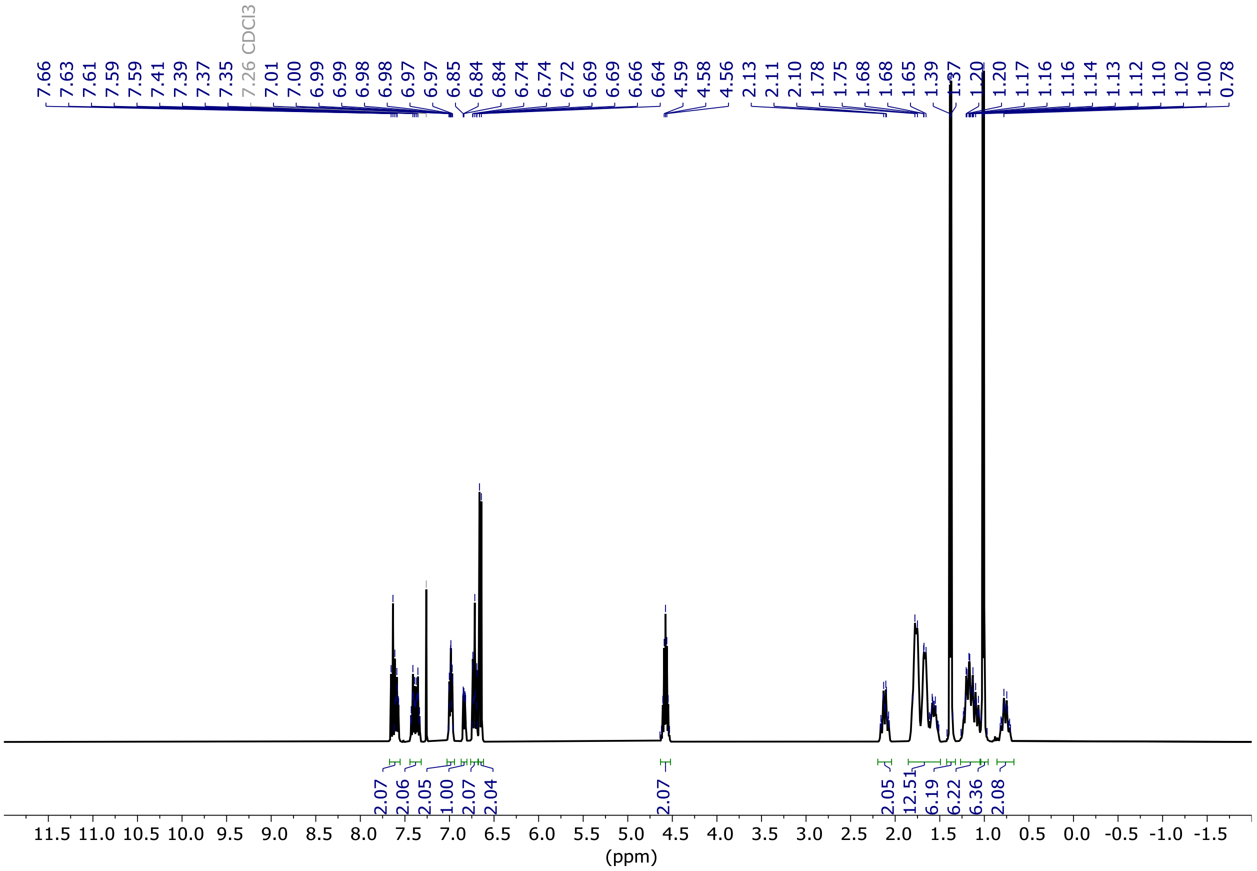

Figure S90. ^{1}H NMR spectrum of **11c**, referenced to $CDCl_3$ (7.26 ppm).

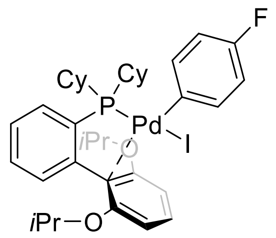

11c

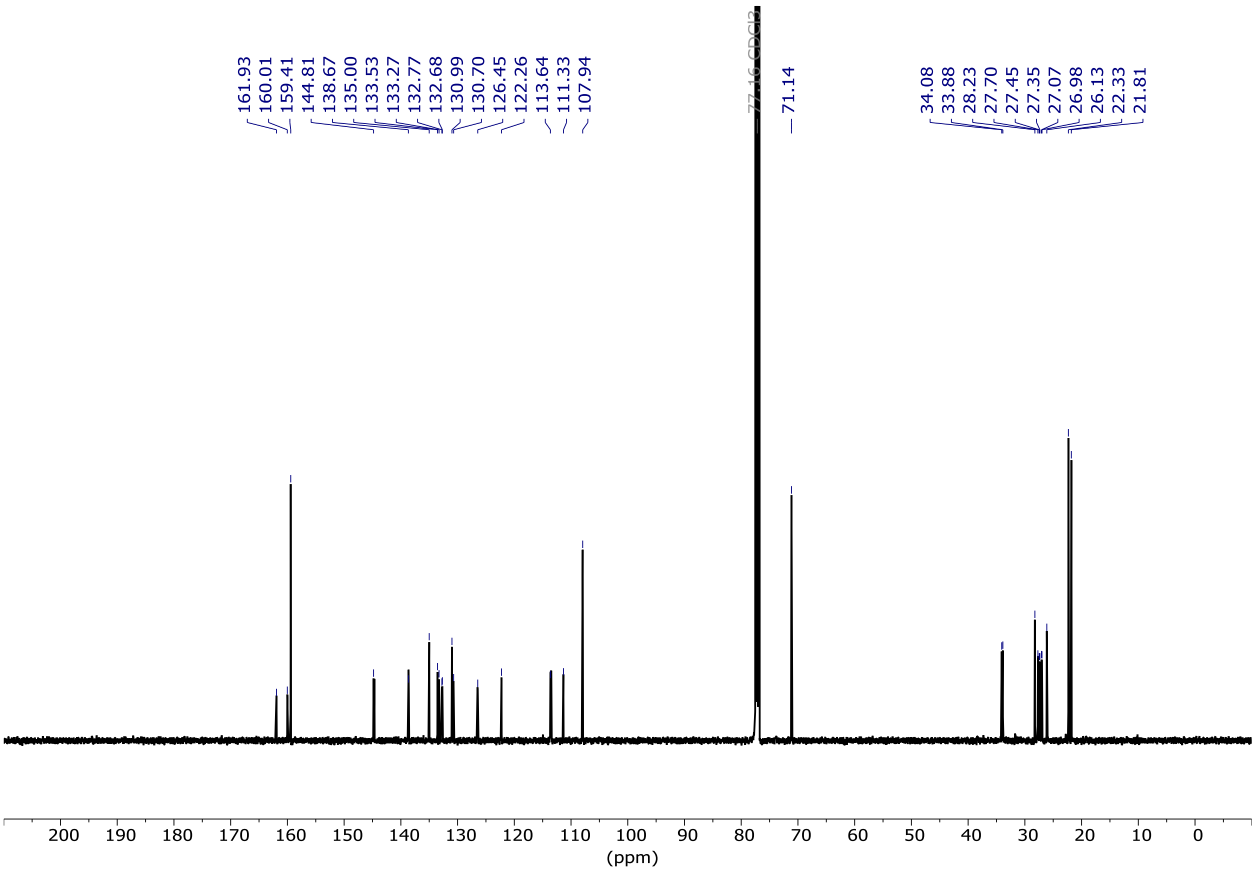

Figure S91. ^{13}C NMR spectrum of **11c**, referenced to $CDCl_3$ (77.16 ppm).

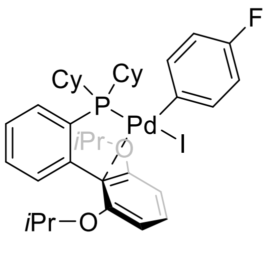

11c

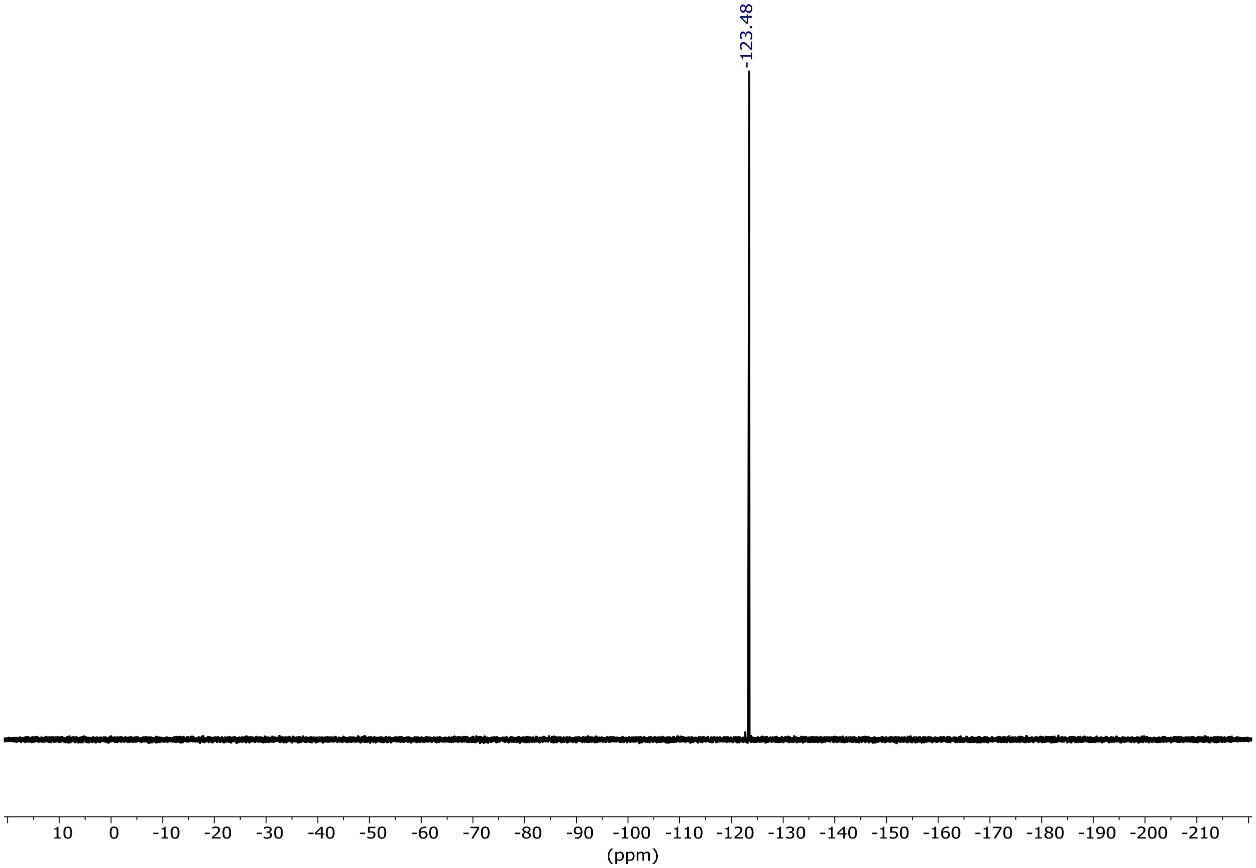

Figure S92. ^{19}F NMR spectrum of **11c**, externally referenced to fluorobenzene (-113.15 ppm).

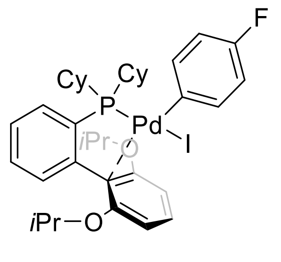

11c

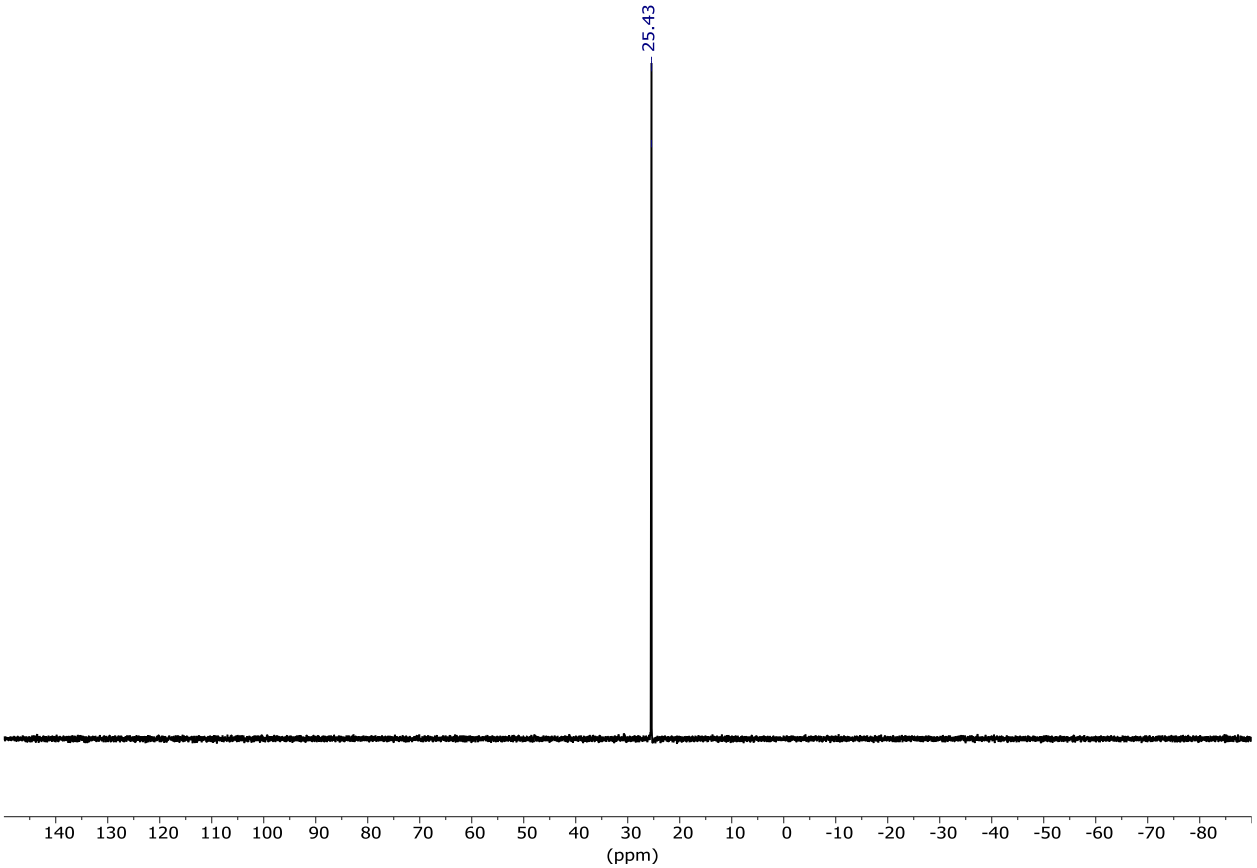

Figure S93. ^{31}P NMR spectrum of **11c**, externally referenced to triphenylphosphine (– 6.5 ppm).

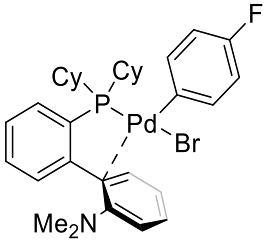

13a

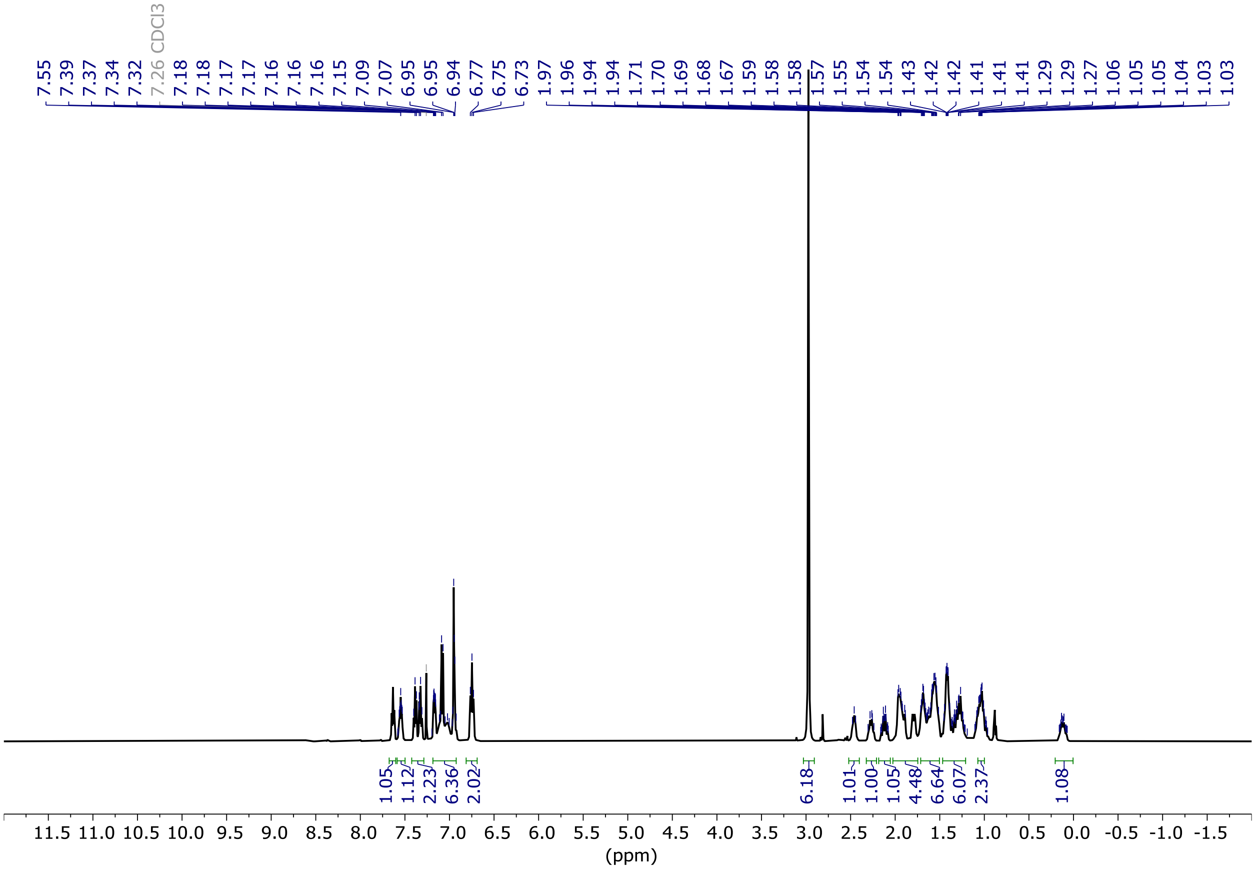

Figure S94. ^{1}H NMR spectrum of **13a**, referenced to $CDCl_3$ (7.26 ppm).

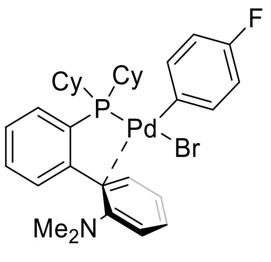

13a

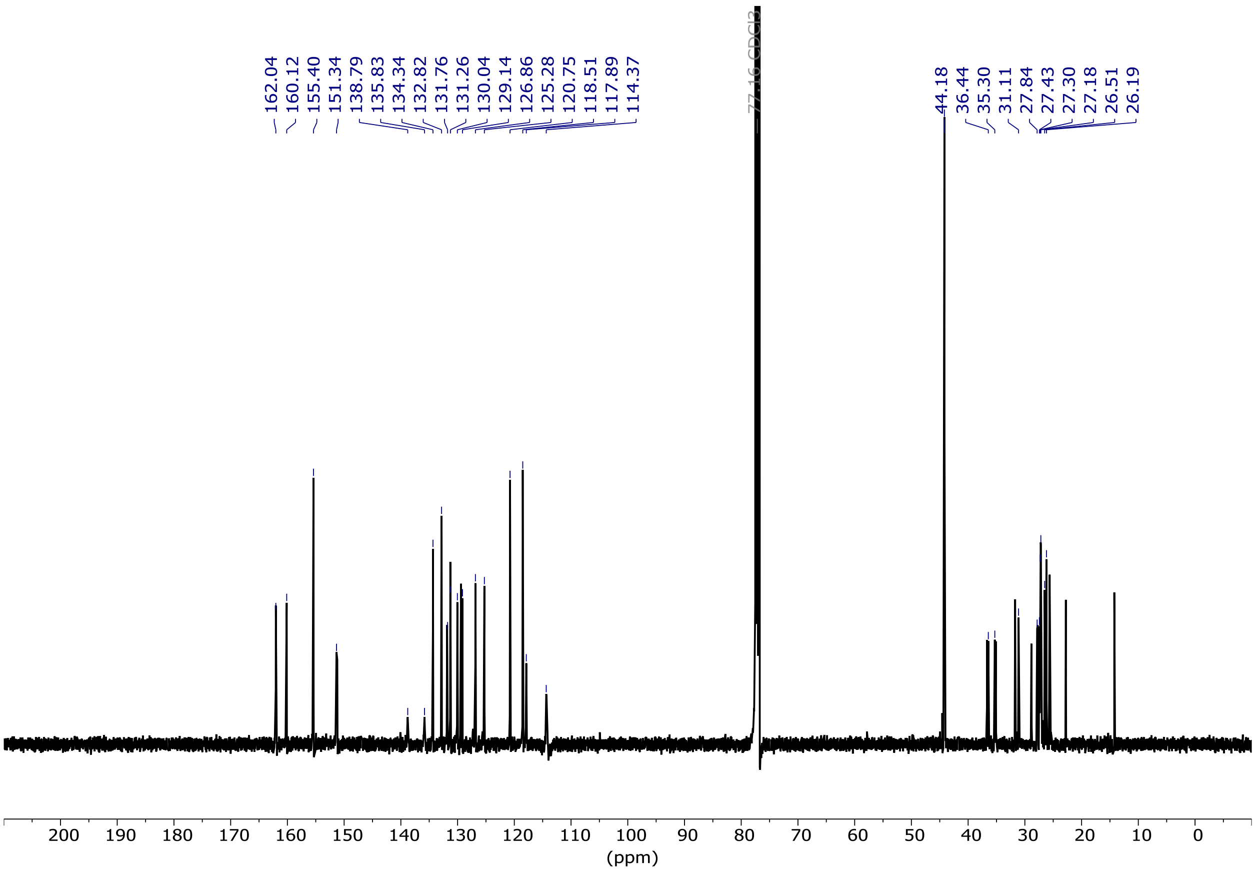

Figure S95. ^{13}C NMR spectrum of **13a**, referenced to $CDCl_3$ (77.16 ppm).

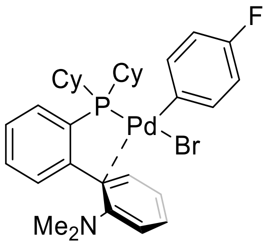

13a

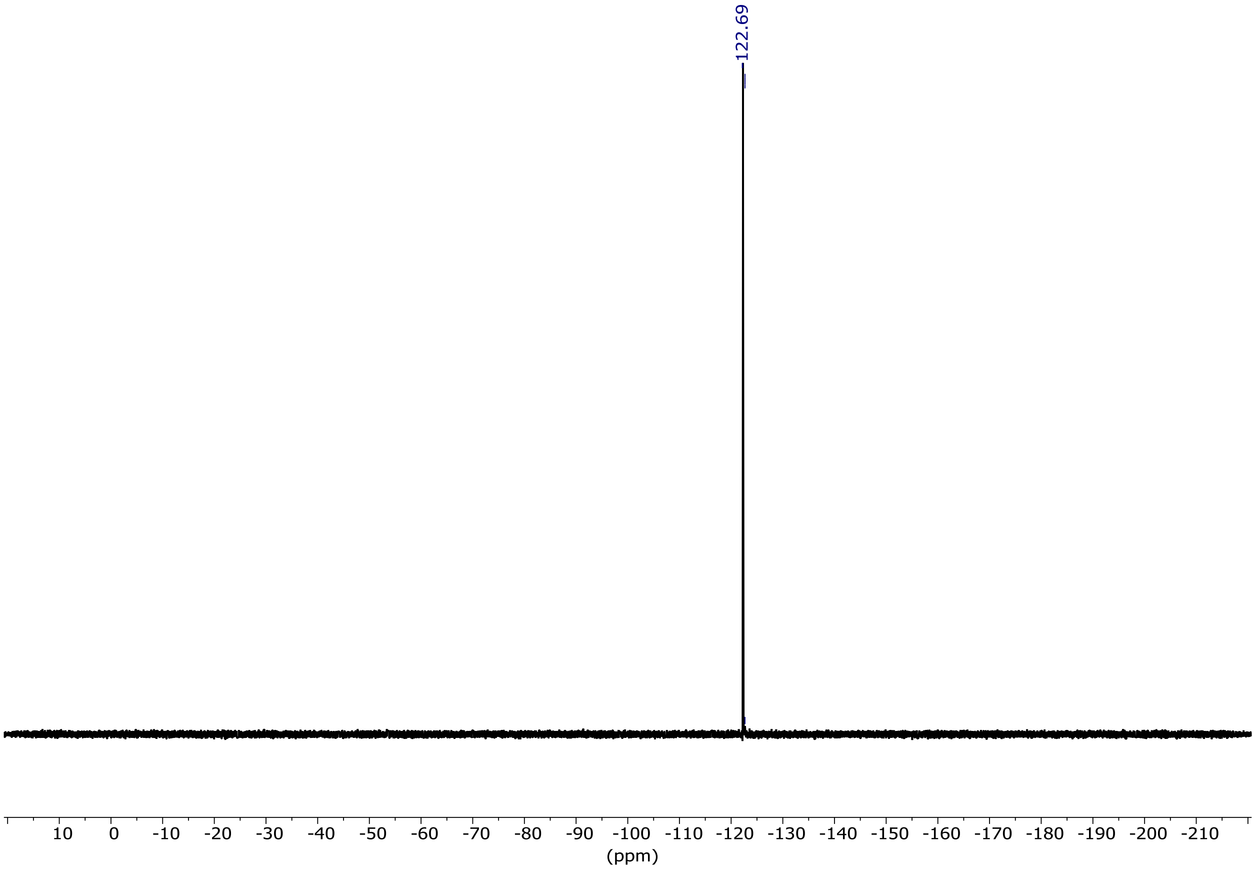

Figure S96. ^{19}F NMR spectrum of **13a**, externally referenced to fluorobenzene (-113.15 ppm).

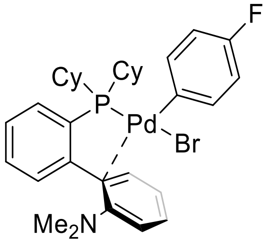

13a

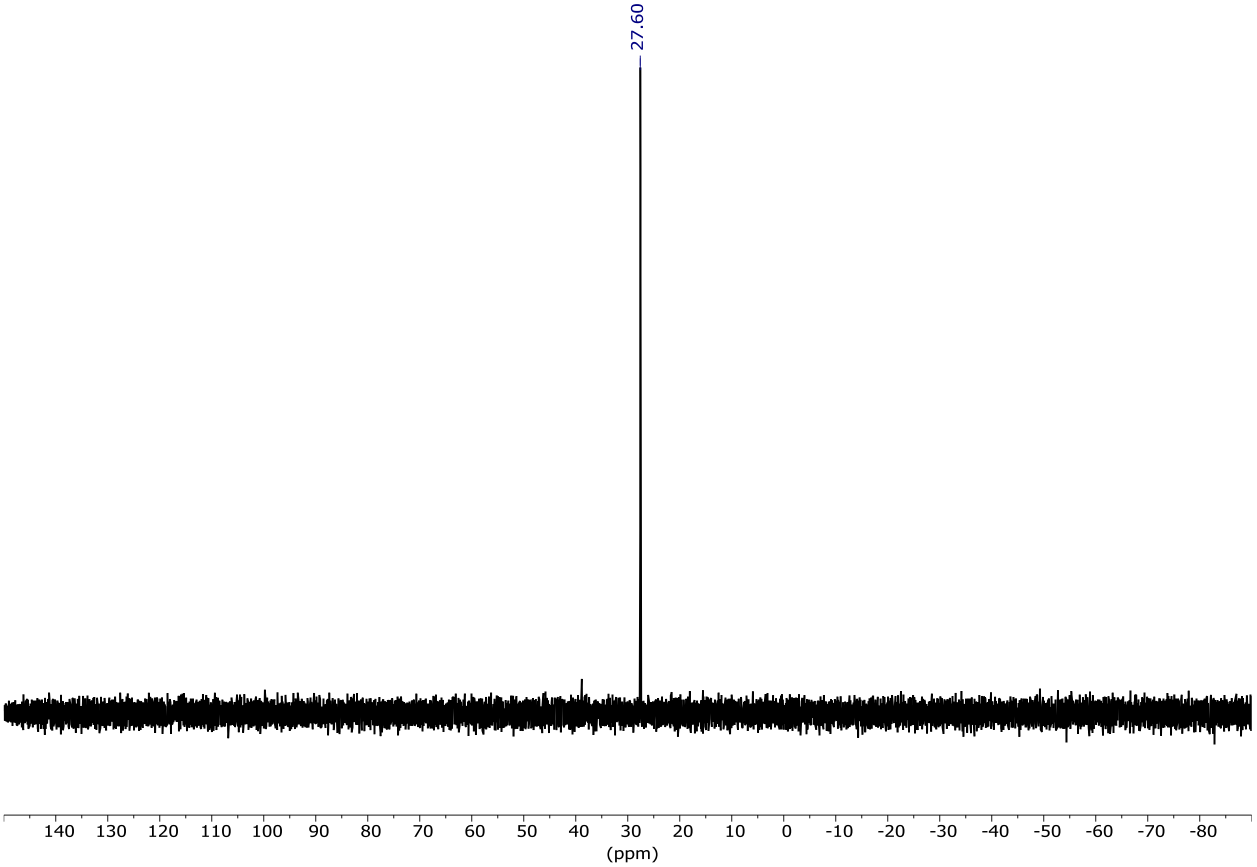

Figure S97. ^{31}P NMR spectrum of **13a**, externally referenced to triphenylphosphine (– 6.5 ppm).

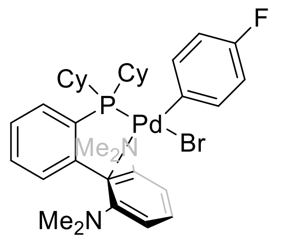

14a

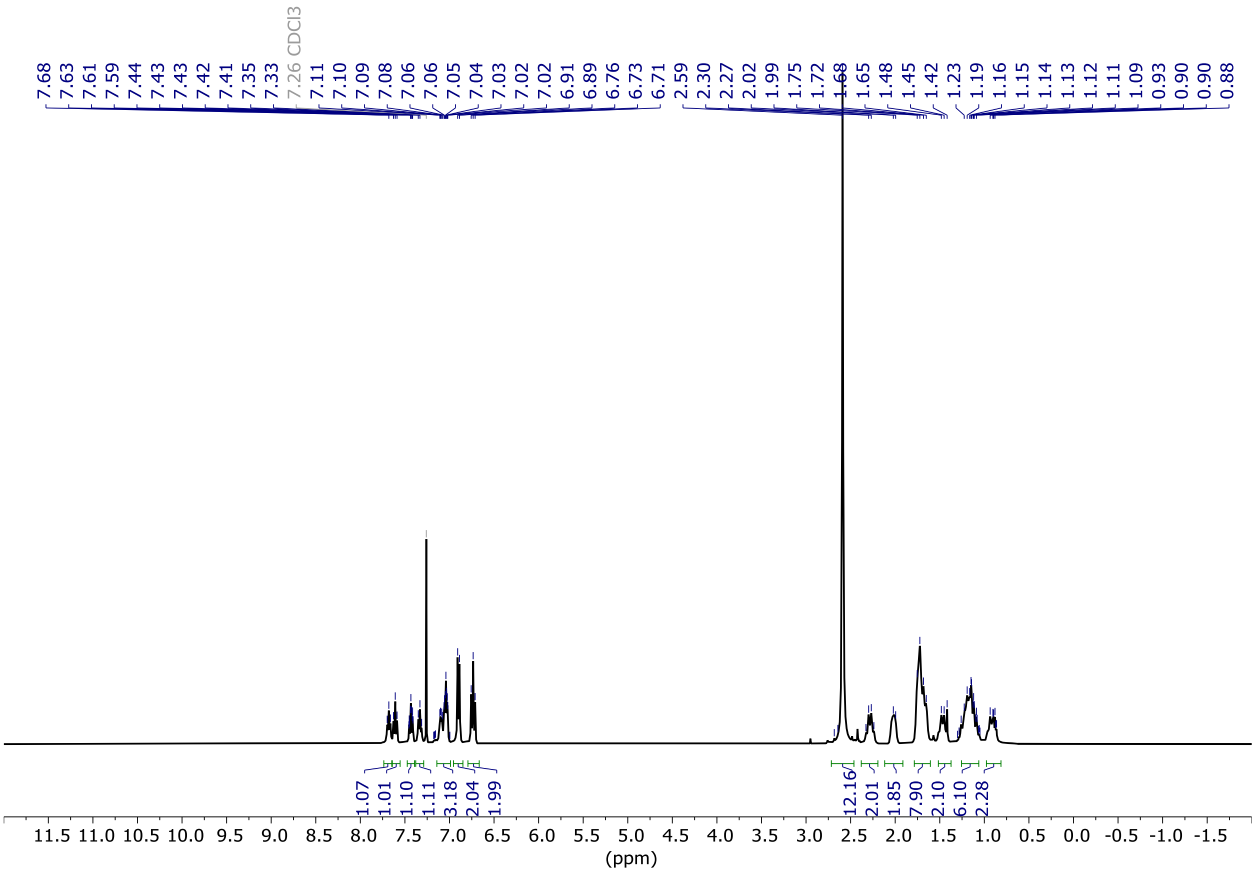

Figure S98. ^{1}H NMR spectrum of **14a**, referenced to $CDCl_3$ (7.26 ppm).

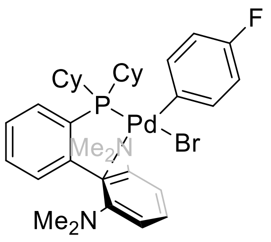

14a

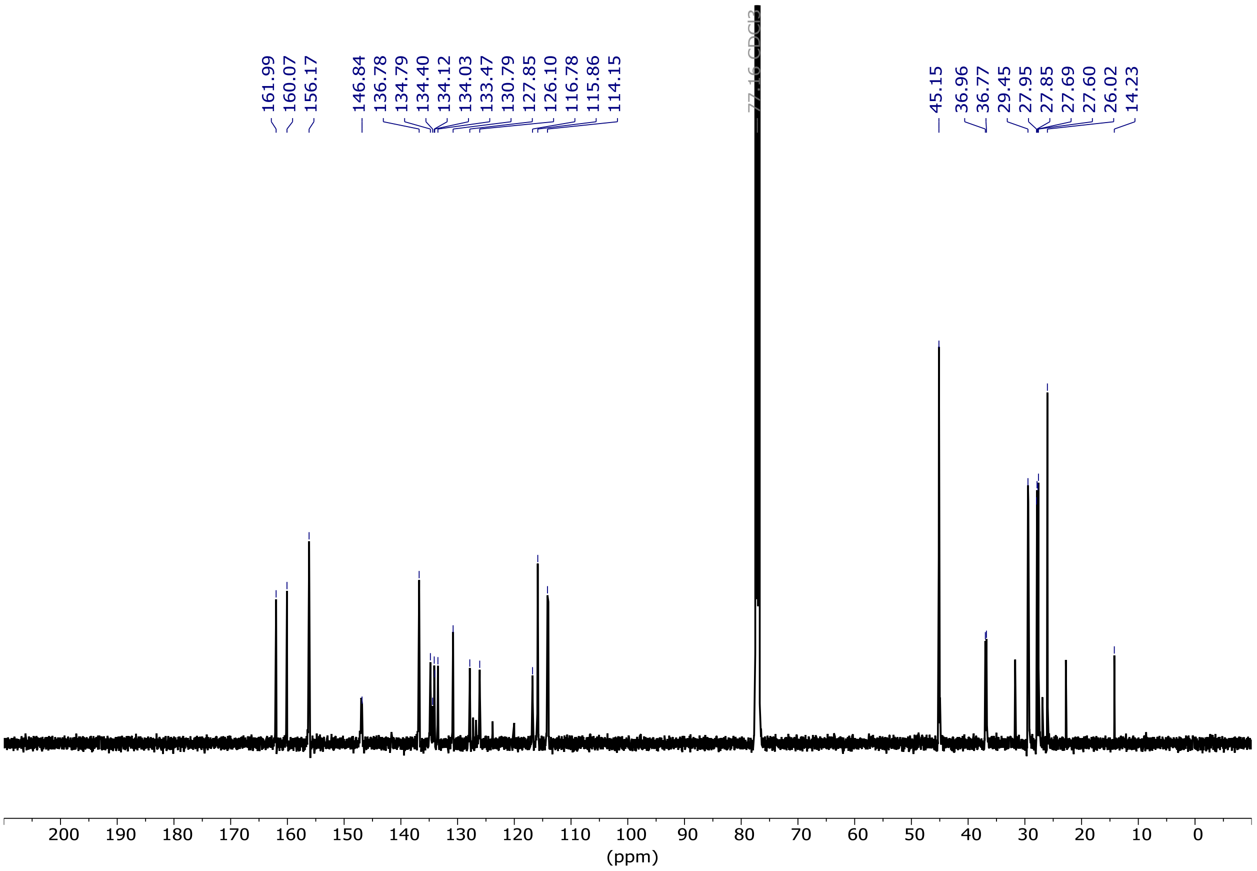

Figure S99. ^{13}C NMR spectrum of **14a**, referenced to $CDCl_3$ (77.16 ppm).

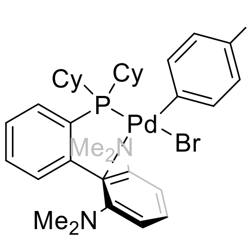

14a

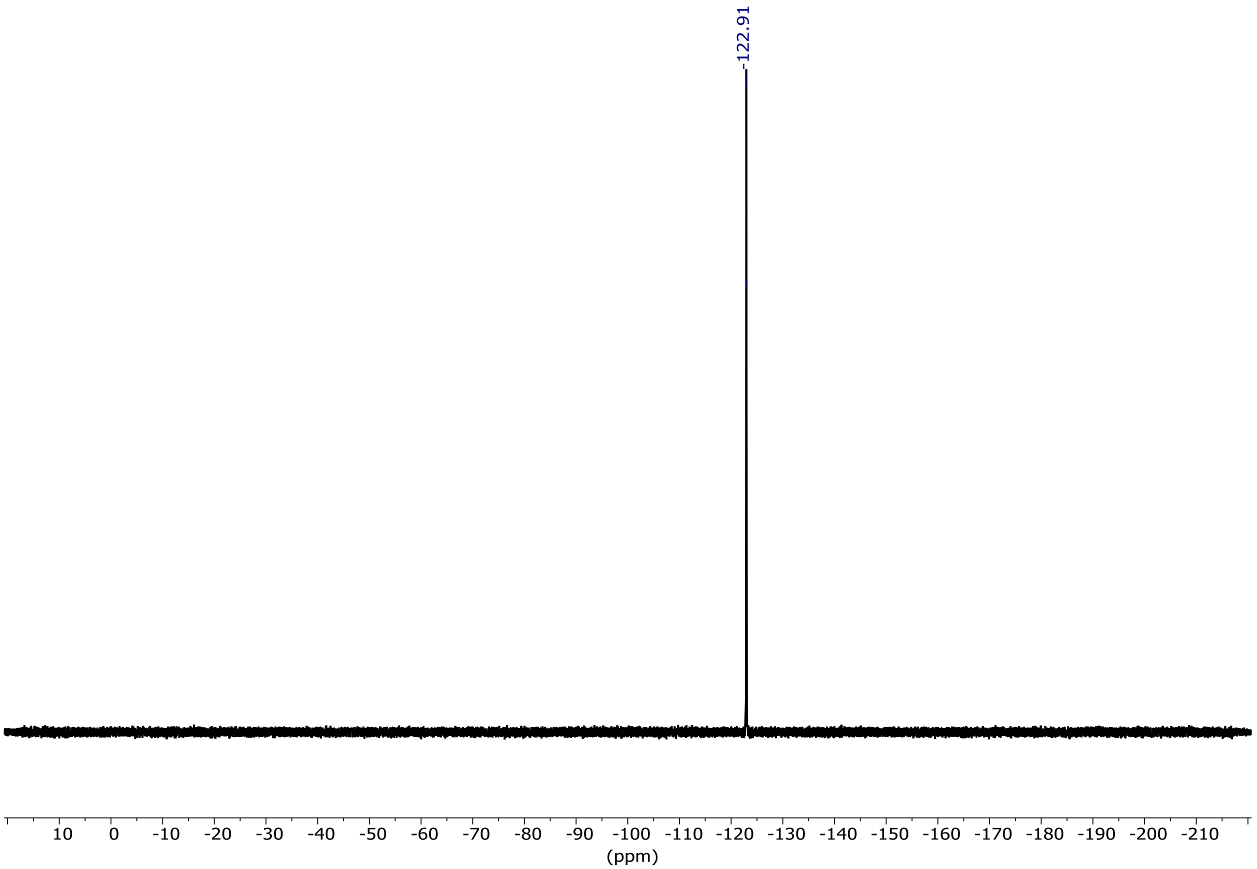

Figure S100. ^{19}F NMR spectrum of **14a**, externally referenced to fluorobenzene (-113.15 ppm).

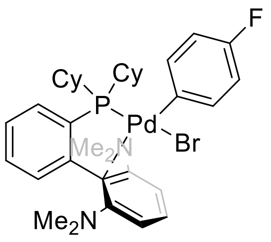

14a

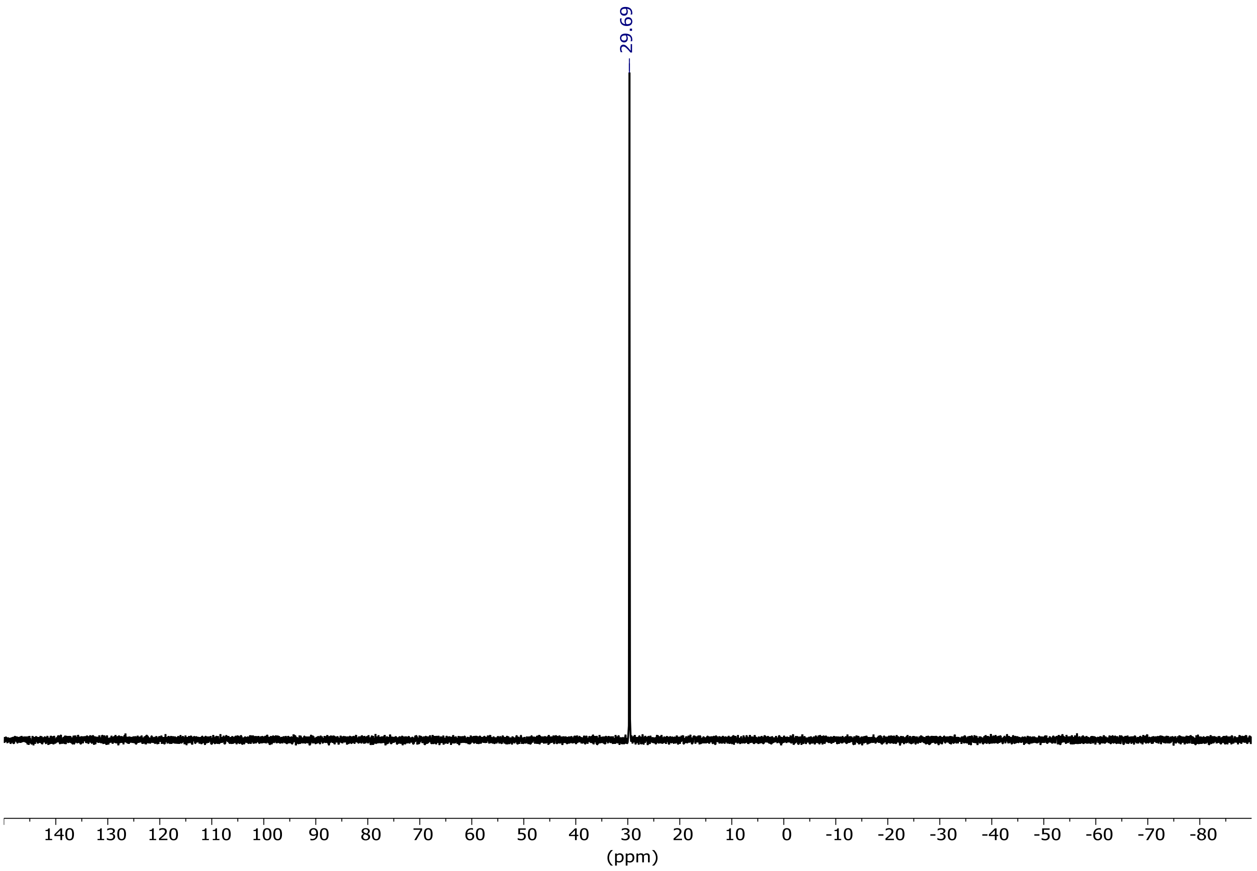

Figure S101. ^{31}P NMR spectrum of **14a**, externally referenced to triphenylphosphine (– 6.5 ppm).

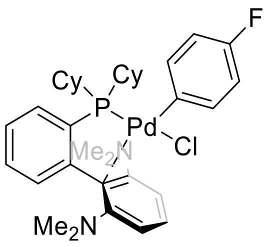

14b

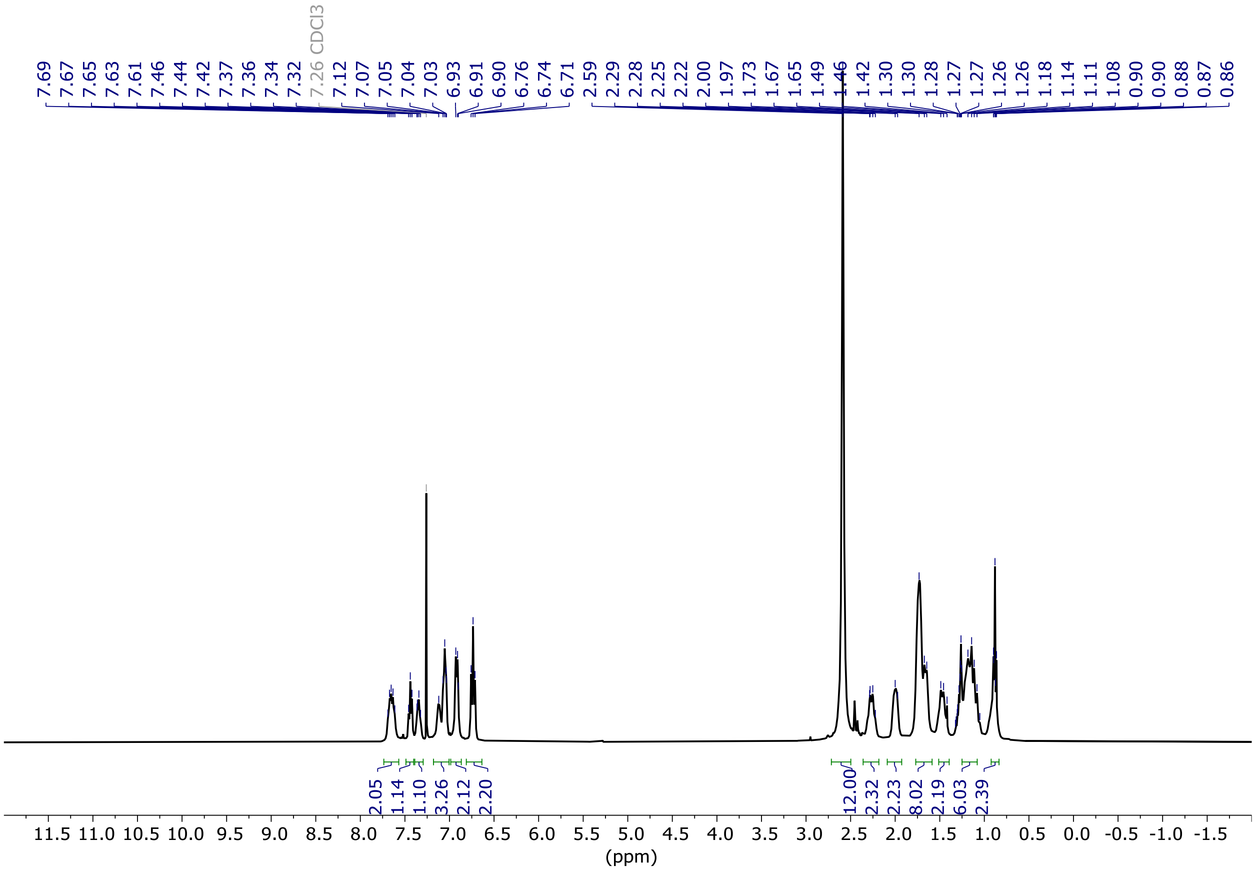

Figure S102. ^{1}H NMR spectrum of **14b**, referenced to $CDCl_3$ (7.26 ppm).

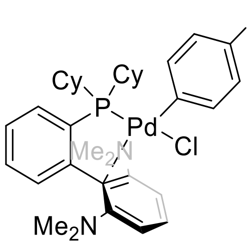

14b

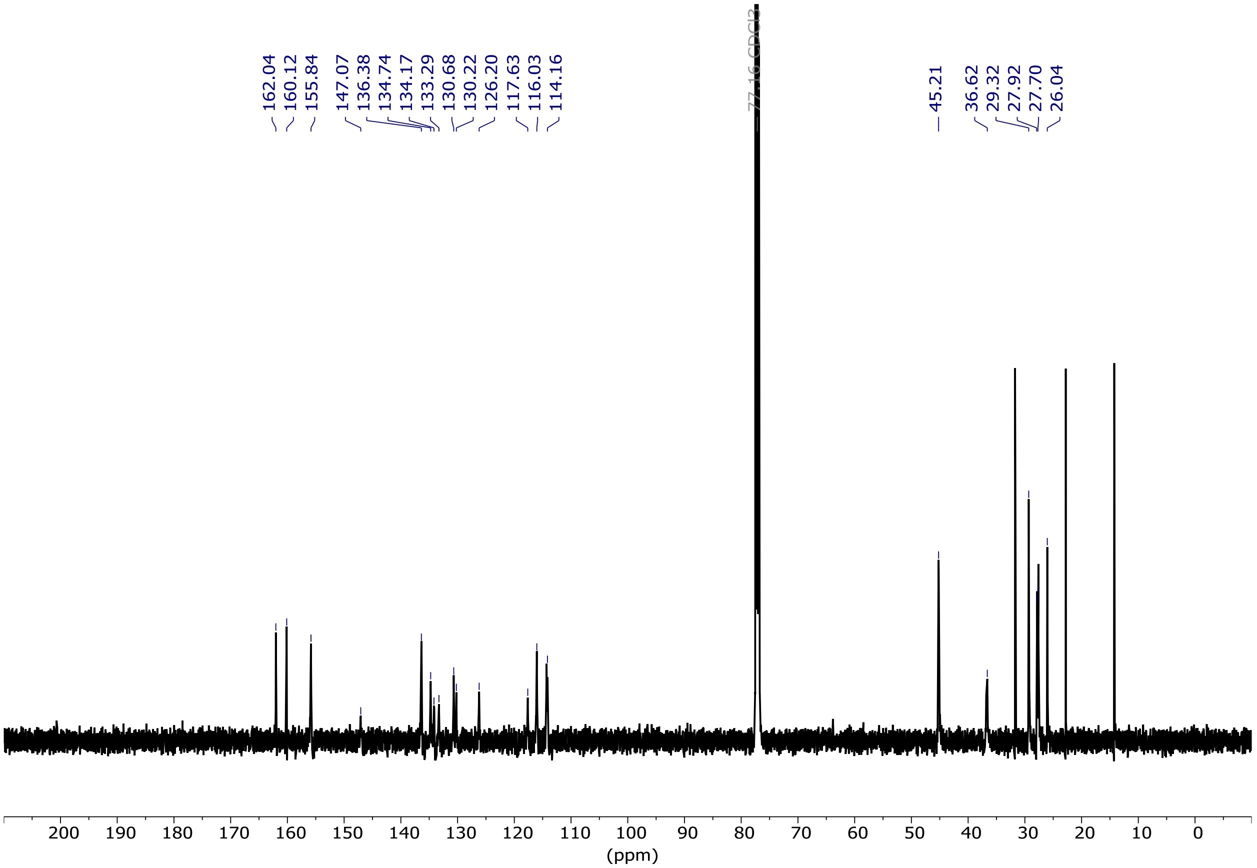

Figure S103. ^{13}C NMR spectrum of **14b**, referenced to $CDCl_3$ (77.16 ppm).

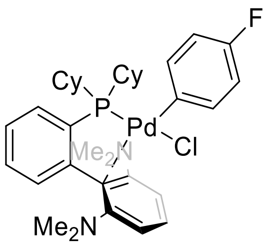

14b

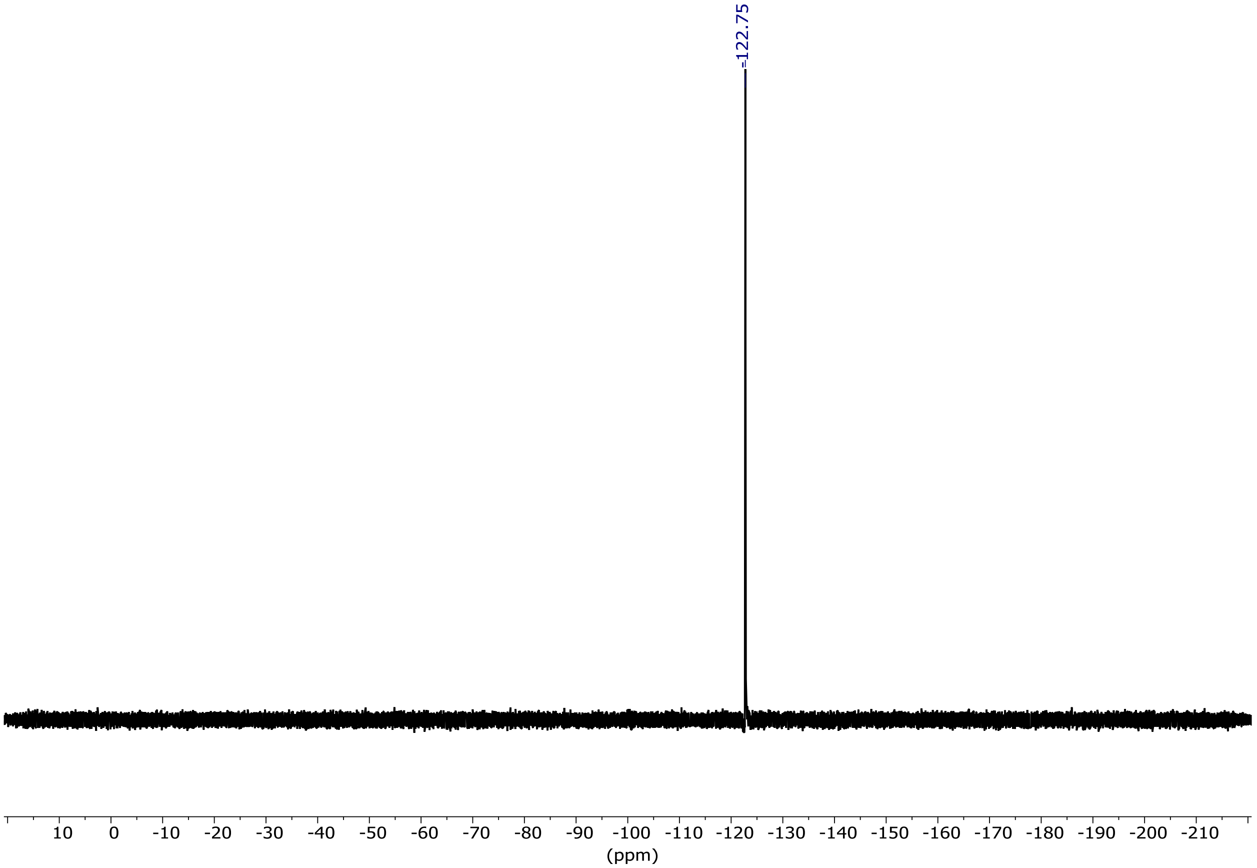

Figure S104. ^{19}F NMR spectrum of **14b**, externally referenced to fluorobenzene (-113.15 ppm).

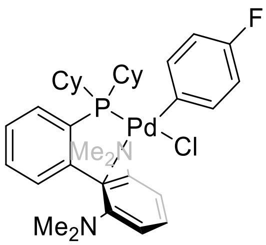

14b

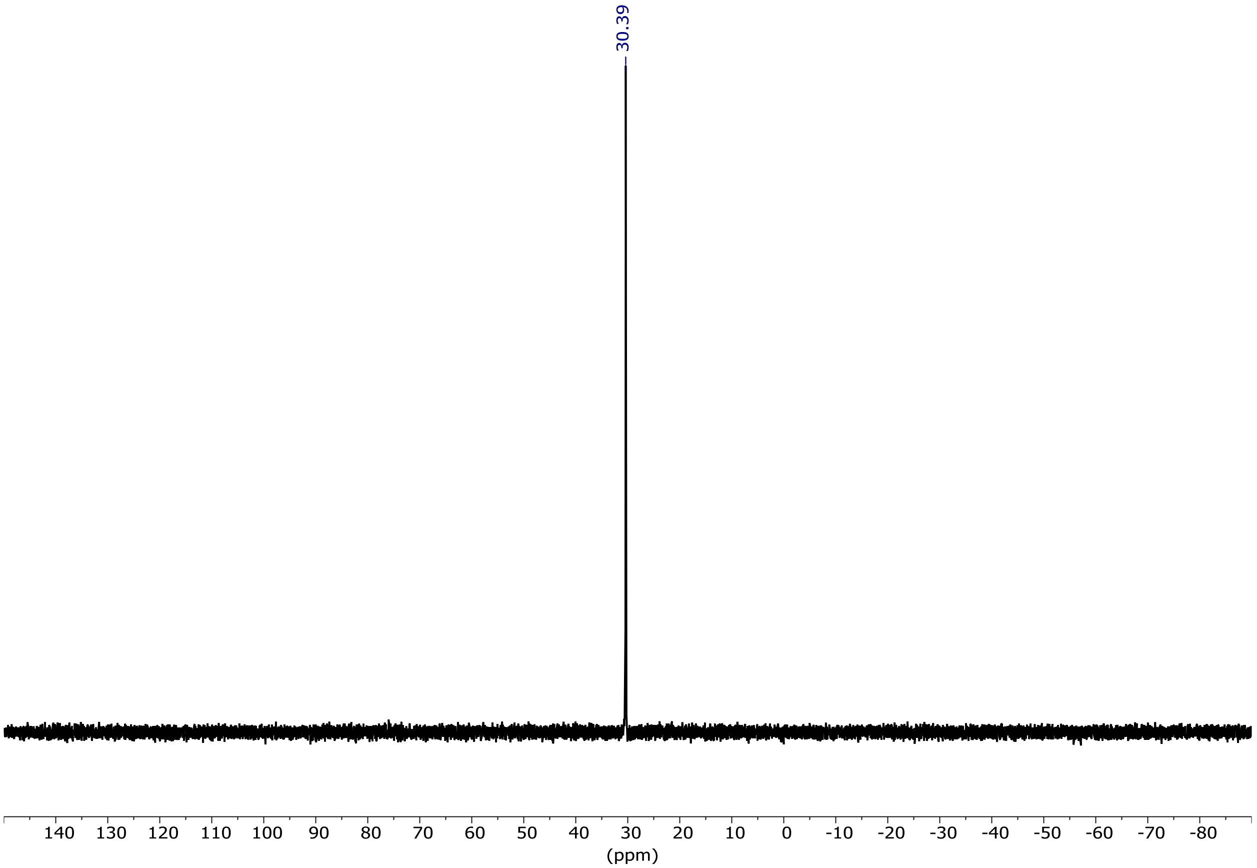

Figure S105. ^{31}P NMR spectrum of **14b**, externally referenced to triphenylphosphine (– 6.5 ppm).

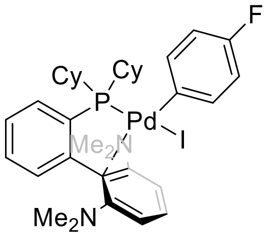

14c

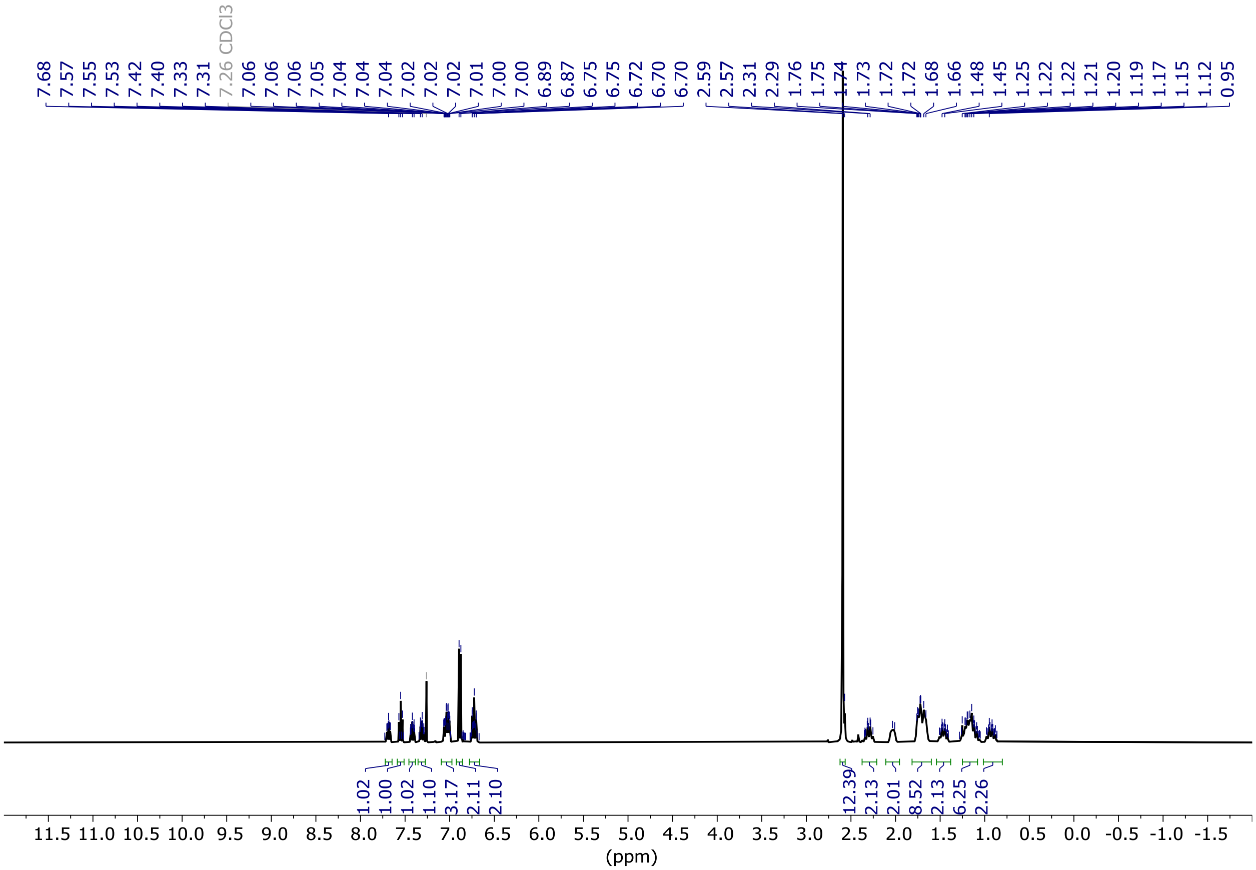

Figure S106. ^{1}H NMR spectrum of **14c**, referenced to $CDCl_3$ (7.26 ppm).

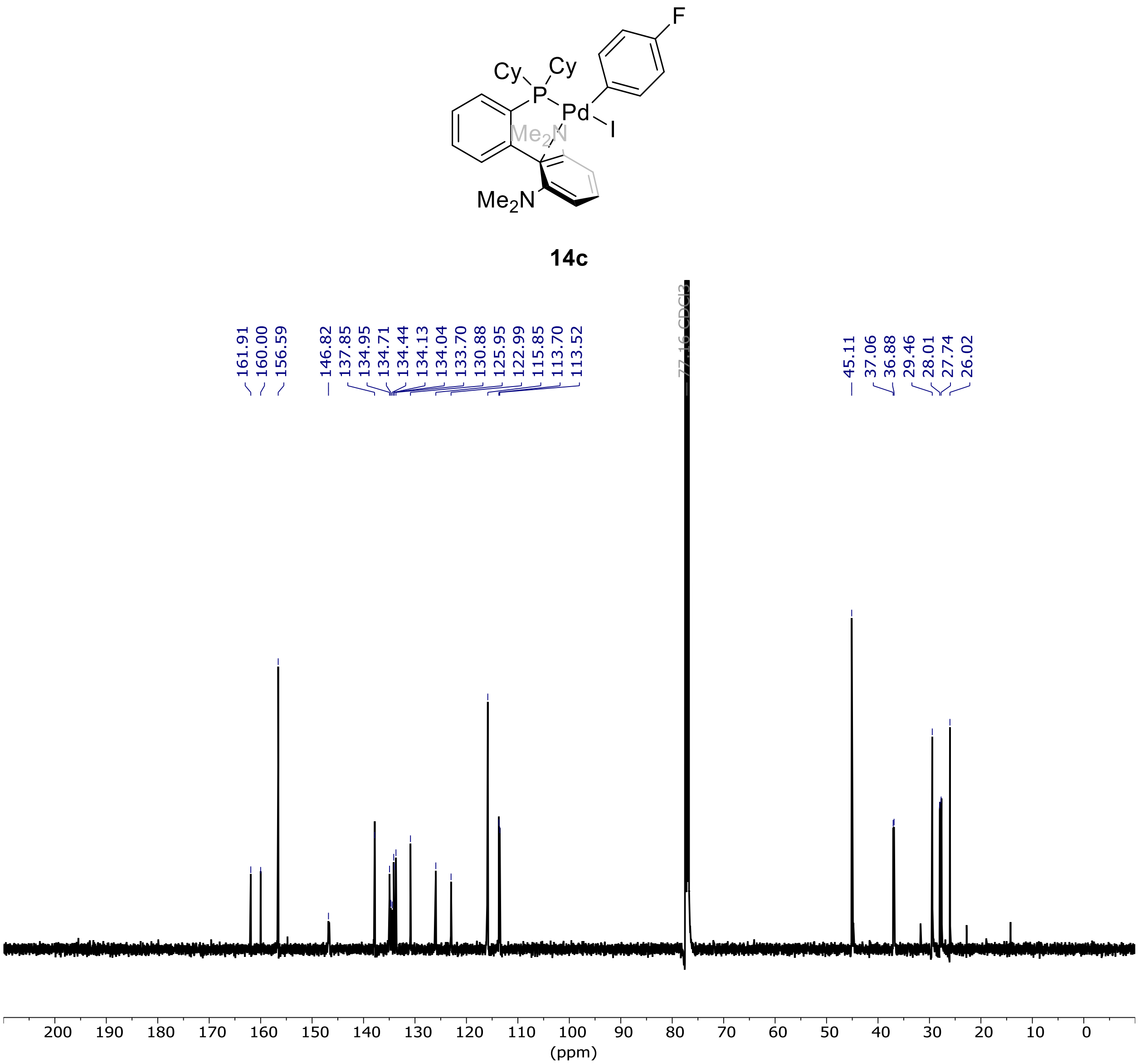

Figure S107. ^{13}C NMR spectrum of **14c**, referenced to $CDCl_3$ (77.16 ppm).

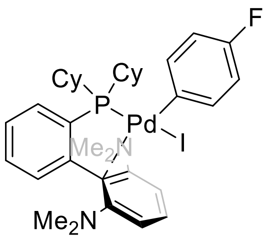

14c

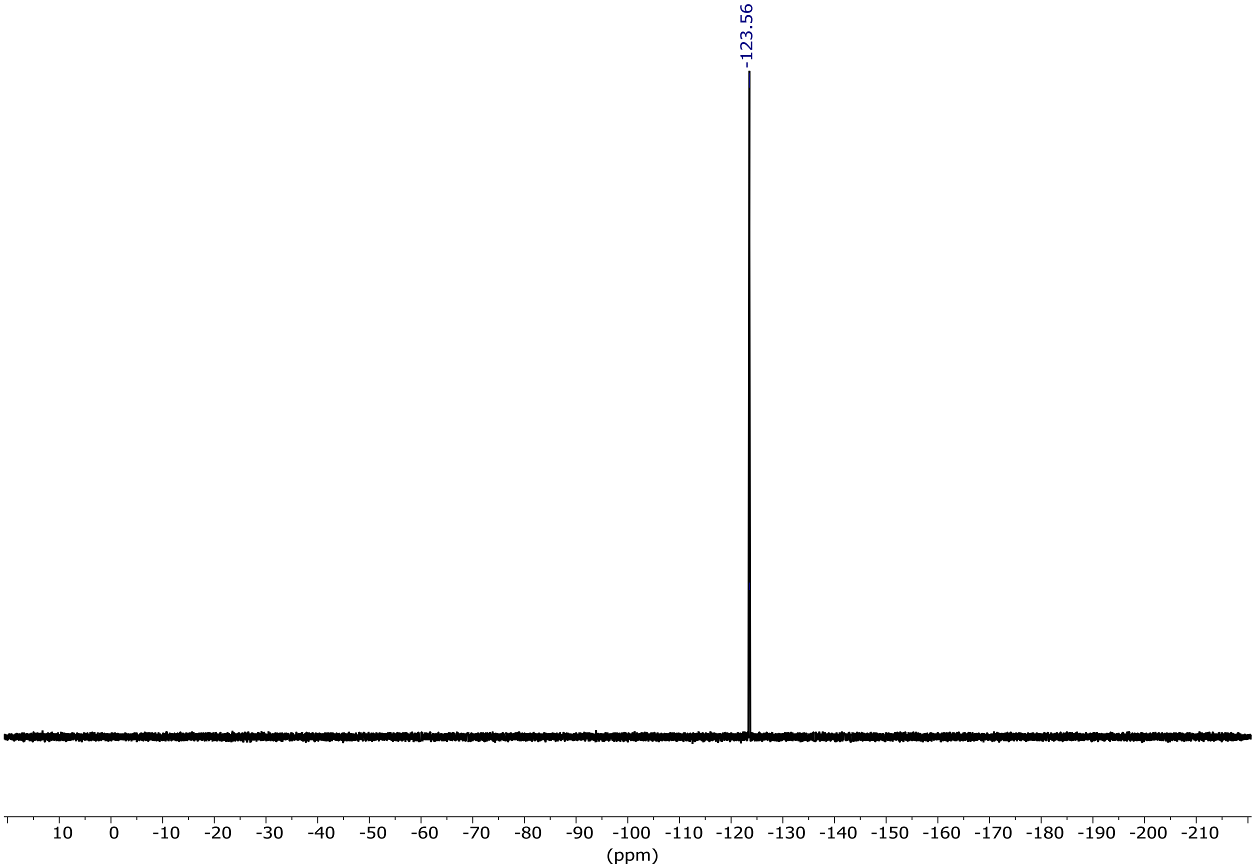

Figure S108. ^{19}F NMR spectrum of **14c**, externally referenced to fluorobenzene (-113.15 ppm).

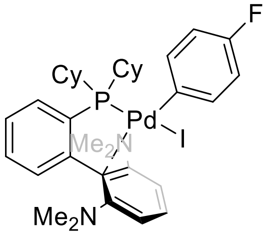

14c

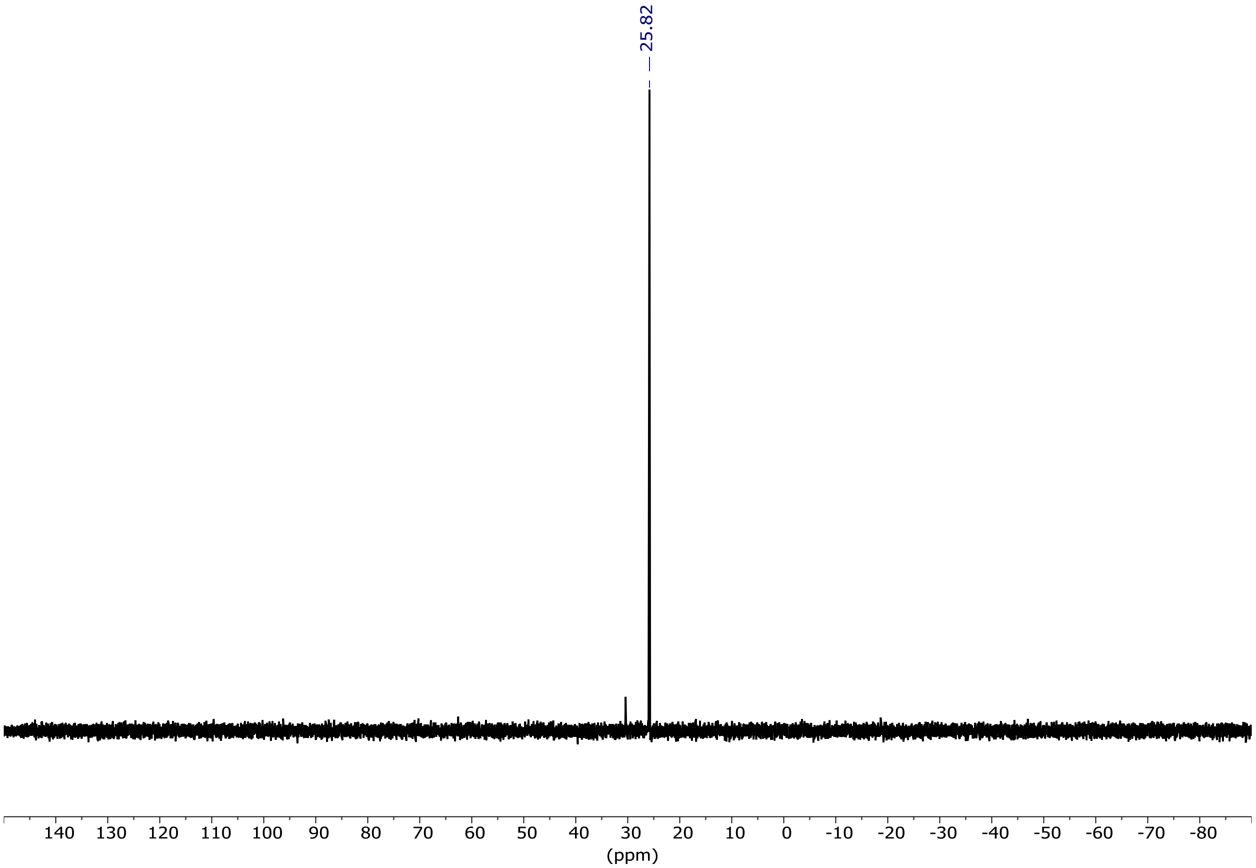

Figure S109. ^{31}P NMR spectrum of **14c**, externally referenced to triphenylphosphine (– 6.5 ppm).

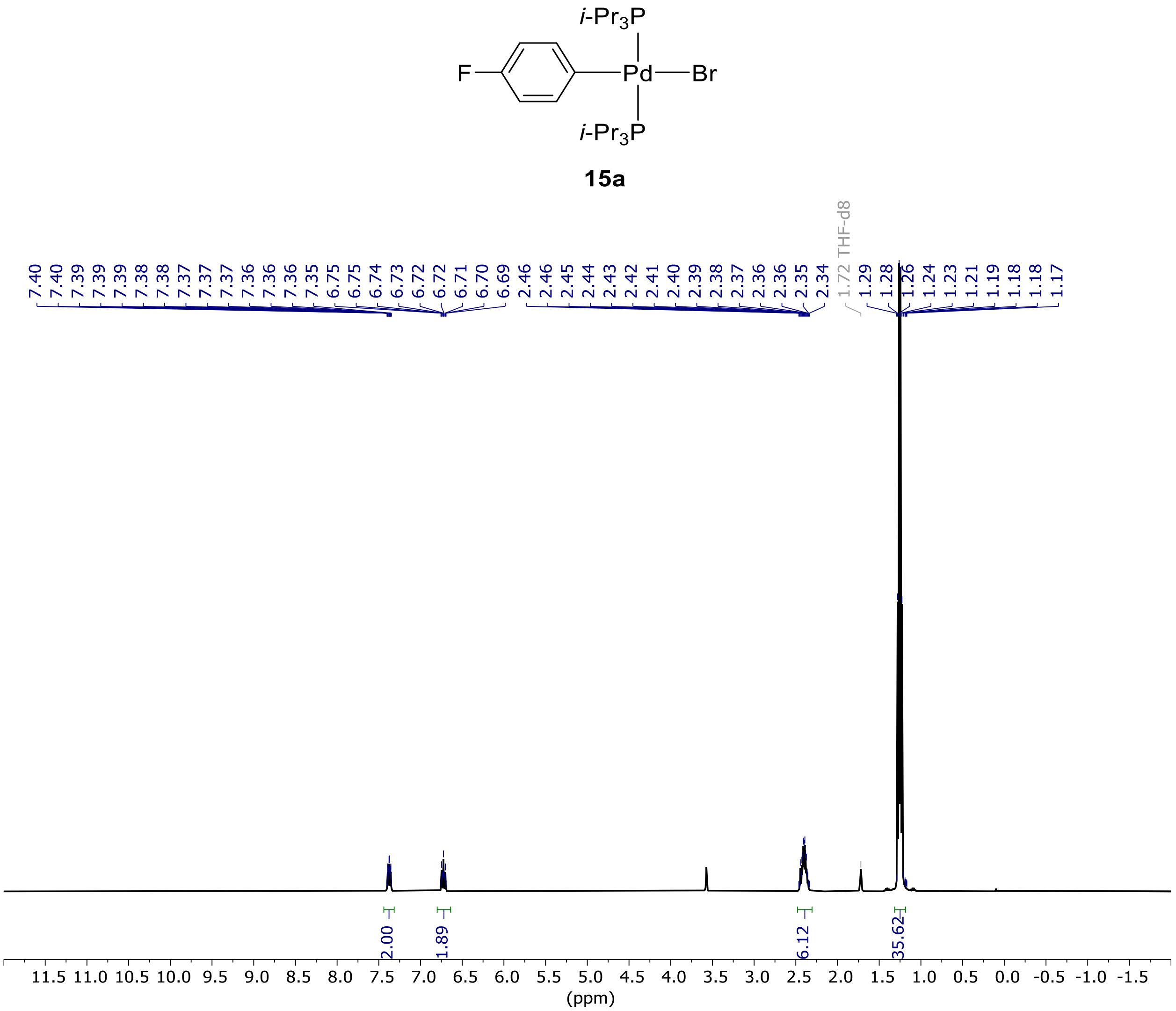

Figure S110. ^{1}H NMR spectrum of **15a**, referenced to THF-d_8 (1.72 ppm).

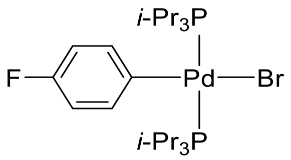

15a

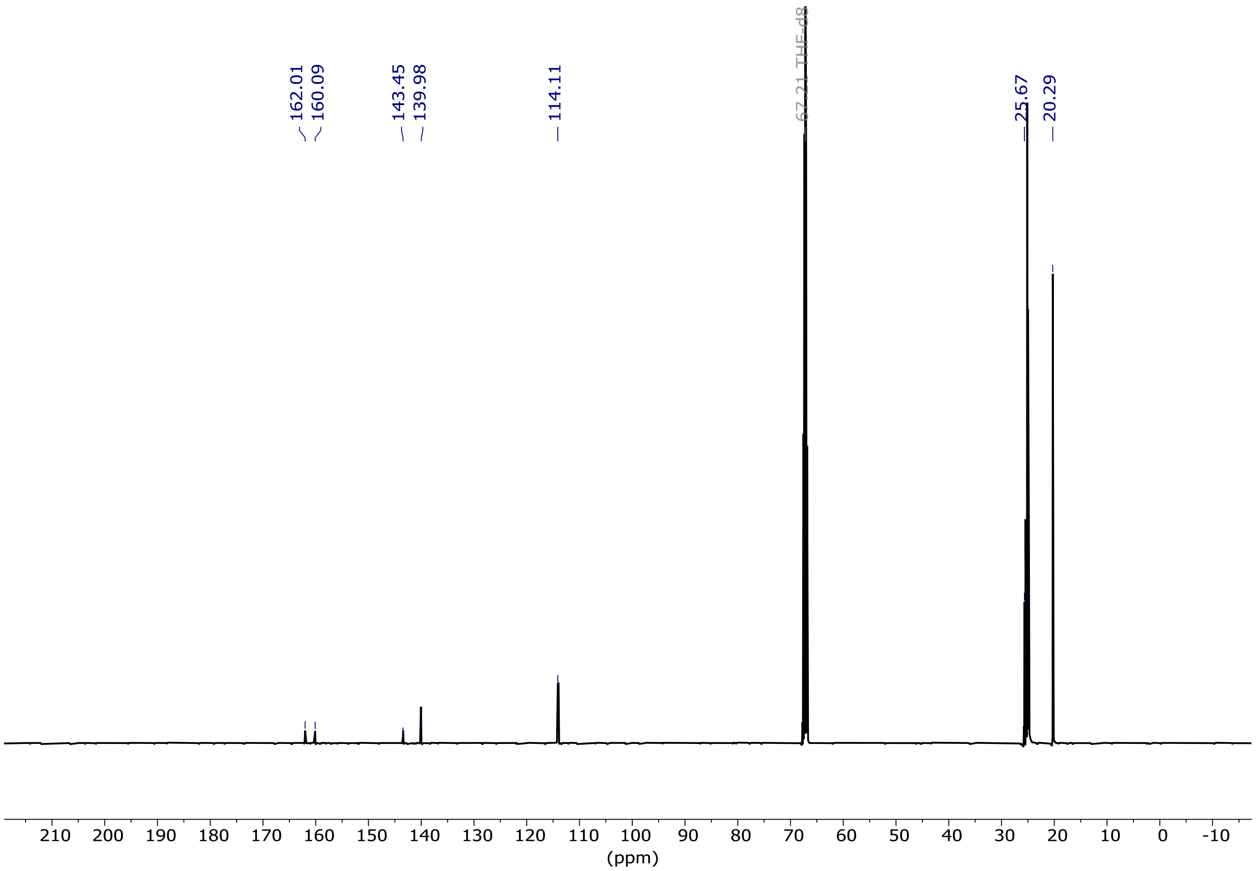

Figure S111. ^{13}C NMR spectrum of **15a**, referenced to THF-d_8 (67.21 ppm).

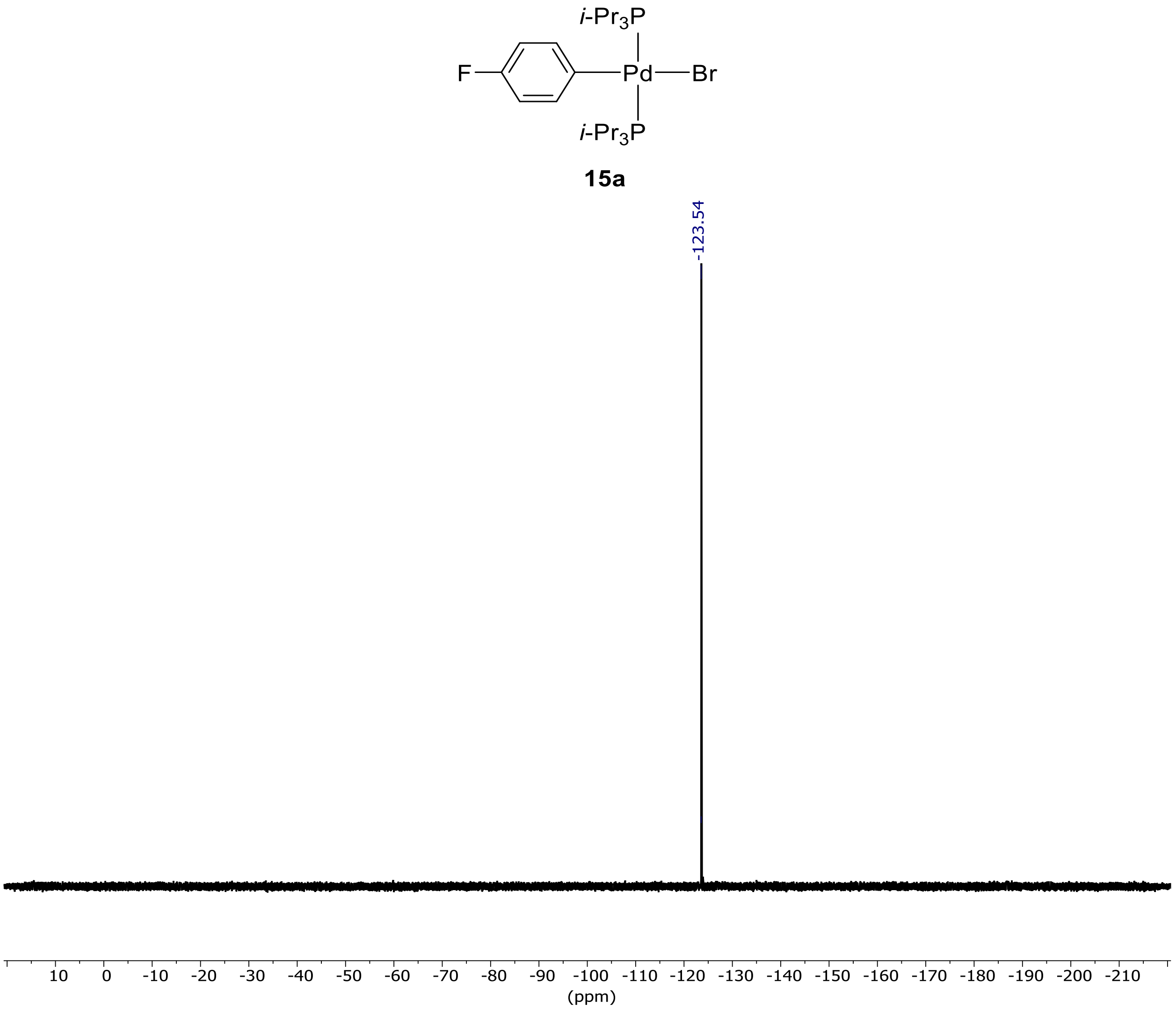

Figure S112. ^{19}F NMR spectrum of **15a**, externally referenced to fluorobenzene (-113.15 ppm).

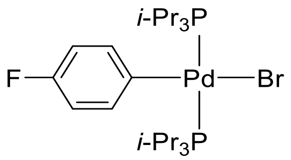

15a

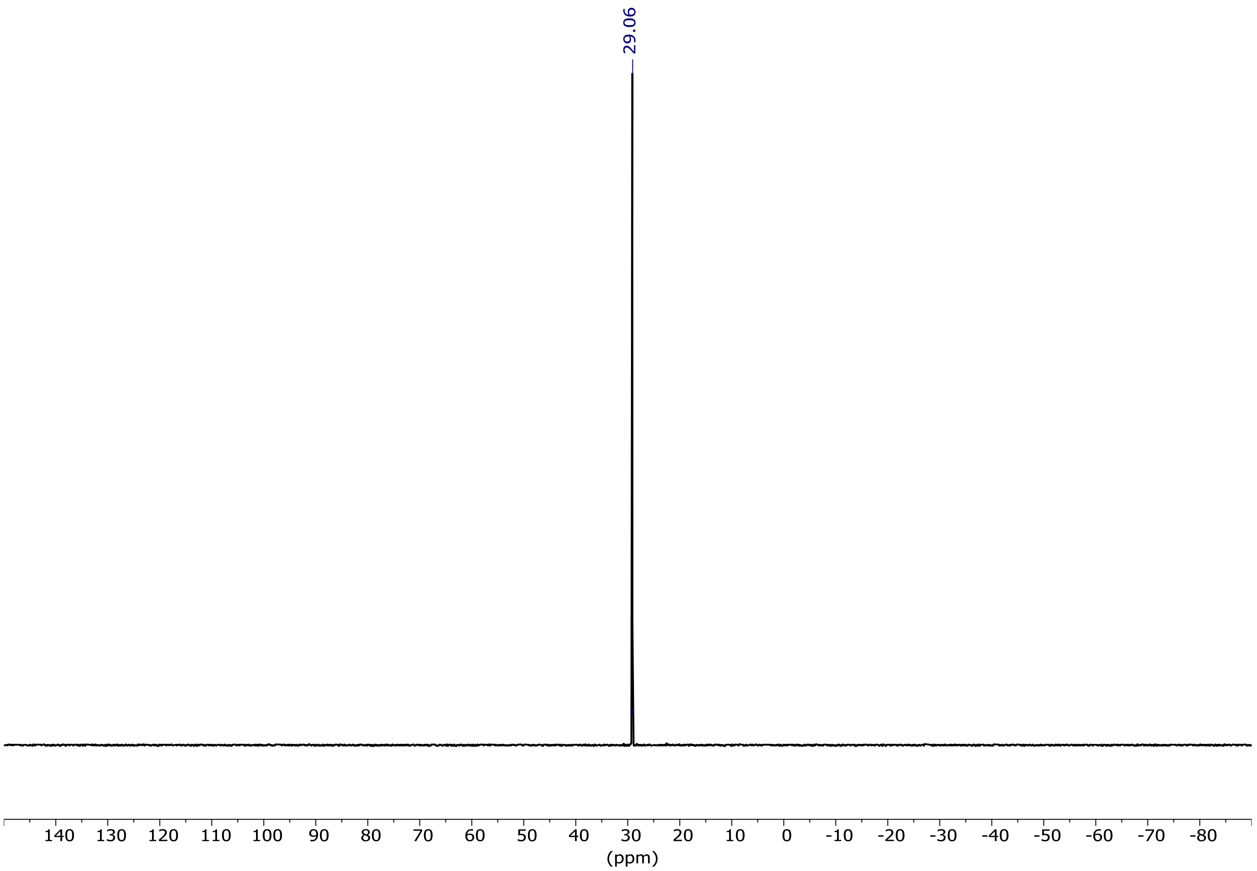

Figure S113. ^{31}P NMR spectrum of **15a**, externally referenced to triphenylphosphine (– 6.5 ppm).

4. Computational Studies

All geometry optimizations of intermediates and transition states were achieved using spin restricted M06L[viii]-D3[ix]/6-31G(d,p)[x]-LANL2DZ(Pd,Br)[xi] method as implemented in Gaussian16.[xii] Moreover, the solvation effects were considered with THF as the solvent used experimentally using the CPCM solvent model.[xiii] Frequency calculations were also conducted at the same level of theory to obtain vibrational frequencies to determine the identity of stationary points as intermediates (no imaginary frequencies) or transition states (only one imaginary frequency), as well as obtain thermal correction to enthalpy and free energy at 298 K. Intrinsic Reaction Coordinate (IRC) calculations were done on the transition states to verify the correct transition state associated with the reaction. The endpoint geometries obtained from the IRC calculations were further optimized to verify the authenticity of the transition state. Also, an extensive conformational search was performed for all the intermediates and transition states and only the lowest-energy species were shown and discussed. Finally, we performed single-point calculations on the optimized geometries using the following levels of theory:

1. RM06L-D3/6-311+g(d,p)-SDD(Pd,Br)-CPCM(THF)//RM06L-D3/6-31g(d,p)-LANL2DZ(Pd,Br)-CPCM(THF)

2. RM06L-D3/def2tzvpp-CPCM(THF)//RM06L-D3/6-31g(d,p)-LANL2DZ(Pd,Br)-CPCM(THF)

All structural figures were generated with CYLview.[xiv] Distances in structural figures are shown in Å and energies are in kcal/mol. NBO analyis was done using Gaussian NBO Version 3.1.[xv] Conceptual density functional theory was used to electrophilicty indices using on Multiwfn.[xvi]

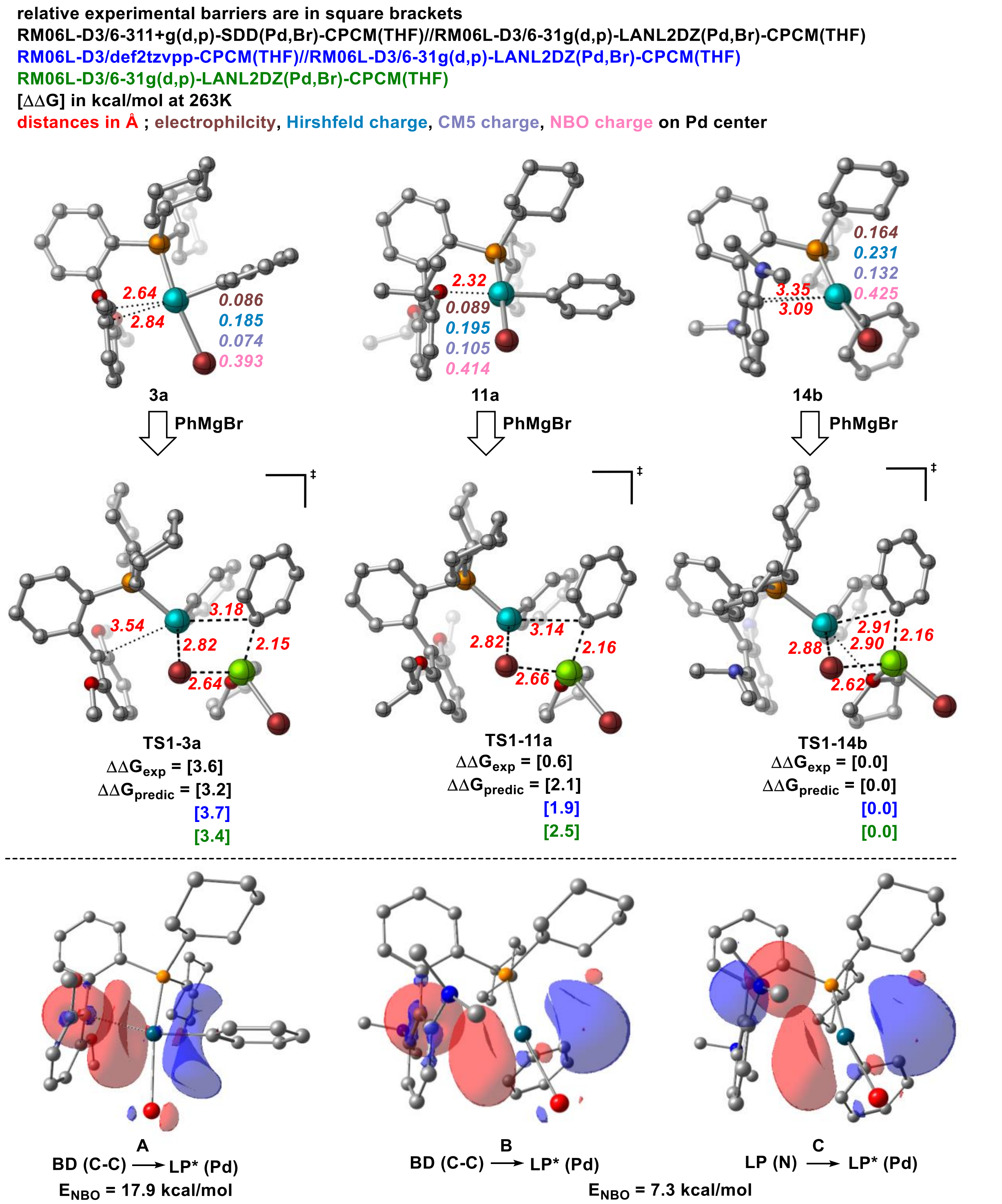

Figure S114. (Top) Atomic charge and electrophilicity analysis for the SPhos, RuPhos and CPhos ligated oxidative addition complexes. (Middle) Transmetalation transition states for SPhos, RuPhos and CPhos ligated system calculated at different levels of theory. (Bottom) Key NBO interactions between ligand and Pd center in the SPhos system (A) and in the CPhos system (B,C).

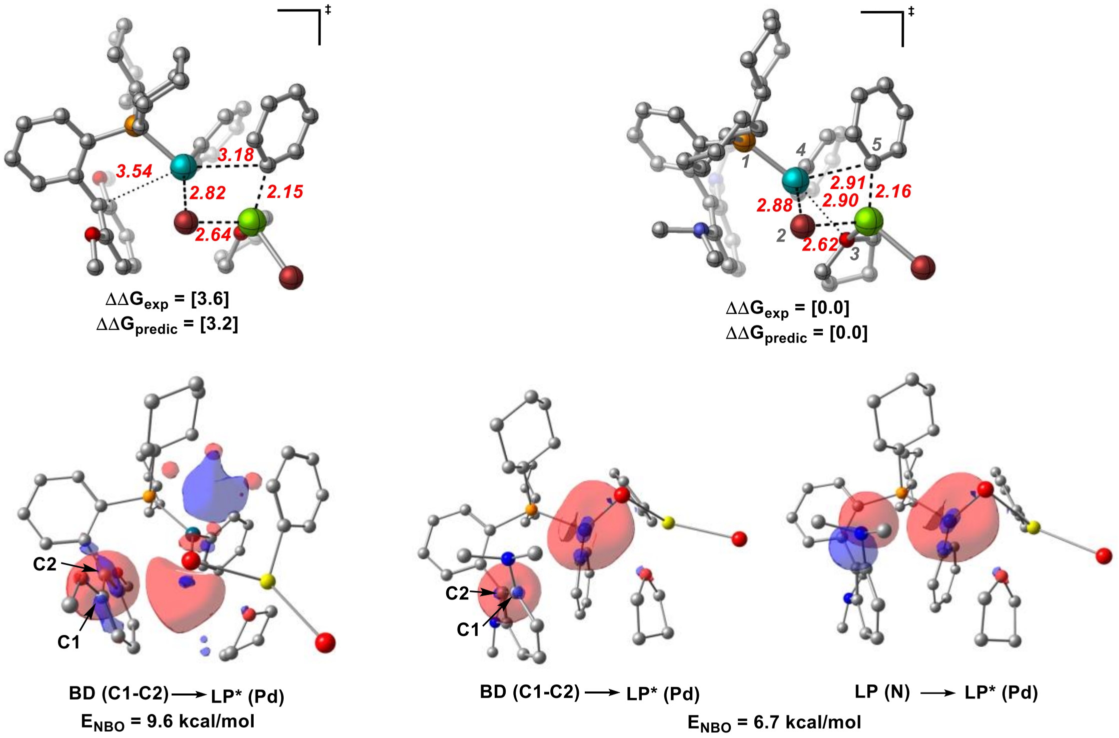

Figure S115. Transmetalation transition state for SPhos (left) and CPhos (right) system along with their key NBO interactions; For SPhos system (E_{NBO} = 9.6 kcal/mol) and For CPhos system (6.7 kcal/mol).

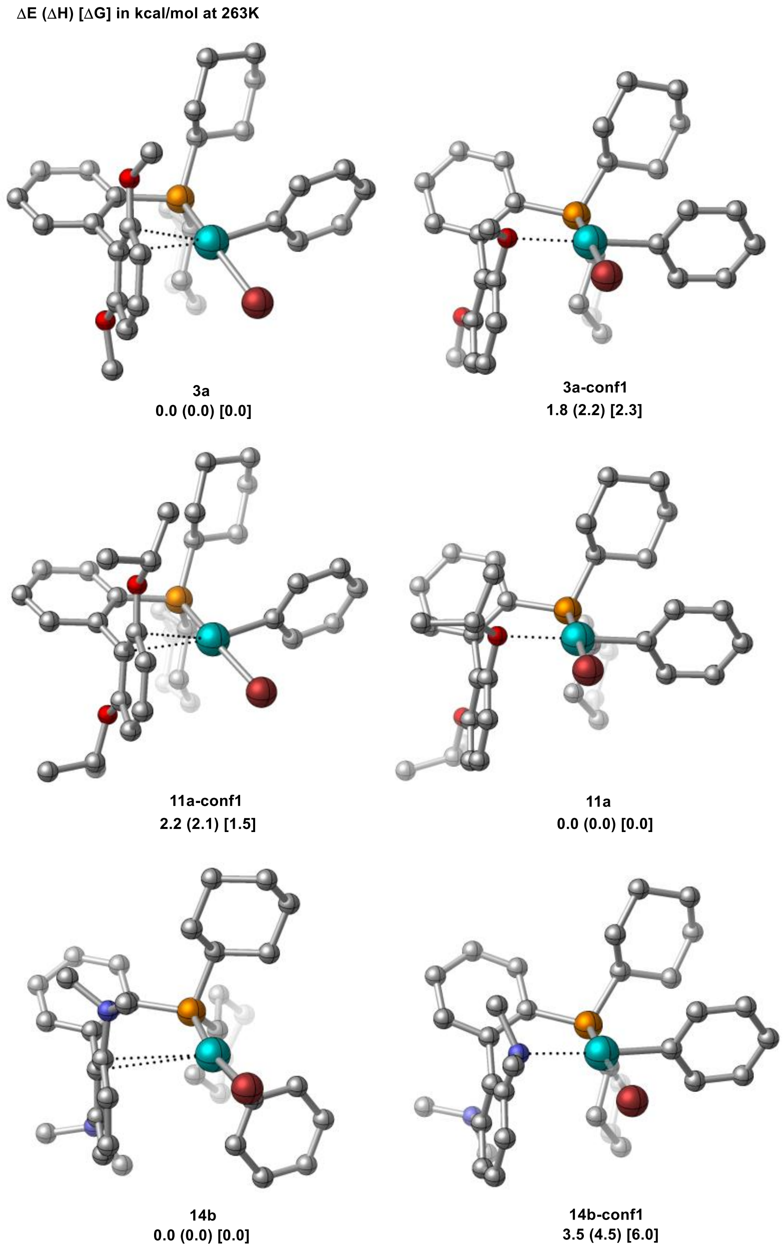

Figure S116. Different conformations for the oxidative addition complexes for SPhos, RuPhos and CPhos.

In the SPhos system, the relatively low steric demand allows the ligand to coordinate through the aromatic C–C bond, while coordination via the oxygen atom is energetically disfavored. In contrast, RuPhos exhibits substantial steric congestion in proximity to the oxygen substituent, and the oxygen atom carries a more negative charge (NBO: –0.603 for RuPhos vs. –0.534 for SPhos). These factors collectively promote Pd–O coordination as the preferred binding mode. For CPhos, the amine nitrogen bears the lowest negative charge (among the three ligand classes considered) (NBO: –0.519); combined with steric constraints, CPhos disfavor Pd–N coordination. Instead,

CPhos preferentially binds through its aromatic C–C bond, with the N-coordinated conformation calculated to be significantly higher in energy.

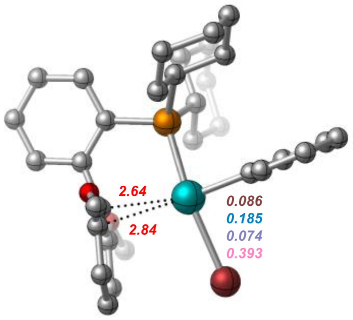

3a

E_{NBO} = 17.9 kcal/mol

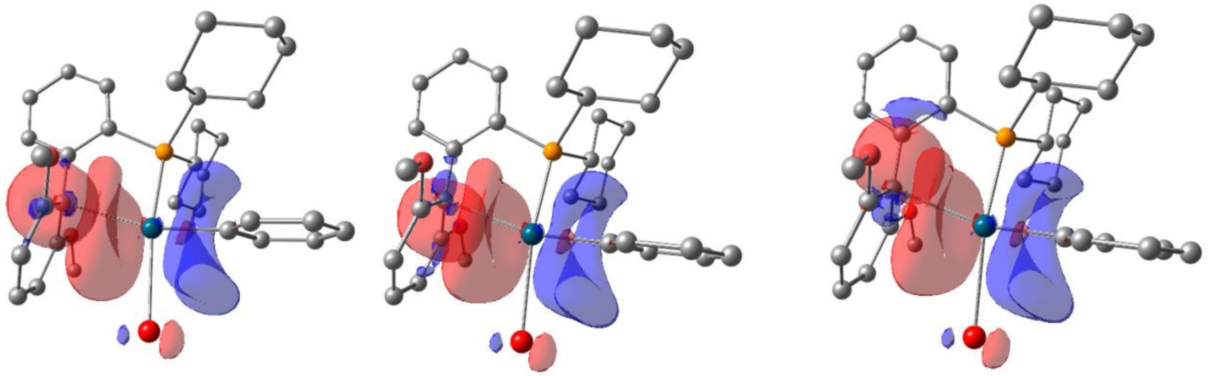

BD (C-C)--> LP* (Pd) BD (C-C)--> LP* (Pd) BD (C-C)--> LP* (Pd)

Figure S117. Key NBO interactions between aromatic C-C bond to the empty d orbital of Pd. Total NBO interaction energy is 17.9 kcal/mol.

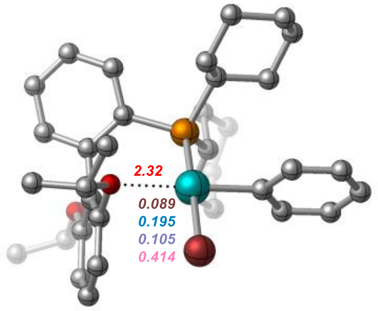

11a

E_{NBO} = 23.8 kcal/mol

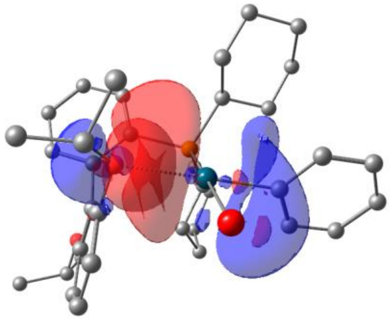

LP (O)--> LP* (Pd)

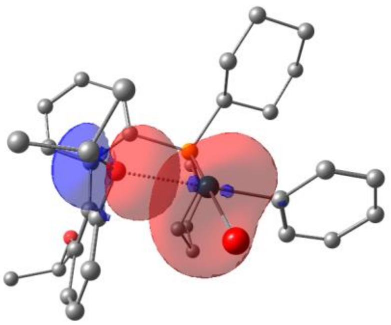

LP (O)--> LP* (Pd)

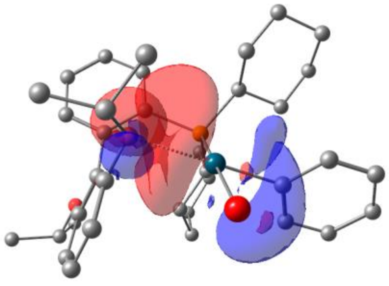

LP (O)--> LP* (Pd)

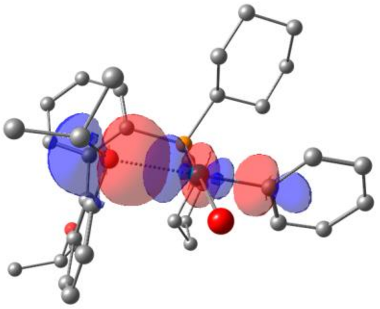

LP (O)--> BD* (Pd-C)

E_{NBO} = 12.0 kcal/mol

Figure S118. Key NBO interactions between O lone pair to the empty d orbital of Pd.

Total NBO interaction energy is 23.8 kcal/mol. In RuPhos system, the lone pair of oxygen is strongly interacting with the Pd atom and this interaction is even stronger than that in SPhos system. However the lone pair of O also donates its electron density to the antibonding orbital of Pd-C (aromatic) bond, thus weakening the Pd-C bond. This donation of electron density to the antibonding orbital of Pd-C bond results in polarization of the Pd-C bond and this polarization in turn enhances the positive charge in the Pd atom.

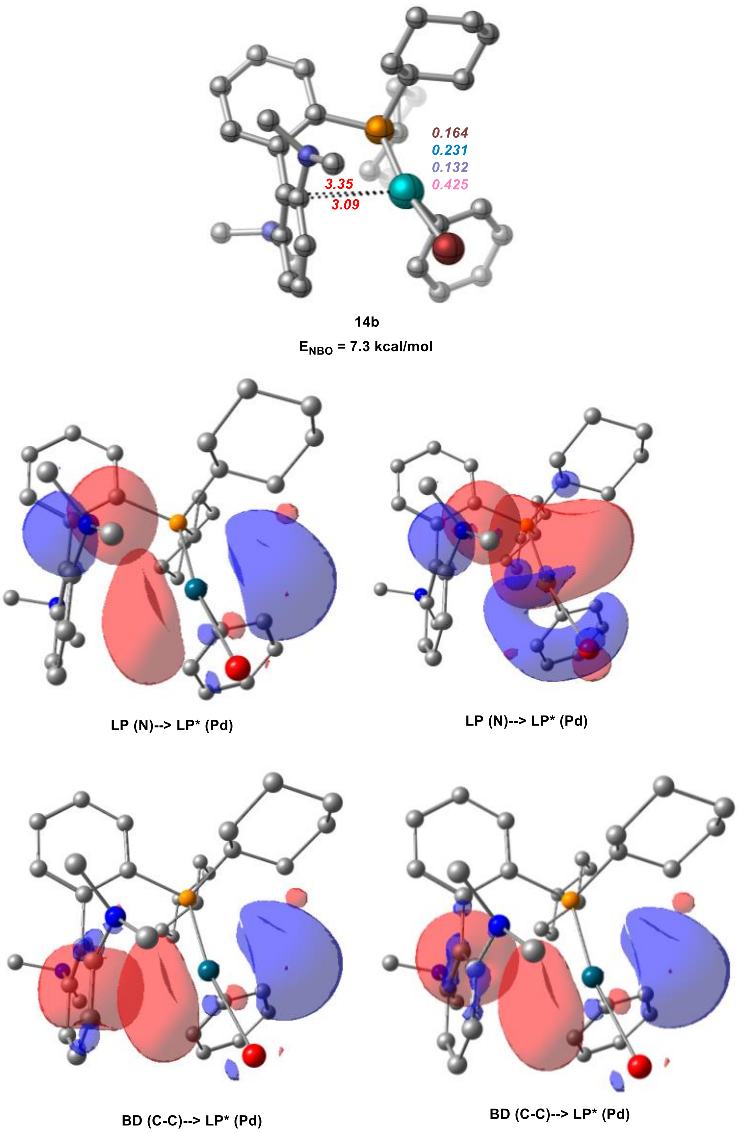

Figure S119. Key NBO interactions between N lone pair and aromatic C-C bond to the empty d orbital of Pd. Total NBO interaction energy is 7.3 kcal/mol.

In the CPhos system, the electron donation into the empty d orbital of Pd from CPhos ligand is the weakest, thus resulting in highest positive charge and highest electrophilicity for the CPhos system.

Table S99. Cartesian coordinates (XYZ format) of all the structures calculated at the B3LYP-D3/def2svp-SDD(Ru)-CPCM(benzene), and single-point energies calculated at M06L-D3/def2tzvpp-SDD(Ru)-SMD(benzene).

THF

E(scf) = --232.422262739 a.u.

ν_{min} = 61.6529 cm^{-1}

C	1.255693	-1.170453	2.152853	H	2.466694	0.438748	-0.279546
O	1.804876	0.043216	1.632869	H	3.719375	-0.118067	0.846721
C	2.671675	-0.262514	0.537441	H	1.549490	-1.788909	-0.503240
C	2.407385	-1.713379	0.174420	H	3.261482	-2.193604	-0.308603
C	2.057957	-2.302615	1.535212	H	1.499110	-3.239685	1.480453
H	1.309586	-1.140675	3.247132	H	2.970018	-2.486575	2.114228
H	0.192485	-1.241955	1.872514				

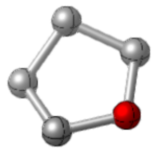

Zero-point correction= 0.116808 (Hartree/Particle)

Thermal correction to Energy= 0.120838

Thermal correction to Enthalpy= 0.121671

Thermal correction to Gibbs Free Energy= 0.092522

Sum of electronic and zero-point Energies= -232.305454

Sum of electronic and thermal Energies= -232.301425

Sum of electronic and thermal Enthalpies= -232.300592

Sum of electronic and thermal Free Energies= -232.329741

RM06L-D3/6-311g(d,p)-SDD(Pd,Br)-CPCM(THF)//RM06L-D3/6-31g(d,p)-LANL2DZ(Pd,Br)-CPCM(THF) = -232.479819

11a

E(scf) = -2032.48703474

ν_{min} = 19.98 cm^{-1}

Br	2.059408	0.894149	-2.916894	C	1.196714	5.587880	-0.779198
Pd	0.635430	2.109234	-1.036547	C	-0.221270	5.860422	-0.427299
C	0.796797	0.505758	0.130265	C	-0.662038	7.169964	-0.666715
C	1.598785	0.496033	1.272327	C	-1.987275	7.545441	-0.497876
C	0.114149	-0.656474	-0.243630	C	-2.913219	6.595607	-0.083143
C	1.692915	-0.660018	2.052496	C	-2.494063	5.297796	0.185218
H	2.150544	1.386015	1.566806	C	-1.153645	4.901633	0.043002
C	0.210289	-1.806448	0.540674	P	-0.660801	3.192475	0.548165
H	-0.502691	-0.665959	-1.139703	C	0.081901	3.383121	2.229725
C	0.993160	-1.809406	1.694855	C	1.447589	4.067338	2.213502
H	2.314981	-0.652056	2.944519	C	2.086891	4.009507	3.597088
H	-0.333433	-2.700926	0.245752	C	1.177443	4.634460	4.650406
H	1.061257	-2.704100	2.307099	C	-0.215030	4.014814	4.633489
C	0.292952	3.935070	-3.808258	C	-0.836251	4.076222	3.240614
O	0.440089	4.013909	-2.353801	C	-2.254525	2.294980	0.873031
C	1.508858	4.726188	-1.835188	C	-2.868636	1.851884	-0.460349
C	2.809635	4.552268	-2.303043	C	-4.191488	1.124268	-0.258780
C	3.830002	5.264195	-1.686794	C	-4.025183	-0.054717	0.691399
C	3.569400	6.133882	-0.630425	C	-3.448851	0.404512	2.024467
C	2.256141	6.306526	-0.183107	C	-2.110180	1.114969	1.843751

O	1.891924	7.121563	0.838924
C	2.896451	7.911600	1.513906
H	1.178659	3.423111	-4.207030
H	3.008089	3.853893	-3.108769
H	4.852843	5.139707	-2.029237
H	4.388094	6.675401	-0.172502
H	0.066979	7.897244	-1.014853
H	-2.294329	8.566339	-0.704319
H	-3.960066	6.856540	0.038590
H	-3.243584	4.587678	0.514564
H	0.229688	2.339476	2.549145
H	2.100624	3.618313	1.451339
H	1.311103	5.118321	1.928212
H	2.286051	2.959818	3.860620
H	3.059701	4.515646	3.581184
H	1.625044	4.545440	5.646246
H	1.084206	5.711949	4.449353
H	-0.152468	2.962078	4.946335
H	-0.866960	4.514770	5.358143
H	-0.971501	5.125047	2.937093
H	-1.835520	3.624649	3.250832
H	-2.940097	3.003158	1.358368
H	-2.996914	2.717360	-1.123875
H	-2.151235	1.185680	-0.964694
H	-4.931367	1.824550	0.156429
H	-4.585961	0.794713	-1.226084
H	-4.981700	-0.567006	0.841853
H	-3.343684	-0.790687	0.238642
H	-4.161003	1.088153	2.510159
H	-3.321462	-0.444432	2.705211
H	-1.371112	0.402538	1.464402
H	-1.744011	1.460311	2.817189
H	3.779034	7.277354	1.680641
C	0.162539	5.332549	-4.368309
H	1.052185	5.934079	-4.161698
H	0.030684	5.289653	-5.452221
H	-0.706517	5.837804	-3.934095
C	-0.932393	3.082325	-4.019422
H	-0.806173	2.106093	-3.539337
H	-1.81551	3.571807	-3.595066
H	-1.103552	2.923565	-5.086711
C	3.253692	9.12213	0.676026
H	4.019146	9.721083	1.176167
H	3.634863	8.838529	-0.308119
H	2.369076	9.749861	0.530715
C	2.28528	8.277884	2.845631
H	2.982534	8.881853	3.430831
H	1.368223	8.856352	2.699555
H	2.038679	7.381149	3.420548

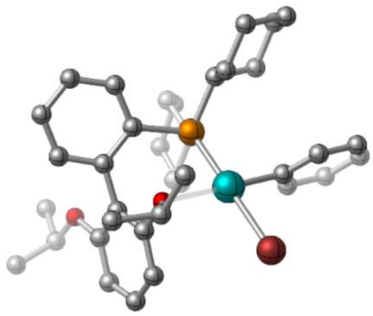

Zero-point correction = 0.765424 (Hartree/Particle)

Thermal correction to Energy = 0.799416

Thermal correction to Enthalpy = 0.800249

Thermal correction to Gibbs Free Energy = 0.702830

Sum of electronic and zero-point Energies = -2031.721611

Sum of electronic and thermal Energies = -2031.687619

Sum of electronic and thermal Enthalpies = -2031.686786

Sum of electronic and thermal Free Energies = -2031.784204

RM06L-D3/6-311g(d,p)-SDD(Pd,Br)-CPCM(THF)//RM06L-D3/6-31g(d,p)-LANL2DZ(Pd,Br)-CPCM(THF) = - 2034.230949

11a-conf1

E(scf) = -1875.22187836 a.u.

ν_{min} = 27.05 cm^{-1}

Br	3.633012	1.656690	-1.265212	H	1.929250	1.130120	2.480281
Pd	1.397388	2.591635	-0.204101	C	0.307882	-1.510686	0.153318
C	1.104151	0.742833	0.520413	H	0.277190	0.032846	-1.347106
C	1.465445	0.397146	1.822834	C	0.640528	-1.850255	1.464167
C	0.539985	-0.218872	-0.321868	H	1.506805	-1.154860	3.311841
C	1.229577	-0.897799	2.292388	H	-0.139057	-2.250154	-0.507066

H	0.449860	-2.853617	1.833956	O	2.413988	6.161581	0.742619
C	-0.166470	3.556472	-4.005993	C	3.544208	6.917403	1.241108
O	-0.033939	4.157754	-2.692818	H	0.703891	2.906407	-4.170460
C	1.194208	4.560763	-2.284324	H	2.336302	4.083483	-4.063973
C	2.352313	4.502134	-3.066212	H	4.442290	4.928762	-3.154671
C	3.545471	4.982334	-2.544512	H	4.584126	5.891593	-0.901796
C	3.633659	5.522624	-1.266606	H	0.311002	7.509528	-1.403100
C	2.478165	5.602897	-0.488108	H	-1.754020	8.589961	-0.573081
C	1.236030	5.110266	-0.972544	H	-3.289320	7.329782	0.933893
C	-0.021698	5.655335	-0.372322	H	-2.730448	5.035400	1.605464
C	-0.356392	6.967877	-0.736187	H	0.034761	2.262209	3.085642
C	-1.516416	7.573883	-0.272078	H	2.082962	3.520985	2.425525
C	-2.375076	6.870836	0.570159	H	1.258728	5.035527	2.739114
C	-2.054990	5.572914	0.945950	H	1.835556	2.848876	4.808535
C	-0.877426	4.956255	0.492064	H	2.662061	4.395174	4.700087
P	-0.431421	3.263172	1.005071	H	0.893201	4.431065	6.475398
C	-0.044099	3.326426	2.811665	H	0.553350	5.597850	5.204040
C	1.313505	3.985548	3.059339	H	-0.702119	2.817528	5.457700
C	1.695957	3.904681	4.532400	H	-1.512122	4.345164	5.778424
C	0.619776	4.520501	5.418451	H	-1.222762	5.027940	3.410751
C	-0.737674	3.876170	5.161714	H	-2.101123	3.503950	3.515233
C	-1.121726	3.969313	3.687482	H	-2.805842	3.036939	1.266471
C	-2.036289	2.344972	0.889424	H	-2.303213	2.996789	-1.159126
C	-2.335405	2.062637	-0.584088	H	-1.527161	1.430910	-0.980389
C	-3.669436	1.351489	-0.763391	H	-4.484315	2.022920	-0.455714
C	-3.722549	0.077371	0.071331	H	-3.833326	1.131025	-1.824827
C	-3.458308	0.379227	1.541977	H	-4.689261	-0.423520	-0.049918
C	-2.112325	1.072017	1.738341	H	-2.958075	-0.624686	-0.294107

H	-4.260081	1.025828	1.928441	H	-0.311207	4.202165	-6.057221
H	-3.487774	-0.540310	2.136978	H	-1.120709	5.266784	-4.89522
H	-1.307974	0.383777	1.454687	C	3.615504	8.253897	0.53181
H	-1.963506	1.307676	2.799324	H	4.473756	8.830153	0.886711
H	4.458592	6.335134	1.065509	H	3.713856	8.131063	-0.549983
C	-1.411608	2.703818	-3.955121	H	2.707812	8.832362	0.730283
H	-1.309397	1.903606	-3.218254	C	3.318408	7.051769	2.728794
H	-2.285600	3.308105	-3.691964	H	2.358294	7.538825	2.927803
H	-1.592242	2.250583	-4.932834	H	3.317767	6.074769	3.217879
C	-0.237353	4.642392	-5.059388	H	4.109312	7.658738	3.175584
H	0.644547	5.287311	-5.03743				

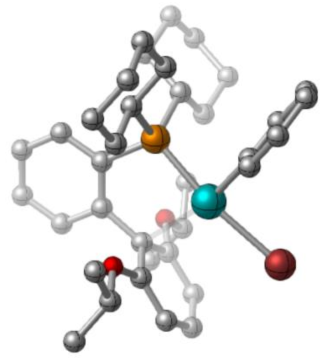

Zero-point correction= 0.652531 (Hartree/Particle)

Thermal correction to Energy= 0.682403

Thermal correction to Enthalpy= 0.683236

Thermal correction to Gibbs Free Energy= 0.594636

Sum of electronic and zero-point Energies= -1874.569348

Sum of electronic and thermal Energies= -1874.539475

Sum of electronic and thermal Enthalpies= -1874.538642

Sum of electronic and thermal Free Energies= -1874.627242

RM06L-D3/6-311g(d,p)-SDD(Pd,Br)-CPCM(THF)//RM06L-D3/6-31g(d,p)-LANL2DZ(Pd,Br)-CPCM(THF) = -1876.935435

11a-int1

E(scf) = -2709.92273615.

ν_{min} = 19.53 cm^{-1}

Br	-3.946342	-1.049328	-3.169805	H	4.513809	1.284606	0.261408
Mg	-1.587634	-1.711230	-2.402059	H	1.747545	-1.412962	2.158514
H	-0.869829	-4.986576	-5.989634	Br	0.269152	-0.133009	-3.539529
H	-0.927947	-5.252585	-3.259867	C	-1.338299	-2.581216	-0.473999
H	-0.264868	-2.702271	-5.505184	C	-2.260743	-2.452757	0.584211
C	-1.723910	-4.335743	-5.775397	C	-0.164250	-3.293712	-0.150203
C	-1.759284	-4.557976	-3.431880	C	-2.030738	-2.956735	1.867020
H	-2.722935	-6.002172	-4.742653	H	-3.202685	-1.925240	0.408661
O	-1.199476	-3.251222	-3.739433	C	0.087027	-3.817395	1.119402
C	-1.256974	-3.023036	-5.178380	H	0.603670	-3.436564	-0.916979
C	-2.570279	-4.925068	-4.652597	C	-0.846662	-3.640346	2.140317
H	-2.276836	-4.188140	-6.704884	H	-2.772986	-2.820559	2.653039
H	-2.331404	-4.461126	-2.504652	H	-0.655640	-4.035079	3.135779
H	-1.972706	-2.209473	-5.356155	H	4.052938	-0.500034	1.935114
H	-3.551258	-4.439137	-4.614661	H	1.013732	-4.353680	1.320157
Pd	-0.141787	1.643849	-1.544655	C	0.488049	3.829466	-3.169337
C	1.186971	0.868649	-0.280070	C	1.446482	3.360894	-4.069598
C	2.479264	1.379367	-0.426769	C	2.770028	3.742768	-3.893755
C	0.916179	-0.129681	0.652117	C	3.153167	4.559456	-2.835658
C	3.510018	0.880907	0.372828	C	2.183504	5.031664	-1.942240
H	2.684649	2.171760	-1.147242	C	0.825588	4.688192	-2.110655
C	1.960078	-0.623862	1.441217	C	-0.237150	5.370465	-1.322135
H	-0.082987	-0.542862	0.761824	C	-0.389300	6.744137	-1.559864
C	3.250838	-0.118057	1.310100	C	-1.388985	7.489096	-0.948290

C	-2.274799	6.865577	-0.075194	H	1.886640	4.427670	4.942955
C	-2.133400	5.508511	0.187427	H	1.044566	5.527424	3.859764
C	-1.119250	4.745220	-0.414303	H	0.256946	2.630256	4.443094
P	-0.909222	3.008107	0.122558	H	-0.593898	4.061217	5.00675
C	0.017636	3.140961	1.724444	H	-1.159132	4.68555	2.672842
C	1.282872	3.986743	1.555791	H	-1.717383	3.055288	3.045884
C	2.109549	3.967409	2.836717	H	-2.992963	3.102248	1.339771
C	1.294499	4.468582	4.022301	H	-3.585675	3.38101	-1.091103
C	0.008653	3.668746	4.180378	H	-3.149902	1.693513	-1.365073
C	-0.818397	3.663569	2.897017	H	-5.366582	2.687244	0.495537
C	-2.592663	2.410562	0.583420	H	-5.595921	1.901049	-1.062252
C	-3.545364	2.394221	-0.612743	H	-5.89176	0.292024	0.846963
C	-4.937019	1.938989	-0.187002	H	-4.582122	-0.177107	-0.231964
C	-4.891860	0.580079	0.504031	H	-4.256121	1.253156	2.457579
C	-3.905704	0.573526	1.666318	H	-3.846999	-0.423549	2.117542
C	-2.520120	1.015314	1.210024	H	-2.139416	0.311438	0.451819
H	1.169304	2.692328	-4.875424	H	-1.8081	0.984061	2.045805
H	3.524367	3.385315	-4.588214	O	-0.835687	3.441555	-3.213506
H	4.194061	4.835584	-2.722008	O	2.446708	5.849035	-0.892514
H	0.295974	7.224205	-2.253551	C	-1.422274	3.12868	-4.514283
H	-1.481016	8.549890	-1.161556	H	-0.802815	2.358852	-4.992618
H	-3.070447	7.428173	0.403366	C	3.818401	6.167039	-0.566555
H	-2.826985	5.038294	0.878625	H	4.31922	6.507863	-1.484668
H	0.324113	2.107337	1.945631	C	-1.469797	4.385514	-5.353357
H	1.872019	3.631980	0.701698	H	-0.468788	4.788722	-5.530204
H	0.996889	5.021876	1.318343	H	-1.923575	4.17324	-6.324642
H	2.453308	2.939706	3.027474	H	-2.069044	5.153307	-4.853571
H	3.012420	4.577535	2.715516	C	-2.775356	2.545774	-4.205461

H	-2.6786	1.654309	-3.577311	H	3.195636	6.992506	1.319456
H	-3.40672	3.274359	-3.686613	C	4.538714	4.963399	0.010423
H	-3.277846	2.250188	-5.129865	H	4.530962	4.107096	-0.668158
C	3.728295	7.31074	0.416979	H	5.581117	5.216446	0.221145
H	4.727597	7.64134	0.709171	H	4.068856	4.652919	0.948273
H	3.193875	8.159497	-0.015489				

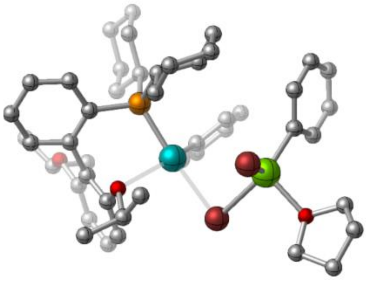

Zero-point correction= 0.975981 (Hartree/Particle)

Thermal correction to Energy= 1.022971

Thermal correction to Enthalpy= 1.023803

Thermal correction to Gibbs Free Energy= 0.896601

Sum of electronic and zero-point Energies= -2708.946755

Sum of electronic and thermal Energies= -2708.899766

Sum of electronic and thermal Enthalpies= -2708.898933

Sum of electronic and thermal Free Energies= -2709.026135

RM06L-D3/6-311g(d,p)-SDD(Pd,Br)-CPCM(THF)//RM06L-D3/6-31g(d,p)-LANL2DZ(Pd,Br)-CPCM(THF) = -2711.993789

TS1-11a

E(scf) = -2709.91901567 a.u.

ν_{min} = -61.69 cm^{-1}

Br	-3.244172	0.133566	-2.646903	C	-0.511185	-2.212240	-1.618146
Mg	-1.196491	-1.352865	-3.472426	C	-1.381810	-2.369591	-0.520135
H	1.590719	2.313586	-4.211016	C	0.740816	-2.850881	-1.475817
H	1.899246	-0.527676	-2.732021	C	-1.039208	-3.082586	0.633305
H	-0.561613	1.472243	-4.987089	H	-2.375387	-1.914531	-0.564454
C	1.647908	1.558554	-5.003318	C	1.103357	-3.573255	-0.339827
C	1.712566	-0.535925	-3.810432	H	1.472947	-2.794913	-2.283604
H	3.443266	0.773808	-4.016080	C	0.214080	-3.681440	0.730763
O	0.345493	-0.069073	-3.994160	H	-1.749573	-3.167487	1.455439
C	0.323055	0.842977	-5.113765	H	0.492523	-4.234876	1.624465
C	2.593324	0.424598	-4.606976	H	4.713308	0.613189	-0.572875
H	1.938484	2.056455	-5.930771	H	2.080443	-4.050528	-0.282950
H	1.780063	-1.567457	-4.178058	C	-5.004880	3.266940	-2.706110
H	0.233887	0.267952	-6.046288	O	-3.828255	3.512463	-1.901403
H	2.988347	-0.071594	-5.497888	C	-2.645359	3.762272	-2.515169
Pd	-1.075135	0.812315	-0.982462	C	-2.400218	3.579172	-3.881941
C	0.858418	0.907972	-0.593118	C	-1.150759	3.910697	-4.390696
C	1.618907	1.816970	-1.327240	C	-0.131295	4.401545	-3.579917
C	1.462957	-0.087609	0.171068	C	-0.381207	4.579560	-2.213845
C	3.012197	1.703559	-1.319889	C	-1.636263	4.261861	-1.671055
H	1.135346	2.600472	-1.905942	C	-2.011979	4.686592	-0.297802
C	2.855974	-0.195689	0.160631	C	-2.350533	6.040544	-0.155565
H	0.861458	-0.802289	0.732110	C	-2.854600	6.551518	1.032178
C	3.630580	0.698205	-0.578071	C	-3.054734	5.696521	2.111638
H	3.607486	2.408326	-1.897948	C	-2.703191	4.357876	1.998169
H	3.329419	-0.991993	0.730188	C	-2.153964	3.835217	0.812660
Br	-1.310132	-2.711736	-5.645306	P	-1.504373	2.118050	0.901310

C	-0.085210	2.287967	2.097571	H	1.441974	4.447082	4.200071
C	0.864475	3.410195	1.668254	H	1.293225	1.393772	4.298256
C	2.149247	3.359710	2.486812	H	0.604235	2.50359	5.475115
C	1.854036	3.451684	3.979250	H	-0.912464	3.432989	3.739553
C	0.847341	2.392673	4.412656	H	-1.137983	1.694019	3.926073
C	-0.432824	2.457964	3.580635	H	-2.971278	1.75183	2.778131
C	-2.798932	1.193823	1.845817	H	-4.469675	2.14701	0.851198
C	-4.117036	1.132263	1.073877	H	-3.937875	0.649001	0.101767
C	-5.169794	0.344094	1.844053	H	-5.422481	0.881574	2.769853
C	-4.667141	-1.051807	2.195169	H	-6.093453	0.288688	1.256428
C	-3.362870	-0.979479	2.980643	H	-5.426143	-1.60393	2.759906
C	-2.300120	-0.203704	2.211193	H	-4.496003	-1.618303	1.266935
O	0.530297	5.046183	-1.316761	H	-3.544794	-0.484512	3.946257
C	1.805436	5.522572	-1.798967	H	-2.993251	-1.984351	3.215484
H	-4.749628	2.530365	-3.478967	H	-2.050777	-0.733064	1.28326
H	-3.170356	3.198346	-4.541714	H	-1.367247	-0.151643	2.788146
H	-0.963085	3.790400	-5.454696	H	2.186174	4.806383	-2.543686
H	0.822991	4.665785	-4.019040	C	-6.017028	2.655872	-1.766324
H	-2.227758	6.691065	-1.018585	H	-5.646376	1.709516	-1.363613
H	-3.109591	7.604456	1.107216	H	-6.22621	3.333167	-0.93169
H	-3.477437	6.064890	3.041401	H	-6.954331	2.464969	-2.294934
H	-2.859913	3.712243	2.855796	C	-5.477932	4.564801	-3.328287
H	0.448363	1.332519	1.993249	H	-4.710199	5.007162	-3.969039
H	1.079051	3.356249	0.597004	H	-6.369467	4.394724	-3.937801
H	0.361279	4.376653	1.826205	H	-5.729574	5.288631	-2.546556
H	2.672335	2.416201	2.269013	C	2.724946	5.518889	-0.600528
H	2.824424	4.166562	2.178328	H	2.85968	4.50494	-0.213642
H	2.7772	3.36316	4.562555	H	3.705156	5.915845	-0.875703

H	2.313511	6.141817	0.200112	H	0.928043	6.881417	-3.246056
C	1.642299	6.895316	-2.41876	H	1.279958	7.603507	-1.667165
H	2.598496	7.261204	-2.801712				

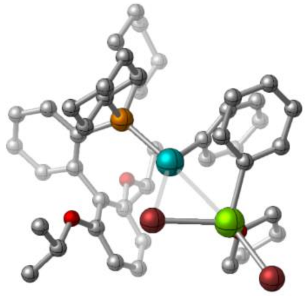

Zero-point correction= 0.976237 (Hartree/Particle)

Thermal correction to Energy= 1.022486

Thermal correction to Enthalpy= 1.023319

Thermal correction to Gibbs Free Energy= 0.898229

Sum of electronic and zero-point Energies= -2708.942778

Sum of electronic and thermal Energies= -2708.896530

Sum of electronic and thermal Enthalpies= -2708.895697

Sum of electronic and thermal Free Energies= -2709.020787

RM06L-D3/6-311g(d,p)-SDD(Pd,Br)-CPCM(THF)//RM06L-D3/6-31g(d,p)-LANL2DZ(Pd,Br)-CPCM(THF) = -2711.987809

3a

E(scf) = -1875.22187836 a.u.

ν_{min} = 27.05 cm^{-1}

Br	3.809851	2.032309	-1.013692	P	-0.485158	3.342663	0.987073
Pd	1.429378	2.792159	-0.150336	C	-0.127658	3.390025	2.801221
C	1.223101	0.917358	0.536489	C	1.178712	4.142023	3.073779
C	1.562045	0.557380	1.840656	C	1.560895	4.057185	4.546916
C	0.737894	-0.049412	-0.347911	C	0.433250	4.546357	5.448721
C	1.377994	-0.759564	2.270322	C	-0.866850	3.804126	5.160871
H	1.970553	1.294855	2.528896	C	-1.252365	3.925704	3.689082
C	0.559006	-1.363688	0.087282	C	-2.032958	2.346529	0.795089
H	0.492491	0.215456	-1.374230	C	-2.271990	2.086307	-0.696103
C	0.866135	-1.719239	1.400028	C	-3.551408	1.293527	-0.928824
H	1.636569	-1.029022	3.291460	C	-3.549304	-0.000692	-0.124193
H	0.172071	-2.107487	-0.605341	C	-3.353530	0.283704	1.360314
H	0.716317	-2.740278	1.738928	C	-2.059466	1.051792	1.613956
C	-0.388121	3.400782	-3.680026	O	2.371582	6.360178	0.671857
O	-0.275986	4.276158	-2.565968	C	3.646248	6.631471	1.236624
C	0.975323	4.680085	-2.228317	H	-0.135076	3.905378	-4.618662
C	2.087919	4.565050	-3.065110	H	0.257883	2.523029	-3.551677
C	3.308314	5.070633	-2.634276	H	-1.430663	3.084387	-3.710702
C	3.462426	5.661229	-1.384702	H	2.005940	4.088589	-4.034374
C	2.351325	5.774117	-0.549839	H	4.173117	4.982583	-3.284830
C	1.081742	5.289287	-0.950954	H	4.433959	6.021303	-1.068750
C	-0.152496	5.802608	-0.285442	H	0.147458	7.725094	-1.193909
C	-0.509088	7.129188	-0.564750	H	-1.940014	8.708584	-0.302178
C	-1.683939	7.679421	-0.068599	H	-3.464938	7.317328	1.094224
C	-2.536583	6.903118	0.712978	H	-2.860298	4.996061	1.624617
C	-2.191287	5.590418	1.008558	H	0.029745	2.328576	3.050820
C	-0.995223	5.032694	0.530283	H	1.982659	3.743417	2.437851

H	1.053797	5.193497	2.775565	H	-4.415882	1.904493	-0.630607
H	1.794274	3.011047	4.794746	H	-3.671715	1.088513	-1.998714
H	2.478005	4.629298	4.727922	H	-4.477537	-0.558557	-0.289397
H	0.711514	4.437937	6.502680	H	-2.729764	-0.644477	-0.477493
H	0.275854	5.621264	5.278082	H	-4.205748	0.871887	1.731829
H	-0.747574	2.740726	5.414980	H	-3.345718	-0.648656	1.935693
H	-1.677332	4.185336	5.791600	H	-1.207187	0.419171	1.341674
H	-1.424161	4.984625	3.452233	H	-1.956113	1.272309	2.683573
H	-2.196624	3.401325	3.491990	H	3.456987	6.972135	2.254292
H	-2.851256	2.986071	1.162860	H	4.264421	5.725899	1.261928
H	-2.294557	3.038591	-1.240103	H	4.175317	7.415184	0.683258
H	-1.412152	1.526467	-1.093909				

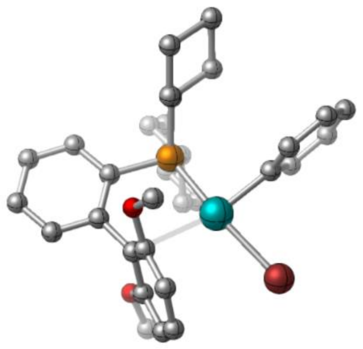

Zero-point correction= 0.652531 (Hartree/Particle)

Thermal correction to Energy= 0.682403

Thermal correction to Enthalpy= 0.683236

Thermal correction to Gibbs Free Energy= 0.594636

Sum of electronic and zero-point Energies= -1874.569348

Sum of electronic and thermal Energies= -1874.539475

Sum of electronic and thermal Enthalpies= -1874.538642

Sum of electronic and thermal Free Energies= -1874.627242

RM06L-D3/6-311g(d,p)-SDD(Pd,Br)-CPCM(THF)//RM06L-D3/6-31g(d,p)-LANL2DZ(Pd,Br)-CPCM(THF) = -1876.935435

3a-conf1

E(scf) = -1875.21899241 a.u.

ν_{min} = 11.99 cm^{-1}

Br	2.202999	0.935953	-2.852809	C	-0.798564	7.152436	-0.738372
Pd	0.645557	2.136592	-1.083846	C	-2.138122	7.482929	-0.590062
C	0.729537	0.504368	0.051716	C	-3.033757	6.506417	-0.171691
C	1.508746	0.444203	1.207460	C	-2.572425	5.228103	0.119441
C	0.008366	-0.621759	-0.356363	C	-1.216777	4.877859	-0.001000
C	1.534769	-0.724740	1.973259	P	-0.664065	3.189940	0.510966
H	2.094315	1.303718	1.524812	C	0.142948	3.396012	2.162630
C	0.038446	-1.785348	0.413608	C	1.553509	3.978802	2.107359
H	-0.584038	-0.595812	-1.267870	C	2.211310	3.888308	3.479838
C	0.792465	-1.837167	1.585417	C	1.379889	4.630070	4.521454
H	2.137694	-0.755535	2.877747	C	-0.058124	4.123503	4.553231
H	-0.534993	-2.651850	0.092747	C	-0.705234	4.175810	3.171322
H	0.807190	-2.741751	2.186508	C	-2.233864	2.280046	0.917615
O	0.506240	4.085493	-2.431325	C	-2.888585	1.793896	-0.381208
C	1.522525	4.809551	-1.822955	C	-4.204221	1.074095	-0.114830
C	2.854367	4.679891	-2.212191	C	-4.005163	-0.074961	0.864906
C	3.817324	5.406614	-1.524880	C	-3.389752	0.426460	2.164911
C	3.469055	6.254232	-0.475750	C	-2.059277	1.135500	1.925358
C	2.126958	6.383297	-0.115376	O	1.688601	7.187939	0.891108
C	1.123163	5.642692	-0.773821	H	3.124670	4.000637	-3.012885
C	-0.315369	5.863519	-0.473570	H	4.861709	5.309746	-1.805187

H	4.238243	6.810643	0.046031
H	-0.091473	7.901054	-1.084540
H	-2.478639	8.489467	-0.813547
H	-4.090486	6.731302	-0.064469
H	-3.302021	4.497681	0.448236
H	0.231148	2.354478	2.508982
H	2.154879	3.482852	1.332281
H	1.483990	5.033761	1.823699
H	2.309195	2.830908	3.769131
H	3.228130	4.295006	3.437356
H	1.836352	4.544210	5.513546
H	1.375607	5.702197	4.273348
H	-0.069729	3.082730	4.909169
H	-0.654634	4.701056	5.267914
H	-0.783150	5.220156	2.833514
H	-1.730343	3.787292	3.216813
H	-2.906397	3.001198	1.402346
H	-3.036723	2.636256	-1.069820

H	-2.188356	1.110165	-0.883517
H	-4.930822	1.787041	0.302535
H	-4.629570	0.715032	-1.058247
H	-4.954993	-0.584303	1.060927
H	-3.336057	-0.822706	0.413256
H	-4.089799	1.122686	2.650288
H	-3.238841	-0.400582	2.867494
H	-1.324026	0.411870	1.559921
H	-1.678113	1.520407	2.877993
C	0.583887	3.965563	-3.860316
H	1.408520	3.315597	-4.163040
H	-0.358854	3.514469	-4.168560
H	0.688968	4.960033	-4.302929
C	2.675454	7.840220	1.675399
H	3.263960	8.546275	1.079006
H	2.133476	8.383356	2.448428
H	3.351325	7.114354	2.144484

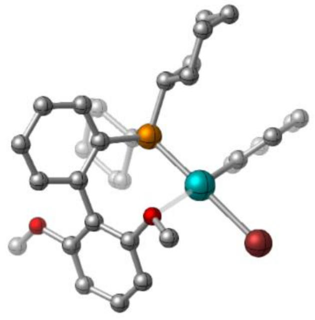

Zero-point correction= 0.653431 (Hartree/Particle)

Thermal correction to Energy= 0.683063

Thermal correction to Enthalpy= 0.683896

Thermal correction to Gibbs Free Energy= 0.595397

Sum of electronic and zero-point Energies= -1874.565562

Sum of electronic and thermal Energies= -1874.535929

Sum of electronic and thermal Enthalpies= -1874.535097

Sum of electronic and thermal Free Energies= -1874.623596

Sum of electronic and thermal Free Energies= -4024.000692

RM06L-D3/6-311g(d,p)-SDD(Pd,Br)-CPCM(THF)//RM06L-D3/6-31g(d,p)-LANL2DZ(Pd,Br)-CPCM(THF) = -1876.932072

3a-int1

E(scf) = -2552.65680293 a.u.

ν_{min} = 12.98 cm^{-1}

Br	-3.402361	-0.389044	-3.338629	H	-1.571123	-1.958231	-5.387348
Mg	-1.365403	-1.612819	-2.389358	H	-3.610883	-3.862282	-4.871775
H	-0.966335	-4.883419	-6.029785	Pd	-0.084761	1.517633	-1.438556
H	-1.326472	-5.188979	-3.321689	C	1.274717	0.881104	-0.136515
H	0.015180	-2.772472	-5.406577	C	2.559518	1.403603	-0.279217
C	-1.697629	-4.085470	-5.864679	C	0.993731	-0.094320	0.816253
C	-1.991161	-4.344959	-3.542071	C	3.577911	0.945968	0.560035
H	-3.083348	-5.551142	-4.985868	H	2.770549	2.164974	-1.029234
O	-1.167266	-3.164095	-3.751300	C	2.026485	-0.547653	1.643372
C	-1.046153	-2.900154	-5.180415	H	-0.002660	-0.519285	0.911011
C	-2.739448	-4.525184	-4.842323	C	3.312206	-0.025832	1.523629
H	-2.125370	-3.815070	-6.831779	H	4.578613	1.358467	0.454820
H	-2.622477	-4.159625	-2.667693	H	1.810450	-1.317238	2.380533

Br	0.802527	-0.324674	-3.247401	C	2.160048	4.045547	2.724449
C	-1.251643	-2.536573	-0.475723	C	1.386060	4.561379	3.931757
C	-2.228595	-2.463304	0.537452	C	0.110870	3.756293	4.154237
C	-0.086543	-3.250121	-0.121088	C	-0.768304	3.718114	2.905138
C	-2.059086	-3.020425	1.807591	C	-2.597259	2.382759	0.625475
H	-3.166495	-1.938099	0.336677	C	-3.563784	2.400711	-0.560472
C	0.105746	-3.824528	1.137009	C	-4.942565	1.900862	-0.142904
H	0.723004	-3.351135	-0.850711	C	-4.866289	0.518948	0.496890
C	-0.881775	-3.701832	2.113859	C	-3.886921	0.499711	1.664535
H	-2.843208	-2.926742	2.558349	C	-2.508491	0.976848	1.224031
H	-0.737855	-4.136790	3.100419	H	1.033823	2.539279	-4.864319
H	4.106233	-0.376792	2.176446	H	3.406682	3.132066	-4.522567
H	1.027906	-4.358529	1.362842	H	4.085476	4.632183	-2.686890
C	0.352749	3.746130	-3.207250	H	0.116065	7.168631	-2.371191
C	1.314744	3.224009	-4.073725	H	-1.630289	8.490429	-1.222358
C	2.649825	3.550839	-3.865944	H	-3.127156	7.375936	0.435240
C	3.038242	4.394437	-2.830349	H	-2.829495	4.999253	0.940675
C	2.057925	4.950084	-2.001229	H	0.357861	2.153916	1.961782
C	0.698541	4.631738	-2.172637	H	1.834497	3.668103	0.611799
C	-0.350023	5.326906	-1.381550	H	0.968686	5.058319	1.239990
C	-0.531605	6.692672	-1.639762	H	2.517550	3.023853	2.921981
C	-1.513065	7.434847	-0.995975	H	3.051802	4.657245	2.544707
C	-2.347395	6.815168	-0.070895	H	2.011169	4.539401	4.831185
C	-2.175448	5.465103	0.209278	H	1.121759	5.615439	3.762823
C	-1.179275	4.704869	-0.425803	H	0.377028	2.724737	4.427063
P	-0.922857	2.980471	0.139130	H	-0.460316	4.161178	4.996815
C	0.030706	3.175474	1.718237	H	-1.126545	4.732219	2.673568
C	1.278240	4.031659	1.482690	H	-1.6554	3.103893	3.099508

H	-2.980314	3.059281	1.404341	O	-0.978065	3.409676	-3.26538
H	-3.630259	3.40859	-0.988693	O	2.330952	5.8217	-0.99304
H	-3.164759	1.746696	-1.350806	C	3.690407	5.974488	-0.621396
H	-5.382433	2.611912	0.572284	H	4.279428	6.450706	-1.413674
H	-5.608118	1.883964	-1.0134	H	3.690869	6.614464	0.260695
H	-5.859871	0.190909	0.82185	H	4.143518	5.006399	-0.36973
H	-4.529603	-0.202073	-0.263418	C	-1.437064	2.762988	-4.454791
H	-4.255266	1.153802	2.468941	H	-2.516342	2.670434	-4.341517
H	-3.811765	-0.506522	2.092275	H	-1.198199	3.365684	-5.337226
H	-2.11061	0.292508	0.455949	H	-1.004894	1.760729	-4.558148
H	-1.799052	0.947005	2.062779				

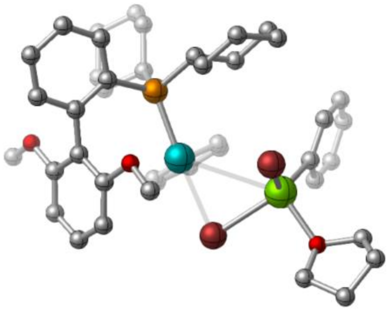

Zero-point correction= 0.862738 (Hartree/Particle)

Thermal correction to Energy= 0.905817

Thermal correction to Enthalpy= 0.906650

Thermal correction to Gibbs Free Energy= 0.785647

Sum of electronic and zero-point Energies= -2551.794065

Sum of electronic and thermal Energies= -2551.750986

Sum of electronic and thermal Enthalpies= -2551.750153

Sum of electronic and thermal Free Energies= -2551.871156

RM06L-D3/6-311g(d,p)-SDD(Pd,Br)-CPCM(THF)//RM06L-D3/6-31g(d,p)-LANL2DZ(Pd,Br)-CPCM(THF) = -2554.69711

TS1-3a

E(scf) = -3471.73675298 a.u.

ν_{min} = -33.56 cm^{-1}

Br	-3.299739	0.295591	-2.640232	H	3.664821	2.154985	-1.978367
Mg	-1.393337	-1.353979	-3.424162	H	3.238728	-1.171610	0.723912
H	1.439416	2.237816	-4.270135	Br	-1.992874	-2.547141	-5.588250
H	1.750070	-0.626498	-2.832702	C	-0.685357	-2.236247	-1.590475
H	-0.759102	1.445632	-4.964333	C	-1.528270	-2.365529	-0.467579
C	1.450419	1.488874	-5.070203	C	0.541475	-2.929169	-1.495232
C	1.522953	-0.619938	-3.903207	C	-1.187479	-3.121370	0.658746
H	3.266846	0.662050	-4.159793	H	-2.494085	-1.848759	-0.465522
O	0.158396	-0.126894	-4.029096	C	0.905465	-3.684806	-0.381320
C	0.107479	0.801244	-5.135541	H	1.252323	-2.888039	-2.323096
C	2.389619	0.333864	-4.722138	C	0.039134	-3.778723	0.708862
H	1.714571	1.989570	-6.003958	H	-1.875608	-3.190694	1.501192
H	1.556843	-1.649030	-4.281914	H	0.314760	-4.363734	1.583077
H	-0.032233	0.241909	-6.071173	H	4.689263	0.329877	-0.626464
H	2.742807	-0.158842	-5.632203	H	1.863507	-4.201816	-0.358569
Pd	-1.076108	0.864419	-0.993862	C	-4.946741	3.468720	-3.331919
C	0.856997	0.830481	-0.608527	O	-3.992087	3.719771	-2.310886
C	1.655898	1.680468	-1.372099	C	-2.701888	3.888765	-2.696324
C	1.415317	-0.181326	0.168869	C	-2.254146	3.739957	-4.014121
C	3.040751	1.494529	-1.379326	C	-0.920447	4.000674	-4.305114
H	1.203199	2.475999	-1.961856	C	-0.022694	4.405426	-3.321427
C	2.800753	-0.362496	0.144187	C	-0.483757	4.547455	-2.008951
H	0.783239	-0.855147	0.746727	C	-1.816081	4.263430	-1.671522
C	3.612735	0.473119	-0.622125	C	-2.351704	4.592427	-0.324119

C	-3.016370	5.824313	-0.226419	H	-3.946553	5.779961	3.031913
C	-3.583862	6.265957	0.959688	H	-2.819308	3.626656	2.912315
C	-3.504654	5.462587	2.092412	H	0.526644	1.413982	2.034264
C	-2.857313	4.237121	2.016942	H	1.275097	3.260415	0.49802
C	-2.264459	3.779127	0.824092	H	0.583191	4.429177	1.604979
P	-1.414125	2.147257	0.916061	H	2.791172	2.408022	2.258151
C	0.036080	2.398918	2.060218	H	3.032206	4.132085	2.012454
C	1.041772	3.431269	1.550160	H	2.936611	3.541605	4.459794
C	2.315531	3.391936	2.387548	H	1.656198	4.654104	3.998142
C	2.021223	3.625589	3.863778	H	1.359656	1.629325	4.345895
C	0.962600	2.654860	4.372258	H	0.715712	2.864654	5.418737
C	-0.305486	2.714112	3.522433	H	-0.73108	3.724509	3.585719
C	-2.570650	1.107463	1.930616	H	-1.055209	2.026218	3.928748
C	-3.858670	0.783053	1.172057	H	-2.840176	1.697767	2.817826
C	-4.800372	-0.050356	2.032894	H	-4.347603	1.705622	0.832371
C	-4.119256	-1.332521	2.496130	H	-3.601526	0.216464	0.26482
C	-2.825600	-1.027054	3.241319	H	-5.111149	0.53678	2.909525
C	-1.880251	-0.173370	2.402243	H	-5.7133	-0.277745	1.472012
O	0.285336	4.998698	-0.981797	H	-4.793455	-1.924284	3.124765
C	1.625751	5.355894	-1.272345	H	-3.89136	-1.952361	1.615316
H	-4.957831	4.278986	-4.070457	H	-3.060638	-0.492106	4.173454
H	-4.749843	2.515461	-3.833867	H	-2.319311	-1.953202	3.538114
H	-5.913068	3.419460	-2.831408	H	-1.555224	-0.737811	1.518043
H	-2.932546	3.427590	-4.799078	H	-0.973805	0.052541	2.976957
H	-0.569620	3.894708	-5.328541	H	2.05181	5.712452	-0.334471
H	1.006342	4.629222	-3.579282	H	2.208283	4.494577	-1.625211
H	-3.084063	6.438189	-1.120884	H	1.675091	6.152492	-2.0233
H	-4.089120	7.226439	0.997039				

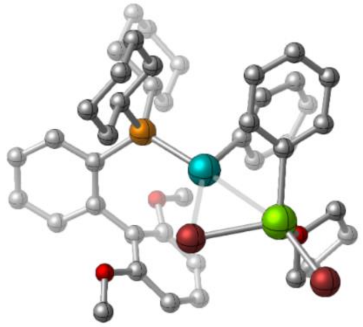

Zero-point correction= 0.864223 (Hartree/Particle)

Thermal correction to Energy= 0.905928

Thermal correction to Enthalpy= 0.906760

Thermal correction to Gibbs Free Energy= 0.791234

Sum of electronic and zero-point Energies= -2551.789488

Sum of electronic and thermal Energies= -2551.747783

Sum of electronic and thermal Enthalpies= -2551.746951

Sum of electronic and thermal Free Energies= -2551.862477.

RM06L-D3/6-311g(d,p)-SDD(Pd,Br)-CPCM(THF)//RM06L-D3/6-31g(d,p)-LANL2DZ(Pd,Br)-CPCM(THF) = -2554.691994

14b

E(scf) = -1914.09299650 a.u.

ν_{min} = 25.30 cm^{-1}

Br	2.519231	0.774923	-1.883268	C	2.358672	0.066661	3.138689
Pd	1.021275	2.224809	-0.285591	H	0.648467	-0.002258	1.825407
C	2.013204	1.592898	1.308643	C	3.551354	0.711462	3.456794
C	3.217929	2.231492	1.600784	H	4.912645	2.298191	2.918967
C	1.581559	0.503858	2.061748	H	2.019811	-0.781900	3.727650
C	3.979709	1.790539	2.685342	H	4.146986	0.372735	4.299371
H	3.560584	3.068754	0.995493	C	1.198207	4.965912	-1.719386

C	2.457558	4.665047	-2.260286	H	-2.132059	8.727844	0.480580
C	3.622566	4.928554	-1.551630	H	-3.717484	7.038006	1.418797
C	3.564003	5.521516	-0.299247	H	-3.048778	4.688702	1.545444
C	2.325757	5.856557	0.260976	H	-0.203264	1.994928	2.943642
C	1.131785	5.579292	-0.436947	H	1.480321	3.815097	2.773571
C	-0.184467	5.930897	0.169596	H	0.267243	4.994195	3.235212
C	-0.602747	7.268994	0.098790	H	1.216336	2.618366	4.922753
C	-1.846523	7.682895	0.556138	H	1.610794	4.306357	5.219826
C	-2.730273	6.743133	1.076600	H	-0.259301	3.521143	6.705816
C	-2.342842	5.411179	1.147318	H	-0.775592	4.825907	5.646737
C	-1.073344	4.984267	0.718763	H	-1.281012	1.844178	5.186350
P	-0.652238	3.216563	0.975432	H	-2.485397	3.031503	5.668166
C	-0.535612	3.035662	2.814644	H	-2.174159	4.251868	3.529338
C	0.566796	3.943039	3.362386	H	-2.626858	2.571806	3.234251
C	0.827456	3.643740	4.833488	H	-3.030560	2.721141	1.070788
C	-0.448291	3.775974	5.657160	H	-2.590036	3.570642	-1.228984
C	-1.562992	2.903034	5.091138	H	-1.638695	2.136759	-1.585546
C	-1.824693	3.214245	3.618537	H	-4.617335	2.168335	-0.879587
C	-2.191102	2.308650	0.490831	H	-3.938210	1.875398	-2.474465
C	-2.495430	2.502543	-0.997144	H	-4.551328	-0.272457	-1.333368
C	-3.750492	1.739734	-1.403453	H	-2.825914	-0.186183	-1.658452
C	-3.633611	0.259239	-1.059571	H	-4.172021	0.418592	1.021478
C	-3.324040	0.060395	0.419547	H	-3.208113	-1.003810	0.651449
C	-2.067504	0.820175	0.830940	H	-1.194155	0.412616	0.295889
H	2.528577	4.200095	-3.236234	H	-1.870673	0.680858	1.902331
H	4.583653	4.674959	-1.990799	N	2.251467	6.433601	1.573665
H	4.476774	5.728122	0.251717	N	0.015075	4.546407	-2.387897
H	0.052804	7.987944	-0.384824	C	2.309699	7.890868	1.566651

H	2.124385	8.263097	2.579396	H	-0.574717	6.172647	-3.627557
H	3.296164	8.269682	1.241795	H	-1.871476	5.081606	-3.099758
H	1.551266	8.311109	0.907373	H	-1.189125	6.24625	-1.963005
C	3.253432	5.933002	2.504118	C	0.197675	3.634916	-3.510169
H	4.265877	6.336942	2.325068	H	-0.780807	3.223604	-3.775281
H	2.962994	6.222434	3.519714	H	0.604268	4.133906	-4.406258
H	3.308347	4.842361	2.460629	H	0.860284	2.805867	-3.246296
C	-0.947359	5.573144	-2.780792				

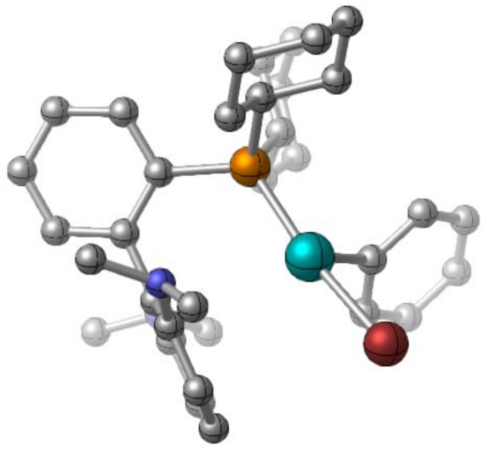

Zero-point correction= 0.733425 (Hartree/Particle)

Thermal correction to Energy= 0.765563

Thermal correction to Enthalpy= 0.766396

Thermal correction to Gibbs Free Energy= 0.673004

Sum of electronic and zero-point Energies= -1913.359571

Sum of electronic and thermal Energies= -1913.327434

Sum of electronic and thermal Enthalpies= -1913.326601

Sum of electronic and thermal Free Energies= -1913.419992

RM06L-D3/6-311g(d,p)-SDD(Pd,Br)-CPCM(THF)//RM06L-D3/6-31g(d,p)-LANL2DZ(Pd,Br)-CPCM(THF)= -1915.804622

14b-conf1

E(scf) = -1914.08738357a.u.

ν_{min} = 20.30 cm^{-1}

Br	2.480984	0.841854	-2.462095	C	-1.293307	4.921839	0.157061
Pd	0.648610	2.201283	-1.083522	P	-0.650773	3.234389	0.586114
C	0.598568	0.536782	0.037198	C	0.279100	3.353624	2.177101
C	1.366997	0.350025	1.186474	C	1.725377	3.811484	2.028122
C	-0.215483	-0.504334	-0.420025	C	2.462038	3.666377	3.355839
C	1.273354	-0.843367	1.907574	C	1.751433	4.420415	4.476899
H	2.040900	1.129854	1.533196	C	0.283434	4.023559	4.586014
C	-0.301888	-1.696695	0.299976	C	-0.435858	4.178219	3.249012
H	-0.790971	-0.390703	-1.336171	C	-2.192896	2.311933	1.090457
C	0.429752	-1.863893	1.475223	C	-2.946606	1.867252	-0.168146
H	1.866049	-0.968309	2.810826	C	-4.256267	1.168443	0.173356
H	-0.948411	-2.493374	-0.060814	C	-4.019234	0.003035	1.124763
H	0.353487	-2.788041	2.040955	C	-3.318970	0.478189	2.391133
C	1.465591	4.931210	-1.881856	C	-1.987211	1.157262	2.080612
C	2.856037	4.911550	-2.088846	H	3.288215	4.291701	-2.860078
C	3.712166	5.666513	-1.305279	H	4.780332	5.626597	-1.497515
C	3.213862	6.475945	-0.297743	H	3.888010	7.075366	0.304706
C	1.839709	6.522087	-0.058689	H	-0.559379	7.653257	-1.728290
C	0.945597	5.738994	-0.838095	H	-2.959311	8.120960	-1.372676
C	-0.515988	5.867533	-0.556303	H	-4.310427	6.536350	0.018119
C	-1.151153	6.988992	-1.104114	H	-3.295499	4.530988	0.893949
C	-2.502679	7.245952	-0.920325	H	0.303069	2.303772	2.505552
C	-3.253930	6.360848	-0.159590	H	2.229120	3.254392	1.225210
C	-2.655775	5.213947	0.349912	H	1.723882	4.858619	1.714153

H	2.521398	2.598587	3.616304	N	0.653889	4.034162	-2.679058
H	3.496163	4.016115	3.253688	N	1.336941	7.298362	1.041477
H	2.263743	4.255081	5.430975	C	-0.730712	4.433725	-3.017442
H	1.810759	5.499852	4.278745	H	-0.768173	5.438792	-3.456712
H	0.209430	2.973977	4.907182	H	-1.099631	3.721036	-3.754919
H	-0.218788	4.619020	5.356336	H	-1.401925	4.389226	-2.167099
H	-0.435457	5.234537	2.939665	C	1.287194	3.654002	-3.960205
H	-1.488027	3.880212	3.344272	H	1.452950	4.539200	-4.589397
H	-2.820107	3.030532	1.633281	H	2.217922	3.117714	-3.810607
H	-3.129107	2.730402	-0.822599	H	0.614836	2.968956	-4.475762
H	-2.298726	1.184790	-0.736210	C	0.899614	8.646713	0.684704
H	-4.939730	1.889897	0.645849	H	0.449012	9.116298	1.564352
H	-4.748935	0.830932	-0.745020	H	1.744095	9.279707	0.358961
H	-4.964537	-0.492931	1.370871	H	0.155428	8.632597	-0.107143
H	-3.390323	-0.748992	0.625195	C	2.268406	7.426455	2.157386
H	-3.974712	1.184663	2.921813	H	3.072205	8.161035	1.975482
H	-3.145923	-0.358862	3.076895	H	1.711581	7.765141	3.037348
H	-1.291390	0.415225	1.682464	H	2.735819	6.467564	2.38979
H	-1.550273	1.533800	3.012755				

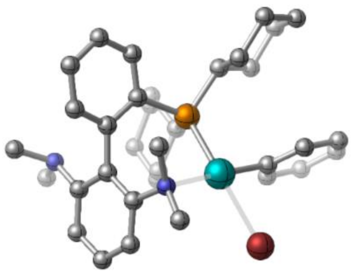

Zero-point correction= 0.735705 (Hartree/Particle)

Thermal correction to Energy= 0.767163

Thermal correction to Enthalpy= 0.767996

Thermal correction to Gibbs Free Energy= 0.676935

Sum of electronic and zero-point Energies= -1913.351679

Sum of electronic and thermal Energies= -1913.320220

Sum of electronic and thermal Enthalpies= -1913.319387

Sum of electronic and thermal Free Energies= -1913.410448

RM06L-D3/6-311g(d,p)-SDD(Pd,Br)-CPCM(THF)//RM06L-D3/6-31g(d,p)-LANL2DZ(Pd,Br)-CPCM(THF) =-1915.799622

14b-int1

E(scf) = -2591.53255957 a.u.

ν_{min} = 15.87 cm^{-1}

Br	-3.535621	-1.035735	-2.993636	H	-2.056137	-4.649347	-1.629573
Mg	-1.342978	-1.888991	-1.989532	H	-1.851059	-2.869862	-4.838231
H	-0.716723	-5.698318	-5.045123	H	-3.358394	-4.996193	-3.665035
H	-0.655417	-5.505766	-2.315585	Pd	-0.285937	1.210895	-1.415637
H	-0.141540	-3.364250	-4.947763	C	1.423303	0.990048	-0.430322
C	-1.578045	-5.037656	-4.902348	C	2.516540	1.689504	-0.930134
C	-1.519340	-4.873745	-2.555617	C	1.550503	0.113335	0.640953
H	-2.486003	-6.536698	-3.572438	C	3.762188	1.532374	-0.318137
O	-1.028184	-3.615741	-3.091582	H	2.409239	2.346782	-1.786231
C	-1.121277	-3.635851	-4.547076	C	2.807586	-0.043013	1.233799
C	-2.364100	-5.455886	-3.664576	H	0.701924	-0.448226	1.021062
H	-2.170389	-5.054220	-5.818950	C	3.908154	0.673911	0.770606

H	4.613749	2.090979	-0.702731
H	2.908194	-0.730989	2.070640
Br	0.559616	-0.522808	-3.338150
C	-0.798311	-2.388907	0.006672
C	-1.470469	-1.979424	1.176890
C	0.442920	-3.022174	0.231892
C	-0.938841	-2.132308	2.460610
H	-2.452987	-1.512494	1.091520
C	0.990272	-3.203037	1.504150
H	1.029050	-3.373159	-0.622866
C	0.306742	-2.738009	2.627368
H	-1.495098	-1.782903	3.330043
H	0.734917	-2.854082	3.620662
H	4.876610	0.556662	1.248382
H	1.958538	-3.687678	1.622322
C	0.178963	3.900543	-3.086990
C	1.000718	3.424654	-4.120211
C	2.361358	3.692959	-4.113635
C	2.932356	4.428352	-3.085517
C	2.148222	4.877551	-2.011726
C	0.753056	4.593867	-1.993475
C	-0.097095	5.199341	-0.930107
C	-0.068995	6.605248	-0.873456
C	-0.823183	7.330648	0.035374
C	-1.650512	6.655481	0.927772
C	-1.693522	5.269163	0.894758
C	-0.922474	4.512233	-0.009273
P	-1.104879	2.686211	0.174943

C	-0.592465	2.271465	1.924207
C	0.779769	2.841933	2.296665
C	1.279275	2.219811	3.596605
C	0.294888	2.446598	4.735966
C	-1.094181	1.948407	4.358781
C	-1.583092	2.578839	3.056172
C	-2.944155	2.452452	0.217247
C	-3.648905	2.944453	-1.042438
C	-5.159505	2.805389	-0.888752
C	-5.556977	1.366753	-0.578799
C	-4.816067	0.831720	0.642162
C	-3.306207	0.995342	0.500431
H	0.570375	2.878085	-4.950364
H	2.984442	3.329922	-4.926377
H	3.999797	4.615866	-3.091317
H	0.566820	7.125012	-1.583731
H	-0.775589	8.415591	0.037129
H	-2.262292	7.198019	1.641898
H	-2.352524	4.762425	1.592145
H	-0.495191	1.174376	1.869401
H	1.508382	2.681431	1.496367
H	0.680002	3.931217	2.423193
H	1.411534	1.138765	3.436400
H	2.269107	2.619172	3.846263
H	0.643325	1.955816	5.651347
H	0.242173	3.521975	4.960173
H	-1.069707	0.855543	4.234252
H	-1.812148	2.153613	5.160389

H	-1.659584	3.665619	3.195627
H	-2.590341	2.214347	2.831898
H	-3.305033	3.064238	1.058229
H	-3.371547	3.984169	-1.248394
H	-3.303494	2.346413	-1.898129
H	-5.496314	3.46644	-0.076028
H	-5.660365	3.155538	-1.798789
H	-6.639947	1.292503	-0.428821
H	-5.311742	0.736052	-1.443824
H	-5.149666	1.369457	1.542794
H	-5.058137	-0.225457	0.805321
H	-2.941864	0.374273	-0.332597
H	-2.795749	0.630453	1.400174
N	-1.235127	3.721295	-3.15247
C	-1.689665	2.739343	-4.128589
H	-2.753386	2.547422	-3.960825
H	-1.574215	3.080465	-5.171737
H	-1.144082	1.797333	-4.013264
C	-1.984103	4.96202	-3.357458
H	-1.77441	5.695011	-2.578109
H	-1.756941	5.415808	-4.336589
H	-3.053782	4.734331	-3.332156
N	2.73875	5.639472	-0.980952
C	2.65605	5.123901	0.375657
H	3.381385	4.311959	0.556906
H	2.861321	5.932093	1.085492
H	1.662109	4.736527	0.585663
C	4.053042	6.186603	-1.244864
H	4.85658	5.428862	-1.231607
H	4.071961	6.690108	-2.214061
H	4.283424	6.922189	-0.469143

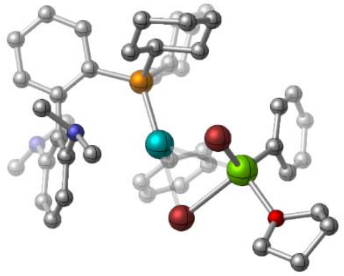

Zero-point correction= 0.945054 (Hartree/Particle)

Thermal correction to Energy= 0.989817

Thermal correction to Enthalpy= 0.990650

Thermal correction to Gibbs Free Energy= 0.868791

Sum of electronic and zero-point Energies= -2590.587505

Sum of electronic and thermal Energies= -2590.542743

Sum of electronic and thermal Enthalpies= -2590.541910

Sum of electronic and thermal Free Energies= -2590.663768

RM06L-D3/6-311g(d,p)-SDD(Pd,Br)-CPCM(THF)//RM06L-D3/6-31g(d,p)-LANL2DZ(Pd,Br)-CPCM(THF) =-2593.571264

TS1-14b

E(scf) = -2591.53440912 a.u.

ν_{min} = -36.55 cm^{-1}

Br	-2.891454	-0.268646	-2.590419	C	1.160194	0.901513	-0.413196
Mg	-1.165162	-2.202114	-2.206364	C	2.028739	1.349709	-1.403147
H	2.370068	0.649056	-4.140895	C	1.637577	0.329553	0.760050
H	2.275724	-1.206811	-1.978309	C	3.407278	1.216470	-1.215349
H	-0.023575	0.701746	-3.934061	H	1.640394	1.776355	-2.323284
C	1.864354	-0.191567	-4.628922	C	3.016940	0.204713	0.940230
C	1.751677	-1.701646	-2.803048	H	0.951799	-0.038329	1.520647
H	3.523253	-1.508943	-4.044970	C	3.902115	0.652324	-0.041004
O	0.448384	-1.052815	-2.946837	H	4.082885	1.565976	-1.994645
C	0.422946	-0.269125	-4.178074	H	3.391494	-0.251460	1.854277
C	2.435405	-1.517134	-4.140470	Br	-0.929547	-4.266168	-3.665382
H	1.948499	-0.052917	-5.708895	C	-0.707261	-2.038115	-0.100815
H	1.581535	-2.753237	-2.550255	C	-1.594506	-1.471656	0.840178
H	-0.217471	-0.790103	-4.900855	C	0.427120	-2.666867	0.459777
H	2.157239	-2.329065	-4.820820	C	-1.370869	-1.502898	2.220883
Pd	-0.724707	0.735798	-0.987198	H	-2.507492	-0.994574	0.480225

C	0.660875	-2.732763	1.833531	C	-5.305174	3.495521	-0.684080
H	1.164616	-3.134422	-0.197536	C	-5.979379	2.182007	-0.305303
C	-0.233110	-2.132984	2.722832	C	-5.318907	1.558945	0.919213
H	-2.087055	-1.043628	2.902479	C	-3.823088	1.369650	0.688032
H	-0.048246	-2.161661	3.794514	H	-0.017749	2.941807	-5.117957
H	4.973836	0.558867	0.109611	H	2.431239	3.040157	-5.323396
H	1.545116	-3.240259	2.215651	H	3.806870	3.955734	-3.500617
C	-0.062690	3.808539	-3.137055	H	1.035374	6.762660	-1.511688
C	0.571316	3.320353	-4.290691	H	0.043869	8.202765	0.228316
C	1.953294	3.397567	-4.414643	H	-1.573261	7.203735	1.864600
C	2.728154	3.927909	-3.396358	H	-2.093739	4.832914	1.742382
C	2.129358	4.356062	-2.198583	H	-0.764724	1.131507	2.037797
C	0.712046	4.293264	-2.057704	H	1.269197	2.595888	1.687328
C	0.062334	4.960910	-0.893922	H	0.453333	3.880136	2.567447
C	0.351392	6.335368	-0.783966	H	1.136451	1.092061	3.637986
C	-0.207104	7.146876	0.189866	H	1.990298	2.576032	4.041063
C	-1.104582	6.594444	1.097996	H	0.328710	1.930513	5.824401
C	-1.397027	5.241029	1.018368	H	-0.040315	3.497447	5.116845
C	-0.822961	4.391472	0.051342	H	-1.367502	0.842922	4.379995
P	-1.304982	2.614215	0.277002	H	-2.11438	2.153125	5.285368
C	-0.841741	2.230788	2.067228	H	-1.900747	3.656164	3.32181
C	0.529316	2.787332	2.465324	H	-2.856059	2.225578	2.956016
C	1.003857	2.176206	3.779665	H	-3.358139	3.392524	1.20532
C	0.002948	2.420226	4.900159	H	-3.331556	4.285597	-1.107463
C	-1.383606	1.936830	4.498415	H	-3.605817	2.66373	-1.735505
C	-1.843687	2.568007	3.184270	H	-5.479664	4.235077	0.111587
C	-3.164536	2.711367	0.361856	H	-5.751456	3.911912	-1.594889
C	-3.802194	3.323783	-0.879940	H	-7.049876	2.336073	-0.13031

H	-5.898752	1.479925	-1.14798
H	-5.475018	2.208363	1.794071
H	-5.782512	0.59618	1.160834
H	-3.677841	0.693555	-0.162358
H	-3.351067	0.885282	1.550901
N	-1.483901	3.886088	-3.074919
C	-2.181624	3.047015	-4.033955
H	-3.247188	3.042497	-3.785702
H	-2.086706	3.40191	-5.075196
H	-1.821056	2.017757	-3.971816
C	-1.997641	5.251685	-3.179994
H	-1.597382	5.894902	-2.395236
H	-1.761227	5.704151	-4.158232
H	-3.086837	5.226313	-3.076854
N	2.927367	4.855947	-1.168325
C	2.721698	4.393376	0.192764
H	3.614595	3.865781	0.555383
H	2.496038	5.215383	0.886194
H	1.897782	3.683242	0.22039
C	4.30123	5.19894	-1.454854
H	4.960768	4.318865	-1.54983
H	4.370079	5.781948	-2.376233
H	4.685731	5.813627	-0.636672

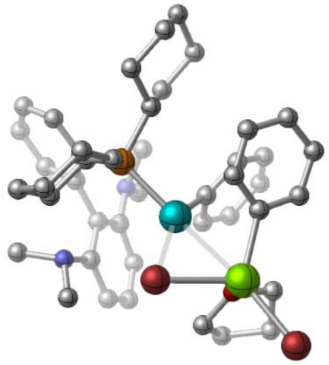

Zero-point correction= 0.946108 (Hartree/Particle)

Thermal correction to Energy= 0.989589

Thermal correction to Enthalpy= 0.990422

Thermal correction to Gibbs Free Energy= 0.873740

Sum of electronic and zero-point Energies= -2590.588301

Sum of electronic and thermal Energies= -2590.544820

Sum of electronic and thermal Enthalpies= -2590.543987

Sum of electronic and thermal Free Energies= -2590.660669

RM06L-D3/6-311g(d,p)-SDD(Pd,Br)-CPCM(THF)//RM06L-D3/6-31g(d,p)-LANL2DZ(Pd,Br)-CPCM(THF) =-2593.570585

5. References

(i) King, R. P.; Krska, S. W.; Buchwald, S. L. A Neophyl Palladacycle as an Air- and Thermally Stable Precursor to Oxidative Addition Complexes. *Org. Lett.* **2021**, *23*, 7927–7932.
(ii) Perego, L. A.; Grimaud, L.; Bellina, F. Mechanistic Studies on the Palladium-Catalyzed Direct C-5 Arylation of Imidazoles: The Fundamental Role of the Azole as a Ligand for Palladium. *Adv. Synth. Catal.* **2016**, *358*, 597–609.
(iii) Molloy, J. J.; Seath, C. P.; West, M. J.; McLaughlin, C.; Fazakerley, N. J.; Kennedy, A. R.; Nelson, D. J.; Watson, A. J. B. Interrogating Pd(II) Anion Metathesis Using a Bifunctional Chemical Probe: A Transmetalation Switch. *J. Am. Chem. Soc.* **2017**, *140*, 126–130.
(iv) Milner, P. J.; Maimone, T. J.; Su, M.; Chen, J.; Müller, P.; Buchwald, S. L. Investigating the Dearomative Rearrangement of Biaryl Phosphine-Ligated Pd(II) Complexes. *J. Am. Chem. Soc.* **2012**, *134*, 19922–19934.
(v) Neukom, J. D.; Perch, N. S.; Wolfe, J. P. Intramolecular Insertion of Alkenes into Pd−N Bonds. Effects of Substrate and Ligand Structure on the Reactivity of (P−P)Pd(Ar)[N(Ar1)(CH2)3CR═CHR′] Complexes. *Organometallics* **2011**, *30*, 1269–1277.
(vi) Wang, J. Y.; Strom, A. E.; Hartwig, J. F. Mechanistic Studies of Palladium-Catalyzed Aminocarbonylation of Aryl Chlorides with Carbon Monoxide and Ammonia. *J. Am. Chem. Soc.* **2018**, *140*, 7979–7993.
(vii) (a) Bryan, Z. J.; Smith, M. L.; McNeil, A. J. Chain-Growth Polymerization of Aryl Grignards Initiated by a Stabilized NHC-Pd Precatalyst. *Macromol. Rapid Commun.* **2012**, *33*, 842–847. (b) Love, B. E.; Jones, E. G. The Use of Salicylaldehyde Phenylhydrazone as an Indicator for the Titration of Organometallic Reagents. *J. Org. Chem.***1999**, *64*, 3755–3756.
(viii) Zhao, Y.; Truhlar, D. G. A new local density functional for main-group thermochemistry, transition metal bonding, thermochemical kinetics, and noncovalent interactions. *J. Chem. Phys.* **2006**, *125*, 194101.
(ix) (a) Grimme, S. Accurate description of van der Waals complexes by density functional theory including empirical corrections. *J. Comput. Chem.* **2004**, *25*, 1463–1473. (b) Grimme, S.; Antony, J.; Ehrlich, S.; Krieg, H. A consistent and accurate ab initio parametrization of density functional dispersion correction (DFT-D) for the 94 elements H-Pu. *J. Chem. Phys.* **2010**, *132*, 154104. (c) Grimme, S. Density functional theory with London dispersion corrections. *WIRES Comput. Mol. Sci.* **2011**, *1*, 211-228. (d) Ehrlich, S.; Moellmann, J.; Grimme, S. Dispersion-Corrected Density Functional Theory for Aromatic Interactions in Complex Systems. *Acc. Chem. Res.* **2013**, *46*, 916–926.
(x) (a) Petersson, G. A.; Tensfeldt, T. G.; Montgomery, J. A., Jr. A Complete Basis Set Model Chemistry. III. The Complete Basis Set-quadratic Configuration Interaction Family of Methods. *J. Chem. Phys.* **1991**, *94*, 6091–6101. (b) Petersson, G. A.; Bennett, A.; Tensfeldt, T. G.; Al-Laham,

M. A.; Shirley, W. A.; Mantzaris, J. A Complete Basis Set Model Chemistry. I. The Total Energies of Closed-shell Atoms and Hydrides of the First-row Elements. *J. Chem. Phys.* **1988**, *89*, 2193–2218.
(xi) (a) T. H. Dunning Jr. and P. J. Hay, in *Modern Theoretical Chemistry*, Ed. H. F. Schaefer III, Vol. 3 (Plenum, New York, 1977) 1-28. (b) (1) Hay, P. J.; Wadt, W. R. Ab initio effective core potentials for molecular calculations. Potentials for the transition metal atoms Sc to Hg. The *J. Chem. Phys.* **1985**, *82*, 270–283. (c) (1) Wadt, W. R.; Hay, P. J. Ab initio effective core potentials for molecular calculations. Potentials for main group elements Na to Bi. *J. Chem. Phys.* **1985**, *82*, 284–298. (d) Hay, P. J.; Wadt, W. R. Ab initio effective core potentials for molecular calculations. Potentials for K to Au including the outermost core orbitals. J. Chem. Phys. **1985**, *82*, 299–310.
(xii) Gaussian 16, Revision C.01, Frisch, M. J.; Trucks, G. W.; Schlegel, H. B.; Scuseria, G. E.; Robb, M. A.; Cheeseman, J. R.; Scalmani, G.; Barone, V.; Petersson, G. A.; Nakatsuji, H.; Li, X.; Caricato, M.; Marenich, A. V.; Bloino, J.; Janesko, B. G.; Gomperts, R.; Mennucci, B.; Hratchian, H. P.; Ortiz, J. V.; Izmaylov, A. F.; Sonnenberg, J. L.; Williams-Young, D.; Ding, F.; Lipparini, F.; Egidi, F.; Goings, J.; Peng, B.; Petrone, A.; Henderson, T.; Ranasinghe, D.; Zakrzewski, V. G.; Gao, J.; Rega, N.; Zheng, G.; Liang, W.; Hada, M.; Ehara, M.; Toyota, K.; Fukuda, R.; Hasegawa, J.; Ishida, M.; Nakajima, T.; Honda, Y.; Kitao, O.; Nakai, H.; Vreven, T.; Throssell, K.; Montgomery, J. A., Jr.; Peralta, J. E.; Ogliaro, F.; Bearpark, M. J.; Heyd, J. J.; Brothers, E. N.; Kudin, K. N.; Staroverov, V. N.; Keith, T. A.; Kobayashi, R.; Normand, J.; Raghavachari, K.; Rendell, A. P.; Burant, J. C.; Iyengar, S. S.; Tomasi, J.; Cossi, M.; Millam, J. M.; Klene, M.; Adamo, C.; Cammi, R.; Ochterski, J. W.; Martin, R. L.; Morokuma, K.; Farkas, O.; Foresman, J. B.; Fox, D. J. Gaussian, Inc., Wallingford CT, 2016.
(xiii) Tomasi, J.; Mennucci, B.; Cammi, R. Quantum mechanical continuum solvation models. *Chem. Rev.* **2005**, *105*, 2999-3093.
(xiv) Legault, C. Y. (2009) CYLview, 1.0b, Universite de Sherbrooke: Sherbrooke, Canada, http://www.cylview.org.
(xv) NBO Version 3.1, E. D. Glendening, A. E. Reed, J. E. Carpenter, and F. Weinhold.
(xvi) Lu, T.; Chen, F. Multiwfn: A multifunctional wavefunction analyzer. *J. Comp. Chem.* **2011**, 33, 580–592.
